# Charged molecular glue discovery enabled by targeted degron display

**DOI:** 10.1038/s41589-026-02182-5

**Published:** 2026-04-06

**Authors:** Zhe Zhuang, Woong Sub Byun, Jakub Chrustowicz, Zuzanna Kozicka, Veronica L. Li, Dinah M. Abeja, Katherine A. Donovan, Sara Sepic, Inchul You, Mikołaj Słabicki, Eric S. Fischer, Stephen M. Hinshaw, Benjamin L. Ebert, Brenda A. Schulman, Nathanael S. Gray

**Affiliations:** 1 Department of Chemical and Systems Biology, ChEM-H, and Stanford Cancer Institute, Stanford School of Medicine, Stanford University, Stanford, CA USA; 2 College of Pharmacy, Dongguk University-Seoul, Goyang, Republic of Korea; 3 Department of Molecular Machines and Signaling, Max Planck Institute of Biochemistry, Martinsried, Germany; 4 Department of Medical Oncology, Dana-Farber Cancer Institute, Boston, MA USA; 5 Broad Institute of MIT and Harvard, Cambridge, MA USA; 6 Department of Pathology, Stanford School of Medicine, Department of Chemistry, ChEM-H, and Wu Tsai Human Performance Alliance, Stanford University, Stanford, CA USA; 7 Department of Cancer Biology, Dana-Farber Cancer Institute, Boston, MA USA; 8 Department of Biological Chemistry and Molecular Pharmacology, Harvard Medical School, Boston, MA USA; 9 Department of Chemistry, School of Natural Sciences, Technical University of Munich, Garching, Germany; 10 Krantz Family Center for Cancer Research, Massachusetts General Hospital Cancer Center, Boston, MA USA

**Keywords:** Small molecules, Structural biology, Post-translational modifications

## Abstract

Small molecules that induce protein interactions hold tremendous potential as new medicines, probes for molecular pathways and tools for agriculture. Explosive growth of targeted protein degradation drug development has spurred renewed interest in proximity-inducing molecules, especially molecular glue degraders (MGDs). These compounds catalyze the destruction of disease-causing proteins by reshaping protein surfaces and promoting cooperative binding between ubiquitylating enzymes and target proteins. MGD discovery for predefined targets is a major challenge in contemporary drug discovery. Here, we solve this important chemical challenge through ‘chemocentric’ MGD discovery of ZZ1, a BET-family protein degrader and a prodrug of a negatively charged glue. ZZ1 activation unmasks a sulfinic acid that binds the modular CTLH ubiquitin ligase complex through a basic pocket in its YPEL5 subunit. These findings demonstrate a previously unrecognized capacity of YPEL5 to recruit CTLH substrates and enable the discovery of MGDs for exceedingly common acidic and basic degrons.

**Figure:**
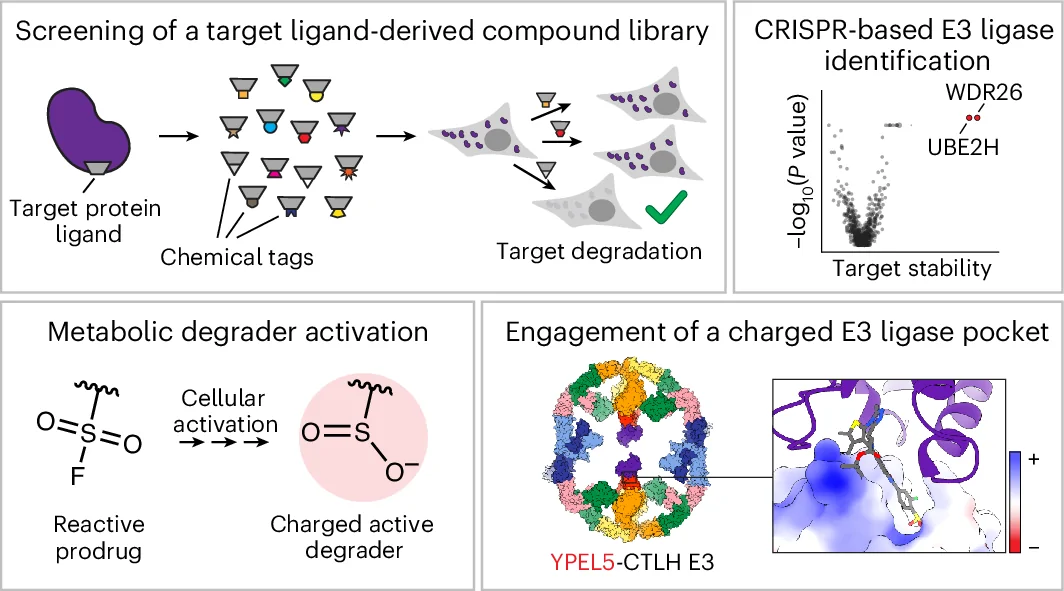

## Abstract (web summary)

The study reveals a ‘chemocentric’ strategy for identifying charged molecular glue degraders, through discovering a bromodomain-binding molecular glue degrader prodrug that is metabolically activated in cells to recruit the YPEL5-CTLH E3 ligase.

## Main

Discovery of small molecules that induce binding between two or more target proteins is an important challenge with major implications for medicine, agriculture and basic biology^1–5^. Molecular glue molecules embody this concept. These are small molecules that bind specific targets to generate composite protein–small molecule surfaces that mediate interactions with proteins that would not otherwise bind the target alone^6^. Such molecules are valuable because they can produce unexpected pharmacology by redirecting enzymatic activities to new substrates. A particularly compelling class of such moieties are molecular glue degraders (MGDs), which promote binding between ubiquitylating enzymes and so-called ‘neosubstrates’, ultimately resulting in targeted protein degradation (TPD)^2^. MGDs have drug-like properties, induce TPD at low compound concentrations by acting catalytically, do not require preexisting ligands for both recruited proteins and can recruit partners without drug-binding pockets^1,7,8^.

Chemical elaboration of known MGDs has enabled refocusing their activities on distinct neosubstrate targets^9–11^. This is an especially important approach in the MGD space, because the ubiquitin ligase–MGD ‘neosurface’ (rather than the compound alone) determines which neosubstrate surfaces bind and how these proteins are presented to ubiquitylation active sites^12^. In a few cases, cryptic target depletion by potent small-molecule inhibitors has led to the discovery of new MGDs^13–16^. Both strategies are limited to degrader discovery; they are not generalizable to glue molecules with other activities or applicable for developing new molecular glues for predefined target proteins. Furthermore, most MGDs bind ubiquitylating enzymes in the Cullin–RING ligase (CRL) family that are broadly expressed and not required for cell proliferation. The dual consequences are drug resistance by ligase downregulation and poor therapeutic indices because of systemic TPD activity^17^. Discovery of new MGDs that use previously undrugged ubiquitylating enzymes is a prominent challenge in contemporary chemical biology^18–20^. Furthermore, different ligase–MGD pairs may bind distinct neosubstrate surfaces on the same target protein. A consequence of this is that each ligase–MGD pair engages an idiosyncratic and unpredictable assortment of neosubstrate proteins and, therefore, offers unique off-target and, ultimately, genetic or epigenetic drug resistance profiles.

The first MGD discovered, auxin, contains a key carboxyl group that directly interacts with an arginine residue in its ubiquitin ligase partner. Auxin is subject to active transport across cell membranes of diverse eukaryotes. However, because the plasma membrane presents a barrier to most charged molecules, current approaches to MGD discovery exclude the large fraction of E3 ligases that bind charged clients. Yet, many of the first E3 ligase families discovered degrade cellular substrates with positive or negative charges. Early examples include N-end-rule degrons^21–23^ and phosphodegrons^24,25^. Relatively more recently identified E3 ligases recognize C-terminal degrons^26–28^ or clusters of acidic or basic residues^29,30^. Participation of these charged E3 ligase systems in cell-cycle signaling, protein quality control, metabolism and cellular differentiation indicates their physiologic prevalence and importance^21–31^.

MGDs bearing complementary charges are needed to access E3 ligase systems dependent on electrostatically driven substrate binding. Such molecules have not emerged from previous MGD discovery campaigns. This is largely because of difficulties in developing cell-permeable charged small molecules, which is a recurring and general challenge in medicinal chemistry. We and others have shown that subtle chemical modifications can endow gain-of-function MGD properties upon existing chemical ligands^32,33^. Molecules discovered by this ‘chemocentric’ strategy primarily target the CRL family. High-resolution structures of ligase–small molecule–neosubstrate ternary complexes are essential for targeted MGD diversification. Except in rare cases^15^, molecular structures have not been reported for most MGDs discovered using this strategy.

Here, we used a target-focused reactive chemical screening strategy, coupled with counterscreening, to identify a protein degradation mechanism that has not yet been exploited for TPD. The resultant and the rationally improved compounds are metabolically activated, target the previously undrugged YPEL5-CTLH E3 complex, and are charged glue (c-Glue) small molecules promoting electrostatically induced proximity.

## Results

### Discovery of a YPEL5-CTLH-dependent bromodomain (BD)-containing protein 4 (BRD4) degrader

**Fig. 1:**
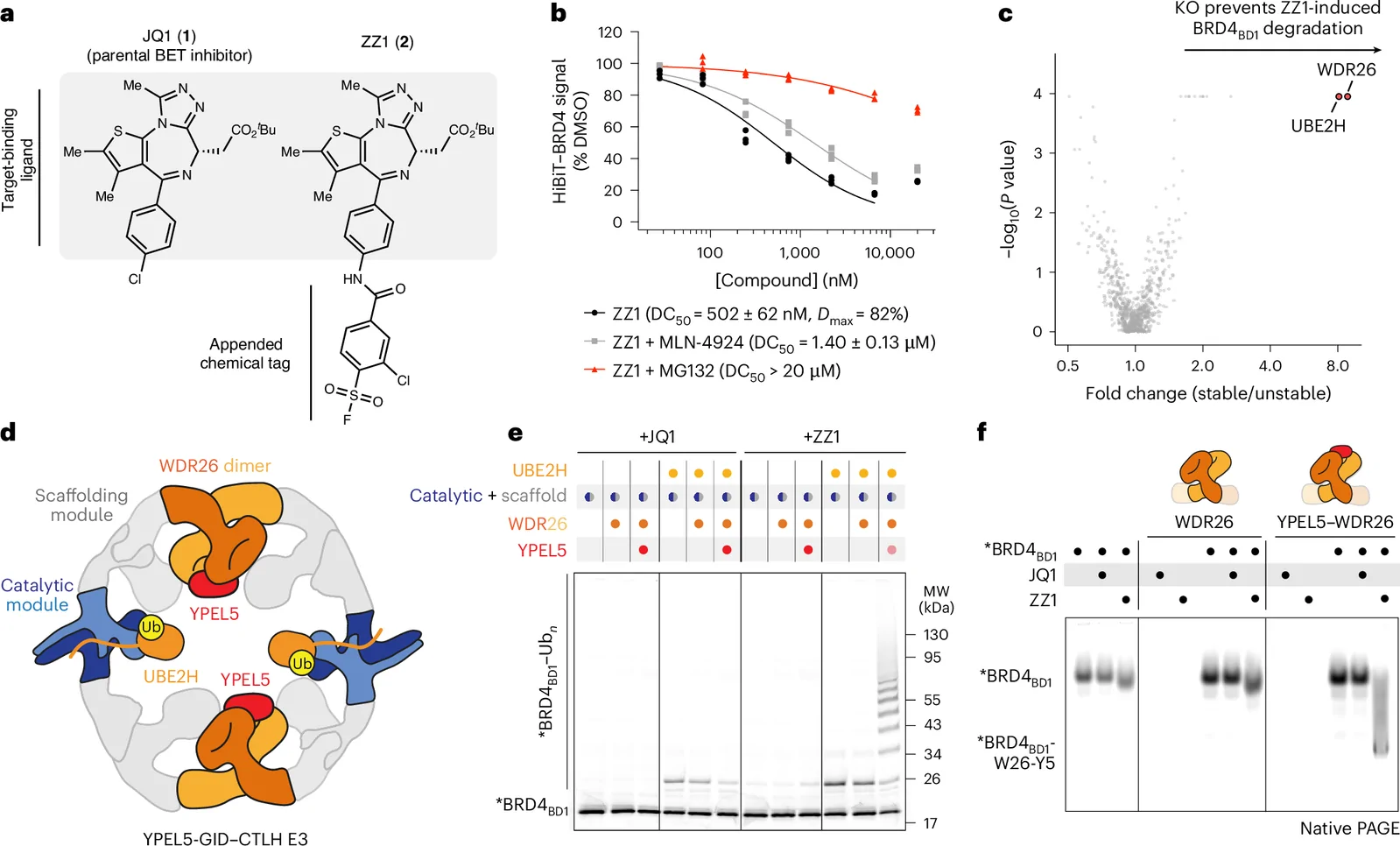
Chemocentric approach yields an MGD for a predefined target. **a**, Chemical structures of the parental BET protein family inhibitor (JQ1) and its derivative (ZZ1) featuring an appended chemical tag conferring degrader activity. **b**, HiBiT–BRD4 assay results for Jurkat cells pretreated with the indicated inhibitors for 1 h, followed by treatment with ZZ1 for 5 h (mean ± s.d.; *n* = 3). **c**, UPS-focused CRISPR screen for BRD4_BD1_–eGFP stability in K562-Cas9 cells treated with 1 µM ZZ1 for 16 h. Guide counts were collapsed to gene level (*n* = 4 guides per gene; two-sided empirical rank-sum test statistics). **d**, Cartoon of the UBE2H~ubiquitin (Ub)-bound YPEL5-CTLH E3 ligase catalytic assembly highlighting its functional modules. **e**, In vitro ubiquitylation assay of fluorescently labeled *BRD4_BD1_ (asterisk denotes an N-terminal FAM label) determining the minimal catalytic assembly sufficient for ZZ1-dependent activity. The examined CTLH E3 ligases comprised the catalytic core (catalytic and scaffolding modules) alone or assembled with substrate regulatory subunits WDR26 and YPEL5. Reactions were quenched after 45 min. Details of the CTLH E3 ligase architecture and assays with a full suite of CTLH E3 assemblies are presented in Extended Data Figs. 2 and 3, respectively. **f**, Native gel mobility shift assay probing ZZ1-induced *BRD4_BD1_ engagement by WDR26 and the YPEL5–WDR26 complex. Transparent regions in the cartoon represent truncated WDR26 domains (the truncations prevent higher-order oligomerization). Source Data

**Extended Data Fig. 1:**
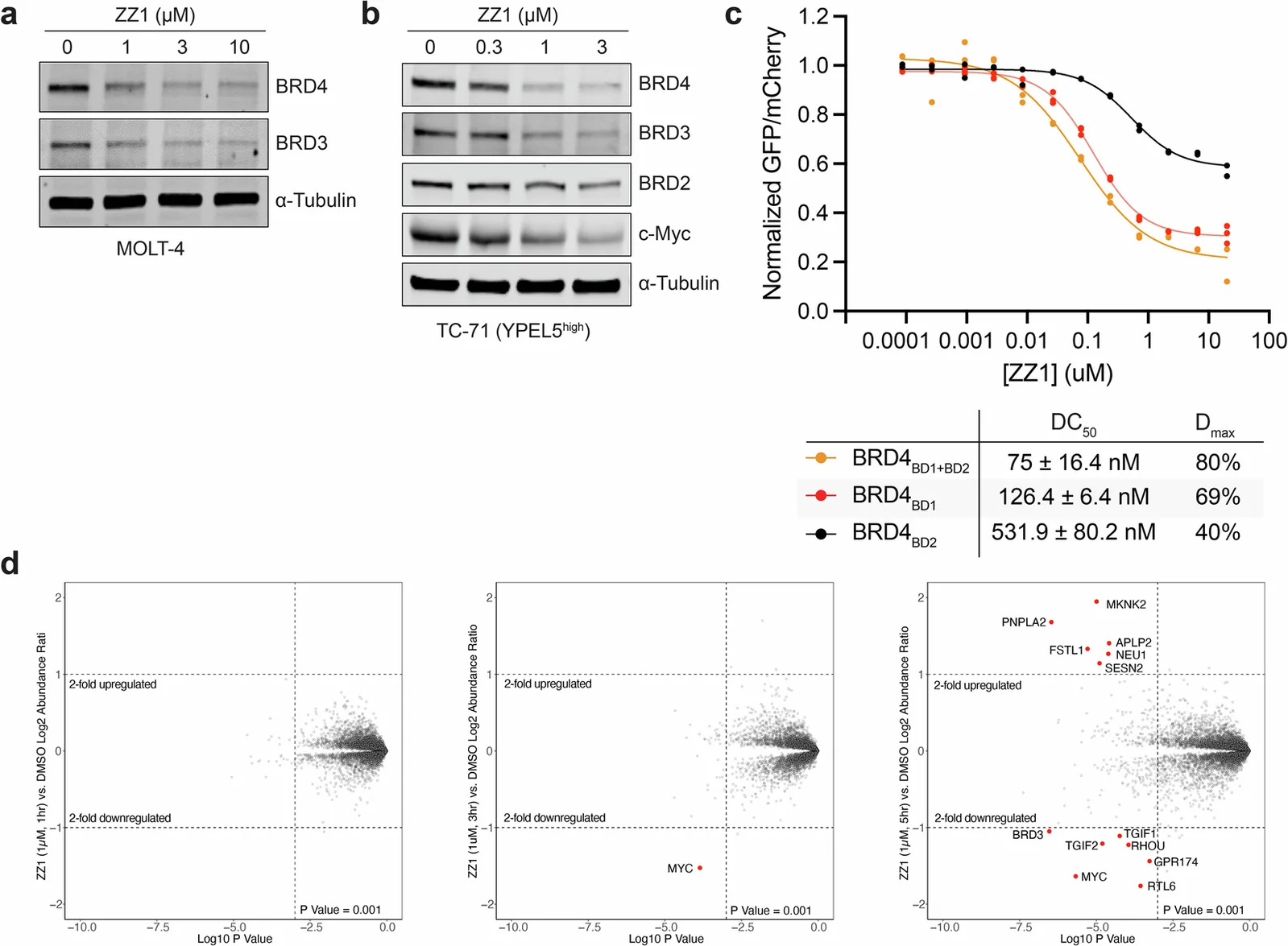
Determining neosubstrate selectivity of ZZ1-induced degradation. **a** Western blots showing BRD4 and BRD3 degradation in MOLT-4 cells after 5 h treatment with ZZ1. **b** Western blots showing BRD2, BRD3, and BRD4 degradation in TC-71 cells after 5 h treatment with ZZ1. **c** Identifying the bromodomain required for ZZ1-induced BRD4 degradation with a cellular fluorescent reporter assay. The examined reporters were either the isolated BRD4 bromodomains (BD1 or BD2) or a tandem construct comprising BD1 and BD2 connected by the intervening native sequence (BRD4_BD1+BD2_) (SD, n = 2). **d** Quantitative proteome-wide mass spectrometry in MOLT-4 cells after 1 h, 3 h, or 5 h treatment with 1 µM ZZ1. Significant changes were assessed by a two-sided moderated t-test as implemented in the Lima package. All Western blot experiments were independently repeated at least twice with similar results. Source Data

To identify MGDs that exploit novel E3 ligases, we used a degron display approach whereby we functionalized a known high-affinity ligand for a target protein with a library of chemical tags containing covalent-acting warheads (Supplementary Table 1). We chose the bromodomain (BD) and extraterminal domain (BET) family of epigenetic readers as model targets and we used the BET BD inhibitor JQ1 (**1**) as a parental substrate-recruiting handle^34,35^. This BRD4–JQ1 system is an established system for the discovery of monovalent glue degraders^15,16,32^. We detected BRD4 degradation using a HiBiT assay based on CRISPR–Cas9-mediated fusion of an 11-residue peptide tag to BRD4 (ref. ^32^). A relatively simple chemical tag appended to JQ1 yielded a functional degrader that we refer to as ZZ1 (**2**) (Fig. 1a). ZZ1 features an amide-linked phenyl ring with chloro and sulfonyl fluoride^36^ groups at the *meta* and *para* positions, respectively. ZZ1 induced selective degradation of BET proteins (BRD3, BRD4 and, to a lesser extent, BRD2) as determined by immunoblotting and quantitative proteome-wide mass spectrometry (MS) (Extended Data Fig. 1a,b,d).

Evidence supporting a novel mechanism of action for ZZ1 surfaced during further examination of BRD4 destabilization. First, the impact of CRL inhibition (through NEDD8-activating enzyme inhibitor MLN-4924) did not mirror the preventative effect of proteasome inhibition on degradation (Fig. 1b). These data suggest the involvement of a novel non-CRL E3 ligase. Second, ZZ1 induced preferential degradation of a specific BD. We used a fluorescent reporter assay to identify the region of BRD4 responsible for ZZ1-induced degradation (Extended Data Fig. 1c)^15^. Despite the similar affinity of JQ1 to both BRD4 BDs, reporters containing BD1 (BRD4_BD1_ and the tandem construct containing both BD1 and BD2, BRD4_BD1+BD2_) were more potently degraded than BD2 alone. Thus, ZZ1 shows preference for targeting a distinct BD compared to most JQ1-based degraders^15,16,32,37^, implying the involvement of a previously unexplored E3 ligase.

**Extended Data Fig. 2:**
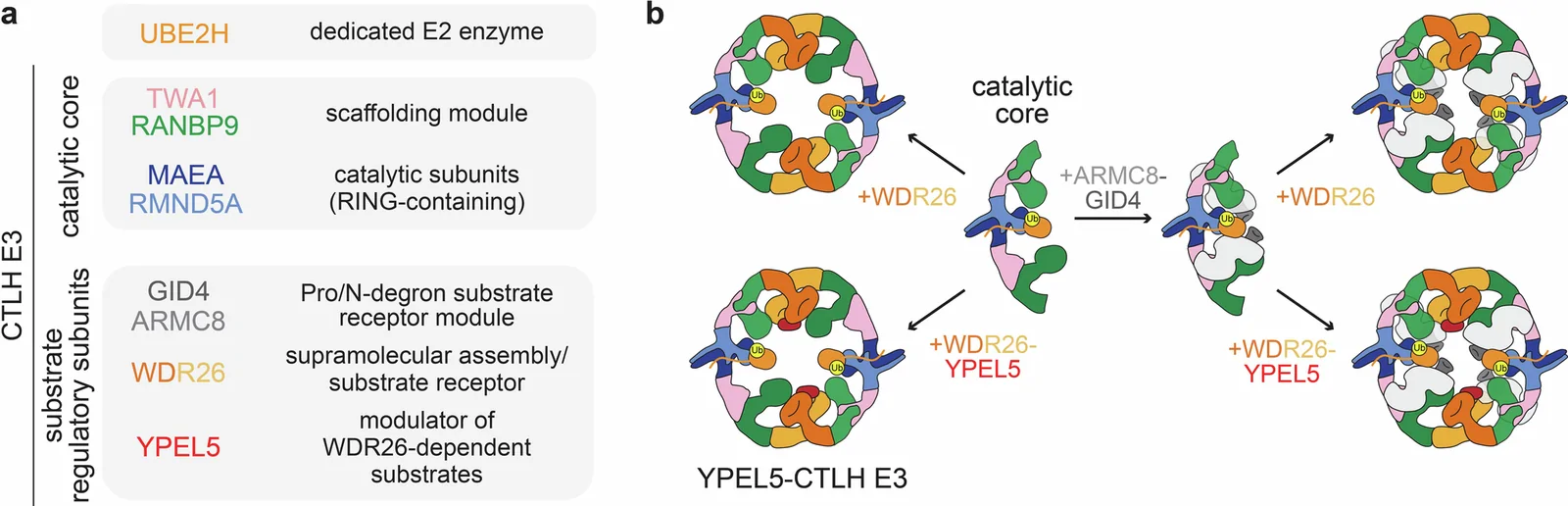
Color-coded guide and cartoon of the CTLH E3 ligase. **a**) Color-coded guide to the CTLH E3 subunits and their reported functions. **b**) Cartoon showing the CTLH E3 ligases. A common catalytic core associates with divergent regulatory and substrate binding subunts. WDR26 is a homodimer that connects two catalytic cores (alone or bound to ARMC8–GID4) into a giant oval structure. Each WDR26 homodimer in the supramolecular assembly can bind one YPEL5 subunit, yielding the YPEL5-CTLH E3. Subunits are colored as in (**a**).

**Extended Data Fig. 3:**
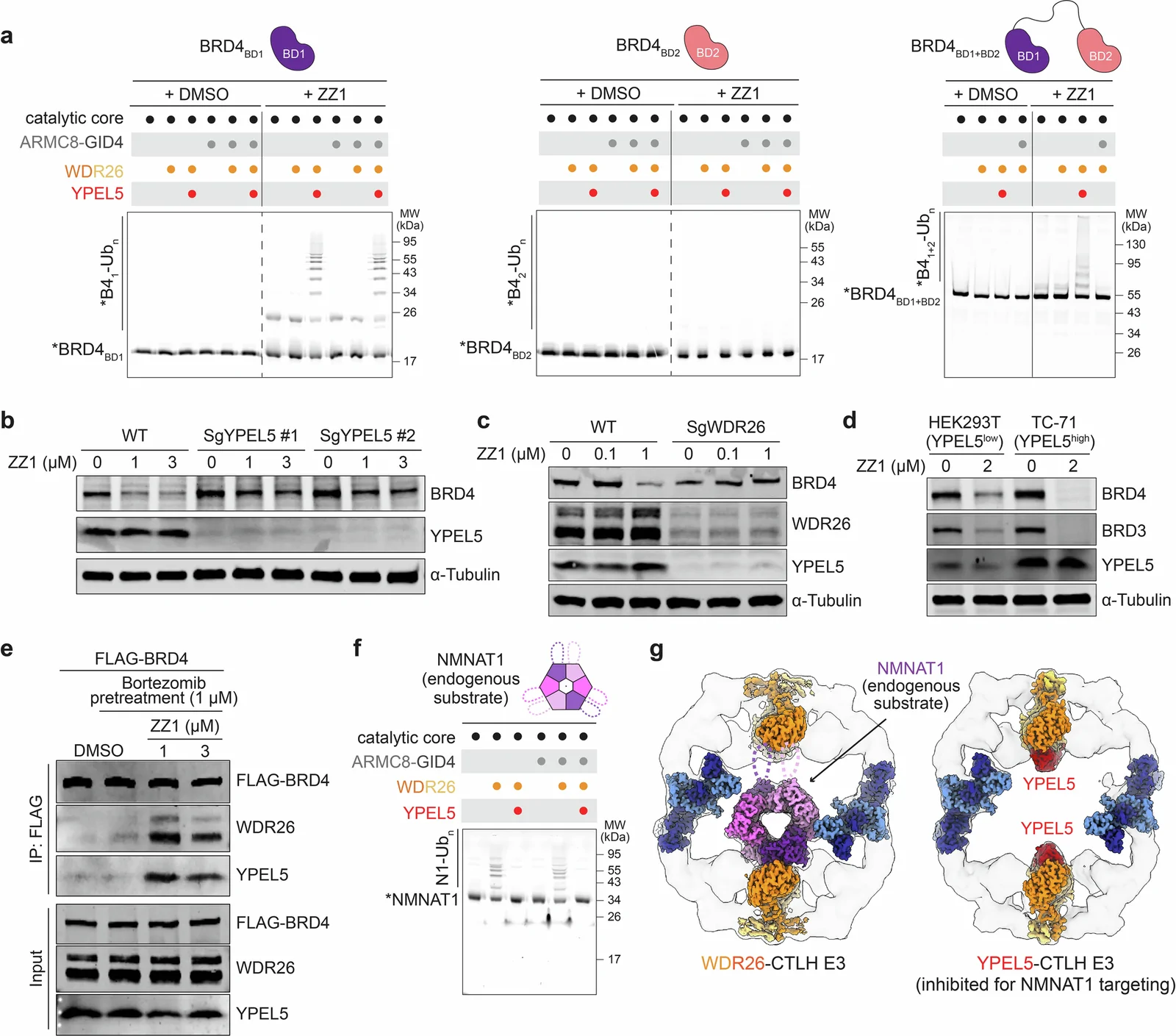
YPEL5-CTLH E3 ligase is an effector for ZZ1-mediated BRD4 targeting. **a**) *In vitro* ubiquitylation assays identifying the CTLH-E3 ligase leveraged by ZZ1. CTLH E3 assemblies shown in (Extended Data Fig. 2b) were tested for activity towards fluorescent BRD4 bromodomain substrates, either in isolation (BRD4_BD1_ and BRD4_BD2_) or in tandem (BRD4_BD1+BD2_). Asterisk denotes a fluorescent FAM label appended to the N-terminus of each substrate. Reactions were quenched after 45 min. **b**) Western blots showing BRD4 degradation in WT or YPEL5-KO Jurkat cells treated with the indicated concentration of ZZ1 for 5 h. **c**) Western blots showing BRD4 degradation in WT or WDR26-KO HEK293T cells treated with the indicated concentration of ZZ1 for 5 h. **d**) Western blots showing BRD4 degradation in HEK293T (with low level of YPEL5 expression: YPEL5^*low*^) or TC-71 (with elevated level of YPEL5 expression: YPEL5^*high*^) cells treated with 2 µM ZZ1 for 5 h. **e**) Co-immunoprecipitation of FLAG-tagged BRD4 and YPEL5-WDR26-CTLH E3 in the presence of ZZ1. FLAG-tagged BRD4 transfected HEK293T cells were preincubated with the proteasomal pathway inhibitor (bortezomib) for 1 h to prevent BRD4 degradation. **f**) *In vitro* ubiquitylation assay as in (a) but performed with the endogenous NMNAT1 substrate to recapitulate its previously reported CTLH E3-dependent regulation. In contrast to its essential role in ZZ1-induced BRD4 ubiquitylation, YPEL5 acts as an inhibitor of NMNAT1 targeting. Reactions were quenched after 45 min. **g**) Previous cryo-EM structures of NMNAT1- and YPEL5-bound CTLH E3 assemblies (EMD-18175 and EMD-18170, respectively)^42^ fit with segmented focused-refined maps of relevant modules (EMD-18345 and EMD-18316, respectively) explaining the biochemically defined mode of NMNAT1 targeting: YPEL5 and the oligomeric NMNAT1 substrate are engaged by the overlapping binding sites on the dimeric WDR26 modules. Consequently, YPEL5 sterically blocks WDR26-mediated NMNAT1 recruitment. All Western blot experiments were independently repeated at least twice with similar results.

To identify ubiquitylation factors required for ZZ1-induced BRD4_BD1_ degradation, we performed a fluorescence-activated cell sorting CRISPR screen, testing 713 genes involved in the ubiquitin–proteasome system (UPS)^13^ (Fig. 1c). The screen identified key components of the CTLH E3 ligase, including its cognate E2 enzyme, UBE2H (refs. ^38,39^), and its subunit, WDR26 (refs. ^40,41^) (Fig. 1d and Extended Data Fig. 2a). To determine whether the CTLH E3 can ubiquitylate BRD4, we reconstituted the ZZ1-mediated activity in vitro using purified proteins. CTLH is not a singular E3 ligase but a collection of assemblies with a common catalytic core and varying auxiliary subunits that control substrate selection^40,42^ (Extended Data Fig. 2a,b). Testing of all known CTLH auxiliary subunits enabled identification of a minimal assembly proficient to catalyze UBE2H-mediated BRD4_BD1_ ubiquitylation. This complex features WDR26 and its binding partner YPEL5 (Fig. 1e and Extended Data Fig. 3a). Accordingly, Cas9-mediated knockout of either WDR26 or YPEL5 prevented ZZ1-induced BRD4 degradation (Extended Data Figs. 2e and 3b). Consistent with this finding, ZZ1 is especially potent in the Ewing sarcoma TC-71 cell line, which has elevated YPEL5 expression^43^ (Extended Data Fig. 3d). A coimmunoprecipitation experiment using BRD4 as bait confirmed the presence of the YPEL5–WDR26 regulatory module in the CTLH E3 assembly that engages BRD4 upon cellular treatment with ZZ1 (Extended Data Fig. 3e). Thus, the CTLH complex containing both WDR26 and YPEL5 is necessary and sufficient for ZZ1-mediated BRD4 degradation.

Biochemical and genetic dependence on YPEL5 raised two questions. First, is YPEL5 a substrate receptor of the CTLH E3? The currently only known endogenous function of YPEL5 is to bind WDR26 and prevent it from recruiting its native substrate^44^ (Extended Data Fig. 3f,g). In contrast, YPEL5 appears to be responsible for degrader-induced recruitment of the neosubstrate, which is supported by ZZ1-dependent comigration of BRD4_BD1_ in a nondenaturing gel shift experiment (Fig. 1f). Second, YPEL5 shows striking structural homology to the thalidomide-binding domain of CRBN (CRBN_CTD_), raising a question whether BRD4–ZZ1 binding occurs through a similar structural mechanism.

### ZZ1 glues BRD4_BD1_ to the CRBN-like domain of YPEL5

**Fig. 2:**
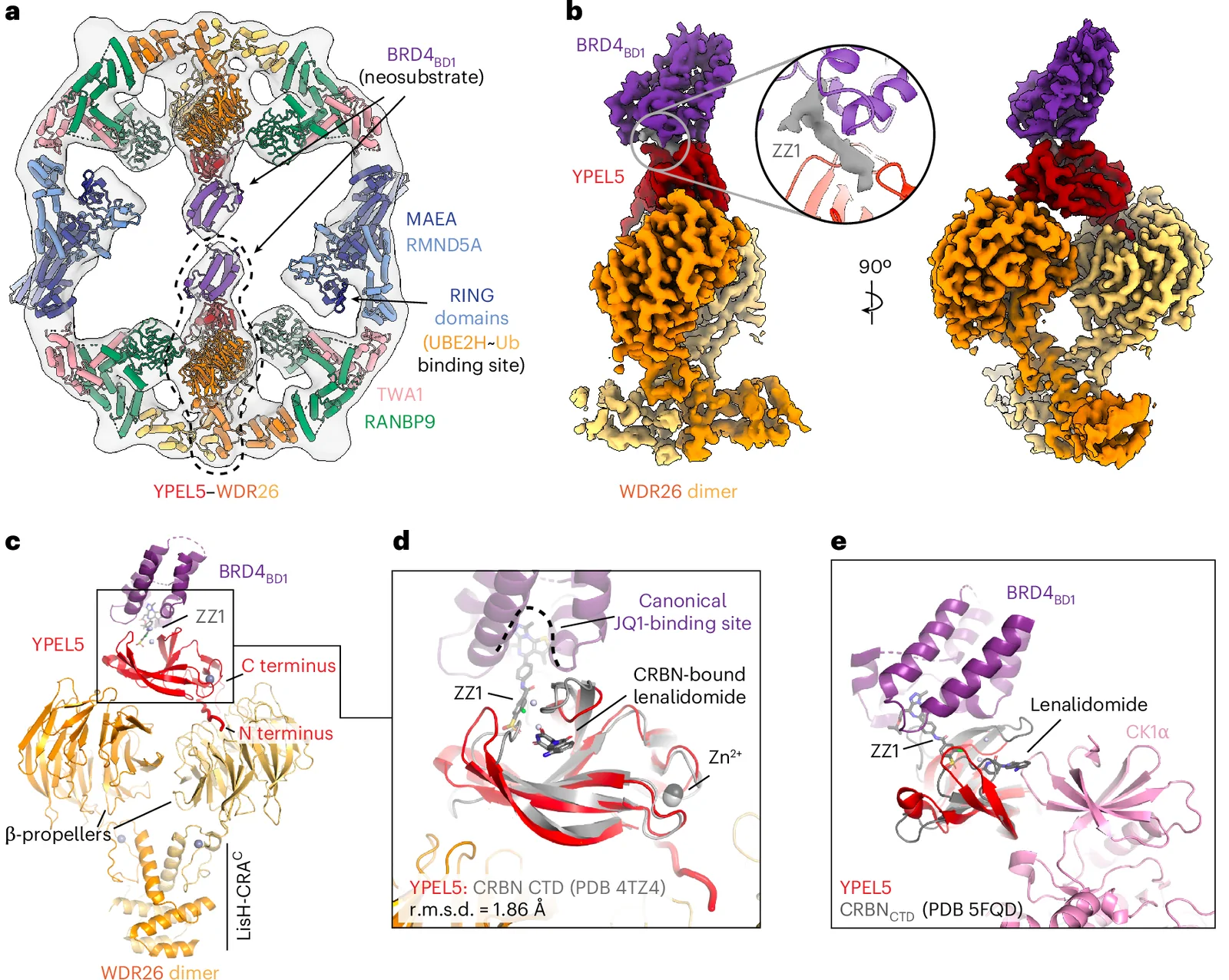
YPEL5 is a ZZ1-induced substrate receptor for BRD4 targeting. **a**, Cryo-EM map of the neosubstrate recognition complex (YPEL5-CTLH E3–ZZ1–BRD4_BD1_) resolved to 12 Å and fitted with prior structures (extracted from PDB 7NSC (ref. ^40^), PDB 8PJN (ref. ^39^), PDB 8QBN (ref. ^44^) and PDB 3MXF (ref. ^34^)) and AlphaFold models^66^ of the constituent CTLH modules. **b**, Map of the YPEL5–WDR26 module–ZZ1–BRD4_BD1_ ternary complex resolved to 3.4 Å and sharpened with DeepEMhancer^67^. The close-up view highlights additional electron density at the interface of YPEL5 and BRD4_BD1_ corresponding to the ZZ1 degrader. **c**, Atomic model of the ZZ1-induced ternary complex depicting the overall YPEL5–WDR26 receptor module architecture and its mode of BRD4_BD1_ engagement. **d**, Close-up view of YPEL5 in complex with ZZ1–BRD4_BD1_ overlaid with lenalidomide-bound CRBN_CTD_ (PDB 4TZ4)^68^, illustrating the structural similarity of their ligand-binding domains. Both domains are stabilized by coordination of a zinc atom and contain a central groove that binds ligands. **e**, YPEL5 and CRBN (PDB 5FQD)^47^ MGD ternary complexes have divergent modes of degrader engagement and neosubstrate positioning.

To understand the mechanism of ZZ1-induced ternary complex formation, we determined a cryo-electron microscopy (cryo-EM) structure of the fully assembled BRD4_BD1_-bound YPEL5-CTLH complex. As reported previously, the WDR26-containing CTLH E3 is a giant two-fold symmetric oval with an external diameter of 300 Å in its longest aspect and a hollow center that accommodates substrates^40,41,44^. The overall map, resolved to ~12 Å, shows two centrally located BRD4_BD1_ molecules, each interacting with two-fold symmetry-related YPEL5–WDR26 modules (Fig. 2a and Supplementary Table 2). This binding mode positions BRD4_BD1_ adjacent to the heterodimeric RING domains of the catalytic CTLH subunits, thus explaining the UBE2H-dependent ubiquitylation. Through focused refinement and signal subtraction with a mask over the aligned YPEL5–WDR26 modules bound to BRD4_BD1_–ZZ1, we determined a subcomplex structure resolved to 3.4 Å. This enabled unambiguous modeling of the MGD-driven ternary complex (Supplementary Table 2 and Supplementary Fig. 1). The modeled interface showed three remarkable features.

First, ZZ1 is a MGD that bridges YPEL5 and BRD4_BD1_ (Fig. 2b,c). The JQ1 moiety of ZZ1 interacts with its canonical binding site on BRD4_BD1_ and the appended degron protrudes out of the BRD4_BD1_ pocket, creating a neomorphic surface for YPEL5 engagement (Fig. 2d). The exposed chemical moiety inserts into the central β-sheet groove of YPEL5, inducing YPEL5–BRD4_BD1_ protein interactions.

**Extended Data Fig. 4:**
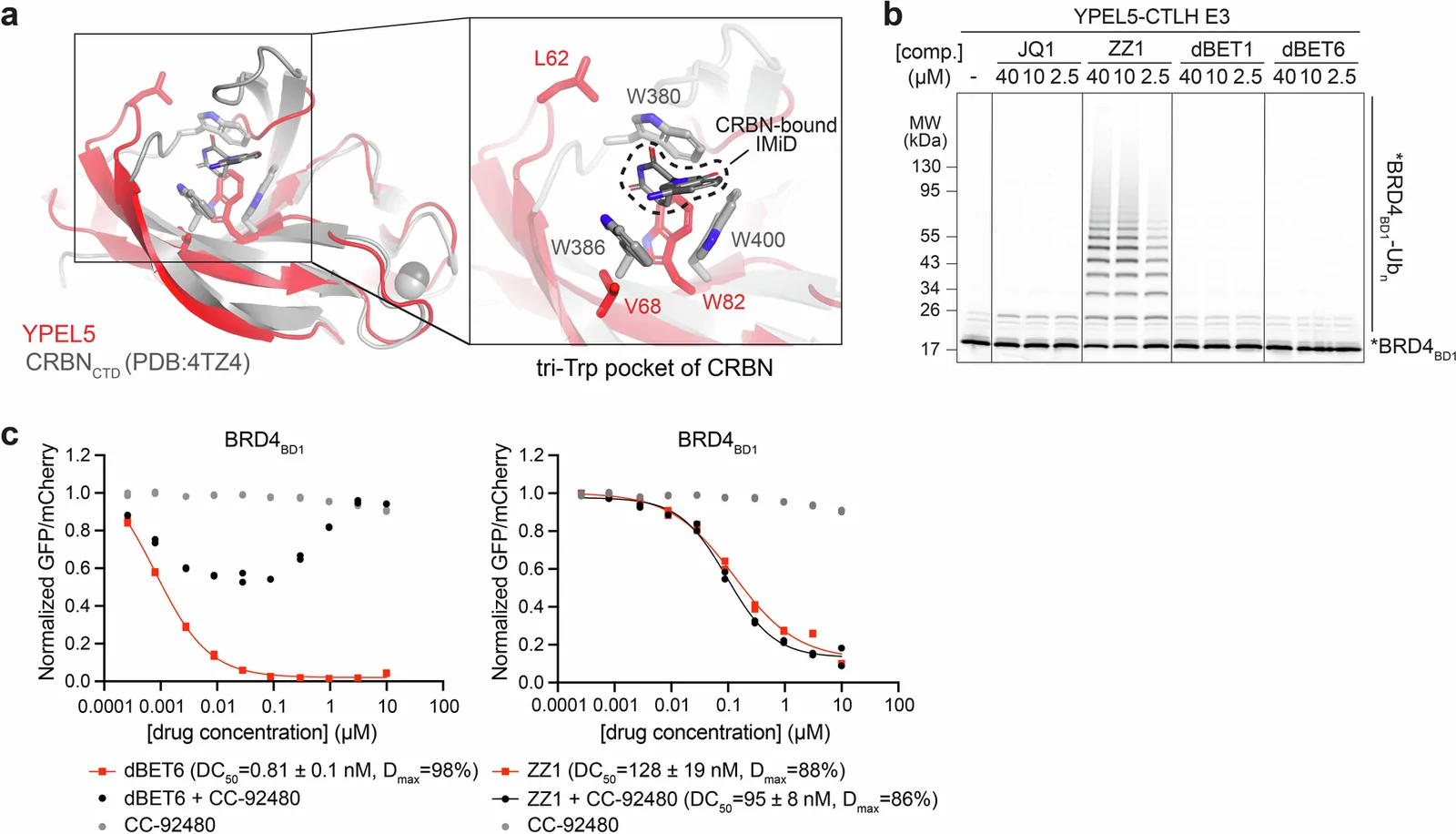
YPEL5 contains the CRBN-like domain with distinct degrader specificity. **a** Structural superposition of YPEL5 and the thalidomide binding domain of CRBN (CRBN_CTD_) engaging an Immunomodulatory Drug (IMiD) MGD (PDB: 4TZ4). The close-up highlights CRBN residues forming the IMiD-engaging hydrophobic site (the ‘tri-Trp pocket’) and the corresponding YPEL5 residues (shown as sticks). Despite adopting a homologous fold, YPEL5 does not possess two out of three residues critical for IMiD binding. **b** Examining ligand-binding preference of YPEL5 by performing *in vitro* BRD4_BD1_ ubiquitylation assay with ZZ1 and CRBN-based PROTACs employing the BRD4 ligand JQ1. **c** Analysis of BRD4_BD1_-eGFP degradation in K562 stability reporter cells treated with indicated compounds or compound combinations (co-treatment with equimolar mixtures) (SD, n = 2). Source Data

Second, ZZ1 binds the ligand-binding pocket in YPEL5 that corresponds to CRBN_CTD_^44^ (Fig. 2d). YPEL5 and CRBN_CTD_ have high structural similarity (root-mean square deviation (r.m.s.d.) = 1.86 Å) despite low sequence identity (~17%). Consistent with the divergent primary sequences, neither the molecular details of degrader engagement (ZZ1 for YPEL5 and thalidomide for CRBN) nor the orientations of the recruited neosubstrates relative to the shared binding groove (Fig. 2e and Extended Data Fig. 4a) are conserved. Consequently, CRBN-based proteolysis-targeting chimeras (PROTACs) using the BRD4 ligand JQ1 did not trigger YPEL5-dependent BRD4_BD1_ ubiquitylation (Extended Data Fig. 4b). This is consistent with a lack of cellular competition between the thalidomide analog CC-92480 and ZZ1 for BRD4_BD1_ reporter degradation (Extended Data Fig. 4c). Thus, YPEL5 and CRBN_CTD_ have distinct ligand-binding preferences despite sharing a fold.

### MGD function of ZZ1 requires its metabolism to sulfinic acid

**Fig. 3:**
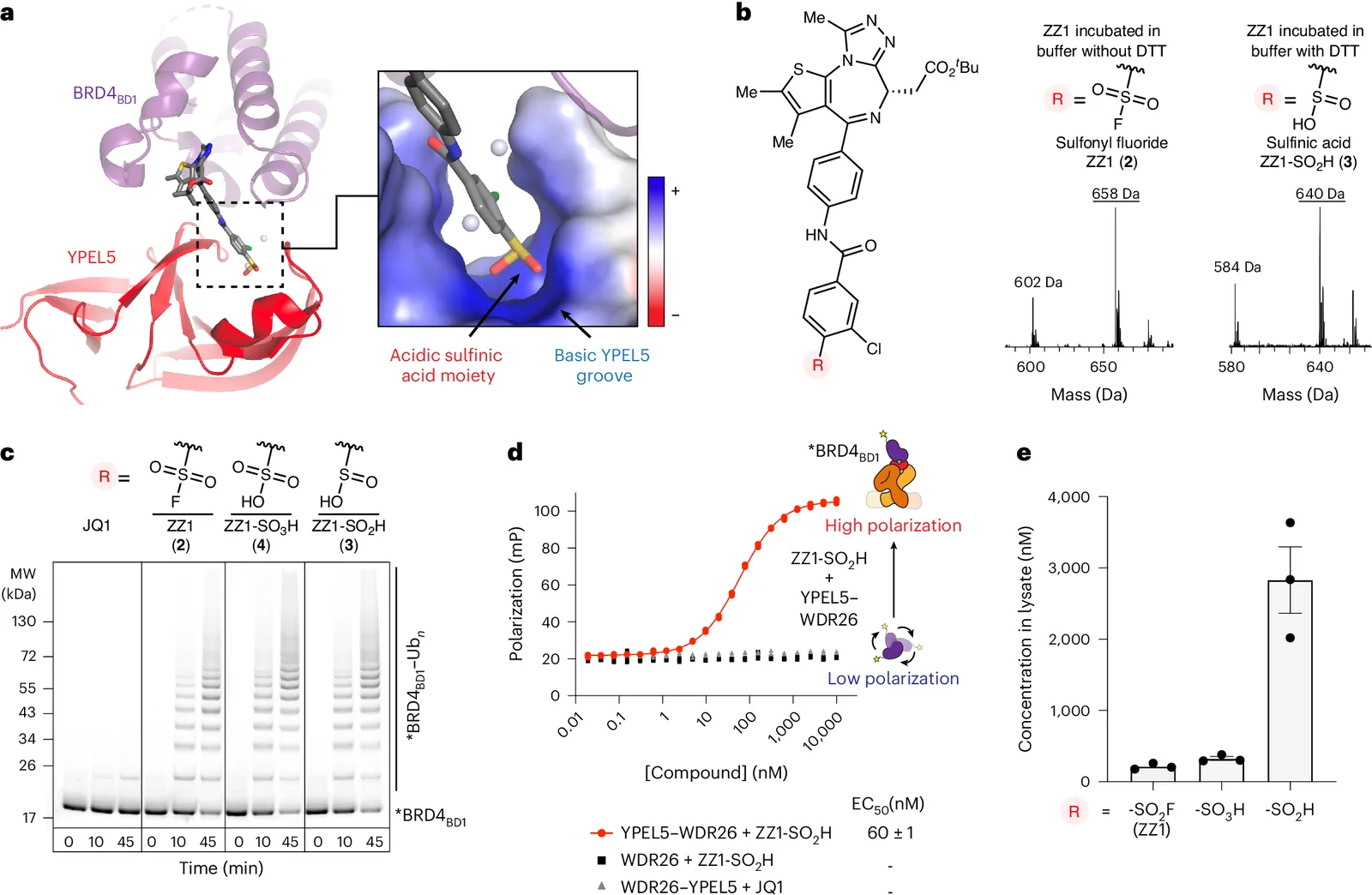
ZZ1 is a metabolically activated prodrug of a charged MGD (c-Glue). **a**, Structure of the ternary complex illustrating electrostatically driven interactions between the negatively charged sulfinic acid moiety of ZZ1-SO_2_H and the basic bottom of the YPEL5-binding groove (represented as an electrostatic potential surface). **b**, Intact MS demonstrates conversion of the sulfonyl fluoride moiety of ZZ1 (middle) to sulfinic acid (right) after incubation in a DTT-containing buffer. Left, the position of the transformed group is indicated in the degrader’s chemical structure. Peaks corresponding to lower-molecular-weight species correspond to ZZ1 and ZZ1-SO_2_H derivatives formed upon hydrolysis of their *tert*-butyl ester. **c**, Stimulation of YPEL5-CTLH E3-dependent in vitro ubiquitylation of *BRD4_BD1_. ZZ1, its sulfinic acid derivative (ZZ1-SO_2_H) and its sulfonic acid derivative (ZZ1-SO_3_H) were tested. **d**, FP assay quantifying the propensity of ZZ1-SO_2_H to induce the ternary complex formation. Binding of *BRD4_BD1_ to the YPEL5–WDR26 module upon degrader titration results in a dose-dependent increase in FP. Fitting polarization values to the [agonist] versus response model yielded the EC_50_. Data shown are the mean ± s.d. (*n* = 3). **e**, High-performance LC analysis of intracellular levels of ZZ1 and its acidic metabolites. Jurkat cells were treated with 5 µM ZZ1 for 5 h. Error bars represent the s.d. (*n* = 3 biological replicates) and heights of the bars indicate the mean. Source Data

**Extended Data Fig. 5:**
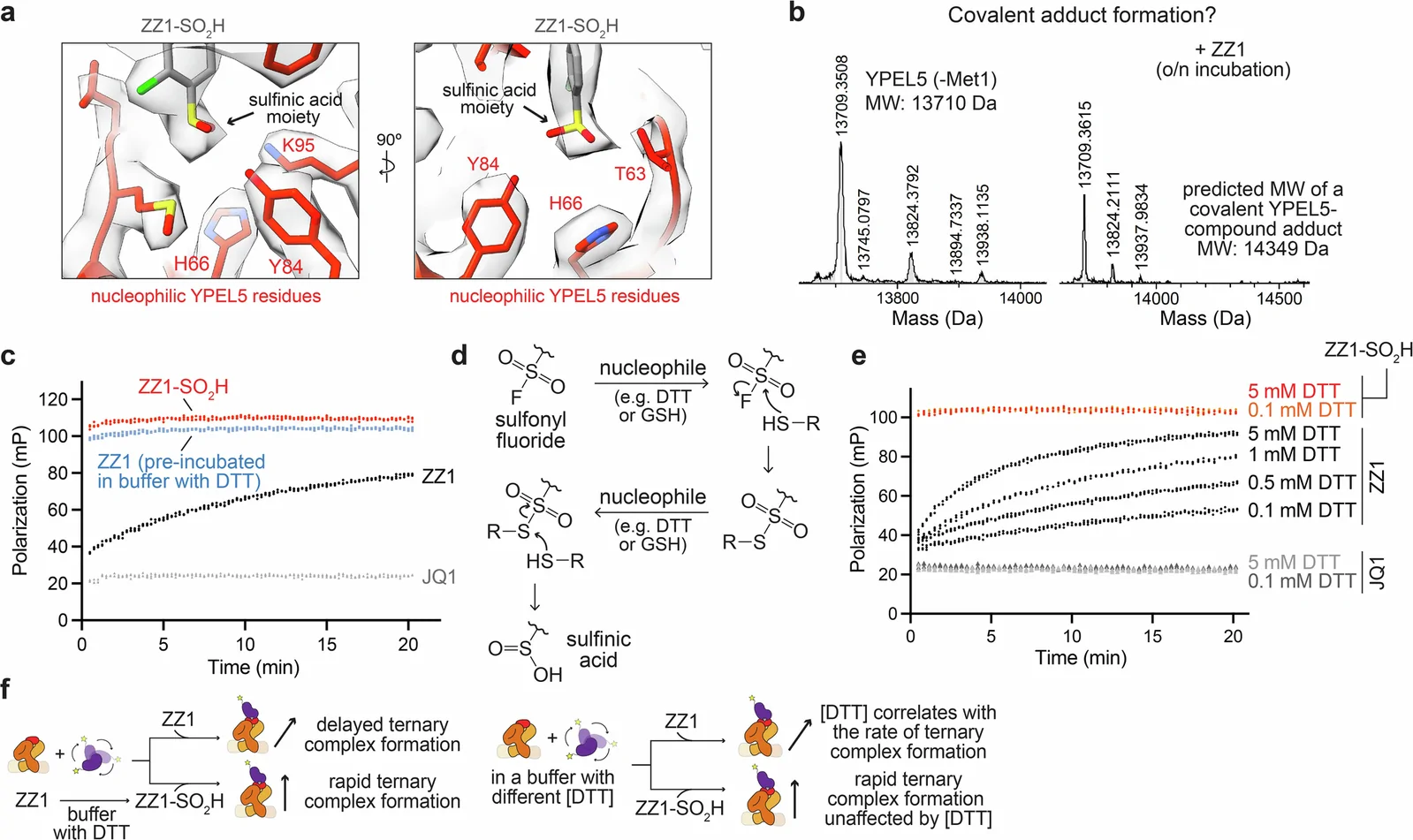
Biochemical assays deciphering the ZZ1 mode-of-action. **a**) Close-up of electron density (gray transparent) for the cryo-EM structure of the ternary complex (shown in Fig. 2b) corresponding to the ZZ1-SO_2_H chemical tag and the surrounding YPEL5 residues, along with their atomic coordinates (shown as sticks). Absence of continuous density between the sulfinic acid and neither YPEL5 nucleophilic amino acid side chains suggests the non-covalent mechanism. **b**) Intact mass spectrometry analysis testing formation of potential ZZ1-induced covalent adducts between YPEL5 (within the YPEL5-WDR26 subcomplex) and BRD4_BD1_. **c**) Real-time FP assay probing kinetics of ternary complex formation induced by ZZ1, ZZ1-SO_2_H, or ZZ1 pre-incubated in the FP buffer. Polarization signal was measured over time after combining the protein mix (*BRD4_BD1_ and YPEL5-WDR26) diluted in the DTT-containing buffer with the indicated compounds. **d**) Schematic of the proposed mechanism of sulfonyl fluoride conversion to sulfinic acid triggered by nucleophilic thiol groups of reducing agents, such as DTT (*in vitro*) or GSH (in cells). **e**) Real-time FP assay testing effect of DTT on the rate of the ZZ1-SO_2_H-induced ternary complex formation. The varying DTT concentrations during the experiment were controlled by the composition of the buffer used for preparing the protein mix (*BRD4_BD1_ and YPEL5-WDR26) prior to degrader addition. **f**) Cartoons showing real-time FP assays to probe kinetics and the effect of DTT on ternary complex formation. Source Data

The third feature shown in the cryo-EM structure is that the bound ZZ1 appears to have undergone a chemical transformation that enables YPEL5 binding. The chemical tag of ZZ1 projects into the basic environment of the YPEL5-binding groove (Fig. 3a). This was unexpected, as the sulfonyl fluoride moiety does not carry a complementary negative charge. In addition, we did not observe a chemical link between ZZ1 and any surrounding YPEL5 nucleophilic amino acid side chains, although the density for ZZ1 itself was well defined (Extended Data Fig. 5a). Accordingly, we found no evidence of a covalent ZZ1–YPEL5 adduct in MS experiments carried out with recombinant proteins (Extended Data Fig. 5b). Thus, although ZZ1 features a common electrophilic covalent chemical warhead (sulfonyl fluoride)^36^, its mechanism of action does not involve covalent bond formation.

**Extended Data Fig. 6:**
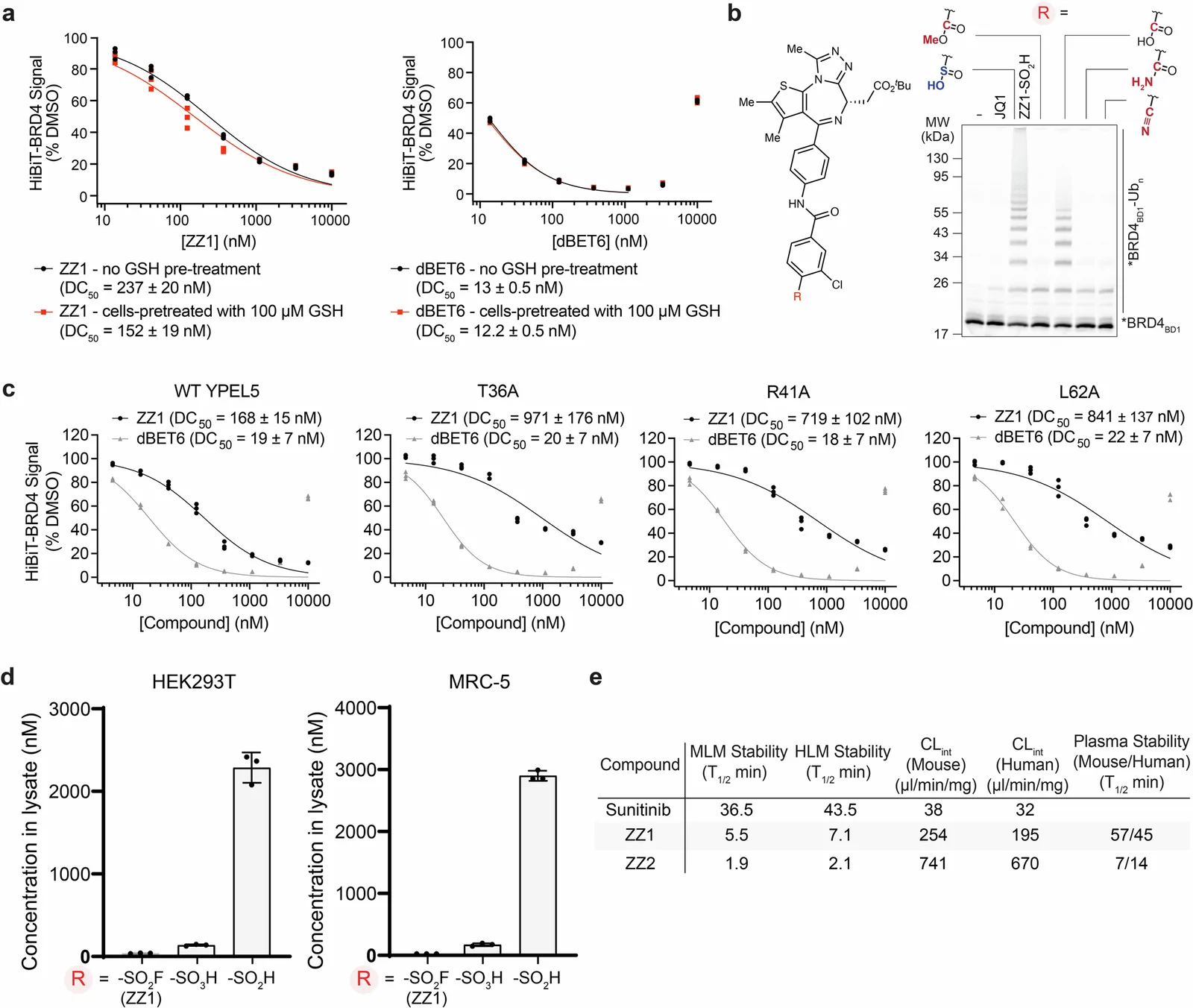
Cellular assays further confirming the ZZ1 mode-of-action. **a**) HiBiT-BRD4 assay results for Jurkat cells pre-treated with 100 µM GSH for 2 h, followed by treatment with the indicated compounds for 5 h (SD, n = 3). **b**) *In vitro* assay testing the series of ZZ1-SO_2_H-derived compounds for inducing YPEL5-CTLH E3-dependent *BRD4_BD1_ ubiquitylation. The tested compounds differed in the chemical moiety replacing the sulfinic acid group (denoted as ‘R’). All reactions were quenched after 30 min. **c**) HiBiT-BRD4 assays for WT and mutant YPEL5-expressing Jurkat cells treated with the indicated compounds for 5 h (SD, n = 3). The bar graph comparing the estimated DC_50_ values (relative to WT YPEL5) is presented in Fig. 4d. **d**) HPLC analysis of intracellular levels of ZZ1 and its acidic metabolites. HEK293T and MRC5 cells were treated with 5 µM ZZ1 for 5 h. Error bars represent SD (n = 3 biological replicates), heights of the bars indicate the mean. **e**) Microsome and plasma stability of ZZ1 and ZZ2. Source Data

We hypothesized that ZZ1 is a prodrug that requires chemical transformation into an acidic moiety for its activity. We tested this idea in several ways. First, MS analysis of ZZ1 incubated in the buffer used for biochemical assays indicated the conversion to a sulfinic acid derivative ZZ1-SO_2_H (**3**) (Fig. 3b). Second, both ZZ1-SO_2_H and the related sulfonic acid version ZZ1-SO_3_H (**4**) promoted robust BRD4_BD1_ ubiquitylation in vitro (Fig. 3c) but were inactive in cellular assays. Third, we established a fluorescence polarization (FP) assay to quantify the propensity of ZZ1 to drive ternary complex formation. The assay measures binding of fluorescently labeled BRD4_BD1_ to YPEL5–WDR26. ZZ1-SO_2_H induces a tight ternary complex between the YPEL5–WDR26 module and BRD4_BD1_ with a half-maximal effective concentration (EC_50_) of ~60 nM (Fig. 3d). Fourth, ZZ1 induced ternary complex formation slowly in biochemical assays when compared to ZZ1-SO_2_H (Extended Data Fig. 5c,f). Preincubating ZZ1 in the reaction buffer alleviated the delay. Fifth, as sulfonyl fluoride is a potent electrophile, we speculated that it might readily react with the nucleophilic thiol groups of the reducing agent dithiothreitol (DTT) present in the assay buffer to yield ZZ1-SO_2_H (Extended Data Fig. 5d). Indeed, ZZ1 transformation in vitro occurred only in the presence of the reducing agent DTT, with the concentration of DTT correlating with the rate of ZZ1-dependent ternary complex formation (Fig. 3b and Extended Data Fig. 5e,f). A similar reaction could take place in the cellular environment, potentially mediated by other thiol-containing molecules such as glutathione (GSH), whose involvement is supported by subtle enhancement of ZZ1 potency in GSH-pretreated cells^45,46^ (Extended Data Fig. 6a). Sixth, metabolomic analyses of lysates from ZZ1-treated cells identified ZZ1-SO_2_H as the predominating intracellular species versus the nonconverted parental compound ZZ1 (Fig. 3e and Extended Data Fig. 6d), consistent with the generally poor drug-like properties of reactive sulfonyl fluoride moieties (Extended Data Fig. 6e). Therefore, sulfinic acid perception is the defining feature of ZZ1-SO_2_H recognition, which is emphasized by designation of ZZ1 as a c-Glue.

### Basis for charge recognition and substrate selectivity

**Fig. 4:**
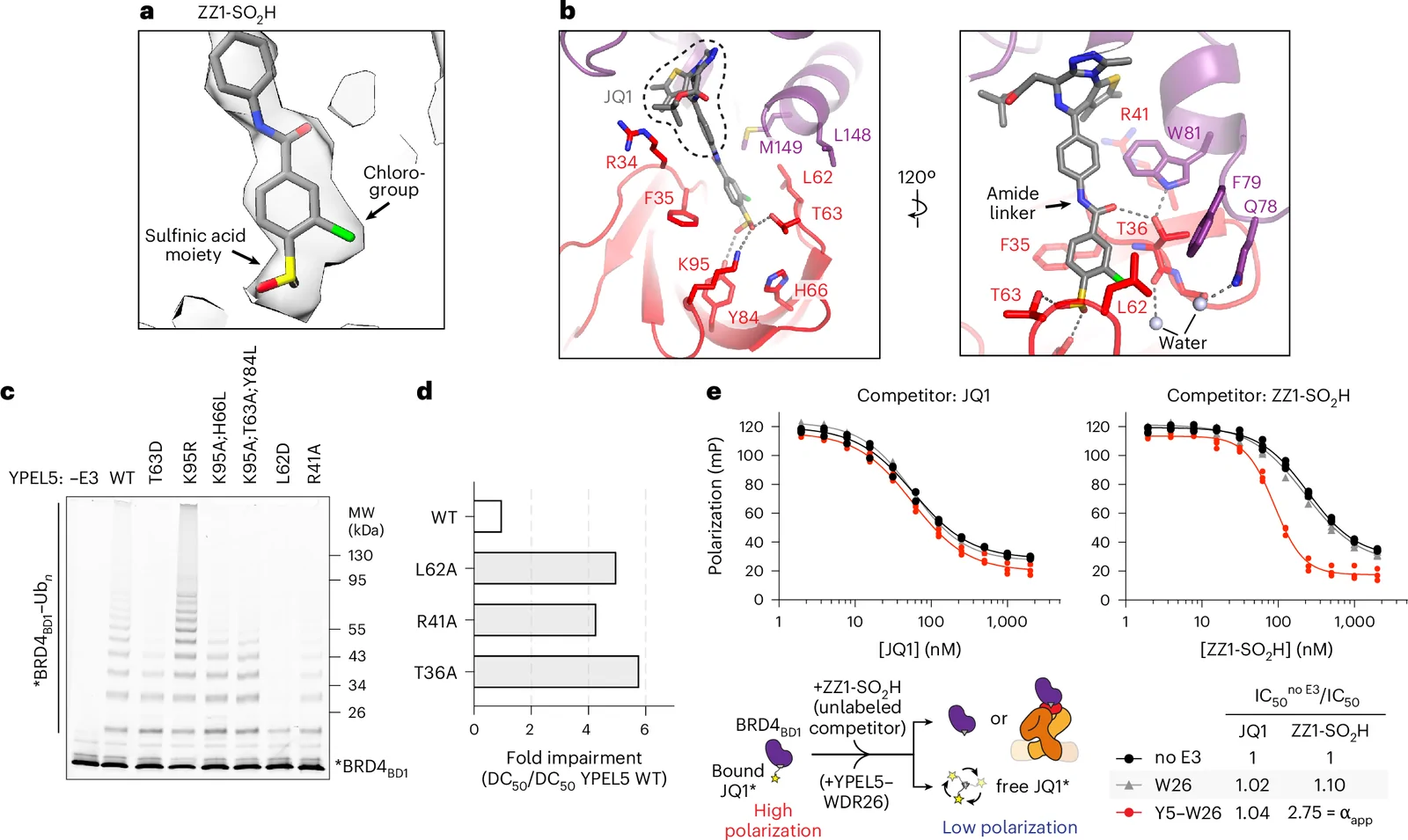
ZZ1-SO_2_H induces cooperative ternary complex formation. **a**, Close-up view of cryo-EM density corresponding to the YPEL5-engaging chemical tag of ZZ1-SO_2_H (compound coordinates shown as sticks). **b**, Molecular details of the ternary complex interface highlighting the constellation of YPEL5 residues engaging ZZ1-SO_2_H and those involved in direct interactions with BRD4_BD1_. The hydrogen bonds are depicted as gray dashes. **c**, In vitro ubiquitylation assay probing YPEL5 residues shown in **b** involved in (1) anchoring the sulfinic acid moiety (T63, H66, K95 and Y84); (2) contacts with the degrader phenyl ring and BRD4 hydrophobic side chains (L62); and (3) direct interactions with BRD4 (R41). Reactions were quenched after 30 min. **d**, Cellular BRD4 degradation assay testing the structurally visualized binding mode. The impact of YPEL5 mutants on ZZ1 potency is illustrated as the ratio of DC_50_ values between mutant and WT YPEL5-expressing Jurkat cells. Plots used for DC_50_ measurements are presented in Extended Data Fig. 7a (*n* = 3). **e**, Competitive FP assay probing cooperativity within the ZZ1-SO_2_H-induced ternary complex. The BRD4_BD1_-bound fluorescent JQ1* tracer was displaced by titration of unlabeled competitors reducing FP. The extent of cooperativity was determined by calculating the ratio of IC_50_ values (estimated by fitting polarization values to the ‘[inhibitor] versus response’ model) in the absence and presence of excess YPEL5–WDR26, corresponding to an apparent cooperativity factor α_app_ (*n* = 3). Source Data

Given the evidence outlined above, we used the converted ZZ1-SO_2_H to model the cryo-EM density and inspected the resulting model to determine the basis of molecular recognition (Fig. 4a,b). Firstly, the combination of electrostatic contacts and hydrogen bonding between the acidic ZZ1-SO_2_H moiety and the bottom of the basic YPEL5 pocket anchors the complex. Accordingly, substituting the acidic and polar residues surrounding the degrader’s sulfinic group with nonpolar amino acids, as well as substituting adjacent T63 with a negatively charged aspartic acid, attenuated this activity (Fig. 4c). By contrast, increasing the basic character of the pocket by substituting K95 to arginine potentiated ZZ1-induced BRD4_BD1_ ubiquitylation (Fig. 4c). Furthermore, replacing ZZ1’s sulfinic acid with a negatively charged carboxylic acid group but not any of the related uncharged moieties tested partially supported BRD4_BD1_ ubiquitylation in biochemical assays (Extended Data Fig. 6b and Supplementary Table 3).

Secondly, hydrophobic residues within the loops lining the entrance to the YPEL5 groove sandwich the phenyl group of the degron tag. Disrupting these hydrophobic contacts by substituting L62, which interacts with both ZZ1 and the surrounding apolar BRD4 residues, nearly completely abolished ZZ1 activity in vitro and considerably impaired BRD4 degradation (Fig. 4c,d and Extended Data Fig. 6c). Additionally, the amide linker between the BRD4 ligand and the appended chemical tag forms a hydrogen bond with YPEL5 T36, thereby buttressing YPEL5 engagement. Substitution at this position weakened ZZ1 potency in cells (Fig. 4d and Extended Data Fig. 6c).

**Extended Data Fig. 7:**
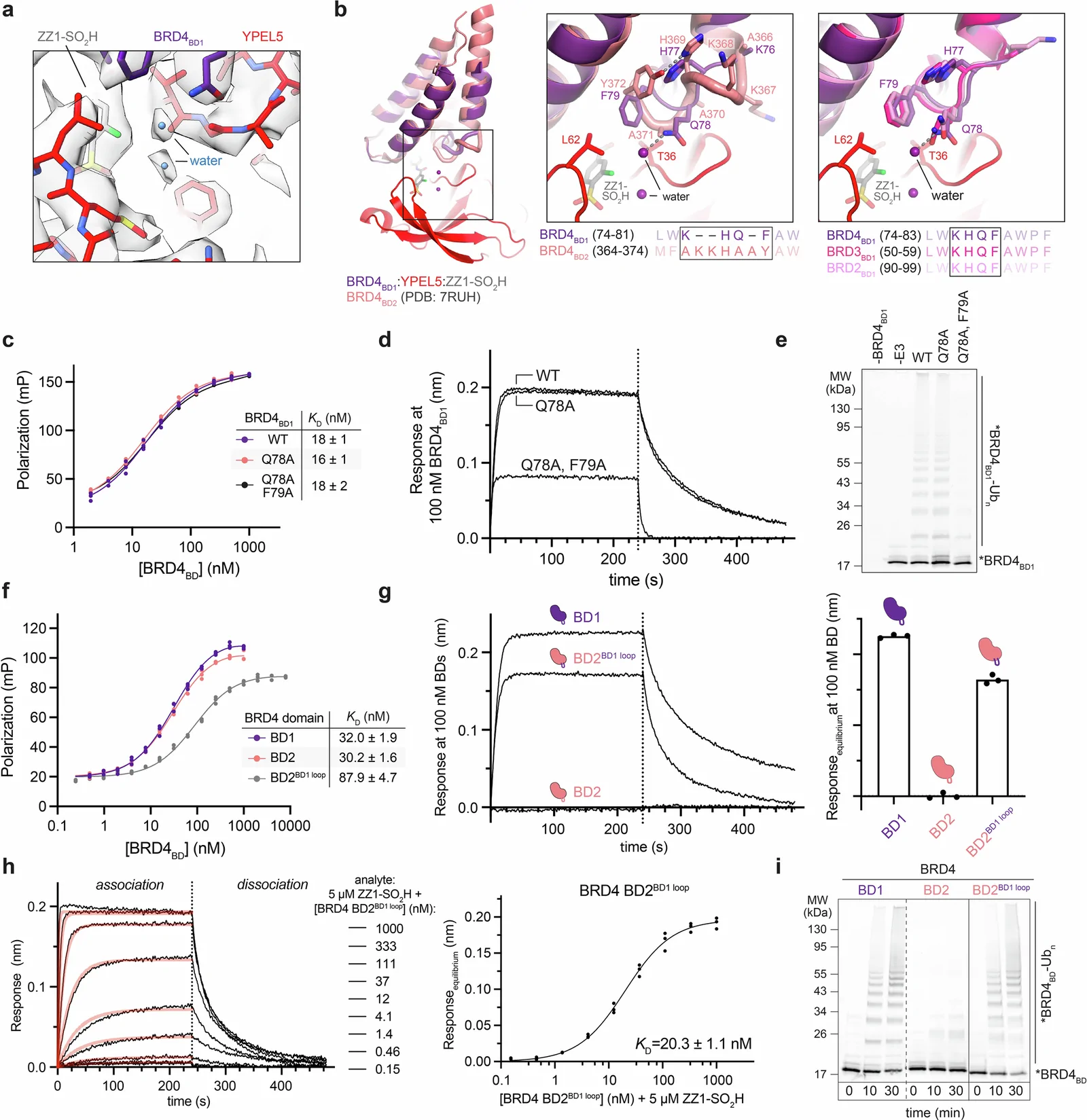
Elucidating structural determinants determining ZZ1’s bromodomain selectivity. **a** Close-up of focused-refined cryo-EM map showing density interpreted as water molecules within the ternary complex. **b** Structural basis for the bromodomain selectivity of ZZ1. Left, superposition of BRD4_BD2_ (PDB: 7RUH) and the YPEL5-ZZ1-BRD4_BD1_ complex (thick ribbon). Middle, the BRD4_BD2_ portion is longer than and has a distinct sequence from the corresponding BRD4_BD1_ loop. This loop is conserved in the BD1 domains of BRD2, BRD3, and BRD4 (right). **c** Testing binding of structure-based BRD4_BD1_ mutants to fluorescent FAM-JQ1 probe with a fluorescence polarization assay (SD, n = 3). **d** Biolayer interferometry (BLI) assay examining importance of the YPEL5-contacting residues within the BD1-specific loop on ternary complex formation. WT and mutant BRD4_BD1_ constructs were tested for binding to immobilized YPEL5-WDR26 (at a single 100 nM concentration of a bromodomain and in the presence of 5 μM ZZ1-SO_2_H). **e** Assay testing ZZ1-SO_2_H-induced ubiquitylation of fluorescent WT and structure-based BRD4_BD1_ mutants, after 20 min reaction. **f** Fluorescence polarization assay measuring binding affinities (equilibrium dissociation constant, *K*_D_) of BRD4 bromodomain constructs for the fluorescent FAM-JQ1 probe (SD, n = 3). **g** BLI assay determining the specificity-determining region of BRD4 (left). WT and loop swap mutant of BRD4 bromodomains were tested for binding to immobilized YPEL5-WDR26 (as in d). The equilibrium response values of the association curve (left) are presented as a bar graph (right; n = 3 biological replicates, heights of the bars indicate the mean). **h** Quantification of the ZZ1-SO_2_H-induced binding affinity (equilibrium dissociation constant, *K*_D_) of the ‘gain-of-function’ BRD4_BD2_ mutant (harboring the BRD4_BD1_ loop) for immobilized YPEL5-WDR26 by BLI. The representative sensorgram (left) shows association and dissociation curves for a dilution series of BRD4_BD1_ (at a constant MGD concentration). The equilibrium responses estimated based on the fits (red lines) of the association-dissociation curves (left; shown in the representative sensorgram) were plotted against the corresponding concentrations of BRD4 BD2^BD1 loop^ for calculation of *K*_D_ (right) (SD, n = 3). **i** Assay testing ZZ1-SO_2_H-induced YPEL5-CTLH E3-dependent ubiquitylation of fluorescent BRD4 BD1 and BD2 constructs. Source Data

**Extended Data Fig. 8:**
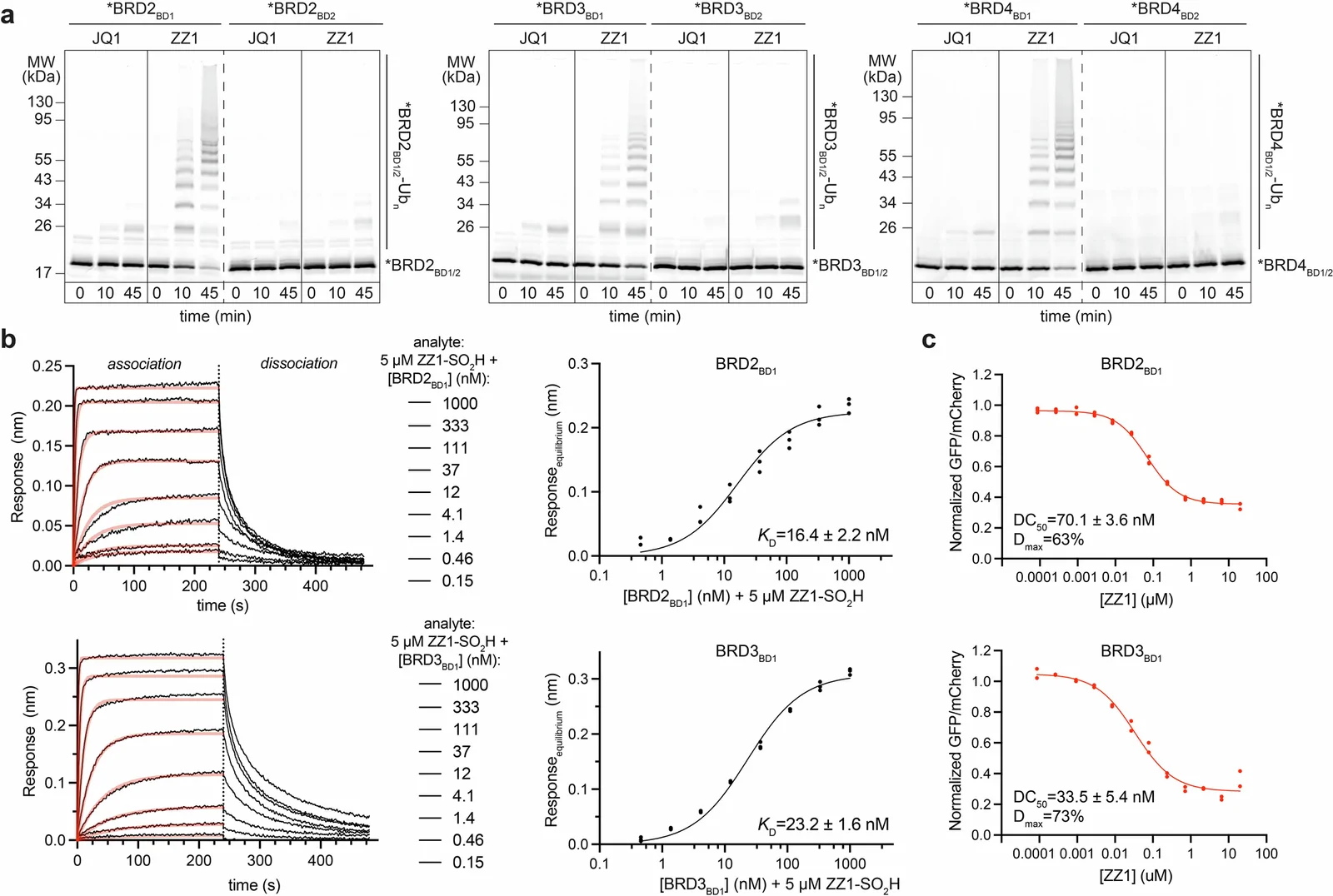
Biochemical and cellular assays confirming that ZZ1 selectively induces BD1 degradation. **a** *In vitro* ubiquitylation assays querying selective targeting of a specific bromodomain by ZZ1-SO_2_H across the BET-family proteins. **b** Quantification of the ZZ1-SO_2_H-induced affinity of the BD1 domains from BRD2 and BRD3 for immobilized YPEL5-WDR26 by BLI (as in Extended Data Fig. 5c and 7 g) (SD, n = 3). **c** Cellular fluorescent reporter assay testing ZZ1-induced degradation of BRD2 and BRD3 BD1 bromodomains (SD, n = 2). Source Data

**Extended Data Fig. 9:**
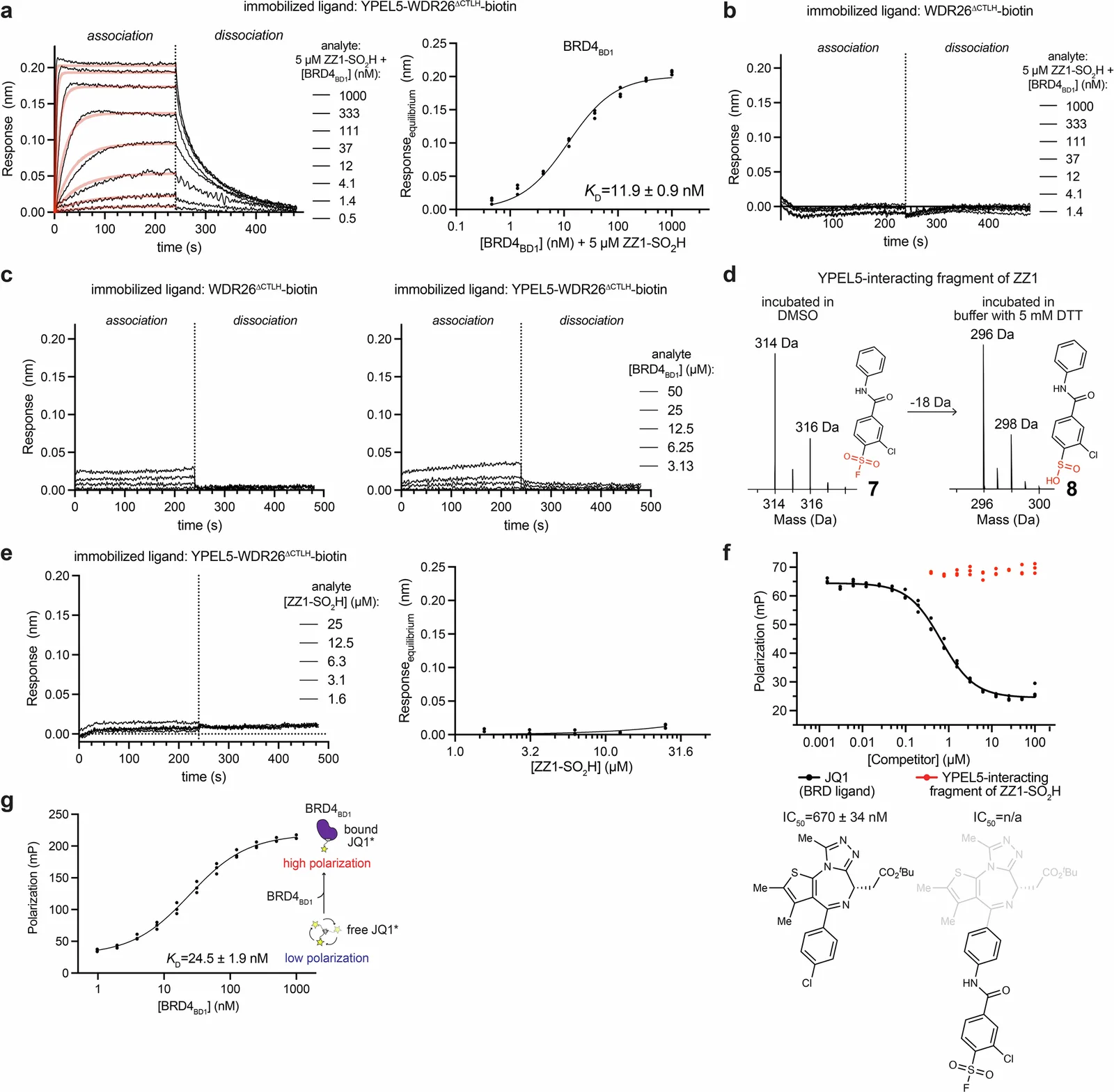
Characterization of ZZ1 specificity and binding properties. **a** BLI assay quantifying binding affinity of YPEL5-WDR26 for ZZ1-SO_2_H-bound BRD4_BD1_. The equilibrium responses estimated based on the fits (red lines) of the association-dissociation curves were plotted against the corresponding BRD4_BD1_ concentrations for calculation of *K*_D_ (right; SD, n = 3). **b** BLI assay as in (a) but performed with isolated dimeric WDR26 construct (in the absence of YPEL5), thus probing YPEL5 dependence of the ZZ1-SO_2_H-induced ternary complex. **c** BLI assay to examine the intrinsic BRD4_BD1_-YPEL5 binary affinity (in the absence of the degrader). BLI data were collected for a series of BRD4_BD1_ concentrations for the immobilized YPEL5-WDR26 complex and WDR26 alone. **d** BLI assay to examine the binary affinity of YPEL5 for ZZ1-SO_2_H (in the absence of the neosubstrate). The equilibrium responses of the association-dissociation curves were plotted against the corresponding degrader’s concentrations (right; SD, n = 3). **e** Intact mass spectrometry analysis confirming conversion of the sulfonyl fluoride version of the YPEL5-interacting fragment of ZZ2 to its acidic sulfinic acid derivative (upon overnight incubation in a DTT-containing buffer). **f** Competitive FP assay testing JQ1 (the high-affinity parental BRD4 ligand) and activated YPEL5-interacting ZZ1-SO_2_H fragment (d) for disrupting the ternary complex comprised of *BRD4_BD1_, ZZ1-SO_2_H and YPEL5-WDR26 (see Fig. 3d). Half maximal inhibitory concentration (IC_50_) was estimated by fitting polarization values to ‘[inhibitor] vs. response’ model (SD, n = 3). **g** FP assay for establishing the competitive ligand displacement experiment probing cooperative ternary complex formation (Fig. 4e). Polarization values upon BRD4_BD1_ titration to the fluorescent JQ1 tracer (FAM-JQ1) were fit to the one-site binding model to estimate the BRD4_BD1_-JQ1 affinity (equilibrium dissociation constant, K_D_) (SD, n = 3). Source Data

Akin to other structurally characterized MGDs^9,47^, ZZ1-SO_2_H promotes direct target protein–E3 ligase interactions^48^, burying a total surface area of ~350 Å^2^. Here, key contacts are made by a short region of BRD4_BD1_ abutting the JQ1-binding site (Fig. 4b and Extended Data Fig. 7a,b). These include stacking of W81 against YPEL5 R41, whose substitution impairs ZZ1 activity both in vitro and in cellulo (Fig. 4b–d). Moreover, Q78 and F79 within the preceding loop contribute to the three-way interface with ZZ1’s chloro group and YPEL5 (Fig. 4b and Extended Data Fig. 7a,b). Notably, while simultaneous substitution of these residues negatively impacts ternary complex formation and ubiquitylation of BRD4_BD1_ (Extended Data Fig. 7c–e), installing the entire BD1-specific loop onto the corresponding region of BRD4_BD2_ promoted its ‘gain-of-function’ targeting in vitro (Extended Data Fig. 7f–h). Accordingly, consistent with the conservation of this loop in the BD1 domains of BRD2 and BRD3 (Extended Data Fig. 7b), they were both efficiently targeted in cellular degradation and in vitro ubiquitylation assays and formed tight ZZ1-mediated ternary complexes with YPEL5 (Extended Data Fig. 7i, 8a–c and 9a,b). Therefore, involvement of specific protein–protein contacts dictates neosubstrate selectivity of ZZ1 for a specific BET BD.

Lastly, we sought to determine the relative contributions of the binary interactions supporting formation of the ternary complex. Despite the seeming structural complementarity, BRD4_BD1_ alone does not appreciably interact with YPEL5 (Extended Data Fig. 9c). Only upon formation of the ZZ1-SO_2_H–BRD4_BD1_ composite surface does YPEL5 bind, with a *K*_D_ of ~12 nM (Extended Data Fig. 9a,b). Meanwhile, we did not observe measurable affinity between the degrader and YPEL5 by biolayer interferometry (BLI) in the absence of BRD4_BD1_ (Extended Data Fig. 9d). Accordingly, only the BRD4_BD1_ ligand JQ1 but not the YPEL5-interacting fragment of ZZ1-SO_2_H could disrupt the ternary complex (Extended Data Fig. 9e,f). As neither the compound nor BRD4_BD1_ can interact with YPEL5 individually, the high affinity of the ternary complex appears to be driven by synergistic contacts between YPEL5 and the composite ZZ1–BRD4_BD1_ surface. To probe the importance of cooperativity, we used a competitive FP assay (Fig. 4e and Extended Data Fig. 9g). Whereas JQ1 displaced the fluorescent JQ1 probe from BRD4_BD1_ with similar half-maximal inhibitory concentration (IC_50_) regardless of the E3 ligase presence, ZZ1-SO_2_H exhibited a 2.75-fold stronger suppressive effect (corresponding to an apparent cooperativity factor α_app_) in the presence of the YPEL5–WDR26 module but not WDR26 alone. The enhanced affinity of a degrader to a target protein in the presence of an E3 ligase indicates the formation of a positively cooperative ternary complex, corroborating the structurally observed binding mode.

### Structure-based optimization yields superior degrader ZZ2

**Fig. 5:**
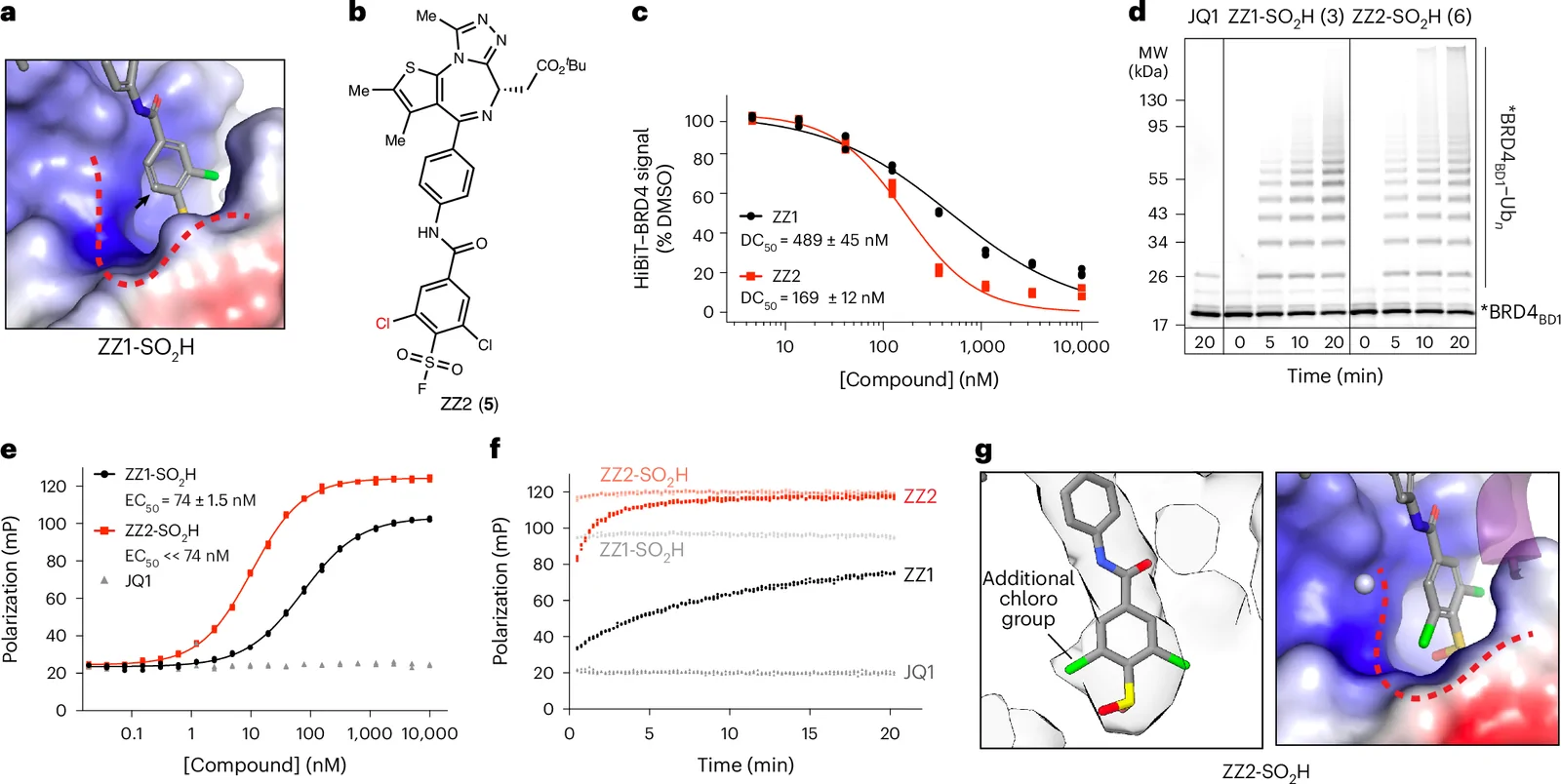
Structure-based improvement of the c-Glue. **a**, Electrostatic potential surface of the ZZ1-SO_2_H-bound YPEL5 groove showcasing a vacant basic pocket (outlined with a red dash) adjacent to the unsubstituted position of the degrader’s chemical tag (indicated by an arrow). **b**, Chemical structure of ZZ2 highlighting the incorporated chloro group (red) at the second *ortho* position of ZZ1’s chemical tag. **c**, HiBiT–BRD4 assay results for Jurkat cells treated with the indicated compounds for 5 h (mean ± s.d.; *n* = 3). **d**, Qualitative comparison of the ability of ZZ1-SO_2_H and ZZ2-SO_2_H to trigger in vitro YPEL5-CTLH E3-catalyzed *BRD4_BD1_ ubiquitylation. ZZ2-SO_2_H was generated by preincubation of ZZ2 in the DTT-containing buffer (Extended Data Fig. 10g). **e**, FP assay quantifying propensity of ZZ1-SO_2_H and ZZ2-SO_2_H to promote ternary complex formation (mean ± s.d.; *n* = 3). Note that polarization values obtained upon ZZ2-SO_2_H titration fit the [agonist] versus response model but its superior MGD activity precludes accurate estimation of EC_50_. **f**, Real-time FP assay testing rates of ternary complex formation triggered by ZZ1 and ZZ2 as well as their acidic derivatives. FP was monitored over time upon mixing *BRD4_BD1_ and YPEL5–WDR26 with different versions of the degraders (*n* = 3). **g**, Close-up view of the ZZ2-SO_2_H-induced ternary complex cryo-EM structure, resolved to 3.4 Å and sharpened with DeepEMhancer. The images highlight the overall fit of the degrader’s chemical tag (left) and the filling of the vacant YPEL5 basic pocket by the introduced chloro group according to the structure-based design (right). Source Data

A preliminary structure–activity relationship study of ZZ1 indicated that the *ortho*-chloro substituent is essential for potency, while substitution with other functional groups failed to yield more effective YPEL5-dependent BRD4 degraders (Supplementary Table 4). We hypothesized that introducing a small electronegative group into a vacant basic pocket adjacent to the unsubstituted *ortho*-position of the ZZ1 chemical tag could enhance electrostatic interaction with the YPEL5 basic groove (Fig. 5a). To test this idea, we synthesized ZZ2 (**5**), which has an additional chloro substituent adjacent to the existing sulfonyl fluoride moiety of ZZ1 (Fig. 5b). In doing so, we engaged in rational structure-based improvement of a fortuitously discovered c-Glue.

**Extended Data Fig. 10:**
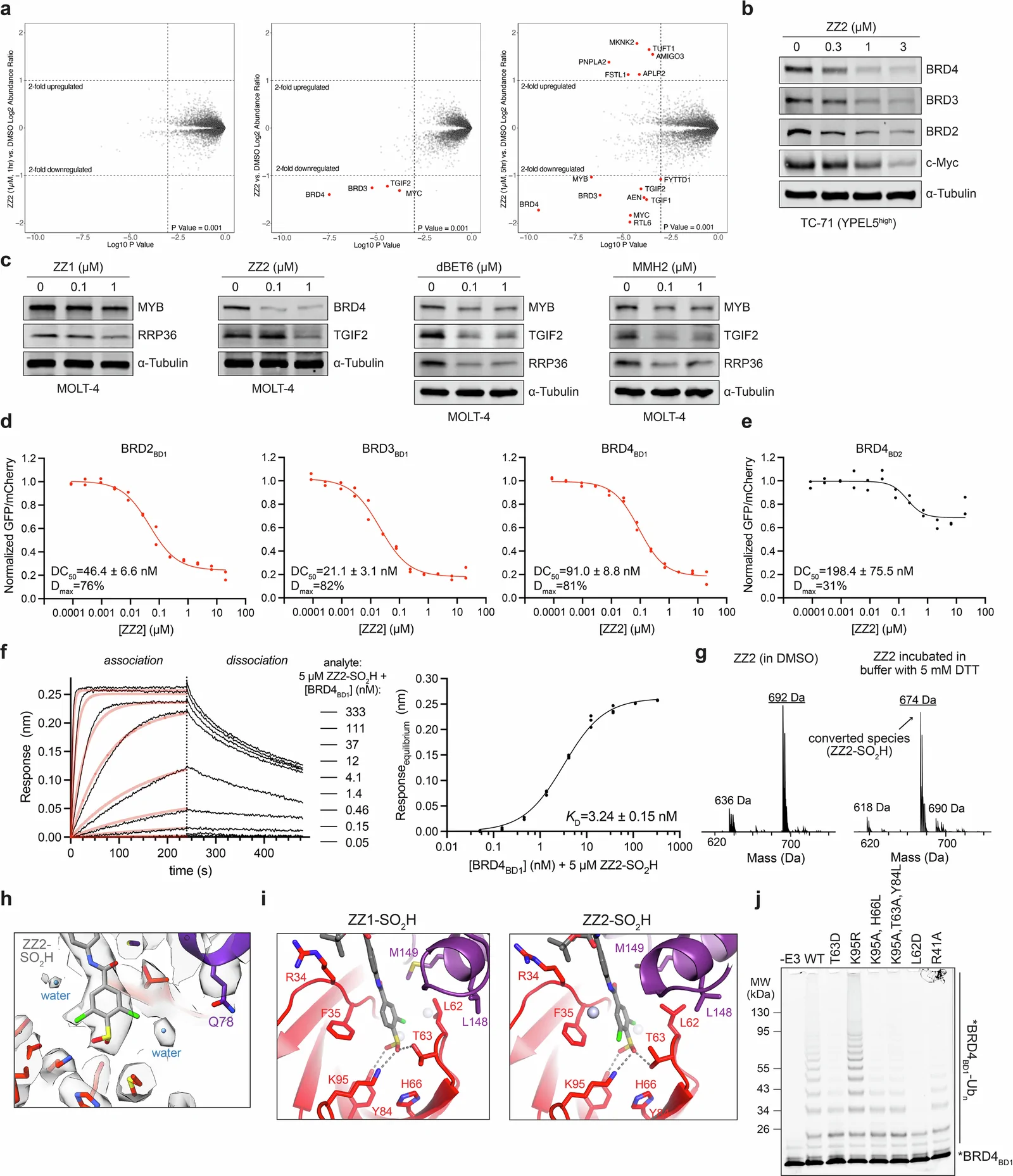
ZZ2 is an improved YPEL5-dependent BD1-specific degrader. **a**) Quantitative proteome-wide mass spectrometry in MOLT-4 cells after 1 h, 3 h, or 5 h treatment with 1 µM ZZ2. Significant changes were assessed by a two-sided moderated t-test as implemented in the Lima package. **b**) Western blots showing BRD2, BRD3, and BRD4 degradation in TC-71 cells after 5 h treatment with ZZ2. **c**) Western blots showing MYB, TGIF2, and RRP36 downregulation in MOLT-4 cells after 3 h treatment with ZZ1, ZZ2, dBET6, or MMH2. **d**) Cellular fluorescent reporter assay testing ZZ2 bromodomain selectivity. The tested reporters contained BD1 domains of BRD2, BRD3, and BRD4 (SD, n = 2). **e**) Cellular fluorescent reporter assay testing ZZ2-induced degradation of the BRD4_BD2_ reporter (SD, n = 2). **f**) Quantification of ZZ2-SO_2_H-induced affinity of BRD4_BD1_ for immobilized YPEL5-WDR26 by BLI (as Extended Data Fig. 7h) (SD, n = 3). **g**) Intact mass spec analysis confirming conversion of ZZ2 into its acidic sulfinic acid derivative upon incubation in a DTT-containing buffer. **h**) Close-up of the focused-refined cryo-EM structure highlighting the well-defined portions of density interpreted as water molecules coordinated within the ZZ2-SO_2_H-induced ternary complex. **i**) Close-up of the ternary complex structures induced by ZZ1-SO_2_H and ZZ2-SO_2_H highlighting their common binding mode. YPEL5 and BRD4 residues involved in protein-protein and/or protein-degrader contacts are shown as sticks. Spheres represent water molecules, while dashes denote hydrogen bonds. **j**) *In vitro* assay testing effect of the structure-based YPEL5 mutants on ZZ2-SO_2_H-induced ubiquitylation of *BRD4_BD1_ (as in Fig. 4c). All Western blot experiments were independently repeated at least twice with similar results. Source Data

ZZ2 is a superior BRD4 degrader when compared to ZZ1 (ZZ2 half-maximal degradation concentration (DC_50_) = 169 nM; ~3-fold more potent than ZZ1) and it retains degradation specificity for BD1 domains of the BET proteins (Fig. 5c and Extended Data Fig. 10a–e). In line with its improved degradation potency, ZZ2-SO_2_H (**6**) promotes more robust BRD4_BD1_ ubiquitylation and mediates a more stable ternary complex manifested by ~4-fold higher affinity of the degrader–BRD4_BD1_ composite surface toward YPEL5 (Extended Data Fig. 10f,g), as well as a substantially lower EC_50_ in the FP assay (Fig. 5d,e and Extended Data Fig. 10g). Moreover, ZZ2 induces the BRD4–compound–YPEL5 ternary complex at a much faster rate in vitro when compared to ZZ1 (Fig. 5f). We surmise that the rate enhancement is the consequence of two factors: improved YPEL5 engagement and accelerated conversion of the sulfonyl fluoride moiety to the active sulfinic acid, facilitated by the electron-withdrawing chloro group. In agreement with the structure-based design, the 3.4-Å cryo-EM structure of the ZZ2-SO_2_H-mediated ternary complex showed an extra portion of electron density corresponding to the new chloro substituent occupying the targeted YPEL5 pocket (Fig. 5g, Supplementary Table 2, Supplementary Fig. 2 and Extended Data Fig. 10h). Aside from the added chloro substituent and its contacts, ZZ2-SO_2_H largely maintains the interactions observed in the ZZ1-SO_2_H-induced complex (aligning with r.m.s.d. = 0.3 Å), which is reflected by the similar effects of structure-based YPEL5 amino acid substitutions on BRD4_BD1_ ubiquitylation induced by both degraders (Extended Data Fig. 10i,j). Therefore, ZZ2 substantiates the importance of electrostatic anchoring of the negatively charged c-Glue in the basic groove of YPEL5. Our initial attempts to use sulfonyl fluoride fragments from ZZ2 as YPEL5 recruiters to develop small-molecule kinase degraders using a similar strategy were unsuccessful (Supplementary Figs. 3 and 4). Thus, adventitious protein–protein interactions drive MGD potency and more potent YPEL5 ligands are likely required to identify new YPEL5-dependent MGDs.

## Discussion

ZZ1 is a CTLH-dependent BRD4 MGD that uses a prodrug mechanism to expose a negatively charged ligase-binding moiety upon its entry into target cells. Cryo-EM structures of the neosubstrate recognition complex show that, once exposed, a negatively charged sulfinic acid moiety interacts with a basic pocket on YPEL5, a CTLH subunit essential to most cancer cell lines^43^. Sulfinic acid perception is a central feature of the recognition complex and we, therefore, designate ZZ1 a c-Glue. The structures also demonstrate structural homology between YPEL5 and the thalidomide-binding domain of the IMiD (immunomodulatory drug) MGD target ubiquitin ligase, CRBN. Contrary to its only known biochemical ability, which is to inhibit a CTLH substrate interaction^44^, YPEL5 acts as a substrate receptor in ZZ1-treated cells. Rational design and synthesis of the improved compound, ZZ2, demonstrated that these properties can be exploited to create more potent YPEL5-dependent c-Glues through structure-guided medicinal chemistry.

ZZ1 and ZZ2 are c-Glues that represent a new strategy for charge-driven proximity pharmacology. Although these compounds are MGDs like the well-known IMiDs, three features set them apart. First, ZZ1 and ZZ2 undergo intracellular chemical activation and, upon conversion, become MGDs that induce a noncovalent and cooperative BRD4–compound–YPEL5 ternary complex. Second, the new compounds use charged interactions in addition to more traditional hydrophobic small molecule–protein contacts. Indeed, the CRBN IMiD-binding site is a hydrophobic ‘tritryptophan pocket’. Third, neither new compound appreciably binds YPEL5 in the absence of their BD-containing targets. This final characteristic is a key distinction; IMiD drugs bind an E3 ligase (CRBN) and cannot bind neosubstrates in its absence. The switch from E3 ligase to target as the primary binding partner indicates that future MGD discovery campaigns may be initiated ‘target-side’, enabling greater control over eventual MGD pharmacology. Recent discovery of covalent MGDs supports this idea and the work we report here formalizes this strategy and eliminates the need for covalency^15,49–51^. The c-Glues we describe herein are bona fide MGDs and retain their potency even at high compound concentrations, bypassing the so-called ‘hook effect’, which is a common limitation of PROTACs and covalent degraders^15^. These findings demonstrate that the CTLH E3 ubiquitin ligase complex is amenable to chemically induced proximity, similar to CRLs targeted by established clinical small-molecule MGDs.

The discovery of YPEL5 as an MGD receptor was unexpected, as its only documented biochemical function is to inhibit ubiquitylation of NMNAT1 (ref. ^44^). YPEL5 and NMNAT1 compete for an overlapping binding site on another CTLH E3 subunit, WDR26. Whether YPEL5 is an endogenous E3 ligase substrate receptor will require identification of its native substrates and the cryo-EM structures presented here strongly suggest these will feature acidic degrons. Nevertheless, our detailed study of YPEL5 demonstrates unexpected functional plasticity for ubiquitin ligases; an inhibitor of substrate ubiquitylation for one set of targets can stimulate the ubiquitylation of a second set. The versatility of the CTLH E3 system showcases this concept^40,41,44^.

CTLH complexes are modular and multiple auxiliary subunits are reported substrate receptors. These include GID4 (refs. ^40,52,53^), WDR26 (refs. ^41,44^) FAM72A (ref. ^54^) and, as now demonstrated in the context of an MGD, YPEL5. Diverse mechanisms of substrate recognition presumably facilitate the recruitment and positioning of a wide range of native substrates and, consequently, neosubstrates when presented with dedicated MGD compounds. Furthermore, the two-fold pseudosymmetric architecture of CTLH (Fig. 2a) exposes asymmetric neosubstrates to two distinct ubiquitylation active sites poised for ubiquitin transfer to distinct sets of neosubstrate lysine side chains. Future YPEL5-dependent small-molecule degraders could be developed to enable a multimeric neosubstrate to engage two substrate-binding modules with enhanced avidity^40^. Avid neosubstrate binding would enable meaningful TPD pharmacology even for relatively weak ternary complex interactions.

Prodrugs offer a compelling strategy for targeted disease therapy^55,56^. Chemotherapeutics such as capecitabine and irinotecan are now classic examples of tumor-cell-specific metabolic prodrug activation^57^ but this strategy is not common in TPDs aside from a handful of covalent degraders^49–51^ and engineered PROTACs^58–62^. Sulfonyl fluoride chemistry, which is not a conventional feature of prodrugs, confers favorable c-Glue characteristics when activated by electron-deficient groups. The first characteristic is cell permeability of the prodrug but not the activated compound. The second characteristic is dependence of prodrug conversion on reducing equivalents. This prevents premature extracellular activation and may confer selective activity in tumor cells, which harbor elevated GSH levels^63^. Using cancer-specific reactive metabolites to activate prodrugs presents a compelling strategy that may enable targeted accumulation of MGDs in disease tissues and could further expand the therapeutic index enabled by tissue-specific ubiquitin ligase expression. The third and most important characteristic, definitional to c-Glue, is the ability to target a positively charged protein cavity. We propose that a c-Glue strategy could be used to target related E3 ligase families with positively charged pockets that accommodate endogenous phosphodegrons (for example, F-box and SOCS-box proteins)^24,25^ or protein C-terminal acids (that is, C-degron pathway E3 ligases)^26–28^.

In addition to the possibility of applying the c-Glue concept to new small-molecule degraders, the structural similarity to CRBN suggests that YPEL5 could be amenable to an MGD discovery strategy analogous to what has been done for IMiDs. Chemical diversification of a CRBN-binding core has produced a plethora of TPD therapeutics that are now entering clinical trials^2,11^. We speculate that YPEL5-binding ligands would likewise enable chemocentric MGD discovery through the elaboration of an E3-binding chemical core. Such libraries would be intrinsically limited in their activities to cells with preexisting high YPEL5 protein levels (for example, YPEL5 is overexpressed in developing erythrocytes and Ewing sarcomas^43,44^), providing a potential path to a therapeutic window. Indeed, the CTLH complex is an attractive candidate TPD effector because of its frequent upregulation in cancer and broad essentiality^64,65^. The latter characteristic will presumably prevent the rapid emergence of ligase loss-driven resistance. CRBN mimicry by the YPEL5-CTLH ubiquitin ligase, enabled by the development of c-Glues, offers a blueprint for the discovery of next-generation TPD therapeutics.

## Methods

### Chemical synthesis

Additional details are provided in the Supplementary Information.

### General cell biology methods

RPMI-1640 medium, DMEM, Iscove’s modified Dulbecco’s medium (IMDM), FBS, penicillin–streptomycin (10,000 U per ml sodium penicillin G and 10,000 μg ml^−1^ streptomycin), trypsin–EDTA solution (1×) and PBS (1×) were purchased from Gibco (Invitrogen). MG132, MLN-4924, bortezomib and dBET6 were purchased from MedChemExpress. All other chemicals were purchased from Sigma-Aldrich, unless indicated otherwise.

### Mammalian cell culture

Human leukemia (Jurkat and MOLT-4) and kidney epithelial (HEK293T) cell lines were obtained from the American Type Culture Collection. The K562-Cas9 was provided by Z. Tothova (Dana-Farber Cancer Institute) and the Ewing sarcoma cell line (TC-71) was obtained from R. George (Dana-Farber Cancer Institute). Cells were cultured in medium (RPMI-1640 for Jurkat, MOLT-4 and K562 cells; DMEM for HEK293T cells; IMDM for TC-71 cells) supplemented with 10% heat-inactivated FBS, 100 U per ml penicillin, 100 µg ml^−1^ streptomycin, and 0.25 µg ml^−1^ amphotericin B. Cells were incubated at 37 °C with 5% CO_2_ in a humidified atmosphere. *Mycoplasma* testing was performed monthly using the MycoAlert *Mycoplasma* detection kit (Lonza) and all lines were negative.

### Generation of HiBiT–BRD4 cells

Introduction of a HiBiT coding sequence into the endogenous *BRD4* locus in Jurkat cells was achieved using CRISPR–Cas9 genome editing. Alt-R CRISPR RNA (crRNA) and *trans*-activating crRNA (tracrRNA) (Integrated DNA Technologies) were resuspended in nuclease-free duplex buffer (Integrated DNA Technologies) at a concentration of 200 µM each. Equal volumes of crRNA and tracrRNA were mixed (final concentration of 100 µM each) and heated for 5 min at 95 °C. After heating, the complex was gradually cooled to room temperature. The oligo complex was then incubated at room temperature for 20 min with the Alt-R Cas9 nuclease V3 (Integrated DNA Technologies) to form the ribonucleoprotein (RNP) complex. The double-stranded DNA homology-directed repair (HDR) template (HiBiT–BRD4 with extensions, shown below), the RNP complex and an electroporation enhancer (Integrated DNA Technologies) were then electroporated into Jurkat cells using an Amaxa 4D-Nucleofector (Lonza). Electroporated cells were transferred to medium with HRD enhancer (Integrated DNA Technologies). Single cells were subsequently isolated by fluorescence-activated cell sorting and HiBiT expression from individual clones was detected with the Nano-Glo HiBiT lytic detection system (Promega). Sequences for the crRNA and HDR donor are provided below.

>crRNA sequence

ACTAGCATGTCTGCGGAGAG

>HDR donor sequence (+) CATTACTGGCAGATTTCTCAATCTCGTCCCAGGGCCGCTCTCCGCAGAGCCAGAACTCCCGCTAATCTTCTTGAACAGCCGCCAGCCGCTCACCATGCTAGTGATCCCATCACATTCTTCACCAGGCACTCTA

>HDR donor sequence (−) TAGAGTGCCTGGTGAAGAATGTGATGGGATCACTAGCATGGTGAGCGGCTGGCGGCTGTTCAAGAAGATTAGCGGGAGTTCTGGCTCTGCGGAGAGCGGCCCTGGGACGAGATTGAGAAATCTGCCAGTAATG

### HiBiT–BRD4 assay

Endogenous BRD4 protein levels were evaluated using the Nano-Glo HiBiT lytic detection system (Promega). In brief, 1.5 × 10^4^ HiBiT–BRD4 Jurkat cells were seeded into 384-well plates and incubated with the indicated concentrations of compounds. After 5 h, the plates were subjected to a Nano-Glo HiBiT lytic detection system as described in the manufacturer’s manual. The HiBiT–BRD4 assays were conducted in biological triplicates. IC_50_ values were determined using a nonlinear regression curve fit in GraphPad Prism version 10.3.1.

### Reporter vectors

The Cilantro 2 reporter vector (PGK.BsmBICloneSite-10aaFlexibleLinker-eGFP.IRES.mCherry. cppt.EF1α.PuroR; Addgene, 74450) was used for flow-based degradation assays. The following boundaries for BRD4 (UniProt O60885) truncations were used: BRD4_BD1_, residues 44–168; BRD4_BD2_, residues 349–461; BRD4_BD1+BD2_, residues 44–461. Reporter cell lines were generated as described previously^15^.

### BRD4 reporter stability analysis

K562-Cas9 cells expressing the BRD4_BD1_–eGFP, BRD4_BD2_–eGFP or BRD4_BD1+BD2_–eGFP degradation reporters were resuspended at 0.7 × 10^6^ cells per ml. Then, 50 µl of cell suspension was seeded in 384-well plates and immediately treated with DMSO or drug for 16 h. The indicated drugs were dispensed with a D300 digital dispenser (Tecan Genomics). The fluorescence signal was quantified by flow cytometry (FACSymphony flow cytometer, BD Biosciences). Using FlowJo (flow cytometry analysis software, BD Biosciences), the geometric mean of the eGFP and mCherry fluorescent signal for round and mCherry-positive cells was calculated. The ratio of eGFP to mCherry was normalized to the average of ten DMSO-treated controls.

### Bison CRISPR screen for BRD4 stability

The Bison CRISPR library targets 713 E1, E2 and E3 ubiquitin ligases, deubiquitinases and control genes and contains 2,852 guide RNAs (Addgene, 169942)^69^. First, 10% (v/v) of the Bison CRISPR library was added to 10 × 10^6^ BRD4_BD1_–eGFP or BRD4_BD2_–eGFP K562-Cas9 cells and transduced (2,400 rpm, 2 h, 37 °C). Then, 8 days later, cells were treated with drug or DMSO for 16 h and four populations were collected (top 5%, top 5–15%, bottom 5–15% and bottom 5%) on the basis of the mean fluorescence intensity ratio of BRD4–eGFP to mCherry on an MA900 Cell Sorter (Sony). Sorted cells were collected by centrifugation, subjected to direct lysis and amplified as described previously^13^. Amplified single guide RNAs (sgRNAs) were quantified using the Illumina NovaSeq SP platform (Genomics Platform, Broad Institute). The screen was analyzed by comparing stable populations (top 5% eGFP/mCherry expression) to unstable populations (bottom 5% eGFP/mCherry expression) as described previously^13^. Note that, except for RMND5B, the statistically significant hits were the only genes encoding CTLH-related proteins targeted by the Bison library. We speculate that the insensitivity of BRD4 degradation to RMND5B knockout is because of its paralog RMND5A (not targeted by the library) having a redundant role or the two proteins being involved in different suites of CTLH E3 complexes, with those containing RMND5A being responsible for ZZ1-dependent activity.

### Western blotting analysis

Total cells lysates were prepared in 2× sample loading buffer (that is, 250 mM Tris-HCl pH 6.8, 4% SDS, 10% glycerol, 0.006% bromophenol blue, 2% β-mercaptoethanol, 50 mM sodium fluoride and 5 mM sodium orthovanadate). The samples with cell lysates were boiled for 5–8 min at 95 °C. The protein concentrations of the cell lysates were quantified using the BCA method and a BCA protein assay kit (Thermo Fisher Scientific). Equal amounts of protein were subjected to 4–20% SDS–PAGE and transferred to nitrocellulose membranes (Bio-Rad, 1620112). The membranes were blocked using Intercept (Tris-buffered saline) blocking buffer (LI-COR Biosciences) and subsequently probed with appropriate primary antibodies at 4 °C overnight: anti-BRD4 (ab128874, Abcam; 63759; Cell Signaling Technology), anti-BRD3 (ab50818, Abcam), anti-α-tubulin (3873, Cell Signaling Technology), anti-BRD2 (5848, Cell Signaling Technology), anti-c-Myc (5605, Cell Signaling Technology), anti-YPEL5 (PA5-26957, Invitrogen), anti-WDR26 (NBP1-83628, Novus Biologicals), anti-FLAG M2 (F1804, Sigma-Aldrich), anti-Myb (17800-1-AP, Proteintech), anti-RRP36 (25440-1-AP, Proteintech) and anti-RRP36 (11522-1-AP, Proteintech). They were then incubated with IRDye 800-labeled goat anti-rabbit IgG (LI-COR Biosciences, 926-32211) or IRDye 680RD goat anti-mouse IgG (LI-COR Biosciences, 926-68070) secondary antibodies at room temperature for 1 h. After washing the membranes with PBS for 30 min, the membranes were detected on a Li-COR Odyssey CLx system.

### Sample preparation for label-free quantitative MS

Cells were lysed by addition of lysis buffer (8 M urea, 50 mM NaCl, 50 mM EPPS pH 8.5 and protease and Phosphatase inhibitors) and homogenization by bead beating (BioSpec) for three repeats of 30 s at 2,400 strokes per min. A Bradford assay was used to determine the final protein concentration in the clarified cell lysate. Then, 50 μg of protein for each sample was reduced, alkylated and precipitated using methanol–chloroform as previously described^70^ and the resulting washed precipitated protein was allowed to air-dry. Precipitated protein was resuspended in 4 M urea and 50 mM HEPES pH 7.4, followed by dilution to 1 M urea with the addition of 200 mM EPPS pH 8. Proteins were digested with the addition of LysC (1:50, enzyme to protein) and trypsin (1:50, enzyme to protein) for 12 h at 37 °C. Sample digests were acidified with formic acid to a pH of 2–3 before desalting using C18 solid-phase extraction plates (SOLA, Thermo Fisher Scientific). Desalted peptides were dried in a vacuum-centrifuged and reconstituted in 0.1% formic acid for liquid chromatography (LC)–MS analysis.

Data were collected using a TimsTOF HT (Bruker Daltonics) coupled to a nanoElute LC pump (Bruker Daltonics) by a CaptiveSpray nanoelectrospray source. Peptides were separated on a reversed-phase C_18_ column (25 cm × 75 µm inner diameter, 1.6 µM; IonOpticks) containing an integrated CaptiveSpray emitter. Peptides were separated using a 50-min gradient of 2–30% buffer B (acetonitrile in 0.1% formic acid) with a flow rate of 250 nl min^−1^ and column temperature maintained at 50 °C.

The trapped ion mobility spectrometry elution voltages were calibrated linearly with three points (Agilent ESI-L tuning mix ions; 622, 922 and 1,222 *m/z*) to determine the reduced ion mobility coefficients (1/*K*_0_). To perform data-independent acquisition parallel accumulation–serial fragmentation (diaPASEF), we used py_diAID^71^, a Python package, to assess the precursor distribution in the *m/z* ion mobility plane to generate a diaPASEF acquisition scheme with variable window isolation widths aligned to the precursor density in *m*/*z*. Data were acquired using 20 cycles with three mobility window scans each (creating 60 windows) covering the diagonal scan line for doubly and triply charged precursors, with singly charged precursors able to be excluded by their position in the *m*/*z* ion mobility plane. These precursor isolation windows were defined between 350 and 1,250 *m*/*z* with a 1/*K*_0_ range of 0.6–1.45 V s cm^−2^.

### LC–MS data analysis

The diaPASEF raw file processing, control of peptide-level and protein-level false discovery rates (FDRs), assembly of proteins from peptides and protein quantification from peptides were performed using library-free analysis in DIA-NN (version 1.8)^72^. Library-free mode performs an in silico digestion of a given protein sequence database alongside deep-learning-based predictions to extract the DIA precursor data into a collection of MS2 spectra. The search results are then used to generate a spectral library that is then used for the targeted analysis of the DIA data searched against a SwissProt human database (January 2021). Database search criteria largely followed the default settings for directDIA including tryptic with two missed cleavages, carbamidomethylation of cysteine and oxidation of methionine, with a precursor *Q* value (FDR) cutoff of 0.01. The precursor quantification strategy was set to robust LC (high accuracy) with retention-time-dependent cross-run normalization. Proteins with a low sum of abundance (<2,000 multiplied by the number of treatments) were excluded from further analysis and resulting data were filtered to only include proteins that had a minimum of three counts in at least four replicates of each independent comparison of treatment sample to the DMSO control. Proteins with missing values were imputed by random selection from a Gaussian distribution either with a mean of the nonmissing values for that treatment group or with a mean equal to the median of the background (in cases when all values for a treatment group were missing) using in-house scripts in the R framework (R Development Core Team, 2014). Significant changes comparing the relative protein abundance of these treatments to the DMSO control were assessed by moderated *t*-test as implemented in the limma package within the R framework^73^.

### Coimmunoprecipitation

HEK293T cells were seeded into a six-well plate (3 × 10^5^ cells per well), cultured overnight and then transfected with 1.5 μg of FLAG-tagged BRD4 plasmid using TransIT-LT1 transfection reagents (Mirus Bio). The transfected cells were cultured for another 36 h, pretreated with 1 μM bortezomib and cotreated with either compound or DMSO for 4 h before collection. The cells were collected and lysed in Pierce IP lysis buffer (Thermo Fisher Scientific) with cOmplete mini protease inhibitor cocktail (Roche) for 30 min on ice and centrifuged for 30 min at 4 °C to remove the insoluble fraction. For immunoprecipitation, 20 μl of precleaned anti-FLAG M2 magnetic beads (Sigma-Aldrich) were added to the lysates. The beads–lysate mix was incubated at 4 °C overnight on a rotator. Beads were magnetically removed and washed three times with PBS and the FLAG-tagged protein was competitively eluted using 3× FLAG peptide (ApexBio Technology). Immunoblotting was carried out as previously described.

### Construction of plasmids

A gBlock (Integrated DNA Technologies) containing a codon-optimized YPEL5–Gly-Ser–FLAG coding sequence was cloned by Gibson assembly into a lentiviral transfer vector (pN106, gift from S. Gourisankar). The construct features the EF1α promoter to drive transgene expression and the mouse Pgk-1 promoter to drive the expression of the Blasticidin resistance gene. *YPEL5* mutations were introduced by overlap extension PCR followed by reassembly with the original cut vector. sgRNA constructs were assembled according to standard procedures as described for BsmBI-cut pXPR_023 (gift from S. Corsello; example in Addgene, 202447). The destination lentiviral transfer vector contains an sgRNA cloning site (U6 promoter) and Cas9-FLAG-P2A-PuroR (EF1α promoter). Codon-optimized YPEL5 and sgRNA sequences are provided below.

>YPEL5_gs_FLAG_opt

ATGGGAAGGATCTTTTTGGATCATATTGGAGGGACACGGCTGTTCTCTTGTGCTAACTGCGATACAATCTTGACAAACAGGTCCGAACTGATCTCTACGAGGTTTACGGGAGCAACCGGCAGAGCGTTCTTGTTTAACAAGGTCGTAAATCTCCAGTATTCCGAGGTACAGGATCGCGTCATGCTCACCGGGAGACACATGGTCAGGGACGTGTCCTGCAAGAACTGTAATTCCAAACTCGGCTGGATTTATGAATTCGCAACAGAGGATTCACAAAGATATAAAGAAGGGCGCGTCATTTTGGAACGAGCACTTGTGCGAGAATCCGAGGGTTTTGAAGAGCACGTCCCGTCTGATAACTCAGGTAGCGATTACAAGGACGACGATGACAAGtaa

>sgNEG

caccgGGTGTGCGTATGAAGCAGTG

>WDR26_sg_1_f

caccgCAGCCTGAATGTCAATAACG

>WDR26_sg_2_f

caccgGAGAGTCTGTAAACGCCGTG

>WDR26_sg_3_f

caccgCCTCTTACCACAATAGCATG

>WDR26_sg_4_f

caccgGACATCCTGACTCTTGCATG

>YPEL5_sg_1_f

caccgAGTACAGTGAAGTTCAAGAT

>YPEL5_sg_2_f

caccgAGTGGCGCCTGTGAAACGAG

>YPEL5_sg_3_f

caccgGTTTGCACAAGAAAACAGAC

### Generation of knockout cells

HEK293T cells were transfected with either pXPR_023/WDR26 or pXPR_023/YPEL5, along with pMD2.G (Addgene, 12259) and psPAX2 (Addgene, 12260), using TransIT-LT1 transfection reagents (Mirus Bio) to produce lentivirus. After 48 h of transfection, the supernatant was collected, filtered and added to HiBiT–BRD4 Jurkat cells in the presence of 5 µg ml^−1^ polybrene (APExBIO, K2701). Following 48 h of infection, the infected cells were passaged and cultured in medium containing 2 µg ml^−1^ puromycin (Gibco, A11138-03) for 3 days. The surviving cells were grown for an additional 5 days. The knockout was confirmed by immunoblotting using either anti-WDR26 or anti-YPEL5 antibody.

### Generation of *YPEL5*-mutant cells

HEK293T cells were transfected with either pN106/YPEL5-FLAG-WT, pN106/YPEL5-FLAG-T36A, pN106/YPEL5-FLAG-R41A or pN106/YPEL5-FLAG-L62A, along with pMD2.G (Addgene, 12259) and psPAX2 (Addgene, 12260), using TransIT-LT1 transfection reagents (Mirus Bio) to produce lentivirus. After 48 h of transfection, the supernatant was collected, filtered and added to YPEL5^−/−^ HiBiT–BRD4 Jurkat cells in the presence of 5 µg ml^−1^ polybrene (APExBIO, K2701). Following 48 h of infection, the infected cells were passaged and cultured in medium containing 10 µg ml^−1^ blasticidin (Gibco, A11139-03) for 5 days. The surviving cells were grown for an additional 7 days. The reexpression of YPEL5 was confirmed by immunoblotting using anti-FLAG antibody.

### Cloning and recombinant protein preparation

The complementary DNA (cDNA) of full-length BRD4 was a gift from P. Howley (Addgene, 14447)^74^, whereas those of BRD2 and BRD3 were acquired from an in-house human cDNA library (Max Planck Institute of Biochemistry). All new constructs were generated with the Gibson assembly method^75^ and mutagenized using the QuickChange site-directed mutagenesis protocol (Agilent). To prepare multigene DNA constructs for insect cell expression, multiple inserts encoding the CTLH E3 subunits were combined into single baculoviral expression vectors with the biGBac assembly method^76^. All plasmids used in this study are listed in the Supplementary Table 5.

#### Insect cell expression and purification

All CTLH E3 complexes and the E1 enzyme UBA1 were expressed in *Trichoplusia* *ni* High Five insect cells (Thermo Fisher Scientific) following three rounds of baculovirus amplification in *Spodoptera* *frugiperda* Sf9 cells (Thermo Fisher Scientific). The infected High Five cells were grown in EX-CELL 420 serum-free medium at 27 °C for 72 h before harvesting by centrifugation (15 min, 450*g*) and resuspension of the pelleted cells in the cold lysis buffer containing 50 mM Tris pH 8, 150 mM NaCl, protease inhibitors (10 μg ml^−1^ leupeptin, 20 μg ml^−1^ aprotinin, EDTA-free cOmplete protease inhibitor tablet (Roche, one tablet per 50 ml of buffer) and 1 mM PMSF) and 5 mM DTT. The resuspended cells were disrupted by sonication and centrifuged (30 min, 20,000*g*) to remove cell debris.

All versions of the CTLH E3 assemblies harbored a Twin-Strep tag fused to the TWA1 C terminus^40^ and were affinity-purified from insect cell lysates by Strep-Tactin affinity (IBA Lifesciences). The eluted proteins were subjected to size-exclusion chromatography (SEC) with a Superose 6 Increase 10/300 GL column (Cytiva) in the final buffer containing 25 mM HEPES pH 7.5, 150 mM NaCl and 1 mM DTT (buffer A, for biochemical assays) or 5 mM DTT (buffer B, for cryo-EM). The WDR26^ΔCTLH^ module (alone and in complex with YPEL5) was expressed as an N-terminal GST fusion. Proteins were purified by GST affinity chromatography (Cytiva) and incubated with tobacco etch virus (TEV) protease (4 °C, overnight) to liberate the GST tag. The digested samples were subjected to SEC with a Superdex 200 10/300 GL column (Cytiva).

#### Bacterial expression and purification

Substrates (the BET-family proteins and NMNAT1) and other reagents for biochemical assays (GID4 substrate receptor, E2 enzyme UBE2H and ubiquitin) were expressed in the codon-enhanced *Escherichia* *coli* BL21 (DE3) RIL cells. Bacterial cells were transformed with respective plasmids (Supplementary Table 5) and grown in Terrific Broth medium supplemented with appropriate antibiotics at 37 °C until an optical density of 0.6. After lowering the temperature to 18 °C, protein expression was induced with 0.4 mM IPTG and carried out for 18 h. Cells were collected by centrifugation (15 min, 4,500*g*) and resuspended in the cold lysis buffer containing 50 mM Tris pH 8, 150 mM NaCl, 1 mM PMSF and 5 mM DTT.

All versions of BRD2, BRD3 and BRD4, as well as UBE2H, were expressed as N-terminal GST fusions and purified analogously to the insect-cell-expressed GST–TEV–WDR26^ΔCTLH^ (described above). To fully phosphorylate the C-terminal extension of UBE2H for ubiquitylation assays (which boosts its CTLH E3-dependent activity), it was coexpressed with the catalytic subunit of the kinase CK2 (CK2α–6×His)^39^ and subjected to anion-exchange chromatography with the HiTrap Q HP column (Cytiva) before final SEC in buffer A.

The 6×His tag was appended to the N or C termini of GID4 (∆1–99) and NMNAT1. After resuspending bacterial cells in the lysis buffer supplemented with 10 mM imidazole, proteins were captured with the Ni-NTA affinity chromatography (Sigma-Aldrich) and subjected to SEC in buffer A.

WT ubiquitin was expressed in a tagless form and purified with the glacial acetic acid method followed by gravity cation-exchange chromatography (GE Healthcare) and SEC as described previously^77^.

### Fluorescence labeling of substrates for biochemical assays

All substrates were fluorescently labeled by sortase A-mediated fusion^78^ of fluorescein (FAM) or TAMRA-containing peptides to the proteins’ N-terminal glycine exposed upon cotranslational cleavage of initiator methionine (for GG–NMNAT1–6×His) or TEV-mediated removal of the GST tag (for GGSGS–BRD2/BRD3/BRD4). Labeling reactions were performed for 30 min at room temperature by mixing 50 μM substrate, 250 μM fluorescent peptide (fluorophore–GSGG–LPETGG, synthesized in the Max Planck Institute of Biochemistry (MPIB) Bioorganic Chemistry and Biophysics Core Facility) and 10 μM sortase A–6×His in reaction buffer containing 50 mM Tris-HCl pH 8, 150 mM NaCl and 10 mM CaCl_2_. The fluorescent substrates were purified by SEC in buffer A.

### Biotinylation of E3 ligase modules for BLI binding assays

To prepare site-specifically biotinylated versions of WDR26^ΔCTLH^ and YPEL5–WDR26^ΔCTLH^ for BLI assays, biotin was conjugated to the AviTag attached to the WDR26^ΔCTLH^ C terminus in the BirA ligase-catalyzed reaction. Biotinylation was performed by mixing 50 μM protein, 1× reaction buffer (50 mM Bicine pH 8.3, 10 mM ATP, 10 mM magnesium acetate and 1 mM biotin) and BirA (at 1:100 BirA-to-protein molar ratio). The mixture was incubated overnight at 4 °C and purified over SEC on the Superdex 200 column in buffer A. Successful biotinylation was confirmed by intact MS (performed in the MPIB MS Core Facility).

### Native PAGE band-shift assay

For initial qualitative analysis of ZZ1-induced ternary complex formation (Fig. 1f), 1 μM FAM-labeled BRD4_BD1_ was incubated with 3 μM WDR26^ΔCTLH^ or YPEL5–WDR26^ΔCTLH^ in the presence of 10 μM JQ1 (Sigma-Aldrich) or ZZ1 (at 2% final DMSO concentration) for 1 h in buffer A. Samples were mixed with a nondenaturing PAGE loading buffer containing 5% (v/v) glycerol, bromophenol blue and Tris–borate (TB) buffer (100 mM Tris and 100 mM boric acid) and run on a native PAGE gel (prerun at 200 V for 5 min) for 50 min (130 V, 4 °C) in TB buffer. Ternary complex formation was assessed by monitoring the downward shift of FAM–BRD4_BD1_ (detected in a fluorescence scan) toward the faster-migrating band corresponding to YPEL5–WDR26^ΔCTLH^ (visualized by Coomassie staining).

The native gel was freshly poured and contained 4.5% (w/v) acrylamide and bis-acrylamide (29:1), 2% (v/v) glycerol, TB buffer, TEMED (75 μl in 100 ml, gel solution) and 0.04% (w/v) APS.

### In vitro ubiquitylation assays

All ubiquitylation assays were performed at room temperature in the final reaction buffer containing 25 mM HEPES pH 7.5, 150 mM NaCl, 0.25 mM DTT, 5 mM ATP and 10 mM MgCl_2_. Samples at indicated time points were quenched by mixing an aliquot of the total reaction mixture with a reducing Laemmli buffer and subjected to SDS–PAGE. Substrate ubiquitylation was visualized by a fluorescence scan of SDS–PAGE gels using the Amersham Typhoon Imager 600 (GE Healthcare).

The initial ubiquitylation assays identifying the ZZ1-co-opted CTLH E3 catalytic assembly and the targeted BRD4 construct (Fig. 1e and Extended Data Fig. 3a,f) were set up by first mixing 0.5 μM CTLH E3 (catalytic core or its supramolecular WDR26-dependent version assembled with different substrate receptor modules; summarized in Extended Data Fig. 2b), 0.25 μM fluorescent substrate (FAM-labeled BRD4 or TAMRA-labeled NMNAT1), 1 μM phosphorylated UBE2H and 10 μM JQ1 or ZZ1 (at 2% final DMSO concentration). The mixture was incubated at room temperature for 1 h and supplemented with 0.2 μM E1 UBA1 and 20 μM ubiquitin to initiate the reaction. To probe the effect of the N-degron substrate receptor GID4 (Extended Data Fig. 3a,f), 1 μM GID4 (∆1–99) was included in the reaction mixtures containing the CTLH E3s assembled with the GID4 adaptor subunit ARMC8.

Having identified YPEL5 as the ZZ1-induced neosubstrate receptor, all the following assays were performed with the YPEL5–CTLH E3 (the assembly of RANBP9–TWA1–ARMC8–RMND5A–MAEA–WDR26–YPEL5) in the analogous reaction conditions. In assays testing the ability of various compounds to induce neosubstrate ubiquitylation (Extended Data Fig. 6b), the master mix containing 0.5 μM YPEL5–CTLH E3, 0.25 μM FAM–BRD4_BD1_ and 1 μM phosphorylated UBE2H was first prepared and then mixed with 10 μM JQ1 or a given degrader derivative (at 2% final DMSO concentration). To test the structurally identified BRD4_BD1_ loop for dictating ZZ1 specificity (Extended Data Fig. 7i), the master mix containing 0.5 μM YPEL5–CTLH E3, 10 μM ZZ1-SO_2_H and 1 μM phosphorylated UBE2H was assembled and then combined with 0.25 μM fluorescent substrate. The mixes were incubated for 15 min at room temperature and supplemented with 0.2 μM E1 UBA1 and 20 μM ubiquitin to initiate the reaction.

To ensure an equal level of complex subunits in the assay querying structure-based YPEL5 mutants (Fig. 4c and Extended Data Fig. 10j), the YPEL5–CTLH E3 complexes were expressed by coinfection of High Five cells with two baculoviruses encoding (1) WDR26–CTLH E3 assembly (RANBP9–TWA1–RMND5A–MAEA–WDR26) and (2) WT or mutant YPEL5. The identity of the coexpressed YPEL5 mutants was confirmed by intact mass analysis, whereas their similar level was assessed by visual inspection of Coomassie-stained SDS–PAGE gels after ubiquitylation reaction. To set up the assay, the master mix containing FAM–BRD4_BD1_, 10 μM ZZ1-SO_2_H and 1 μM phosphorylated UBE2H was first prepared and then combined with 0.5 μM YPEL5–CTLH E3 (containing WT or mutant YPEL5). The mixtures were incubated for 15 min at room temperature and supplemented with 0.2 μM E1 UBA1 and 20 μM ubiquitin to initiate the reaction.

### Intact MS analyses probing the ZZ1 mode of action

To analyze whether ZZ1’s mode of action involves the formation of a covalent adduct (Extended Data Fig. 5b), we incubated 5 μM YPEL5–WDR26^ΔCTLH^ with 10 μM BRD4_BD1_ and 20 μM ZZ1 (at 2% final DMSO concentration) in buffer A at room temperature overnight and analyzed the samples by intact MS (performed in the MPIB MS Core Facility).

To monitor the DTT-triggered conversion of degraders to their sulfinic acid versions (Fig. 3b and Extended Data Figs. 9d and 10g), DMSO stocks of ZZ1 or ZZ2 were incubated with buffer B (containing 5 mM DTT) for 2 h at room temperature and analyzed by intact MS along with their DMSO-diluted controls.

### FP assays

#### Quantification of ternary complex formation

To quantify ternary complex formation (Figs. 3d and 5e), we used FP assay monitoring association of the fluorescent FAM–BRD_BD1_ tracer with YPEL5–WDR26^ΔCTLH^ upon degrader titration. To set up binding reactions, the mixtures of 20 nM FAM–BRD_BD1_ and 100 nM YPEL5–WDR26^ΔCTLH^ or WDR26^ΔCTLH^ in the binding buffer (25 mM HEPES pH 7.5, 150 NaCl, 5 mM DTT, 1 mg ml^−1^ BSA and 0.1% Tween-20) were combined with equal volumes of a twofold dilution series of the degraders or JQ1 (prepared in DMSO and then diluted with the binding buffer to reach the final DMSO concentration of 2% for all titration points) and incubated for 1 h at room temperature before transferring to 384-well flat-bottom black plates (Greiner). FP was calculated by measuring perpendicular and parallel fluorescence intensity values using the excitation and emission wavelengths of 482 nm and 530 nm, respectively, in a CLARIOstar microplate reader (BMG Labtech). Polarization values obtained from three independent experiments were plotted in GraphPad Prism version 10.3.1. The potency of the degraders was expressed as the EC_50_ estimated by nonlinear regression using a four-parameter [agonist] versus response model. Because of the substantially enhanced reactivity of ZZ2, its sulfinic acid version was prepared by 1 h incubation with the binding buffer before mixing with the protein mix and analyzed by intact MS to confirm the conversion (Extended Data Fig. 10g).

#### Real-time FP monitoring the rate of ternary complex formation

To probe relative rates of ternary complex formation induced by the nonconverted and sulfinic acid versions of degraders (Extended Data Fig. 5c and Fig. 5e), FP was monitored upon combining the mixture of 10 nM FAM–BRD_BD1_ and 50 nM YPEL5–WDR26^ΔCTLH^ in the binding buffer (containing 1 mM DTT) with 10 μM DMSO-dissolved ZZ1, ZZ2 and their sulfinic acid derivatives (resulting in the final 2% DMSO concentration). Contact of nonconverted degraders with the DTT-containing buffer initiated their activation, resulting in a gradual increase in polarization. Experiments were also performed with ZZ1 and ZZ2 preincubated with the binding buffer before adding them to the protein mixture. An analogous FP assay was performed to query the impact of DTT concentration on the rate of ternary complex formation (Extended Data Fig. 5e). Here, the concentration of DTT in the binding buffer used for preparing the tracer–E3 ligase mixture varied from 0.1 to 5 mM.

#### Competitive FP probing cooperative E3 ligase–neosubstrate interactions

To test whether the tight ternary complex is driven by cooperative degrader-induced E3 ligase–target interactions (Fig. 4e), we monitored propensities of ZZ1-SO_2_H and JQ1 to displace the BRD4_BD1_-bound JQ1–FITC tracer (Tocris Bioscience) in the presence and absence of YPEL5–WDR26^ΔCTLH^. To determine conditions for the competitive FP assay, we first performed the binary binding experiment in a noncompetitive format (Extended Data Fig. 9g). A twofold dilution series of BRD4_BD1_ prepared in the binding buffer was mixed with equal volumes of 20 nM JQ1–FITC. Polarization data from three independent experiments were fitted to the one-site binding model in GraphPad Prism version 10.3.1 to determine the *K*_D_ for JQ1–BRD4_BD1_ interactions. The analogous assay was performed to analyze the impact of exchanging the structurally-determined BRD4_BD2_ region with the corresponding YPEL5/degrader-contacting loop of BRD4_BD1_ on engaging the parental JQ1 inhibitor (Extended Data Fig. 7f).

To ensure sufficient signal for the competitive assay, we identified BRD4_BD1_ concentration at which the FP signal reaches ∼60% saturation (that is 30 nM). Subsequently, a 2-fold dilution series of unlabeled competitors ZZ1-SO_2_H or JQ1 (prepared in DMSO and then diluted with the binding buffer to reach the final DMSO concentration of 2% for all titration points) was combined with equal volumes of the mixture of 20 nM JQ1–FITC and 60 nM BRD4_BD1_ alone or premixed with 600 nM YPEL5–WDR26^ΔCTLH^. This led to displacement of JQ1–FITC from BRD4_BD1_, thus decreasing FP. The values of polarization from three independent experiments were plotted in GraphPad Prism version 10.3.1. The four-parameter [inhibitor] versus response model was applied to estimate the IC_50_, reflecting relative binding strengths of the titrated competitors toward BRD4_BD1_. The extent of cooperativity was expressed as the apparent cooperativity factor (α_app_) defined as a ratio between the IC_50_ values in the absence and presence of the harnessed E3 ligase.

#### Competitive FP probing the binary degrader–YPEL5/BRD4_BD1_ interactions

As a measure of the relative strength of degrader–YPEL5 and degrader–BRD4_BD1_ interactions, we measured the propensity of the YPEL5-interacting and BRD4_BD1_-interacting parts of ZZ1-SO_2_H to disrupt the ternary complex (comprising FAM–BRD_BD1_, YPEL5–WDR26^ΔCTLH^ and ZZ1-SO_2_H) (Extended Data Fig. 9f). To ensure sufficient signal for the competitive assay, we identified the ZZ1-SO_2_H concentration at which the FP signal reached ∼60% saturation (that is, 110 nM). A twofold dilution series of unlabeled competitors JQ1 and the YPEL5-interacting part of ZZ1-SO_2_H (at 2% final DMSO concentration for all titration points) was combined with equal volumes of the mixture of 20 nM FAM–BRD_BD1_, 100 nM YPEL5–WDR26^ΔCTLH^ and 220 nM ZZ1-SO_2_H. The sulfinic acid version of the YPEL5-engaging ZZ1-SO_2_H fragment (synthesized in the sulfonyl fluoride form) was prepared by its overnight incubation with the DTT-containing binding buffer before mixing with the protein mix and analyzed by intact MS to confirm the conversion (Extended Data Fig. 9d). The values of polarization from three independent experiments were plotted in GraphPad Prism version 10.3.1. The four-parameter [inhibitor] versus response model was applied to estimate the IC_50_.

#### Octet BLI assays

To quantify interactions between the degrader-bound neosubstrates and the CTLH E3 neosubstrate receptor module, we determined the *K*_D_ using Octet BLI. Briefly, 5 μg ml^−1^ of the biotinylated ligands (WDR26^ΔCTLH^ or YPEL5–WDR26^ΔCTLH^) were immobilized on the Streptavidin biosensors (Sartorius), washed and equilibrated in the BLI binding buffer. The association step was performed by dipping the loaded sensors in the threefold dilution series of the neosubstrate analytes in the presence of the constant saturating degrader concentration (5 μM ZZ1-SO_2_H or ZZ2-SO_2_H), followed by dissociation in the binding buffer (Extended Data Figs. 8b, 9a and 10f). To query the intrinsic affinity between BRD4_BD1_ and the CTLH E3 modules, the association step was performed without the degrader (Extended Data Fig. 9c). Similarly, the ability of ZZ1-SO_2_H to engage YPEL5 on its own was tested by performing the assay in the absence of BRD4_BD1_ by titrating the degrader (Extended Data Fig. 9e). For qualitative comparison of YPEL5–WDR26^ΔCTLH^ binding to the BRD4_BD1/BD2_ WT and loop mutants, BLI experiments were performed at a single neosubstrate concentration of 100 nM in the presence of 5 μM ZZ1-SO_2_H (Extended Data Fig. 7d,g).

For each measurement, one of the loaded sensors was dipped in the reference solution containing 0 or 5 μM degrader in the BLI binding buffer (in the absence of the analyte protein). The reference response values were subtracted from the responses elicited by all the neosubstrate-containing solutions before data analysis. All BLI experiments were carried out at 20 °C using the eight-channel Octet R8 system (Sartorius). Concentrated proteins and compounds were diluted in the BLI binding buffer composed of 25 mM HEPES pH 7.5, 150 mM NaCl, 1 mM DTT and 0.05% Tween-20.

The raw data were processed with Octet analysis software (Sartorius) by applying a global fitting with linked *R*_max_ values (assuming a 1:1 binding model). The equilibrium response wavelength shifts from three independent measurements were extracted and plotted in GraphPad Prism version 10.3.1 for steady-state analysis. *K*_D_ was estimated by nonlinear regression using the one-site binding model.

### Single-particle cryo-EM

#### Sample preparation and imaging

The YPEL5–CTLH E3 for structural studies was expressed by coinfection of High Five cells with three separate baculoviruses encoding (1) the CTLH E3 catalytic core (RANBP9–TWA1–RMND5A–MAEA); (2) WDR26; and (3) YPEL5). The peak fractions (excluding those partially overlapping with the void volume peak) of the SEC-purified complex were pooled and concentrated to 6 mg ml^−1^. The cryo-EM sample was prepared by combining 2.7 μM (2.5 mg ml^−1^) YPEL5–CTLH E3, 11 μM BRD4_BD1_ and 20 μM ZZ1 or ZZ2 in buffer B (containing 5 mM DTT) and incubating the mixture for 1 h on ice. Shortly before plunging, samples were supplemented with the 0.1% octyl-β-glucoside detergent (Sigma-Aldrich)^44^, which mitigates complex disassembly and protein aggregation during plunging and facilitates particle distribution.

Cryo-EM grids were prepared using Vitrobot Mark IV (Thermo Fisher Scientific) operated at 4 °C and 100% humidity. Then, 3.5 μl of samples were applied to glow-discharged holey carbon R1.2/1.3, Cu 300-mesh grids (Quantifoil), blotted with Whatman no. 1 filter paper (blot time: 3 s, blot force: 3) and vitrified by plunging into liquid ethane.

#### Data collection and processing

Details of cryo-EM data collection are listed in Supplementary Table 2, whereas the flowchart of data processing workflow is presented in Supplementary Fig. 1 and 2.

Grids were first prescreened on either a Talos Arctica or Glacios transmission electron microscope (Thermo Fisher Scientific) operated at 200 kV, equipped with a Falcon III (Thermo Fisher Scientific) or K2 (Gatan) direct electron detector, respectively. The high-resolution datasets were acquired on a Titan Krios microscope (Thermo Fisher Scientific) operated at 300 kV, equipped with a postcolumn GIF and a K3 Summit direct electron detector (Gatan) operating in a counting mode. The automated data acquisition was carried out with SerialEM^79^, while the Focus software^80^ was used for on-the-fly discarding of poor-quality images and data preprocessing.

Video frames were motion-corrected with dose-weighting using MotionCor2 (ref. ^81^) and subjected to estimation of the contrast transfer function (CTF) with Gctf^82^ integrated in Focus. Particles were automatically picked with Gautomatch (K. Zhang) using our previously published map of the YPEL5-CTLH E3 (EMD-18170)^44^ as a template. All the subsequent stages of data processing were carried out with RELION (versions 4.0 and 5.0)^83,84^. To clean up the data while preserving particle views corresponding to less populated orientations, the extracted particles were subjected directly to three rounds of unmasked three-dimensional (3D) classification. The final classification revealed the coexistence of multiple types of BRD4_BD1_-bound YPEL5-CTLH E3 assemblies with similar shapes and dimensions but differing stoichiometries of the catalytic and YPEL5–WDR26 modules as previously reported^44^. The compositional heterogeneity and dynamic nature of the supramolecular CTLH E3 ligases limit the resolution of their overall maps^39^. To gain molecular insights into the architecture of the ternary complex, we generated masks around the BRD4_BD1_–YPEL5–WDR26-containing regions in the 3D refined maps of each type of the CTLH E3 assembly and extracted the encompassed densities by signal subtraction. In doing so, we not only overcame the challenge of complex heterogeneity but also substantially increased the number of particles available for subsequent 3D classifications. The generated pools of signal-subtracted particles were joined, 3D-classified and aligned by 3D refinement with a mask over the BRD4_BD1_-bound YPEL5–WDR26 and the better-resolved copy of the scaffolding module (RANBP9–TWA1). The map was further improved by local refinement with a mask around the BRD4_BD1_-bound YPEL5–WDR26 module. Additional rounds of focused 3D classification (without particle alignment) enriching for particles with the most complete and well-resolved features followed by local refinements with Blush regularization^85^ yielded the final high-resolution reconstructions permitting model building. For the map of the ZZ2-SO_2_H-induced ternary complex, the final round of local refinement was preceded by CTF refinement. Maps were postprocessed by *B*-factor sharpening and high-resolution noise substitution in RELION. To aid in building the atomic models, the refined maps were also sharpened with DeepEMhancer^67^ (using the ‘highRes’ model) and are deposited as additional maps in the EM Data Bank. The estimated resolutions of the final maps are based on the gold-standard Fourier shell correlation cutoff of 0.143.

#### Model building, refinement and analysis

Atomic models were manually built and refined with Coot (version 0.9.8.7)^86^, whereas degrader coordinates and the corresponding restraints applied during model refinement were generated using the JLigand^87^ interface. The refinement and validation statistics of the built models are listed in Supplementary Table 2.

The structure of the ZZ1-SO_2_H-induced ternary complex was obtained by first docking the previous coordinates of the YPEL5–WDR26 module (PDB 8QBN)^44^ and BRD4_BD1_ (PDB 3MXF)^34^ in UCSF Chimera (version 1.11.2)^88^ into the postprocessed map and manually refining the differing parts (for example, removing the parts of the BRD4 BD facing away from the YPEL5 interface, which are less ordered and, thus, blurry or invisible in the high-resolution reconstruction). This left an unoccupied segment of electron density filling the canonical acetyl-lysine-binding pocket of BRD4_BD1_ and the central basic groove of YPEL5 that supported fitting ZZ1-SO_2_H. The coordinates of the -SO_2_H group (wherein the sulfur center adopts the pyramidal geometry and features the lone pair of electrons) were oriented to best fit the map while maximizing polar interactions with the surrounding YPEL5 side chains. Prominent blobs of electron density at the three-way interface of ZZ1-SO_2_H chloro, YPEL5 and BRD4_BD1_ were assigned as water molecules, which appear to participate in the formation of a cooperative ternary complex. To obtain the structure of the ternary complex with the improved degrader ZZ2-SO_2_H, we docked the coordinates of the ZZ1-SO_2_H-driven complex and manually refined differing areas. As in the first structure, the degrader density was clearly resolved (including the region corresponding to the appended chloro group), which enabled its unambiguous fitting.

Both models were subjected to iterative rounds of manual building and real-space refinement in PHENIX (version 1.21.1)^89^ until a satisfactory model quality, in terms of geometry and agreement with the map, was achieved. Configurations of the zinc-binding sites within WDR26 and YPEL5, as well as of the degraders, were restrained during real-space refinement. The final model was validated with MolProbity^90^. Structure visualization and analyses were carried out with UCSF Chimera (version 1.11.2)^88^, UCSF ChimeraX (version 1.7.1)^91^ and PyMOL (version 3.0.3; Schrödinger). The surface area of the YPEL5–BRD4_BD1_ interface in the ternary complex structure was calculated with the PISA version 1.52 tool of the European Bioinformatics Institute^48^.

### LC–MS detection of ZZ1

Following treatment of cells with 5 μM of ZZ1 for 5 h, cells were washed two times with PBS and lysed in 100 μl of a 2:1:1 mixture of acetonitrile, methanol and water. The cell pellets were vortexed well to ensure complete lysis and centrifuged at 4 °C for 10 min at 15,000 rpm; the supernatant was transferred to an LC–MS vial.

Untargeted metabolomics measurements were performed using an Agilent 6530 Quadrupole time-of-flight LC–MS instrument. MS analysis was performed using electrospray ionization (ESI) in positive mode. The dual ESI source parameters were set as follows: gas temperature, 250 °C; the drying gas flow, 8 L min^−1^; nebulizer pressure, 25 psi; sheath gas temperature, 300 °C; sheath gas flow, 12 L min^−1^; capillary voltage, 3,500 V; fragmentor voltage, 175 V. Separation of metabolites was conducted using an Eclipse Plus C18 LC column (Agilent, 959961-902). Mobile phases were as follows: buffer A, water with 0.1% formic acid; buffer B, acetonitrile with 0.1% formic acid. The LC gradient started at 70% B, increased to 100% B from 0 to 4 min and was then held constant at 100% B from 4 to 5 min before going back down to 70% B from 5 to 5.1 min. The flow rate was at a constant 0.7 ml min^−1^ throughout the run.

### Statistics and reproducibility

All western blot analyses and ubiquitination assays were independently repeated to ensure reproducibility. Western blot analyses were performed using lysates from at least two independent biological replicates per condition. Representative blots are shown and all independent experiments yielded consistent results. No samples or data points were excluded from the analysis and all representative images were selected on the basis of reproducibility across independent experiments.

### Reporting summary

Further information on research design is available in the Nature Portfolio Reporting Summary linked to this article.

## Online content

Any methods, additional references, Nature Portfolio reporting summaries, source data, extended data, supplementary information, acknowledgements, peer review information; details of author contributions and competing interests; and statements of data and code availability are available at 10.1038/s41589-026-02182-5.

## Supplementary information

Supplementary Information Supplementary Figs. 1–4, Tables 1–5, chemical synthesis procedure and characterization and uncropped Blot for Supplementary Figs. 3 and 4.

Reporting Summary

## Source data

Source Data Figs. 1 and 3–5 and Extended Data Figs. 1, 4, 6–8 and 10 Uncropped images and statistical source data.

Source Data Figs. 1 and 3–5 and Extended Data Figs. 1 and 4–10 Uncropped images and statistical source data.

## Data Availability

Data generated in this study are provided in the manuscript and its Supplementary Information. Cryo-EM density maps were deposited to the EM Data Bank with accession codes EMD-54216, EMD-54215 and EMD-54194. The atomic models were deposited to the PDB with the accession codes 9RSD and 9RSC. Source data are provided with this paper.
