# Supplementary material for: Charged molecular glue discovery enabled by targeted degron display
Source: Nat Chem Biol. 2026 Apr 6;22(9):1446–55. doi: 10.1038/s41589-026-02182-5 (PMC13355314; doi:10.1038/s41589-026-02182-5)

nature chemical biology

Supplementary information

https://doi.org/10.1038/s41589-026-02182-5

Charged molecular glue discovery enabled by targeted degron display

In the format provided by the authors and unedited

Table of Contents

Supplementary Figures	4
Supplementary Tables	10
General Information	16
General Procedure of preparing c-Glues	17
Preparation of c-Glues	18
GSH chemical stability of ZZ1 and ZZ2	66
Reference	67
^{1}H and ^{13}C NMR Spectra and LC trace	68
Uncropped Blot for Supplementary Figures	72

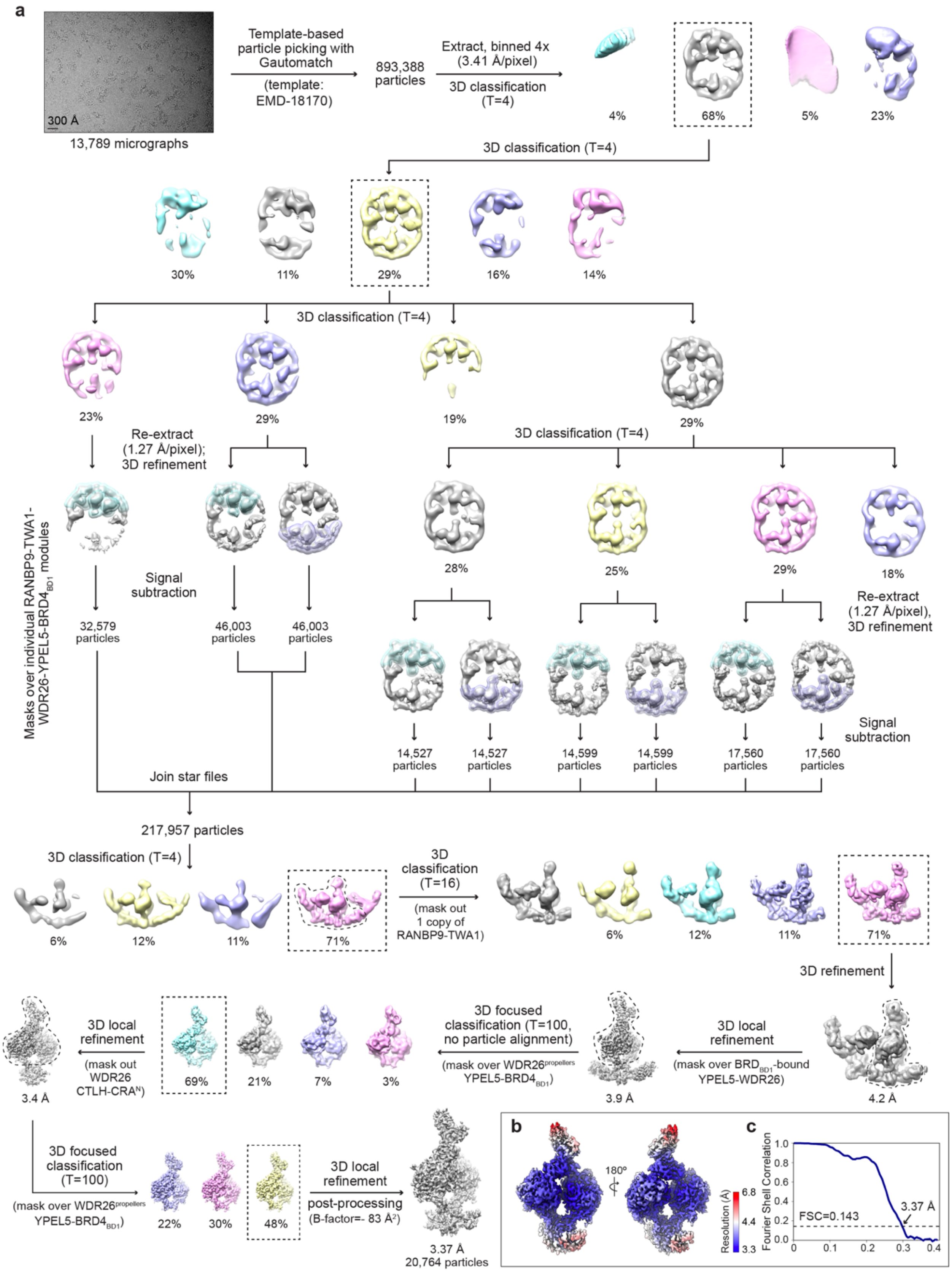
a
300 Å
13,789 micrographs
Template-based particle picking with Gautomatch
(template: EMD-18170)
893,388 particles
Extract, binned 4x (3.41 Å/pixel)
3D classification (T=4)
4%
68%
5%
23%
3D classification (T=4)
30%
11%
29%
16%
14%
3D classification (T=4)
23%
29%
19%
29%
Re-extract (1.27 Å/pixel); 3D refinement
3D classification (T=4)
Masks over individual RANBP9-TWA1-WDR26-YPEL5-BRD4BD1 modules
Signal subtraction
28%
25%
29%
18%
Re-extract (1.27 Å/pixel), 3D refinement
32,579 particles
46,003 particles
46,003 particles
14,527 particles
14,527 particles
14,599 particles
14,599 particles
17,560 particles
17,560 particles
Signal subtraction
Join star files
217,957 particles
3D classification (T=4)
6%
12%
11%
71%
3D classification (T=16)
(mask out 1 copy of RANBP9-TWA1)
6%
12%
11%
71%
3D refinement
3D local refinement
(mask over BRDBD1-bound YPEL5-WDR26)
4.2 Å
3D focused classification (T=100, no particle alignment)
(mask over WDR26propellers YPEL5-BRD4BD1)
3.9 Å
69%
21%
7%
3%
3D local refinement
(mask out WDR26 CTLH-CRAN)
3.4 Å
3D focused classification (T=100)
(mask over WDR26propellers YPEL5-BRD4BD1)
22%
30%
48%
3D local refinement
post-processing
(B-factor=- 83 Å2)
3.37 Å
20,764 particles
b
180°
Resolution (Å)
6.8
4.4
3.3
c
Fourier Shell Correlation
FSC=0.143
3.37 Å
1.0
0.8
0.6
0.4
0.2
0.0
0
0.1
0.2
0.3
0.4

Supplementary Figure 1: Determination of the cryo-EM structure of the ZZ1-SO_2H-induced ternary complex

a) Flowchart of the cryo-EM data processing workflow generating the focused-refined map of the ternary complex comprising ZZ1-SO_2H c-Glue, YPEL5-WDR26 E3 receptor module and $BRD4_{BD1}$ neosubstrate. The scale bar in the motion-corrected representative micrograph corresponds to 300 Å.

b) The final post-processed map color-coded to illustrate variations in its local resolution.

c) Gold-standard Fourier shell correlation (FSC) plot. The dotted line represents 0.143 cut-off criterion for estimating nominal resolution.

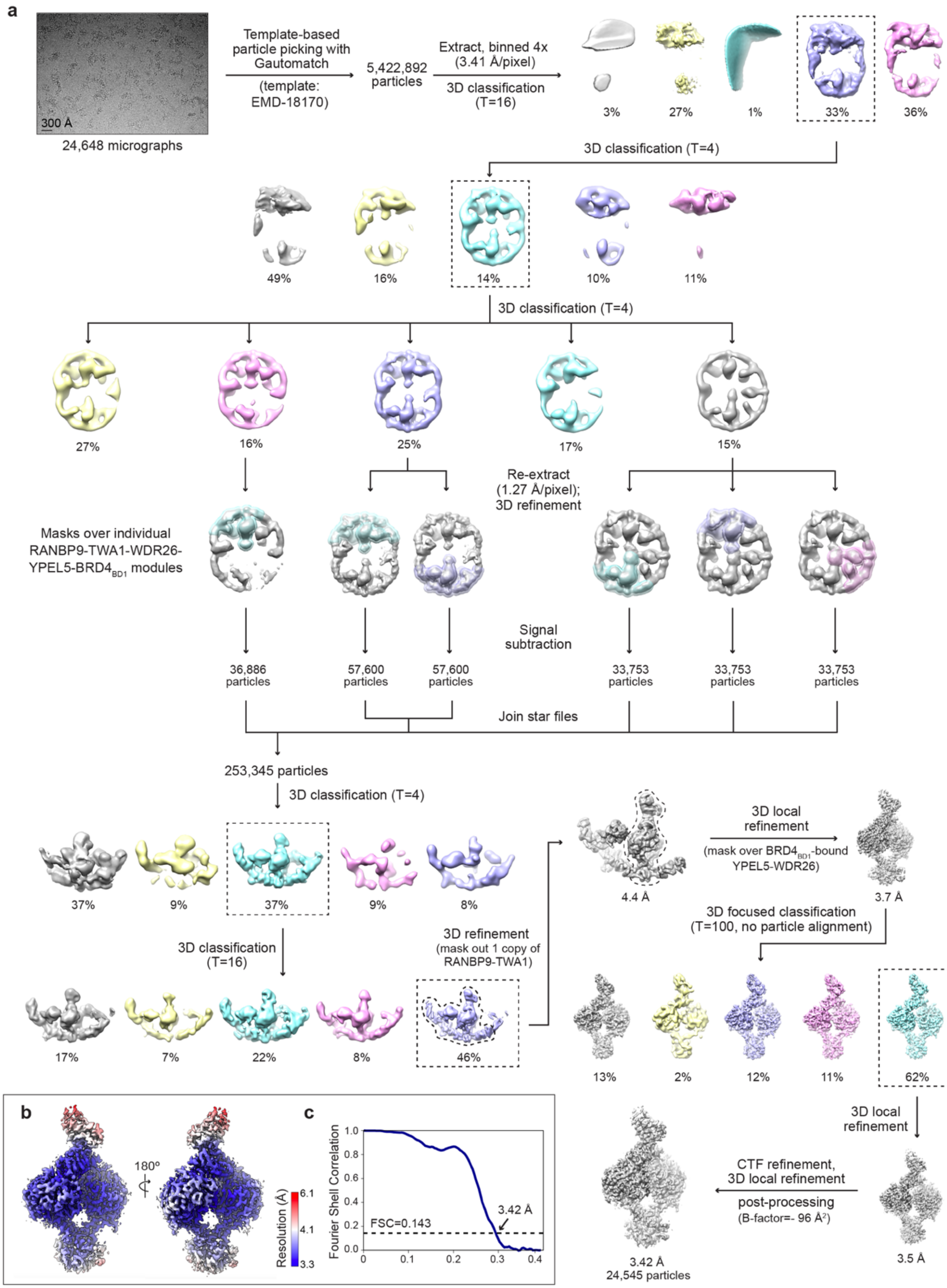
a
300 Å
24,648 micrographs
Template-based particle picking with Gautomatch
(template: EMD-18170)
5,422,892 particles
Extract, binned 4x (3.41 Å/pixel)
3D classification (T=16)
3%
27%
1%
33%
36%
3D classification (T=4)
49%
16%
14%
10%
11%
3D classification (T=4)
27%
16%
25%
17%
15%
Re-extract (1.27 Å/pixel); 3D refinement
Masks over individual RANBP9-TWA1-WDR26-YPEL5-BRD4BD1 modules
Signal subtraction
36,886 particles
57,600 particles
57,600 particles
33,753 particles
33,753 particles
33,753 particles
Join star files
253,345 particles
3D classification (T=4)
37%
9%
37%
9%
8%
3D classification (T=16)
17%
7%
22%
8%
46%
3D refinement (mask out 1 copy of RANBP9-TWA1)
4.4 Å
3D local refinement
(mask over BRD4BD1-bound YPEL5-WDR26)
3.7 Å
3D focused classification (T=100, no particle alignment)
13%
2%
12%
11%
62%
3D local refinement
3.5 Å
CTF refinement, 3D local refinement
post-processing (B-factor=- 96 Å2)
3.42 Å
24,545 particles
b
180°
Resolution (Å)
6.1
4.1
3.3
c
Fourier Shell Correlation
FSC=0.143
3.42 Å
1.0
0.8
0.6
0.4
0.2
0.0
0
0.1
0.2
0.3
0.4

Supplementary Figure 2: Determination of the cryo-EM structure of the ZZ2-SO_2H-induced ternary complex

a) Flowchart of the cryo-EM data processing workflow generating the focused-refined map of the ternary complex comprising the improved ZZ2-SO_2H c-Glue, YPEL5-WDR26 E3 receptor module and $BRD4_{BD1}$ neosubstrate. The scale bar in the motion-corrected representative micrograph corresponds to 300 Å.

b) The final post-processed map color-coded to illustrate variations in its local resolution.

c) Gold-standard Fourier shell correlation (FSC) plot. The dotted line represents 0.143 cut-off criterion for estimating nominal resolution.

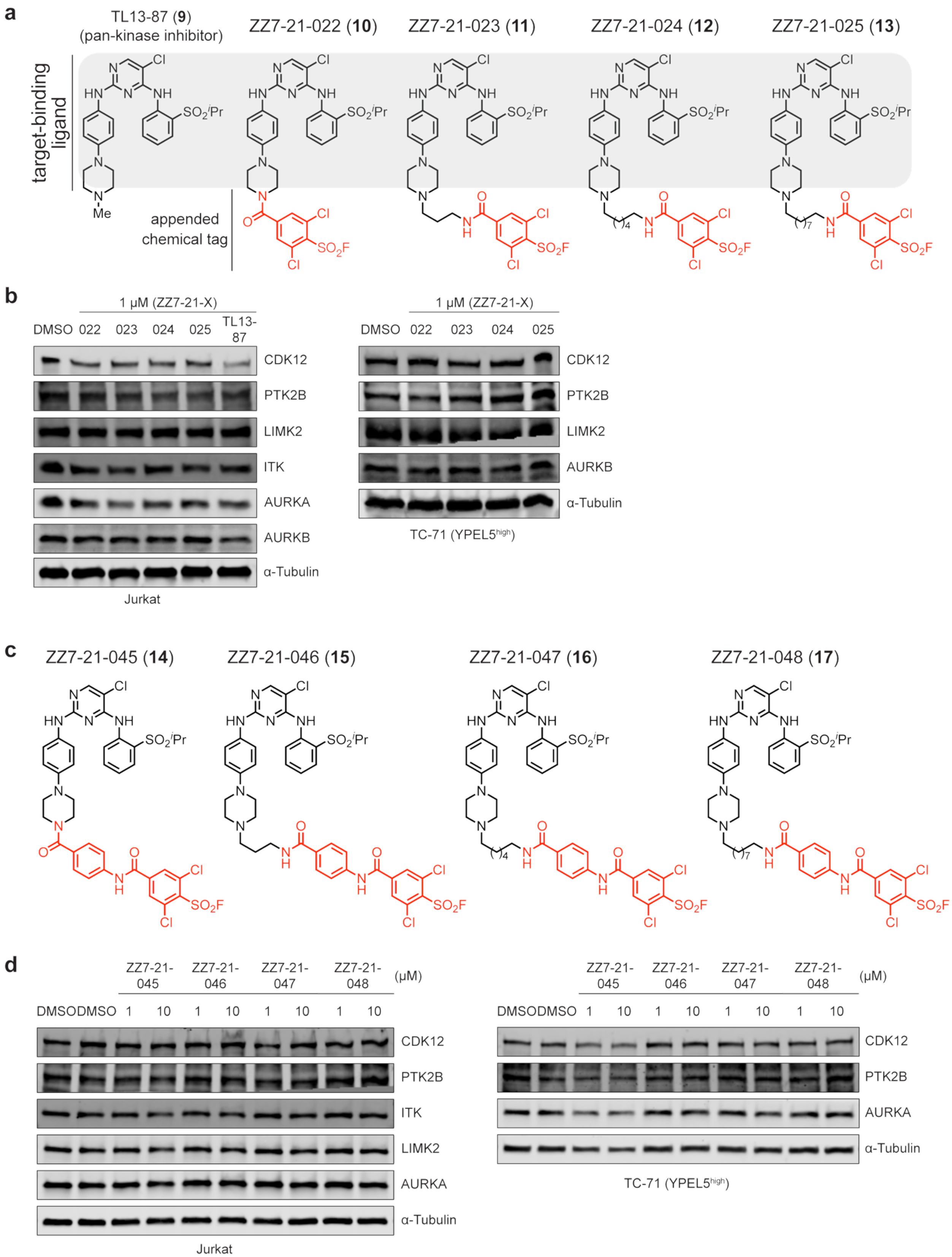

Supplementary Figure 3: Attempts to develop YPEL5-dependent pan-kinase degraders

a) Chemical structures of the pan-kinase inhibitor (TL13-87) and its derivatives featuring an appended sulfonyl fluoride chemical tag with different linker lengths.
b) Western blots results of kinase degradations in Jurkat or TC-71 cells after 3 h treatment with pan-kinase degraders. Western blot experiments were independently repeated at least twice with similar results.
c) Chemical structures of pan-kinase degraders featuring an appended phenyl-linked sulfonyl fluoride chemical tag with different linker lengths.
d) Western blots results of kinase degradations in Jurkat or TC-71 cells after 3 h treatment with pan-kinase degraders. Western blot experiments were independently repeated at least twice with similar results.

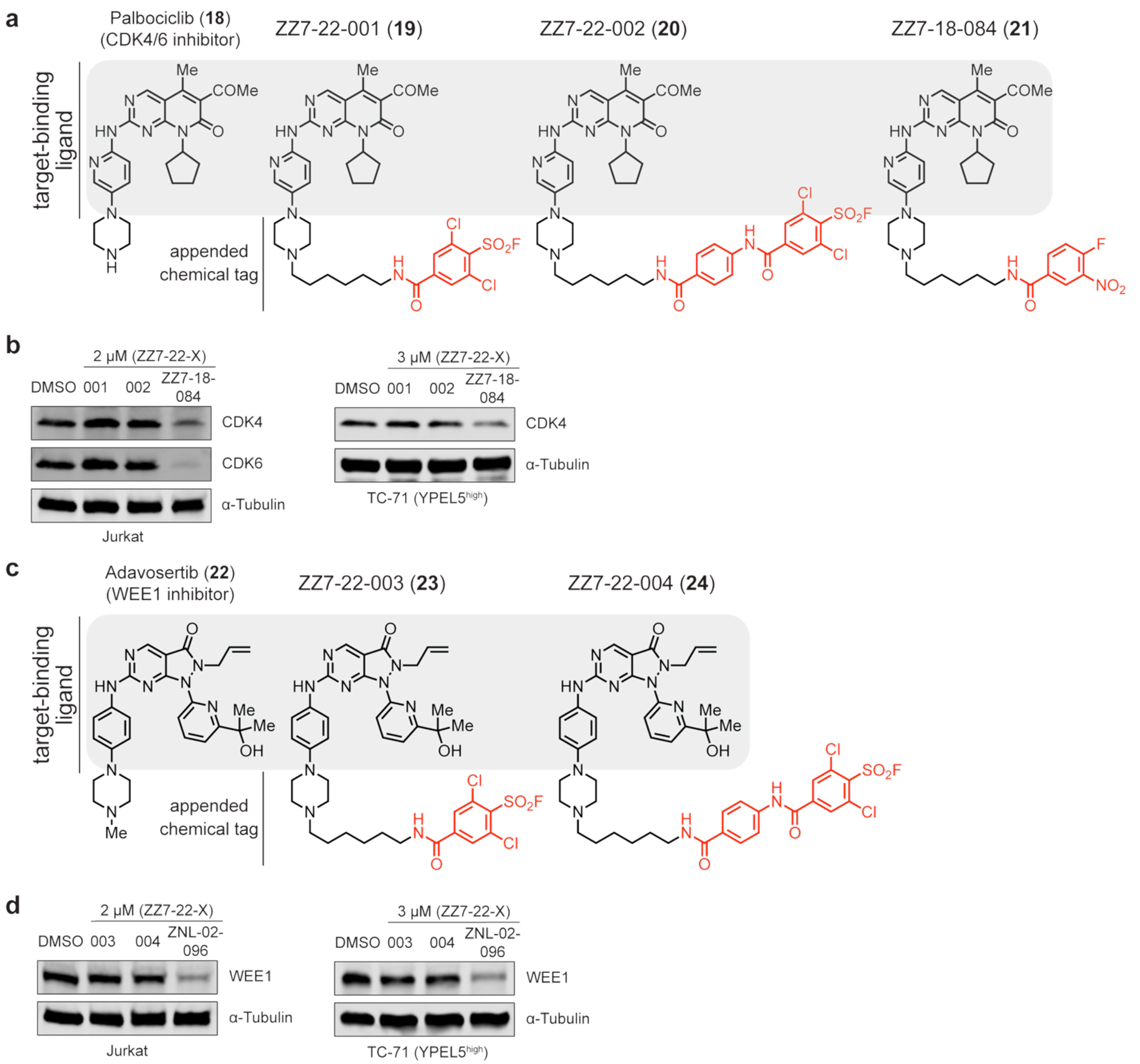

Supplementary Figure 4: Attempts to develop YPEL5-dependent CDK4/6 and WEE1 degraders

a) Chemical structures of the CDK4/6 inhibitor (palbociclib) and its derivatives featuring appended sulfonyl fluoride chemical tags.

b) Western blots results of kinase degradations in Jurkat or TC-71 cells after 5 h treatment with CDK4/6 degraders. ZZ7-18-084 was used as positive control. Western blot experiments were independently repeated at least twice with similar results.

c) Chemical structures of the WEE1 inhibitor (adavosertib) and its derivatives featuring appended sulfonyl fluoride chemical tags. ZNL-02-096 was used as positive control.

d) Western blots results of kinase degradations in Jurkat or TC-71 cells after 5 h treatment with WEE1 degraders. Western blot experiments were independently repeated at least twice with similar results.

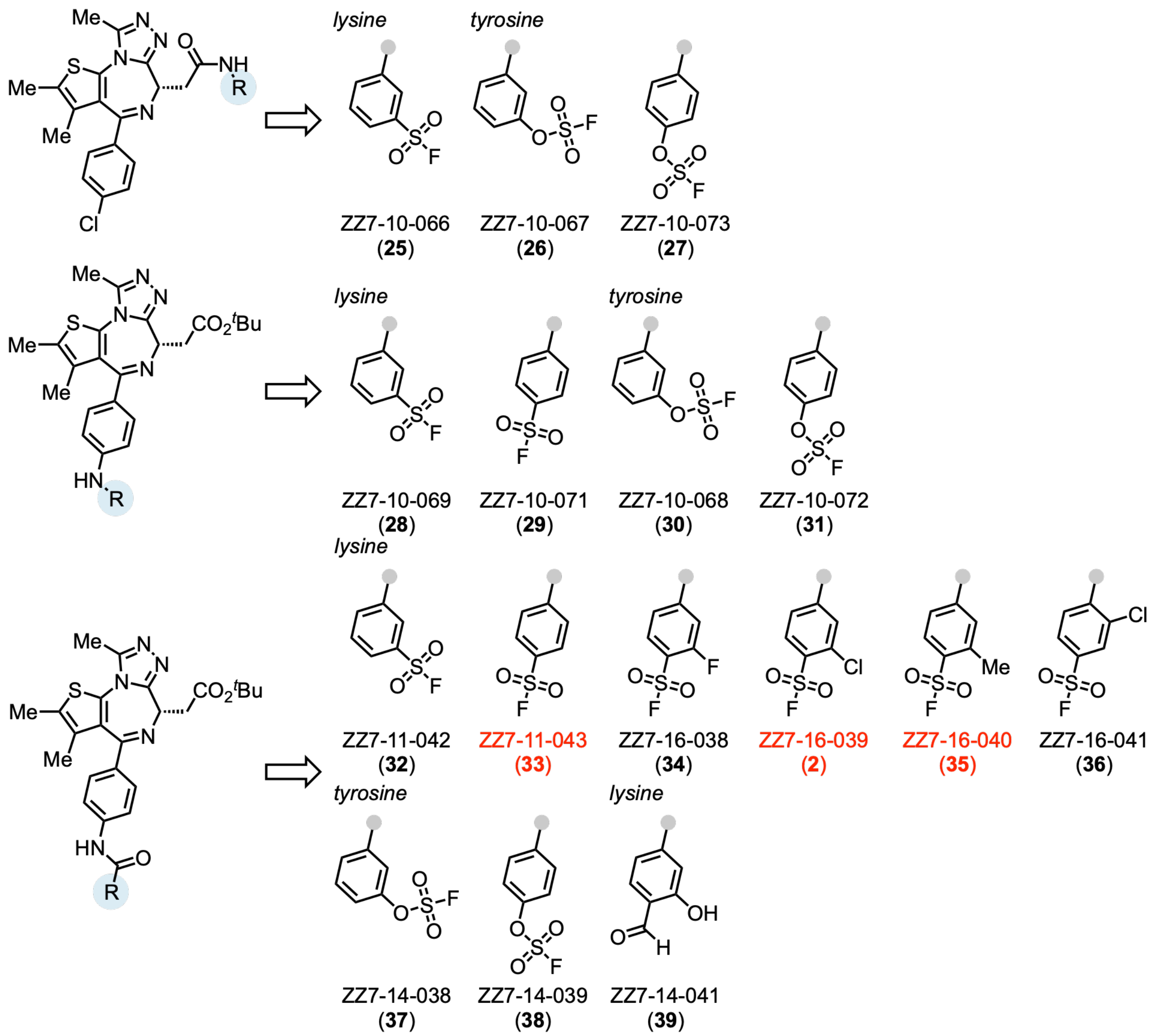

Supplementary Table 1: Construction of a library of electrophilic BRD4 molecular glue degraders through a degron display approach (with hits shown in red).

Supplementary Table 2. Cryo-EM data collection, refinement and validation statistics

	ZZ1-SO_2H-induced assembly of the YPEL5-CTLH E3 ligase and BRD4$_{BD1}$ (EMD-54194)	Ternary complex of ZZ1-SO_2H, BRD$_{BD1}$, and YPEL5-WDR26 (EMD-54216) (PDB 9RSD)	Ternary complex of ZZ2-SO_2H, BRD$_{BD1}$, and YPEL5-WDR26 (EMD-54215) (PDB 9RSC)
**Data collection and processing**			
Magnification	105,000		105,000
Voltage (kV)	300		300
Electron exposure (e–/Å^2)	65.5		63.6
Defocus range (μm)	-(0.8 – 2.3)		-(0.8 – 2.3)
Pixel size (Å)	0.851		0.851
Symmetry imposed	C2	C1	C1
Initial particle images (no.)	893,388		5,422,892
Final particle images (no.)	2,464	20,764	24,545
Map resolution (Å)	11.77	3.38	3.42
FSC threshold	0.143	0.143	0.143
Map resolution range (Å)		3.3-6.8	3.3-6.1
**Refinement**			
Initial model used (PDB code)		8QBN, 3MXF	9GHQ
Map sharpening *B* factor (Å^2)		-83	-96
Model composition			
Non-hydrogen atoms		7450	7573
Protein residues		975	981
Ligands		3 Zn^{2+}, ZZ1-SO_2H	3 Zn^{2+}, ZZ2-SO_2H
B factors (Å^2)			
Protein		44.60	35.69
Ligand		71.65	44.7
R.m.s. deviations			
Bond lengths (Å)		0.00	0.00
Bond angles (°)		0.52	0.55
Validation			
MolProbity score		1.56	1.53
Clashscore		4.88	4.72
Poor rotamers (%)		0.00	0.00
Ramachandran plot			
Favored (%)		95.62	95.85
Allowed (%)		4.38	4.15
Disallowed (%)		0.00	0.00

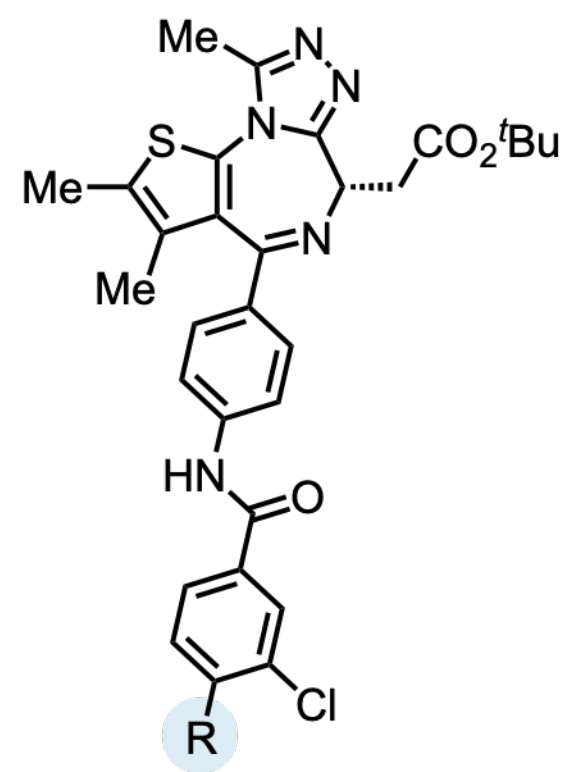

compound	R	HiBiT-BRD4 DC$_{50}$ (nM)	HiBiT-BRD4 D$_{max}$ (%)
ZZ1 (**2**)	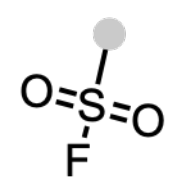	502	82
ZZ7-23-086 (**40**)	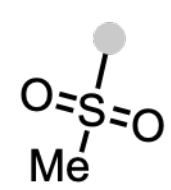	> 10,000	N.A.
ZZ7-23-087 (**41**)	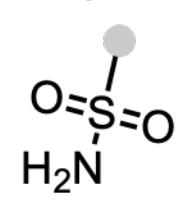	> 10,000	N.A.
ZZ7-23-088 (**42**)[a]	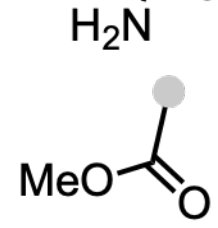	3,660	51
ZZ7-23-089 (**43**)[a]	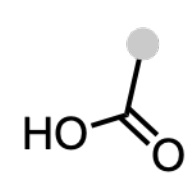	3,820	50
ZZ7-23-090 (**44**)	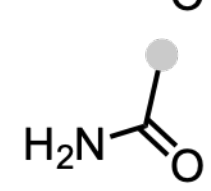	> 10,000	N.A.
ZZ7-23-091 (**45**)	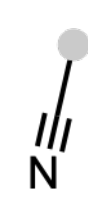	> 10,000	N.A.

[a]YPEL5-independent

Supplementary Table 3: Exploration of sulfinic acid bioisosteres in the BRD4 molecular glue degrader ZZ1. N.A., not applicable.

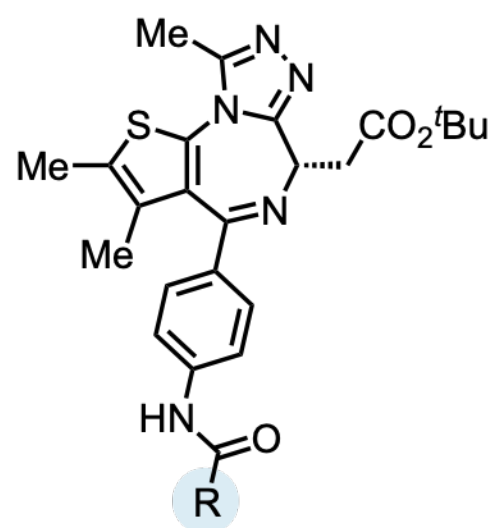

compound	R	HiBiT-BRD4 DC_{50} (nM)	HiBiT-BRD4 D_{max} (%)
ZZ1 (**2**)	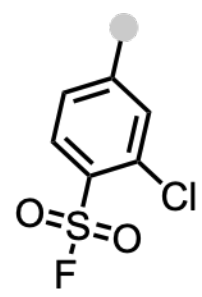	502	82
ZZ7-11-043 (**33**)	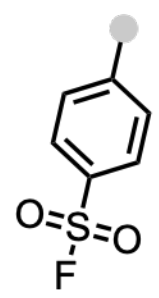	977	75
ZZ7-16-041 (**36**)	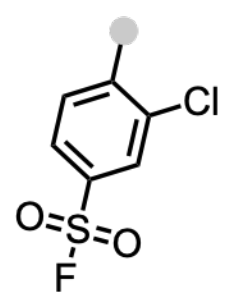	> 10,000	N.A.
ZZ7-16-040 (**35**)	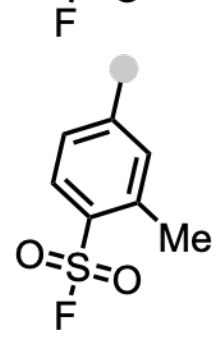	782	67
ZZ7-20-072 (**46**)	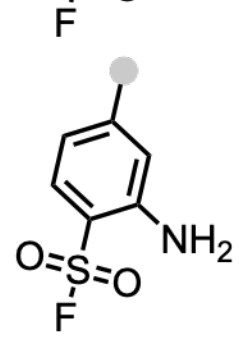	> 10,000	N.A.
ZZ7-16-038 (**34**)	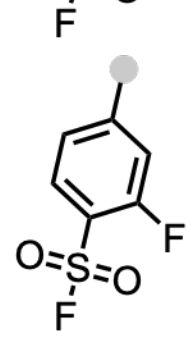	3,790	72
ZZ7-20-090 (**47**)	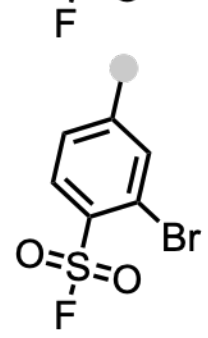	433	77
ZZ7-20-071 (**48**)[a]	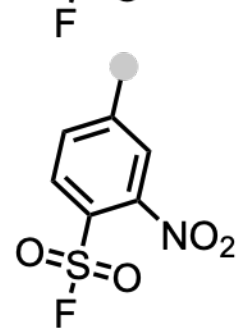	287	59
ZZ2 (**5**)	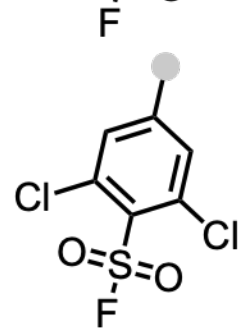	169	90

[a]DCAF16-dependent

Supplementary Table 4: Structure-activity relationship (SAR) of the BRD4 molecular glue degrader ZZ1. N.A., not applicable.

Supplementary Table 5. List of constructs for recombinant protein expression

PLASMID NAME	SOURCE
pBIG1 RANBP9:TWA1-TEV-2xS:RMND5A:MAEA	Chrustowicz, Sherpa et al[39].
pBIG2 RANBP9:TWA1-TEV-2xS:RMND5A:MAEA:ARMC8	Sherpa, Chrustowicz et al[40].
pBIG2 RANBP9:TWA1-TEV-2xS:RMND5A:MAEA:WDR26	This study
pLIB GST-TEV-GSGS-WDR26$^{\Delta CTLH}$ (aa 157-273>GSGS linker)	This study
pLIB GST-TEV-GSGS-WDR26$^{\Delta CTLH}$-Avi-6xHis	This study
pLIB WDR26	Sherpa, Chrustowicz et al[40].
pLIB YPEL5	Sherpa, Chrustowicz et al[40].
pLIB YPEL5 T63D	This study
pLIB YPEL5 K95R	This study
pLIB YPEL5 L62D	This study
pLIB YPEL5 R41A	This study
pLIB YPEL5 K95A, H66L	This study
pLIB YPEL5 K95A,T63A,Y84L	This study
pLIB GST-TEV-UBA1	Qiao et al[51].
pRSF 6xHis-GID4 (aa 100-300)	Sherpa, Chrustowicz et al[40].
pRSF CK2α-6xHis	Chrustowicz, Sherpa et al[39].
pRSF GG-NMNAT1-6xHis	Gottemukkala, Chrustowicz, Sherpa et al[44].
pGEX GST-TEV-UBE2H	Chrustowicz, Sherpa et al[39].
pGEX GST-TEV-GGGSGS-BRD4$_{BD1+BD2}$ (aa 44-460)	This study
pGEX GST-TEV-GGGSGS-BRD4$_{BD1}$ (aa 44-168)	This study
pGEX GST-TEV-GGGSGS-BRD4$_{BD2}$ (aa 333-460)	This study
pGEX GST-TEV-GGGSGS-BRD2$_{BD1}$ (aa 60-184)	This study
pGEX GST-TEV-GGGSGS-BRD2$_{BD2}$ (aa 329-456)	This study
pGEX GST-TEV-GGGSGS-BRD3$_{BD1}$ (aa 20-144)	This study
pGEX GST-TEV-GGGSGS-BRD3$_{BD2}$ (aa 291-418)	This study
pET3b ubiquitin	Kaiser et al[77].
pET29 sortase A-6xHis	Chen et al[78].

General Information.

3-Chloro-4-(fluorosulfonyl)benzoic acid and 3,5-dichloro-4-(fluorosulfonyl)benzoic acid were purchased from Enamine. T_3P was purchased from Combi-Blocks. Other reagents were purchased at the highest commercial quality and used without further purification, unless otherwise stated. Analytical thin layer chromatography was performed on 0.25 mm silica gel 60-F254. Visualization was carried out with short-wave UV light or $KMnO_4$ and heat as developing agents. ^{1}H NMR spectra were recorded on a 500 MHz Bruker Avance III spectrometer. Chemical shifts were quoted in parts per million (ppm) referenced to 2.50 ppm for DMSO-d_6. The following abbreviations (or combinations thereof) were used to explain multiplicities: s = singlet, d = doublet, t = triplet, q = quartet, m = multiplet, br = broad. Coupling constants, *J*, were reported in Hertz unit (Hz). Purification was carried out by flash column chromatography using CombiFlash®Rf with Teledyne Isco RediSep® normal-phase silica flash columns or preparative RP-HPLC using Waters SunFireTM Prep C18 column (19 x 100 mm, 5 μm particle size). Liquid chromatography–mass spectrometry (LC–MS) was recorded on a Waters ACQUITY UPLC/MS system using ESI-TOF (electrospray ionization-time of flight).

General Procedure of preparing c-Glues.

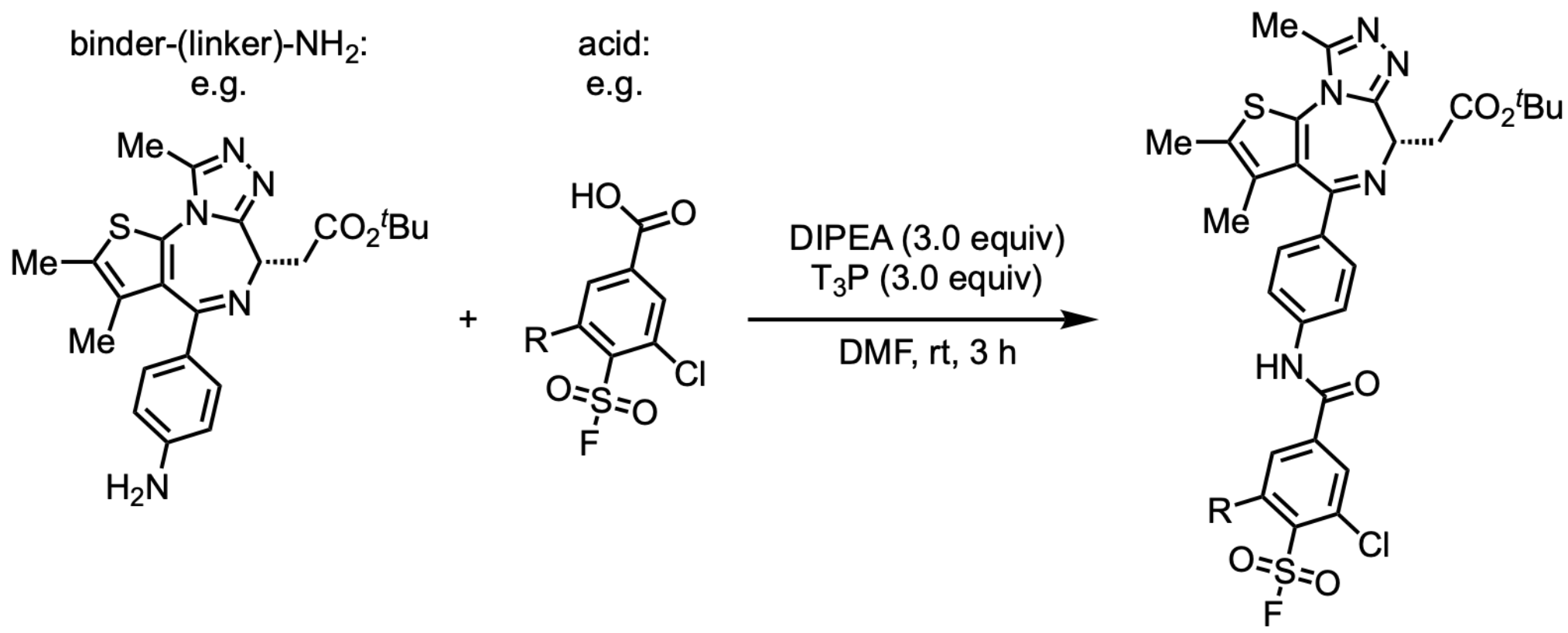

Into a culture tube, binder-(linker)-NH_2 (0.01 mmol) was weighed in air and a magnetic stir bar was added. Then the corresponding acid (3.0 equiv), DIPEA (3.0 equiv, 5 µL), DMF (0.5 mL) and the 1-propanephosphonic anhydride 50% in DMF (T_3P) (3.0 equiv) were added in this order. The reaction mixture was stirred at rt for 3 hours (600 rpm). The crude mixture was purified by preparative RP-HPLC to afford the desired product c-Glue (e.g.: ZZ1 (2) or ZZ2 (5)).

Preparation of c-Glues.

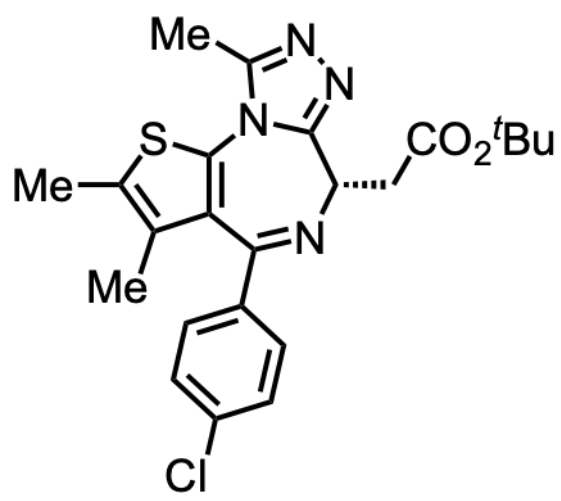

***tert*-butyl (*S*)-2-(4-(4-chlorophenyl)-2,3,9-trimethyl-6*H*-thieno[3,2-*f*][1,2,4]triazolo[4,3-*a*][1,4]diazepin-6-yl)acetate (JQ1; 1)**

JQ1 is a commercially available BET inhibitor.

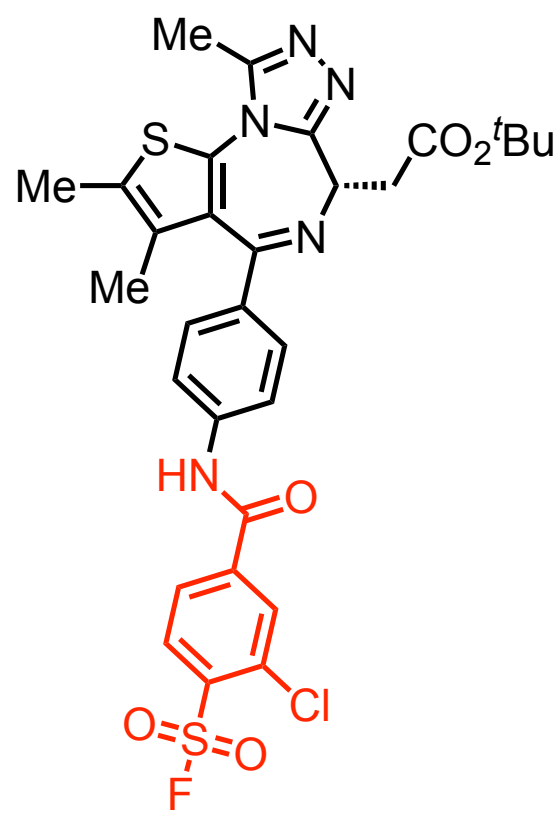

***tert*-butyl (*S*)-2-(4-(4-(3-chloro-4-(fluorosulfonyl)benzamido)phenyl)-2,3,9-trimethyl-6*H*-thieno[3,2-*f*][1,2,4]triazolo[4,3-*a*][1,4]diazepin-6-yl)acetate (ZZ7-16-039; ZZ1; 2)**

Following General Procedure using JQ1-NH_2[1], purification by preparative RP-HPLC afforded the title compound (2.60 mg, 40% yield).

^{1}H NMR (500 MHz, DMSO-d_6) δ 10.85 (s, 1H), 8.42 (s, 1H), 8.38 (d, *J* = 8.3 Hz, 1H), 8.18 (d, *J* = 8.3 Hz, 1H), 7.84 (d, *J* = 8.4 Hz, 2H), 7.46 (d, *J* = 8.4 Hz, 2H), 4.47 – 4.36 (m, 1H), 3.40 – 3.26 (m, 2H), 2.61 (s, 3H), 2.43 (s, 3H), 1.67 (s, 3H), 1.43 (s, 9H).

^{13}C NMR (125 MHz, DMSO) δ 169.8, 163.5, 162.7, 155.0, 149.9, 142.8, 140.6, 133.5, 132.4, 132.2, 131.9, 131.9, 131.6, 130.8, 130.1, 130.0, 129.1, 127.9, 119.9, 80.2, 53.5, 37.5, 27.8, 14.0, 12.7, 11.3.

LC–MS (ESI-TOF) Calcd for $C_{30}H_{30}ClFN_5O_5S_2^+$ $[M+H]^+$: 658.14; found: 658.36.

HRMS (ESI-TOF) Calcd for $C_{30}H_{30}ClFN_5O_5S_2^+$ $[M+H]^+$: 658.1356; found: 658.1348.

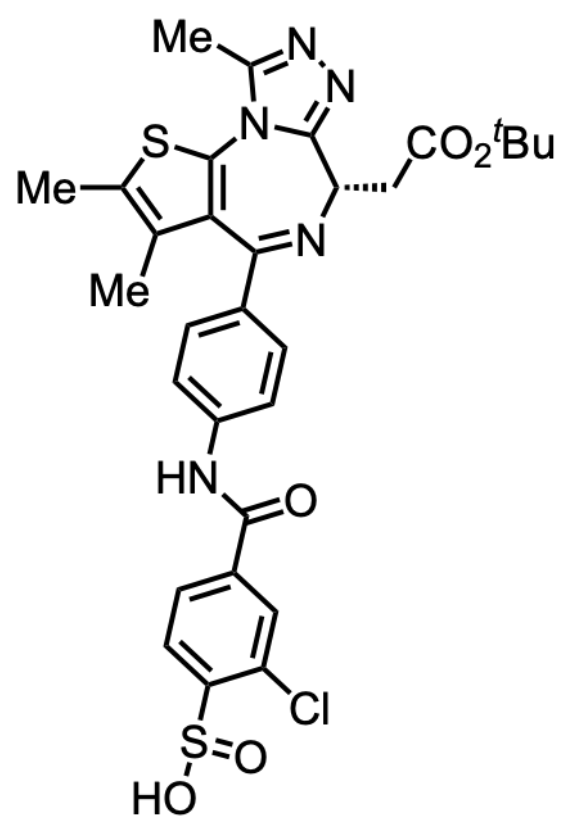

4-((4-((*S*)-6-(2-(*tert*-butoxy)-2-oxoethyl)-2,3,9-trimethyl-6*H*-thieno[3,2-*f*][1,2,4]triazolo[4,3-*a*][1,4]diazepin-4-yl)phenyl)carbamoyl)-2-chlorobenzenesulfinic acid (ZZ7-21-004; ZZ1-SO_2H; 3)

Following literature procedure[2] with slight modification: ZZ1 (0.005 mmol), Ac-Cys-OMe (3.0 equiv), DIPEA (3.0 equiv), DMF (1.0 mL), rt, 6 h. Purification by preparative RP-HPLC afforded the title compound (0.50 mg, 16% yield).

^{1}H NMR (500 MHz, DMSO-d_6) δ 10.66 (s, 1H), 8.30 (br s, 1H), 8.13 – 8.09 (m, 1H), 7.99 – 7.93 (m, 2H), 7.84 (d, *J* = 8.8 Hz, 2H), 7.44 (d, *J* = 8.3 Hz, 2H), 4.42 (dd, *J* = 8.1, 6.3 Hz, 1H), 3.36 – 3.29 (m, 2H), 2.89 (s, 3H), 2.73 (s, 3H), 1.67 (s, 3H), 1.43 (s, 9H).

LC–MS (ESI-TOF) Calcd for $C_{30}H_{31}ClN_5O_5S_2^+$ [M+H]$^+$: 640.15; found: 640.26.

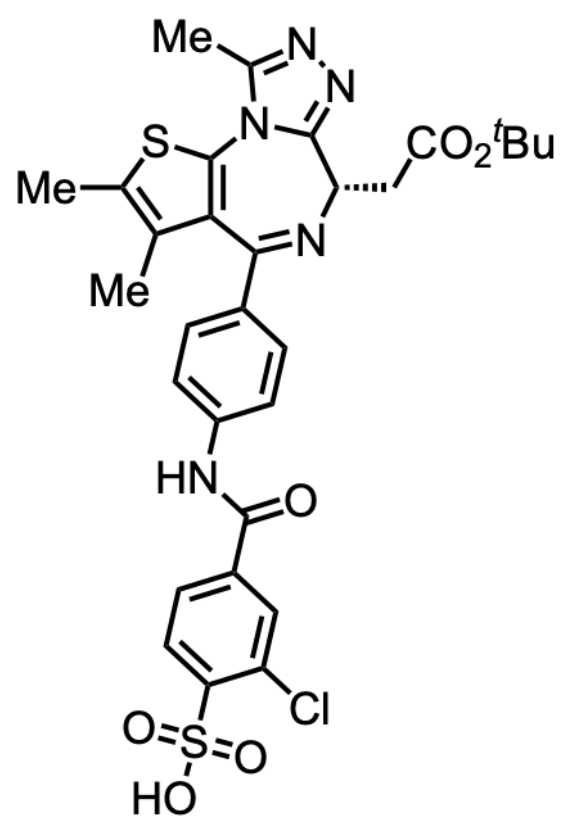

(*S*)-4-((4-(6-(2-(*tert*-butoxy)-2-oxoethyl)-2,3,9-trimethyl-6*H*-thieno[3,2-*f*][1,2,4]triazolo[4,3-*a*][1,4]diazepin-4-yl)phenyl)carbamoyl)-2-chlorobenzenesulfonic acid (ZZ7-21-003; ZZ1-SO_3H; 4)

Following literature procedure[3] with slight modification: ZZ1 (0.005 mmol), NaOH (2.0 M, 3.0 equiv), THF (1.0 mL), rt, 1 h. Purification by preparative RP-HPLC afforded the title compound (2.95 mg, 90% yield).

^{1}H NMR (500 MHz, DMSO-d_6) δ 10.55 (s, 1H), 8.07 – 7.93 (d, J = 23.6 Hz, 2H), 7.93 – 7.79 (m, 3H), 7.54 – 7.39 (m, 2H), 4.51 – 4.40 (m, 1H), 3.38 – 3.31 (m, 2H), 2.62 (s, 3H), 2.43 (s, 3H), 1.67 (s, 3H), 1.43 (s, 9H).

LC–MS (ESI-TOF) Calcd for $C_{30}H_{31}ClN_5O_6S_2^+$ [M+H]$^+$: 656.14; found: 656.21.

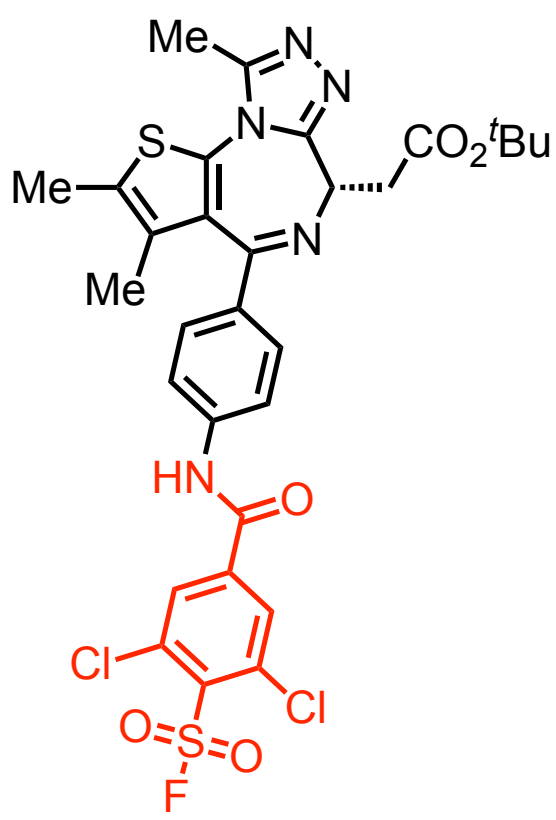

***tert*-butyl (*S*)-2-(4-(4-(3,5-dichloro-4-(fluorosulfonyl)benzamido)phenyl)-2,3,9-trimethyl-6*H*-thieno[3,2-*f*][1,2,4]triazolo[4,3-*a*][1,4]diazepin-6-yl)acetate (ZZ7-20-070; ZZ2; 5)**

Following General Procedure using JQ1-NH_2, purification by preparative RP-HPLC afforded the title compound (3.30 mg, 48% yield).

^{1}H NMR (500 MHz, DMSO-d_6) δ 10.87 (s, 1H), 8.33 (s, 2H), 7.82 (d, *J* = 8.7 Hz, 2H), 7.46 (d, *J* = 8.7 Hz, 2H), 4.45 – 4.39 (m, 1H), 3.40 – 3.25 (m, 2H), 2.61 (s, 3H), 2.43 (s, 3H), 1.67 (s, 3H), 1.43 (s, 9H).

^{13}C NMR (125 MHz, DMSO) δ 169.8, 163.5, 161.4, 155.0, 149.9, 141.5, 140.4, 134.8, 133.7, 131.9, 131.0, 130.8, 130.1, 130.0, 129.1, 119.9, 80.2, 53.5, 37.5, 27.8, 14.0, 12.7, 11.3.

LC–MS (ESI-TOF) Calcd for $C_{30}H_{29}Cl_2FN_5O_5S_2^+$ $[M+H]^+$: 692.10; found: 692.42.

HRMS (ESI-TOF) Calcd for $C_{30}H_{30}ClFN_5O_5S_2^+$ $[M+H]^+$: 692.0966; found: 692.0958.

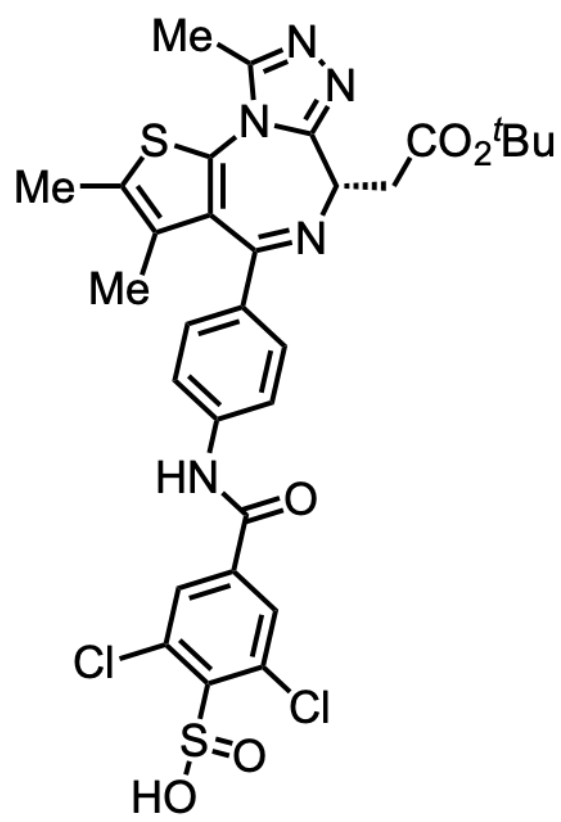

4-((4-((*S*)-6-(2-(*tert*-butoxy)-2-oxoethyl)-2,3,9-trimethyl-6*H*-thieno[3,2-*f*][1,2,4]triazolo[4,3-*a*][1,4]diazepin-4-yl)phenyl)carbamoyl)-2,6-dichlorobenzenesulfinic acid (ZZ2-SO_2H; 6)

ZZ2-SO_2H was generated by pre-incubation of ZZ2 in the DTT-containing buffer: DMSO stock of ZZ2 was incubated with buffer B (containing 5 mM DTT) for 1 h at room temperature and analyzed by intact mass spectrometry along with their DMSO-diluted controls.

LC–MS (ESI-TOF) Calcd for $C_{30}H_{30}Cl_2N_5O_5S_2^+$ $[M+H]^+$: 674; found: 674.

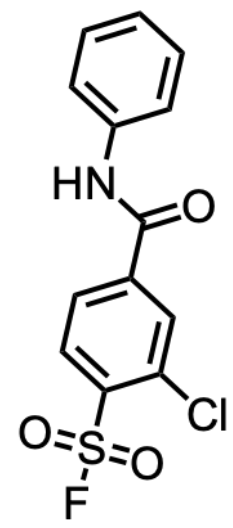

2-chloro-4-(phenylcarbamoyl)benzenesulfonyl fluoride (ZZ7-21-029; 7)

Following General Procedure, purification by preparative RP-HPLC afforded the title compound (1.10 mg, 35% yield).

^{1}H NMR (500 MHz, DMSO-d_6) δ 10.65 (s, 1H), 8.46 – 8.33 (m, 2H), 8.19 (d, *J* = 8.2 Hz, 1H), 7.83 – 7.70 (m, 2H), 7.44 – 7.33 (m, 2H), 7.20 – 7.08 (m, 1H).

LC–MS (ESI-TOF) Calcd for $C_{13}H_{10}ClFNO_3S^+$ $[M+H]^+$: 314.00; found: 314.20.

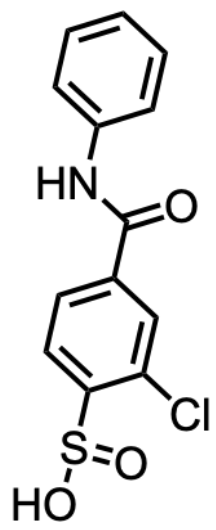

2-chloro-4-(phenylcarbamoyl)benzenesulfinic acid (8)

The sulfinic acid version of the YPEL5-engaging ZZ1-SO_2H fragment 8 was generated by pre-incubation of 7 (sulfonyl fluoride form) in the DTT-containing buffer: DMSO stock of 7 was incubated with buffer B (containing 5 mM DTT) overnight at room temperature and analyzed by intact mass spectrometry along with their DMSO-diluted controls.

LC–MS (ESI-TOF) Calcd for $C_{13}H_{11}ClNO_3S^+$ $[M+H]^+$: 296 and 298; found: 298.

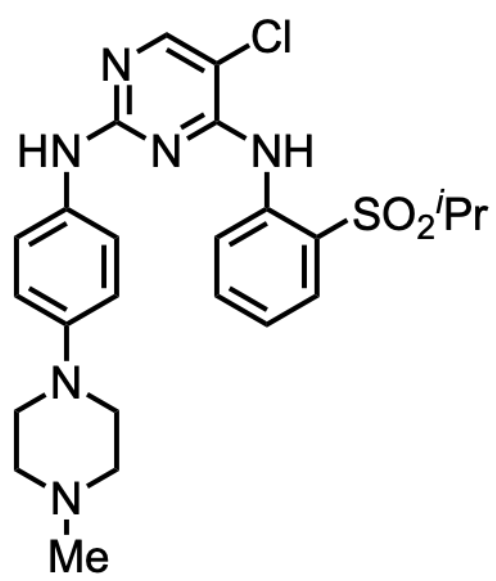

5-chloro-*N*^{4}-(2-(isopropylsulfonyl)phenyl)-*N*^{2}-(4-(4-methylpiperazin-1-yl)phenyl)pyrimidine-2,4-diamine (TL13-87; 9)

TL13-87 is a reported pan-kinase inhibitor.

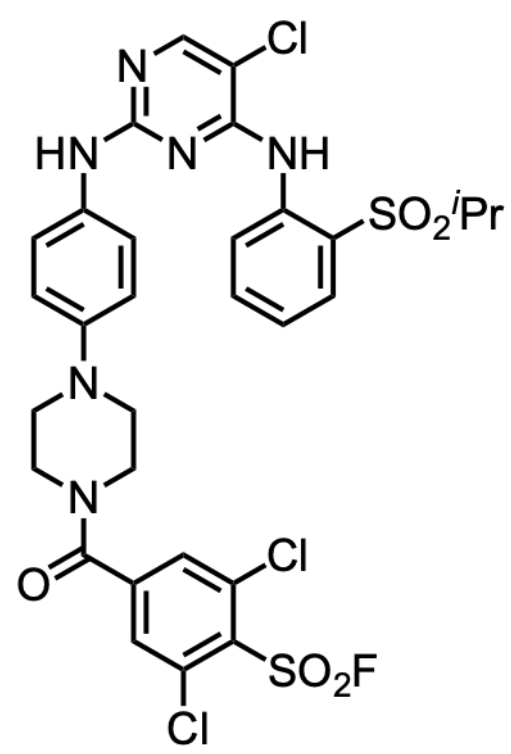

2,6-dichloro-4-(4-(4-((5-chloro-4-((2-(isopropylsulfonyl)phenyl)amino)pyrimidin-2-yl)amino)phenyl)piperazine-1-carbonyl)benzenesulfonyl fluoride (ZZ7-21-022; 10)

Following General Procedure using TL13-87-NH_2[4], purification by preparative RP-HPLC afforded the title compound (4.92 mg, 66% yield).

^{1}H NMR (500 MHz, DMSO-d_6) δ 9.51 (s, 1H), 9.40 (s, 1H), 8.61 (br s, 1H), 8.25 (s, 1H), 7.99 (s, 2H), 7.84 (dd, J = 7.9, 1.6 Hz, 1H), 7.78 – 7.71 (m, 1H), 7.46 (d, J = 8.4 Hz, 2H), 7.41 – 7.34 (m, 1H), 6.92 (d, J = 9.0 Hz, 2H), 3.78 (t, J = 5.0 Hz, 2H), 3.46 – 3.43 (m, 3H), 3.21 (t, J = 5.2 Hz, 2H), 3.08 (t, J = 5.1 Hz, 2H), 1.16 (d, J = 6.8 Hz, 6H).

LC–MS (ESI-TOF) Calcd for $C_{30}H_{29}Cl_3FN_6O_5S_2^+$ $[M+H]^+$: 741.07; found: 741.15.

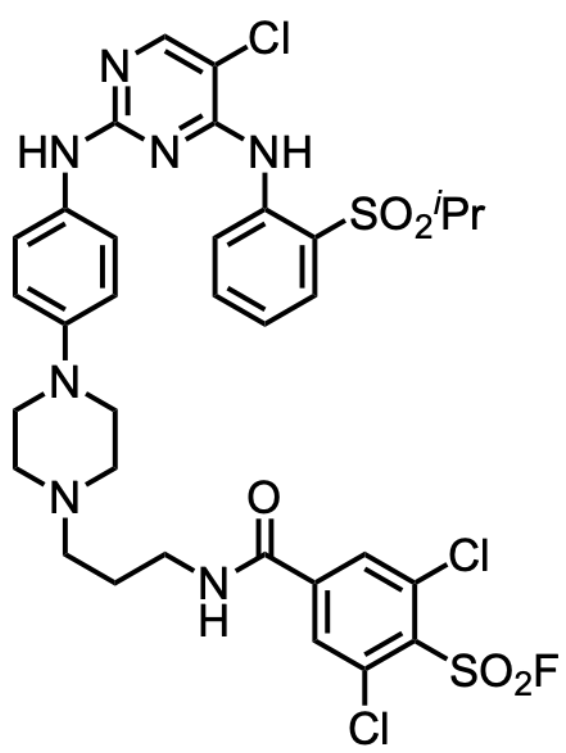

2,6-dichloro-4-((3-(4-(4-((5-chloro-4-((2-(isopropylsulfonyl)phenyl)amino)pyrimidin-2-yl)amino)phenyl)piperazin-1-yl)propyl)carbamoyl)benzenesulfonyl fluoride (ZZ7-21-023; 11)

Following General Procedure TL13-87-NH_2, purification by preparative RP-HPLC afforded the title compound (1.43 mg, 18% yield).

^{1}H NMR (500 MHz, DMSO-d_6) δ 9.49 (s, 1H), 9.38 (s, 1H), 9.17 (br s, 1H), 8.64 (br s, 1H), 8.24 (s, 1H), 8.21 – 8.12 (m, 2H), 7.84 (d, *J* = 7.7 Hz, 1H), 7.74 (s, 1H), 7.48 (d, *J* = 8.0 Hz, 2H), 7.41 – 7.32 (m, 1H), 6.94 (s, 2H), 3.78 – 3.71 (m, 2H), 3.10 – 2.93 (m, 8H), 2.83 – 2.79 (m, 3H), 1.65 – 1.57 (m, 2H), 1.16 (d, *J* = 6.8 Hz, 6H).

LC–MS (ESI-TOF) Calcd for $C_{33}H_{36}Cl_3FN_7O_5S_2^+$ $[M+H]^+$: 798.13; found: 798.20.

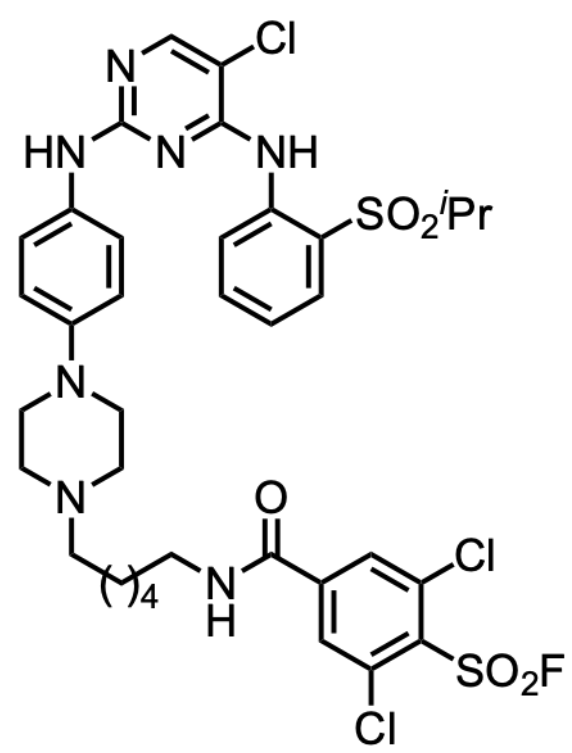

2,6-dichloro-4-((6-(4-(4-((5-chloro-4-((2-(isopropylsulfonyl)phenyl)amino)pyrimidin-2-yl)amino)phenyl)piperazin-1-yl)hexyl)carbamoyl)benzenesulfonyl fluoride (ZZ7-21-024; 12)

Following General Procedure TL13-87-NH_2, purification by preparative RP-HPLC afforded the title compound (1.60 mg, 19% yield).

^{1}H NMR (500 MHz, DMSO-d_6) δ 9.50 (s, 1H), 9.39 (s, 1H), 8.99 (t, J = 5.6 Hz, 1H), 8.64 (br s, 1H), 8.25 (s, 1H), 8.20 (s, 2H), 7.85 (dd, J = 7.9, 1.6 Hz, 1H), 7.78 – 7.70 (m, 1H), 7.48 (d, J = 8.4 Hz, 2H), 7.37 (t, J = 7.6 Hz, 1H), 6.94 (d, J = 9.1 Hz, 2H), 3.74 (d, J = 12.9 Hz, 2H), 3.47 – 3.43 (m, 3H), 3.30 (q, J = 6.7 Hz, 2H), 3.20 – 3.10 (m, 4H), 2.93 (t, J = 12.2 Hz, 2H), 1.74 – 1.63 (m, 2H), 1.59 – 1.46 (m, 3H), 1.38 – 1.33 (m, 3H), 1.17 (d, J = 6.8 Hz, 6H).

LC–MS (ESI-TOF) Calcd for $C_{36}H_{42}Cl_3FN_7O_5S_2^+$ $[M+H]^+$: 840.17; found: 840.15.

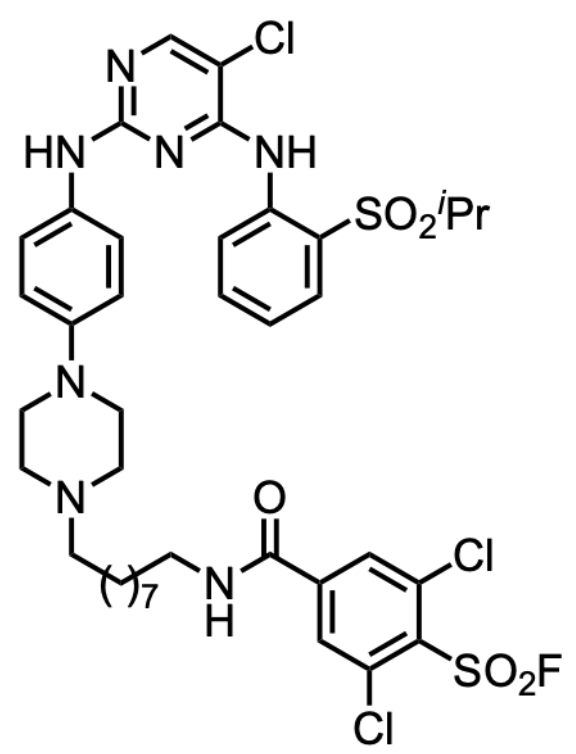

2,6-dichloro-4-((9-(4-(4-((5-chloro-4-((2-(isopropylsulfonyl)phenyl)amino)pyrimidin-2-yl)amino)phenyl)piperazin-1-yl)nonyl)carbamoyl)benzenesulfonyl fluoride (ZZ7-21-025; 13)

Following General Procedure TL13-87-NH_2, purification by preparative RP-HPLC afforded the title compound (0.97 mg, 11% yield).

^{1}H NMR (500 MHz, DMSO) δ 9.49 (s, 1H), 9.37 (s, 1H), 8.97 (s, 1H), 8.64 (br s, 1H), 8.24 (s, 1H), 8.21 – 8.10 (m, 2H), 7.84 (d, *J* = 7.7 Hz, 1H), 7.80 – 7.68 (m, 1H), 7.48 (d, *J* = 8.2 Hz, 2H), 7.43 – 7.32 (m, 1H), 6.93 (d, *J* = 8.2 Hz, 2H), 3.79 – 3.72 (m, 2H), 3.37 – 3.20 (m, 9H), 3.15 – 3.05 (m, 2H), 1.70 – 1.63 (m, 2H), 1.57 – 1.42 (m, 8H), 1.37 – 1.26 (m, 4H), 1.16 (d, *J* = 6.8 Hz, 6H).

LC–MS (ESI-TOF) Calcd for $C_{39}H_{48}Cl_3FN_7O_5S_2^+$ $[M+H]^+$: 882.22; found: 882.21.

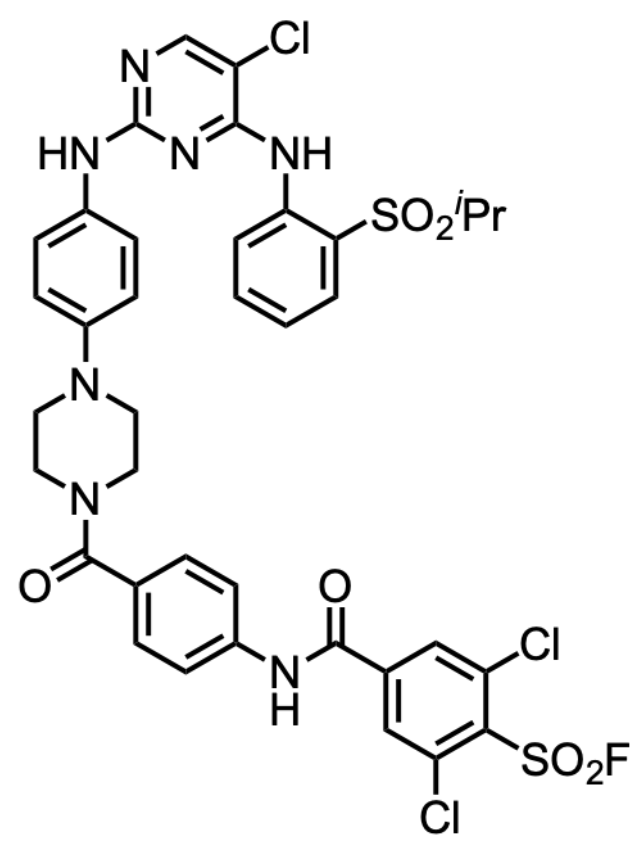

2,6-dichloro-4-((4-(4-(4-((5-chloro-4-((2-(isopropylsulfonyl)phenyl)amino)pyrimidin-2-yl)amino)phenyl)piperazine-1-carbonyl)phenyl)carbamoyl)benzenesulfonyl fluoride (ZZ7-21-045; 14)

Following General Procedure TL13-87-NH_2, purification by preparative RP-HPLC afforded the title compound (1.08 mg, 13% yield).

^{1}H NMR (500 MHz, DMSO-d_6) δ 10.86 (s, 1H), 9.49 (s, 1H), 9.37 (d, J = 13.6 Hz, 1H), 8.79 (br s, 1H), 8.64 (br s, 1H), 8.34 (s, 1H), 8.24 (d, J = 2.8 Hz, 1H), 7.88 – 7.80 (m, 3H), 7.75 (t, J = 8.1 Hz, 1H), 7.55 – 7.42 (m, 4H), 7.37 (t, J = 7.7 Hz, 1H), 6.97 – 6.86 (m, 2H), 3.48 – 3.40 (m, 1H), 3.34 – 3.20 (m, 4H), 3.19 – 3.07 (m, 4H), 1.16 (d, J = 6.7 Hz, 6H).

LC–MS (ESI-TOF) Calcd for $C_{37}H_{34}Cl_3FN_7O_6S_2^+$ $[M+H]^+$: 860.11; found: 860.10.

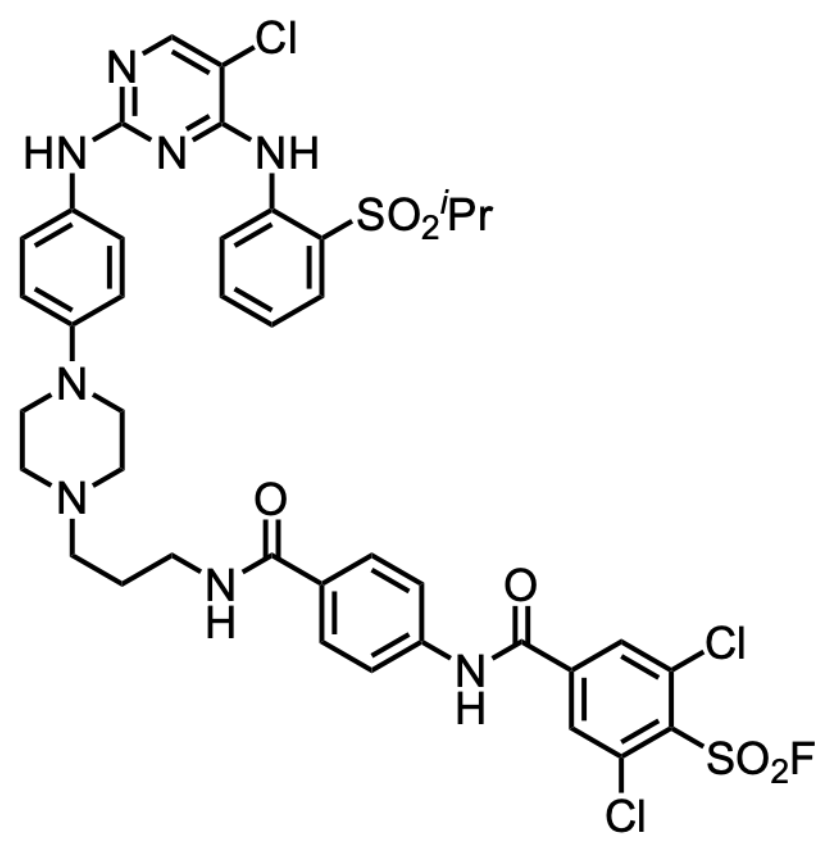

2,6-dichloro-4-((4-((3-(4-(4-((5-chloro-4-((2-(isopropylsulfonyl)phenyl)amino)pyrimidin-2-yl)amino)phenyl)piperazin-1-yl)propyl)carbamoyl)phenyl)carbamoyl)benzenesulfonyl fluoride (ZZ7-21-046; 15)

Following General Procedure TL13-87-NH_2, purification by preparative RP-HPLC afforded the title compound (1.17 mg, 13% yield).

^{1}H NMR (500 MHz, DMSO-d_6) δ 10.90 (br s, 1H), 9.68 (s, 1H), 9.50 (s, 1H), 9.38 (s, 1H), 8.64 (t, *J* = 5.8 Hz, 2H), 8.33 (s, 2H), 8.24 (s, 1H), 7.94 – 7.83 (m, 3H), 7.74 (t, *J* = 7.9 Hz, 1H), 7.48 (d, *J* = 8.4 Hz, 2H), 7.37 (t, *J* = 7.6 Hz, 2H), 6.94 (d, *J* = 9.0 Hz, 2H), 3.76 (d, *J* = 12.5 Hz, 2H), 3.61 (d, *J* = 11.3 Hz, 2H), 3.26 – 3.13 (m, 6H), 3.00 – 2.89 (m, 3H), 1.97 (t, *J* = 8.1 Hz, 2H), 1.16 (d, *J* = 6.8 Hz, 6H).

LC–MS (ESI-TOF) Calcd for $C_{40}H_{41}Cl_3FN_8O_6S_2^+$ $[M+H]^+$: 917.16; found: 917.11.

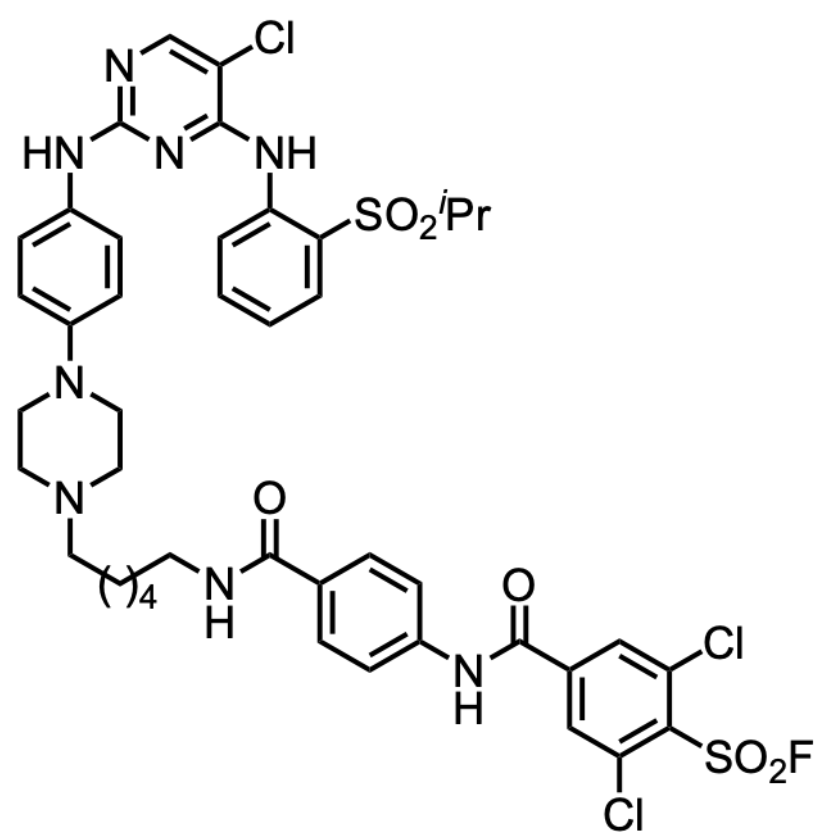

2,6-dichloro-4-((4-((6-(4-(4-((5-chloro-4-((2-(isopropylsulfonyl)phenyl)amino)pyrimidin-2-yl)amino)phenyl)piperazin-1-yl)hexyl)carbamoyl)phenyl)carbamoyl)benzenesulfonyl fluoride (ZZ7-21-047; 16)

Following General Procedure TL13-87-NH_2, purification by preparative RP-HPLC afforded the title compound (0.97 mg, 10% yield).

^{1}H NMR (500 MHz, DMSO-d_6) δ 10.87 (s, 1H), 9.55 (br s, 1H), 9.50 (s, 1H), 9.38 (s, 1H), 8.63 (br s, 1H), 8.45 (t, *J* = 5.6 Hz, 1H), 8.33 (s, 2H), 8.24 (s, 1H), 7.90 – 7.82 (m, 4H), 7.74 (t, *J* = 7.9 Hz, 1H), 7.48 (d, *J* = 8.4 Hz, 2H), 7.37 (t, *J* = 7.7 Hz, 1H), 6.93 (d, *J* = 8.9 Hz, 2H), 3.74 (d, *J* = 13.0 Hz, 2H), 3.58 (d, *J* = 11.9 Hz, 2H), 3.29 – 3.25 (m, 3H), 3.17 – 3.12 (m, 3H), 2.92 (t, *J* = 12.4 Hz, 3H), 1.74 – 1.63 (m, 2H), 1.62 – 1.50 (m, 2H), 1.40 – 1.33 (m, 4H), 1.16 (d, *J* = 6.8 Hz, 6H).

LC–MS (ESI-TOF) Calcd for $C_{43}H_{47}Cl_3FN_8O_6S_2^+$ $[M+H]^+$: 959.21; found: 959.31.

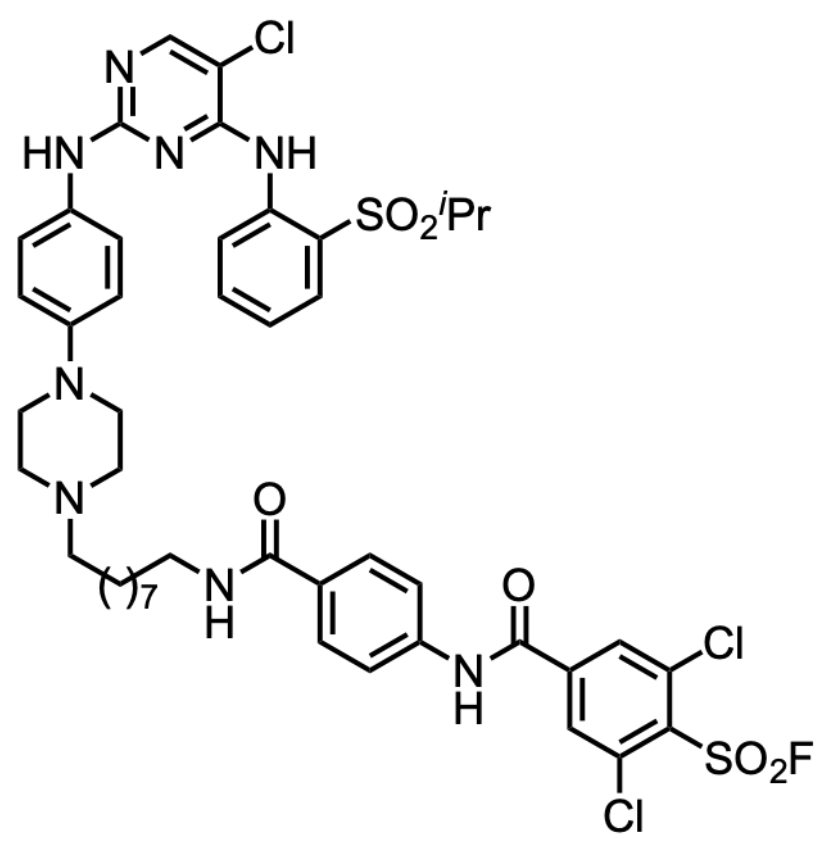

2,6-dichloro-4-((4-((9-(4-(4-((5-chloro-4-((2-(isopropylsulfonyl)phenyl)amino)pyrimidin-2-yl)amino)phenyl)piperazin-1-yl)nonyl)carbamoyl)phenyl)carbamoyl)benzenesulfonyl fluoride (ZZ7-21-048; 17)

Following General Procedure TL13-87-NH_2, purification by preparative RP-HPLC afforded the title compound (0.62 mg, 6% yield).

^{1}H NMR (500 MHz, DMSO-d_6) δ 10.86 (d, J = 3.3 Hz, 1H), 9.50 (s, 1H), 9.38 (s, 1H), 8.63 (br s, 1H), 8.42 (t, J = 5.6 Hz, 1H), 8.33 (s, 2H), 8.24 (s, 1H), 7.90 – 7.81 (m, 5H), 7.74 (t, J = 7.9 Hz, 1H), 7.48 (d, J = 8.4 Hz, 2H), 7.41 – 7.32 (m, 1H), 6.93 (d, J = 9.0 Hz, 2H), 3.73 (d, J = 13.0 Hz, 2H), 3.57 (d, J = 11.6 Hz, 2H), 3.27 – 3.22 (m, 3H), 3.15 – 3.10 (m, 3H), 2.92 (t, J = 12.6 Hz, 3H), 1.72 – 1.62 (m, 2H), 1.58 – 1.48 (m, 2H), 1.38 – 1.25 (m, 10H), 1.16 (d, J = 6.8 Hz, 6H).

LC–MS (ESI-TOF) Calcd for $C_{46}H_{53}Cl_3FN_8O_6S_2^+$ [M+H]$^+$: 1001.26; found: 1001.32.

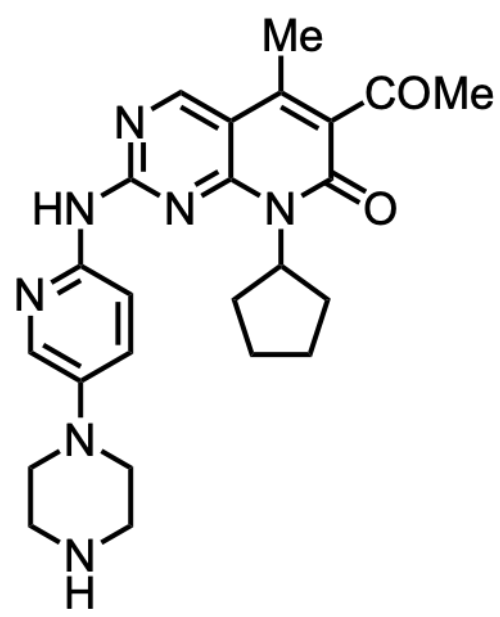

6-acetyl-8-cyclopentyl-5-methyl-2-((5-(piperazin-1-yl)pyridin-2-yl)amino)pyrido[2,3-*d*]pyrimidin-7(8*H*)-one (Palbociclib; 18)

Palbociclib is a commercially available CDK4/6 inhibitor.

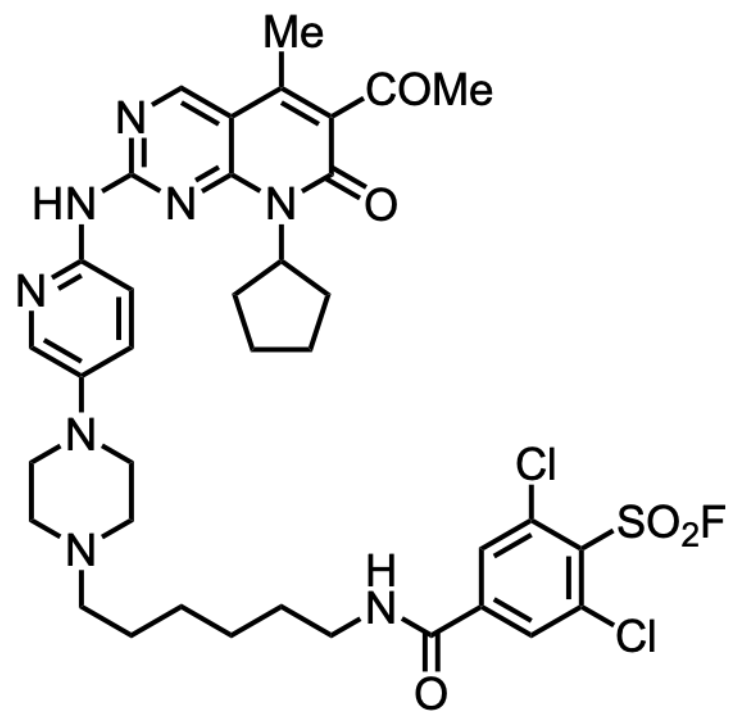

4-((6-(4-(6-((6-acetyl-8-cyclopentyl-5-methyl-7-oxo-7,8-dihydropyrido[2,3-*d*]pyrimidin-2-yl)amino)pyridin-3-yl)piperazin-1-yl)hexyl)carbamoyl)-2,6-dichlorobenzenesulfonyl fluoride (ZZ7-22-001; 19)

Following General Procedure using Palbociclib-NH_2[5], purification by preparative RP-HPLC afforded the title compound (0.58 mg, 7% yield).

^{1}H NMR (500 MHz, DMSO-d_6) δ 10.27 (s, 1H), 9.64 (br s, 1H), 8.99 (t, *J* = 5.6 Hz, 1H), 8.96 (s, 1H), 8.19 (s, 2H), 8.12 (d, *J* = 3.0 Hz, 1H), 7.90 (d, *J* = 9.0 Hz, 1H), 7.59 (dd, *J* = 9.1, 3.1 Hz, 1H), 5.82 (t, *J* = 8.9 Hz, 1H), 3.86 (d, *J* = 12.9 Hz, 2H), 3.63 – 3.57 (m, 2H), 3.30 (q, *J* = 6.7 Hz, 2H), 3.20 – 3.14 (m, 4H), 3.06 – 2.99 (m, 2H), 2.42 (s, 3H), 2.31 (s, 3H), 2.28 – 2.20 (m, 2H), 1.93 – 1.86 (m, 2H), 1.81 – 1.75 (m, 2H), 1.72 – 1.65 (m, 2H), 1.62 – 1.54 (m, 4H), 1.39 – 1.31 (m, 4H). LC–MS (ESI-TOF) Calcd for $C_{37}H_{44}Cl_2FN_8O_5S^+$ $[M+H]^+$: 801.25; found: 801.35.

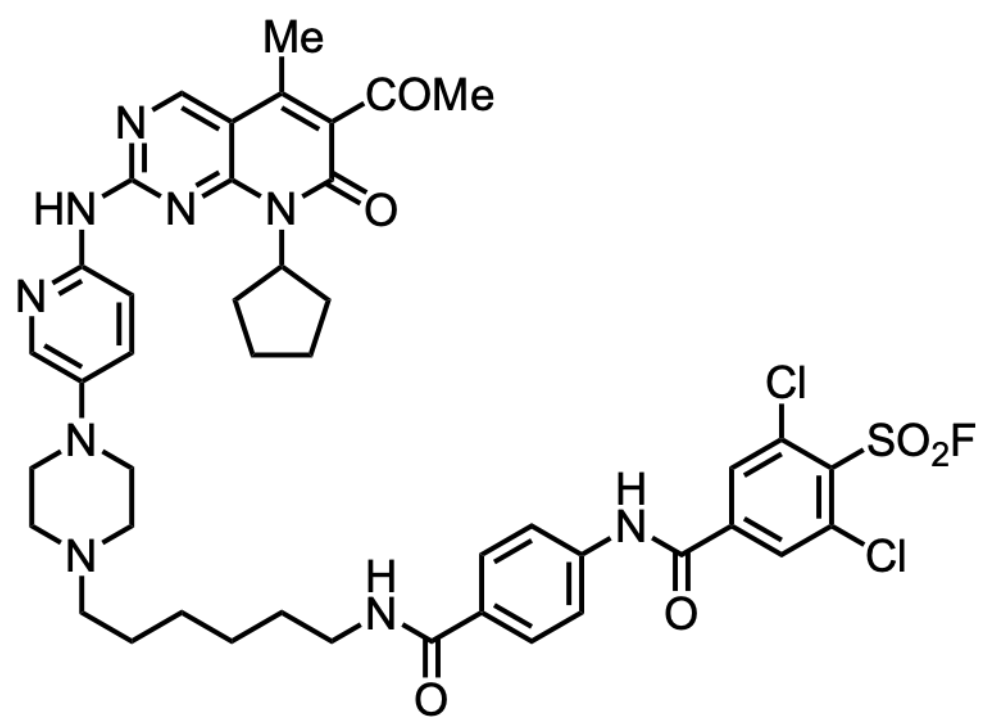

4-((4-((6-(4-(6-((6-acetyl-8-cyclopentyl-5-methyl-7-oxo-7,8-dihydropyrido[2,3-*d*]pyrimidin-2-yl)amino)pyridin-3-yl)piperazin-1-yl)hexyl)carbamoyl)phenyl)carbamoyl)-2,6-dichlorobenzenesulfonyl fluoride (ZZ7-22-002; 20)

Following General Procedure using Palbociclib-NH_2, purification by preparative RP-HPLC afforded the title compound (1.06 mg, 12% yield).

^{1}H NMR (500 MHz, DMSO-d_6) δ 10.87 (s, 1H), 10.20 (s, 1H), 9.71 (br s, 1H), 8.96 (s, 1H), 8.44 (t, *J* = 5.7 Hz, 1H), 8.34 (s, 2H), 8.13 (d, *J* = 3.0 Hz, 1H), 7.95 – 7.80 (m, 5H), 7.61 – 7.53 (m, 1H), 5.92 – 5.76 (m, 1H), 3.85 (d, *J* = 12.7 Hz, 2H), 3.60 (d, *J* = 12.1 Hz, 2H), 3.18 – 3.12 (m, 6H), 3.06 – 3.00 (m, 2H), 2.42 (s, 3H), 2.31 (s, 3H), 2.29 – 2.21 (m, 2H), 1.94 – 1.83 (m, 2H), 1.83 – 1.73 (m, 2H), 1.73 – 1.64 (m, 2H), 1.64 – 1.53 (m, 4H), 1.41 – 1.29 (m, 4H).

LC–MS (ESI-TOF) Calcd for $C_{44}H_{49}Cl_2FN_9O_6S^+$ $[M+H]^+$: 920.29; found: 920.16.

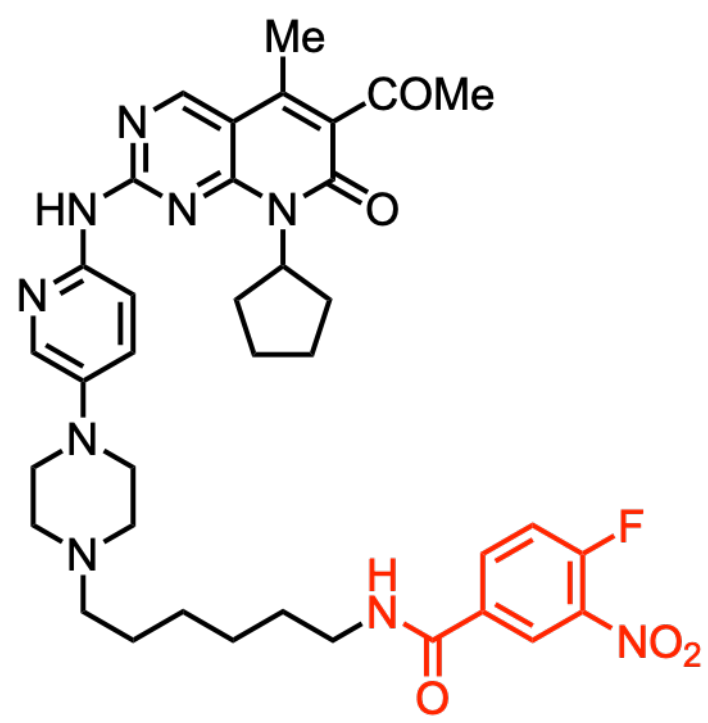

***N*-(6-(4-(6-((6-acetyl-8-cyclopentyl-5-methyl-7-oxo-7,8-dihydropyrido[2,3-*d*]pyrimidin-2-yl)amino)pyridin-3-yl)piperazin-1-yl)hexyl)-4-fluoro-3-nitrobenzamide (ZZ7-18-084; 21)**

ZZ7-18-084 is a reported CDK4/6 degrader.[6]

^{1}H NMR (500 MHz, DMSO-d_6) δ 10.25 (s, 1H), 9.71 (br s, 1H), 8.97 (s, 1H), 8.84 (t, *J* = 5.6 Hz, 1H), 8.62 (dd, *J* = 7.3, 2.3 Hz, 1H), 8.32 – 8.24 (m, 1H), 8.13 (d, *J* = 3.0 Hz, 1H), 7.91 (d, *J* = 9.1 Hz, 1H), 7.72 (dd, *J* = 11.1, 8.7 Hz, 1H), 7.58 (dd, *J* = 9.1, 3.0 Hz, 1H), 5.91 – 5.73 (m, 1H), 3.86 (d, *J* = 12.9 Hz, 2H), 3.62 – 3.59 (m, 2H), 3.35 – 3.26 (m, 2H), 3.24 – 3.12 (m, 4H), 3.02 (t, *J* = 12.4 Hz, 2H), 2.42 (s, 3H), 2.31 (s, 3H), 2.29 – 2.19 (m, 2H), 1.95 – 1.84 (m, 2H), 1.84 – 1.74 (m, 2H), 1.74 – 1.65 (m, 2H), 1.64 – 1.52 (m, 4H), 1.44 – 1.30 (m, 4H).

HRMS (ESI-TOF) Calcd for $C_{37}H_{45}FN_9O_5^+$ [M+H]$^+$: 714.35; found: 714.40.

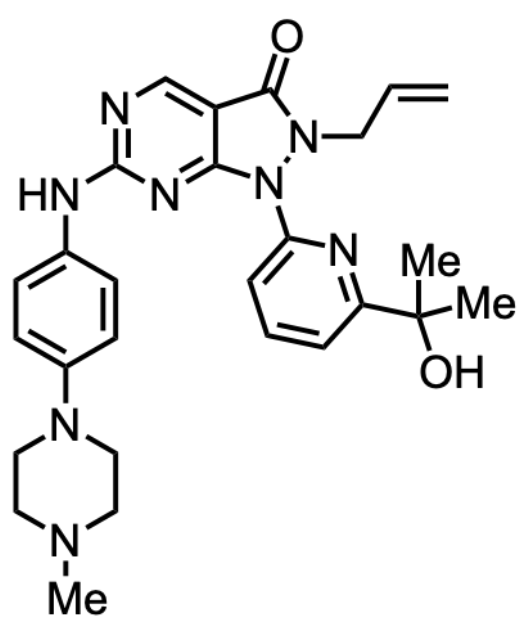

2-allyl-1-(6-(2-hydroxypropan-2-yl)pyridin-2-yl)-6-((4-(4-methylpiperazin-1-yl)phenyl)amino)-1,2-dihydro-3*H*-pyrazolo[3,4-*d*]pyrimidin-3-one (Adavosertib; 22)

Adavosertib is a commercially available WEE1 inhibitor.

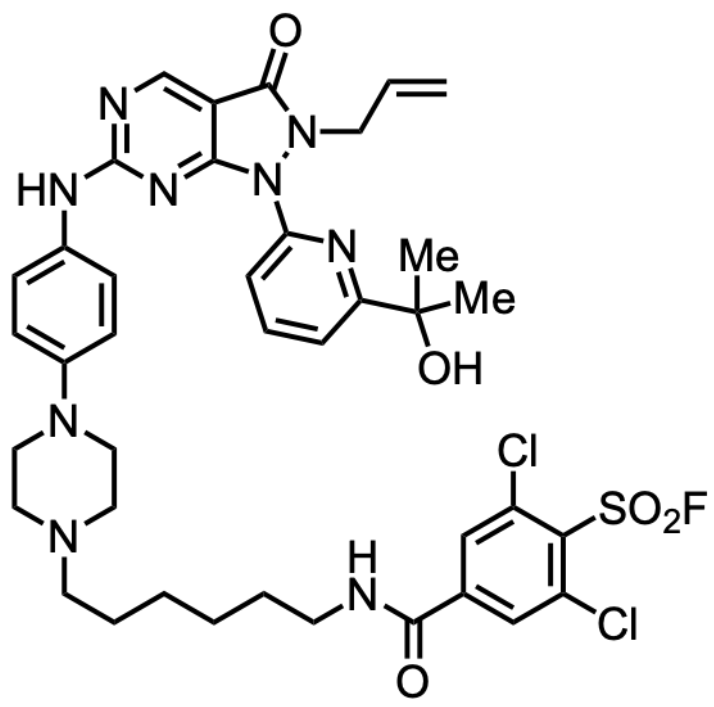

4-((6-(4-(4-((2-allyl-1-(6-(2-hydroxypropan-2-yl)pyridin-2-yl)-3-oxo-2,3-dihydro-1*H*-pyrazolo[3,4-*d*]pyrimidin-6-yl)amino)phenyl)piperazin-1-yl)hexyl)carbamoyl)-2,6-dichlorobenzenesulfonyl fluoride (ZZ7-22-003; 23)

Following General Procedure using Adavosertib-NH_2[7], purification by preparative RP-HPLC afforded the title compound (0.72 mg, 9% yield).

^{1}H NMR (500 MHz, DMSO-d_6) δ 10.20 (br s, 1H), 9.61 (br s, 1H), 8.99 (t, *J* = 5.6 Hz, 1H), 8.84 (s, 1H), 8.19 (s, 2H), 8.03 (t, *J* = 7.9 Hz, 1H), 7.74 (d, *J* = 8.0 Hz, 1H), 7.66 – 7.57 (m, 3H), 7.00 (d, *J* = 8.8 Hz, 2H), 5.66 (ddt, *J* = 16.7, 10.3, 5.9 Hz, 1H), 4.99 (dd, *J* = 10.3, 1.6 Hz, 1H), 4.82 (dd, *J* = 16.7, 1.6 Hz, 1H), 4.68 (d, *J* = 5.9 Hz, 2H), 3.79 (d, *J* = 12.9 Hz, 2H), 3.58 (d, *J* = 11.9 Hz, 2H), 3.32 – 3.28 (m, 2H), 3.17 – 3.10 (m, 4H), 2.95 (t, *J* = 12.4 Hz, 2H), 1.73 – 1.65 (m, 2H), 1.61 – 1.51 (m, 2H), 1.46 (s, 6H), 1.39 – 1.32 (m, 4H).

LC–MS (ESI-TOF) Calcd for $C_{39}H_{45}Cl_2FN_9O_5S^+$ $[M+H]^+$: 840.26; found: 840.30.

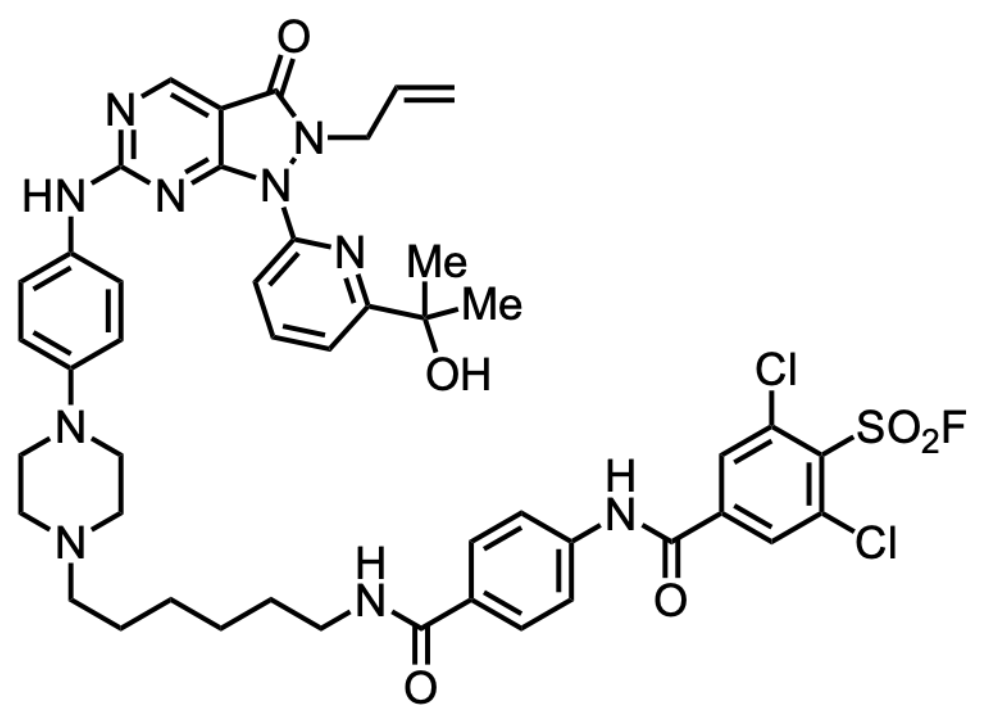

4-((4-((6-(4-(4-((2-allyl-1-(6-(2-hydroxypropan-2-yl)pyridin-2-yl)-3-oxo-2,3-dihydro-1*H*-pyrazolo[3,4-*d*]pyrimidin-6-yl)amino)phenyl)piperazin-1-yl)hexyl)carbamoyl)phenyl)carbamoyl)-2,6-dichlorobenzenesulfonyl fluoride (ZZ7-22-004; 24)

Following General Procedure using Adavosertib-NH_2, purification by preparative RP-HPLC afforded the title compound (0.70 mg, 7% yield).

^{1}H NMR (500 MHz, DMSO-d_6) δ 10.87 (s, 1H), 10.20 (br s, 1H), 9.59 (br s, 1H), 8.84 (s, 1H), 8.45 (t, *J* = 5.4 Hz, 1H), 8.33 (s, 2H), 8.03 (t, *J* = 7.9 Hz, 1H), 7.89 (d, *J* = 8.5 Hz, 2H), 7.84 (d, *J* = 8.5 Hz, 2H), 7.74 (d, *J* = 8.0 Hz, 1H), 7.70 – 7.53 (m, 3H), 7.00 (d, *J* = 8.7 Hz, 2H), 5.66 (ddt, *J* = 16.8, 10.4, 5.9 Hz, 1H), 5.05 – 4.96 (m, 1H), 4.82 (dd, *J* = 16.8, 1.6 Hz, 1H), 4.68 (d, *J* = 5.9 Hz, 2H), 3.78 (d, *J* = 12.8 Hz, 2H), 3.59 (d, *J* = 11.8 Hz, 2H), 3.29 – 3.24 (m, 2H), 3.20 – 3.09 (m, 4H), 2.94 (t, *J* = 12.2 Hz, 2H), 1.76 – 1.64 (m, 2H), 1.60 – 1.52 (m, 2H), 1.46 (s, 6H), 1.41 – 1.31 (m, 4H).

LC–MS (ESI-TOF) Calcd for $C_{46}H_{50}Cl_2FN_{10}O_6S^+$ $[M+H]^+$: 959.30; found: 959.31.

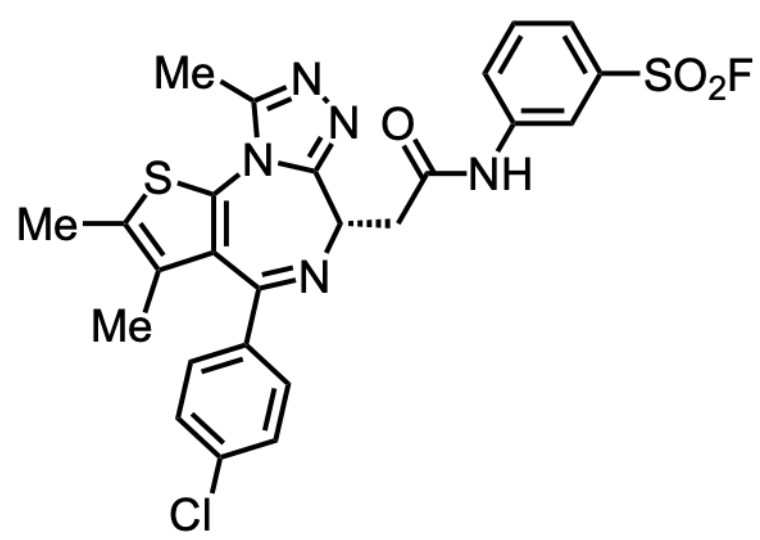

(*S*)-3-(2-(4-(4-chlorophenyl)-2,3,9-trimethyl-6*H*-thieno[3,2-*f*][1,2,4]triazolo[4,3-*a*][1,4]diazepin-6-yl)acetamido)benzenesulfonyl fluoride (ZZ7-10-066; 25)

Following General Procedure using JQ1-acid, purification by preparative RP-HPLC afforded the title compound (1.37 mg, 25% yield).

^{1}H NMR (500 MHz, DMSO-d$_6$) δ 10.94 (s, 1H), 8.52 (t, *J* = 2.1 Hz, 1H), 7.94 – 7.88 (m, 1H), 7.81 – 7.76 (m, 1H), 7.76 – 7.70 (m, 1H), 7.44 (d, *J* = 8.6 Hz, 2H), 7.38 (d, *J* = 8.6 Hz, 2H), 4.60 (t, *J* = 7.2 Hz, 1H), 3.58 – 3.52 (m, 2H), 2.58 (s, 3H), 2.38 (s, 3H), 1.59 (s, 3H).

LC–MS (ESI-TOF) Calcd for $C_{25}H_{22}ClFN_5O_3S_2^+$ [M+H]$^+$: 558.08; found: 558.19.

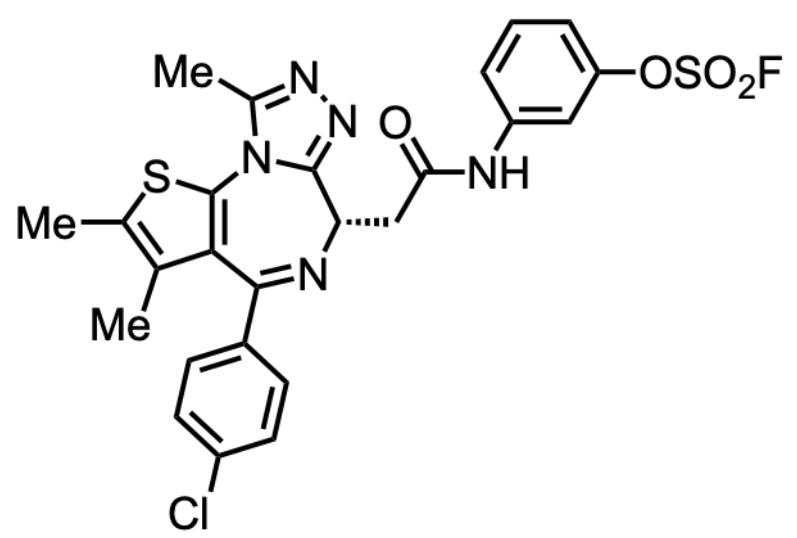

(*S*)-3-(2-(4-(4-chlorophenyl)-2,3,9-trimethyl-6*H*-thieno[3,2-*f*][1,2,4]triazolo[4,3-*a*][1,4]diazepin-6-yl)acetamido)phenyl sulfurofluoridate (ZZ7-10-067; 26)

Following General Procedure using JQ1-acid, purification by preparative RP-HPLC afforded the title compound (2.62 mg, 46% yield).

^{1}H NMR (500 MHz, DMSO-d_6) δ 10.76 (s, 1H), 7.99 (t, J = 2.3 Hz, 1H), 7.64 – 7.58 (m, 1H), 7.55 (t, J = 8.2 Hz, 1H), 7.48 (d, J = 8.7 Hz, 2H), 7.42 (d, J = 8.7 Hz, 2H), 7.27 (dd, J = 8.0, 2.6 Hz, 1H), 4.61 (t, J = 7.1 Hz, 1H), 3.56 – 3.54 (m, 2H), 2.61 (s, 3H), 2.42 (s, 3H), 1.63 (s, 3H).

LC–MS (ESI-TOF) Calcd for $C_{25}H_{22}ClFN_5O_4S_2^+$ $[M+H]^+$: 574.08; found: 574.29.

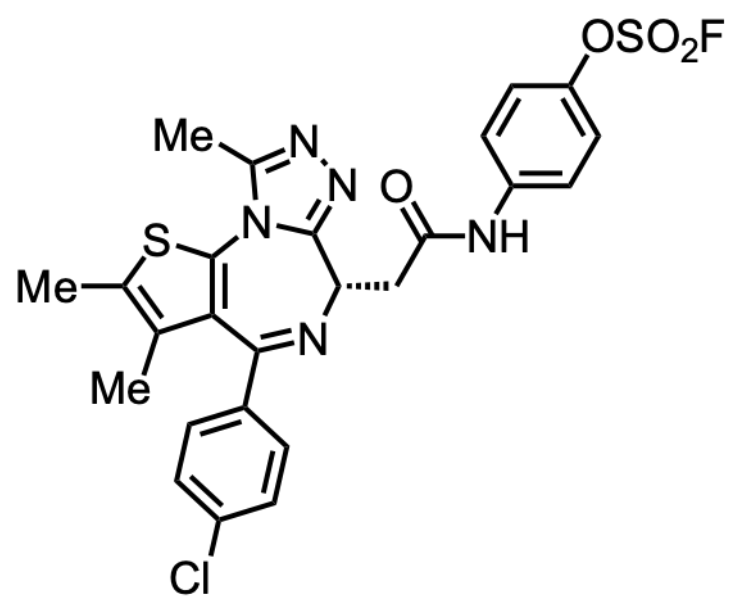

(*S*)-4-(2-(4-(4-chlorophenyl)-2,3,9-trimethyl-6*H*-thieno[3,2-*f*][1,2,4]triazolo[4,3-*a*][1,4]diazepin-6-yl)acetamido)phenyl sulfurofluoridate (ZZ7-10-073; 27)

Following General Procedure using JQ1-acid, purification by preparative RP-HPLC afforded the title compound (0.85 mg, 15% yield).

^{1}H NMR (500 MHz, DMSO-d$_6$) δ 10.65 (s, 1H), 7.79 (d, J = 9.2 Hz, 2H), 7.54 (d, J = 9.2 Hz, 2H), 7.47 (d, J = 8.7 Hz, 2H), 7.40 (d, J = 8.7 Hz, 2H), 4.59 (t, J = 7.1 Hz, 1H), 3.47 – 3.45 (m, 2H), 2.59 (s, 3H), 2.40 (s, 3H), 1.62 (s, 3H).

LC–MS (ESI-TOF) Calcd for $C_{25}H_{22}ClFN_5O_4S_2^+$ $[M+H]^+$: 574.08; found: 574.19.

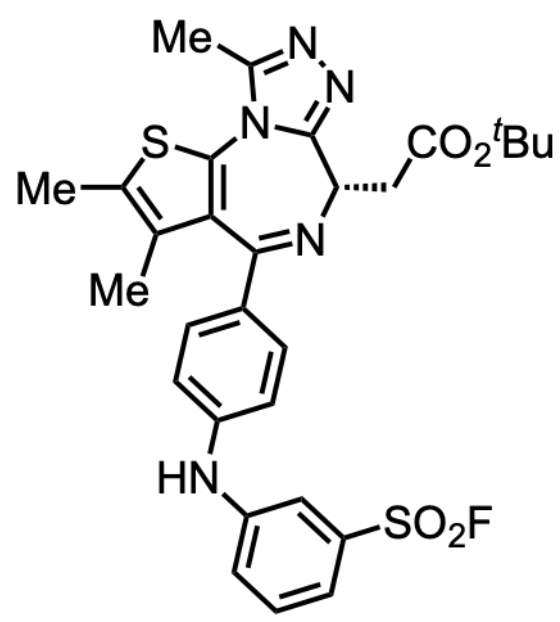

***tert*-butyl (*S*)-2-(4-(4-((3-(fluorosulfonyl)phenyl)amino)phenyl)-2,3,9-trimethyl-6*H*-thieno[3,2-*f*][1,2,4]triazolo[4,3-*a*][1,4]diazepin-6-yl)acetate (ZZ7-10-069; 28)**

Following literature procedure[1] with slight modification: JQ1 (0.01 mmol), XPhos Pd G4 (10%), Cs_2CO_3 (1.0 equiv), aniline (1.5 equiv), toluene (1.0 mL), 120 °C, 3 h. Purification by preparative RP-HPLC afforded the title compound (3.15 mg, 53% yield).

^{1}H NMR (500 MHz, DMSO-d_6) δ 9.19 (s, 1H), 7.66 – 7.60 (m, 3H), 7.54 (dt, J = 6.1, 2.5 Hz, 1H), 7.40 (d, J = 8.2 Hz, 2H), 7.17 (d, J = 9.0 Hz, 2H), 4.43 (dd, J = 8.2, 6.4 Hz, 1H), 3.34 – 3.29 (m, 2H), 2.60 (s, 3H), 2.43 (s, 3H), 1.73 (s, 3H), 1.42 (s, 9H).

LC–MS (ESI-TOF) Calcd for $C_{29}H_{31}FN_5O_4S_2^+$ $[M+H]^+$: 596.18; found: 596.40.

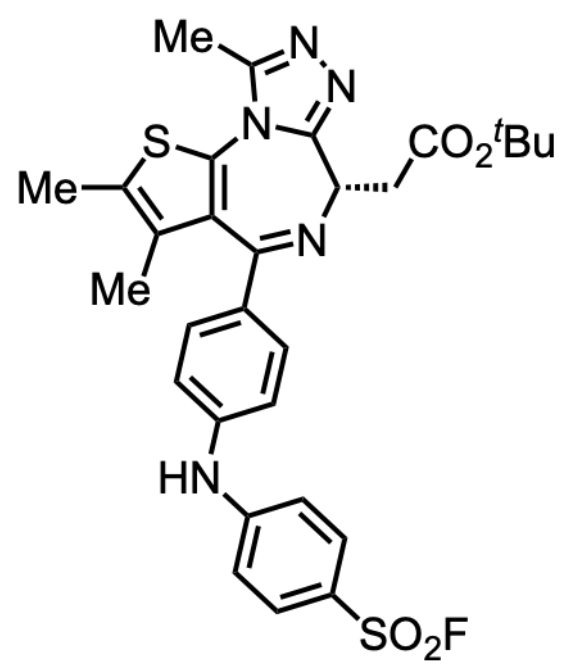

***tert*-butyl (*S*)-2-(4-(4-((4-(fluorosulfonyl)phenyl)amino)phenyl)-2,3,9-trimethyl-6*H*-thieno[3,2-*f*][1,2,4]triazolo[4,3-*a*][1,4]diazepin-6-yl)acetate (ZZ7-10-071; 29)**

Following literature procedure[1] with slight modification: JQ1 (0.01 mmol), XPhos Pd G4 (10%), Cs_2CO_3 (1.0 equiv), aniline (1.5 equiv), toluene (1.0 mL), 120 °C, 3 h. Purification by preparative RP-HPLC afforded the title compound (0.55 mg, 9% yield).

^{1}H NMR (500 MHz, DMSO-d$_6$) δ 9.55 (s, 1H), 7.89 (d, *J* = 9.0 Hz, 2H), 7.42 (d, *J* = 8.3 Hz, 2H), 7.31 – 7.23 (m, 4H), 4.41 (td, *J* = 8.6, 6.2 Hz, 1H), 3.29 – 3.27 (m, 2H), 2.60 (s, 3H), 2.43 (s, 3H), 1.73 (s, 3H), 1.43 (s, 9H).

LC–MS (ESI-TOF) Calcd for $C_{29}H_{31}FN_5O_4S_2^+$ [M+H]$^+$: 596.18; found: 596.40.

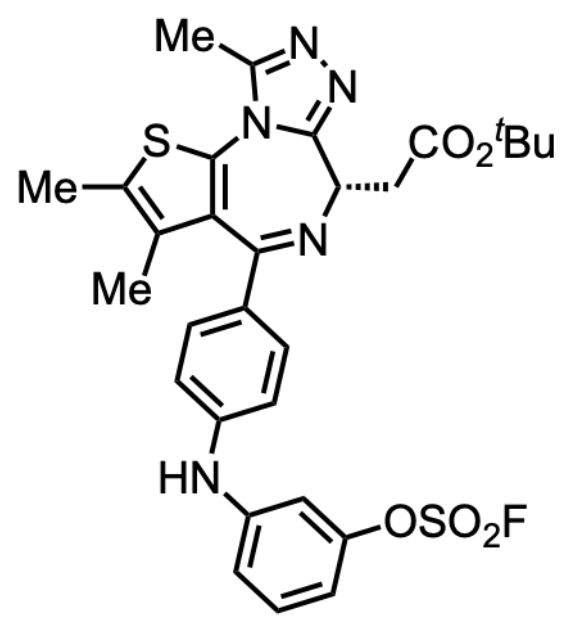

***tert*-butyl (*S*)-2-(4-(4-((3-((fluorosulfonyl)oxy)phenyl)amino)phenyl)-2,3,9-trimethyl-6*H*-thieno[3,2-*f*][1,2,4]triazolo[4,3-*a*][1,4]diazepin-6-yl)acetate (ZZ7-10-068; 30)**

Following literature procedure[1] with slight modification: JQ1 (0.01 mmol), XPhos Pd G4 (10%), Cs_2CO_3 (1.0 equiv), aniline (1.5 equiv), toluene (1.0 mL), 120 °C, 3 h. Purification by preparative RP-HPLC afforded the title compound (2.80 mg, 46% yield).

^1H NMR (500 MHz, DMSO-d_6) δ 9.04 (s, 1H), 7.44 (t, J = 8.2 Hz, 1H), 7.36 (d, J = 8.2 Hz, 2H), 7.25 – 7.18 (m, 2H), 7.13 (d, J = 9.0 Hz, 2H), 7.03 (dd, J = 8.4, 2.3 Hz, 1H), 4.43 (dd, J = 8.1, 6.4 Hz, 1H), 3.33 – 3.30 (m, 2H), 2.60 (s, 3H), 2.43 (s, 3H), 1.73 (s, 3H), 1.42 (s, 9H).

LC–MS (ESI-TOF) Calcd for $C_{29}H_{31}FN_5O_5S_2^+$ $[M+H]^+$: 612.17; found: 612.30.

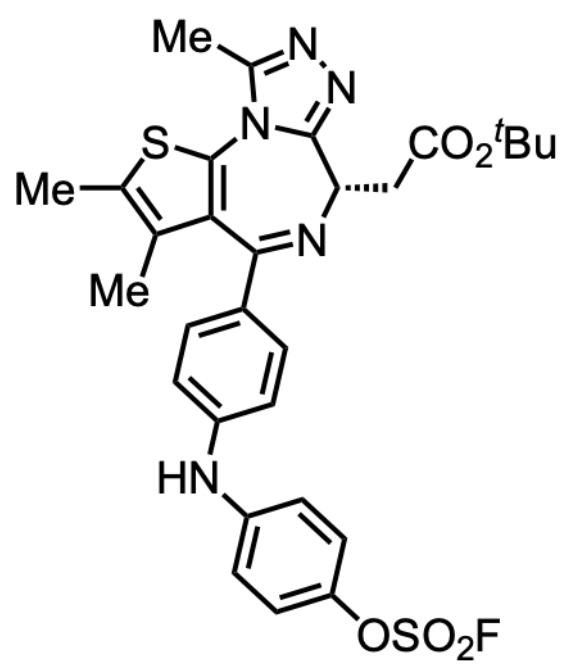

***tert*-butyl (*S*)-2-(4-(4-((4-((fluorosulfonyl)oxy)phenyl)amino)phenyl)-2,3,9-trimethyl-6*H*-thieno[3,2-*f*][1,2,4]triazolo[4,3-*a*][1,4]diazepin-6-yl)acetate (ZZ7-10-072; 31)**

Following literature procedure[1] with slight modification: JQ1 (0.01 mmol), XPhos Pd G4 (10%), Cs_2CO_3 (1.0 equiv), aniline (1.5 equiv), toluene (1.0 mL), 120 °C, 3 h. Purification by preparative RP-HPLC afforded the title compound (0.81 mg, 13% yield).

^{1}H NMR (500 MHz, DMSO-d$_6$) δ 8.86 (s, 1H), 7.44 (d, *J* = 9.0 Hz, 2H), 7.35 – 7.29 (m, 2H), 7.22 (d, *J* = 9.1 Hz, 2H), 7.10 (d, *J* = 9.1 Hz, 2H), 4.40 – 4.30 (m, 1H), 3.31 – 3.28 (m, 2H), 2.59 (s, 3H), 2.42 (s, 3H), 1.73 (s, 3H), 1.42 (s, 9H).

LC–MS (ESI-TOF) Calcd for $C_{29}H_{31}FN_5O_5S_2^+$ [M+H]$^+$: 612.17; found: 612.70.

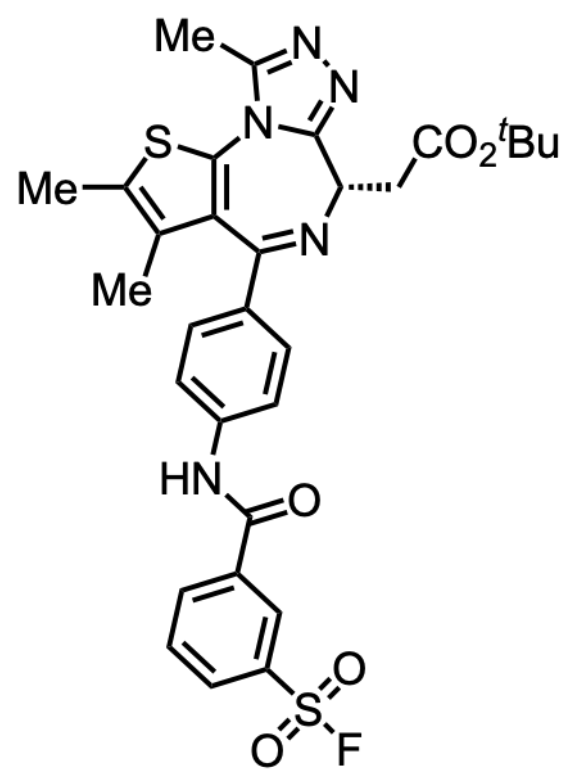

***tert*-butyl (*S*)-2-(4-(4-(3-(fluorosulfonyl)benzamido)phenyl)-2,3,9-trimethyl-6*H*-thieno[3,2-*f*][1,2,4]triazolo[4,3-*a*][1,4]diazepin-6-yl)acetate (ZZ7-11-042; 32)**

Following General Procedure using JQ1-NH_2, purification by preparative RP-HPLC afforded the title compound (0.78 mg, 13% yield).

^{1}H NMR (500 MHz, DMSO-d_6) δ 10.82 (s, 1H), 8.61 (s, 1H), 8.46 (d, *J* = 8.1 Hz, 1H), 8.39 – 8.30 (m, 1H), 7.95 (t, *J* = 7.9 Hz, 1H), 7.87 – 7.75 (m, 2H), 7.44 (d, *J* = 8.5 Hz, 2H), 4.41 (dd, *J* = 8.1, 6.5 Hz, 1H), 3.34 – 3.30 (m, 2H), 2.60 (s, 3H), 2.41 (s, 3H), 1.66 (s, 3H), 1.41 (s, 9H).

LC–MS (ESI-TOF) Calcd for $C_{30}H_{31}FN_5O_5S_2^+$ $[M+H]^+$: 624.17; found: 624.45.

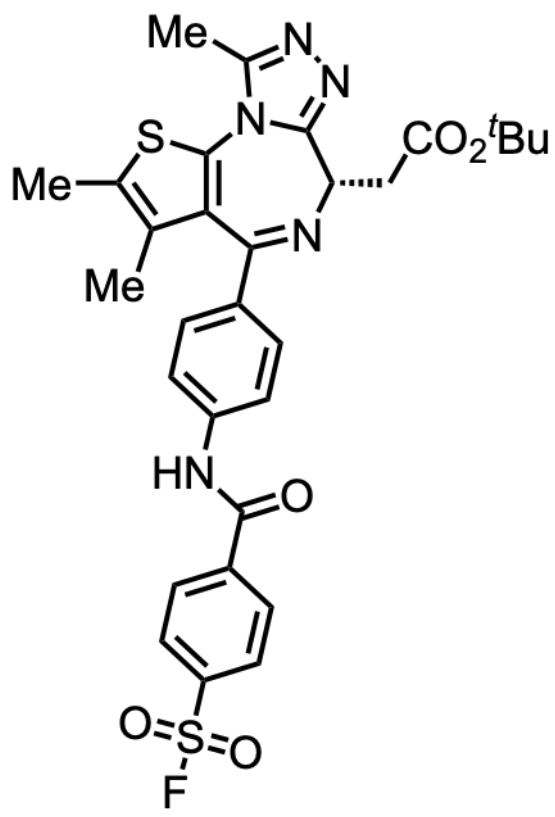

***tert*-butyl (*S*)-2-(4-(4-(4-(fluorosulfonyl)benzamido)phenyl)-2,3,9-trimethyl-6*H*-thieno[3,2-*f*][1,2,4]triazolo[4,3-*a*][1,4]diazepin-6-yl)acetate (ZZ7-11-043; 33)**

Following General Procedure using JQ1-NH_2, purification by preparative RP-HPLC afforded the title compound (0.80 mg, 13% yield).

^{1}H NMR (500 MHz, DMSO-d_6) δ 10.68 (s, 1H), 8.17 (d, *J* = 8.5 Hz, 2H), 8.08 (d, *J* = 8.5 Hz, 2H), 7.84 (d, *J* = 8.7 Hz, 2H), 7.44 (d, *J* = 8.7 Hz, 2H), 4.40 (dd, *J* = 8.2, 6.3 Hz, 1H), 3.32 – 3.30 (m, 2H), 2.61 (s, 3H), 2.43 (s, 3H), 1.68 (s, 3H), 1.43 (s, 9H).

LC–MS (ESI-TOF) Calcd for $C_{30}H_{31}FN_5O_5S_2^+$ $[M+H]^+$: 624.17; found: 624.40.

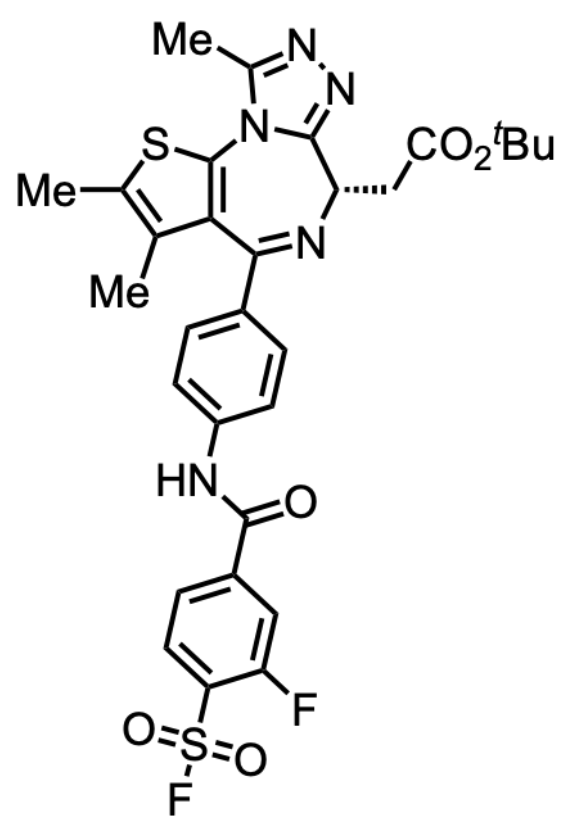

***tert*-butyl (*S*)-2-(4-(4-(3-fluoro-4-(fluorosulfonyl)benzamido)phenyl)-2,3,9-trimethyl-6*H*-thieno[3,2-*f*][1,2,4]triazolo[4,3-*a*][1,4]diazepin-6-yl)acetate (ZZ7-16-038; 34)**

Following General Procedure using JQ1-NH_2, purification by preparative RP-HPLC afforded the title compound (1.30 mg, 20% yield).

^{1}H NMR (500 MHz, DMSO-d_6) δ 10.81 (s, 1H), 8.30 – 8.25 (m, 1H), 8.22 (d, *J* = 9.8 Hz, 1H), 8.07 (d, *J* = 8.6 Hz, 1H), 7.82 (d, *J* = 8.8 Hz, 2H), 7.50 (d, *J* = 8.8 Hz, 2H), 4.43 – 4.38 (m, 1H), 3.24 – 3.21 (m, 2H), 2.61 (s, 3H), 2.42 (s, 3H), 1.67 (s, 3H), 1.43 (s, 9H).

LC–MS (ESI-TOF) Calcd for $C_{30}H_{30}F_2N_5O_5S_2^+$ $[M+H]^+$: 642.17; found: 642.25.

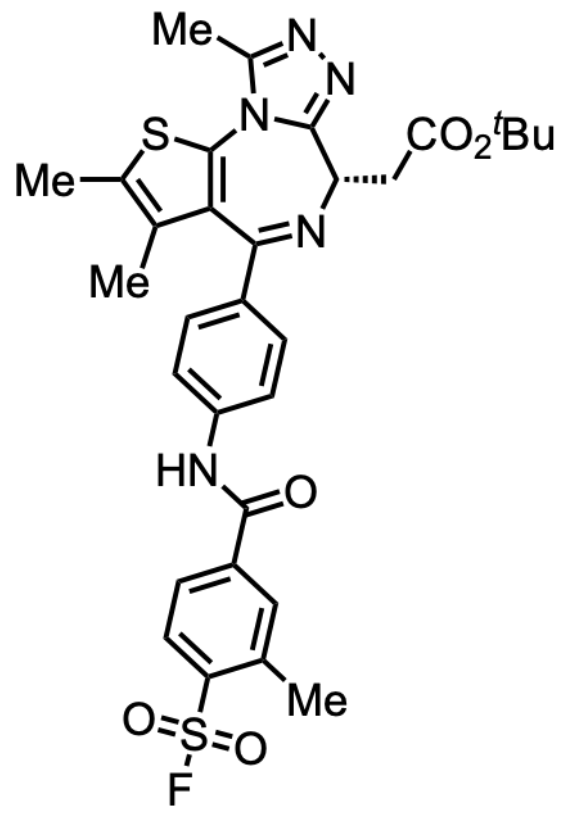

***tert*-butyl (*S*)-2-(4-(4-(4-(fluorosulfonyl)-3-methylbenzamido)phenyl)-2,3,9-trimethyl-6*H*-thieno[3,2-*f*][1,2,4]triazolo[4,3-*a*][1,4]diazepin-6-yl)acetate (ZZ7-16-040; 35)**

Following General Procedure using JQ1-NH_2, purification by preparative RP-HPLC afforded the title compound (1.40 mg, 22% yield).

^{1}H NMR (500 MHz, DMSO-d_6) δ 10.78 (s, 1H), 8.22 (d, J = 8.4 Hz, 1H), 8.13 (s, 1H), 8.04 (d, J = 8.3 Hz, 1H), 7.83 (d, J = 8.9 Hz, 2H), 7.53 – 7.39 (m, 2H), 4.46 – 4.39 (m, 1H), 3.38 – 3.31 (m, 2H), 2.71 (s, 3H), 2.62 (s, 3H), 2.43 (s, 3H), 1.67 (s, 3H), 1.43 (s, 9H).

LC–MS (ESI-TOF) Calcd for $C_{31}H_{33}FN_5O_5S_2^+$ $[M+H]^+$: 638.19; found: 638.25.

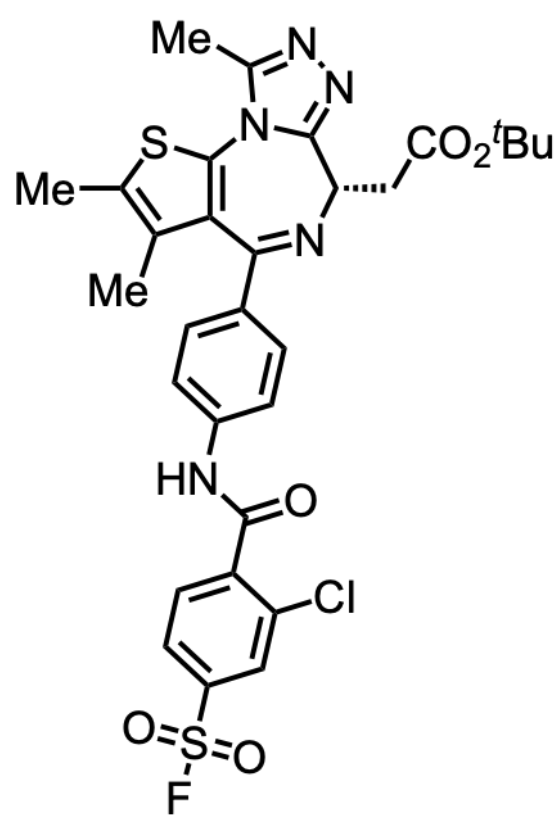

***tert*-butyl (*S*)-2-(4-(4-(2-chloro-4-(fluorosulfonyl)benzamido)phenyl)-2,3,9-trimethyl-6*H*-thieno[3,2-*f*][1,2,4]triazolo[4,3-*a*][1,4]diazepin-6-yl)acetate (ZZ7-16-041; 36)**

Following General Procedure using JQ1-NH_2, purification by preparative RP-HPLC afforded the title compound (1.86 mg, 28% yield).

^{1}H NMR (500 MHz, DMSO-d_6) δ 10.98 (s, 1H), 8.42 (d, *J* = 1.9 Hz, 1H), 8.23 (dd, *J* = 8.1, 1.9 Hz, 1H), 8.03 (d, *J* = 8.1 Hz, 1H), 7.74 (d, *J* = 8.6 Hz, 2H), 7.45 (d, *J* = 8.6 Hz, 2H), 4.45 – 4.38 (m, 1H), 3.36 – 3.27 (m, 2H), 2.61 (s, 3H), 2.43 (s, 3H), 1.67 (s, 3H), 1.42 (s, 9H).

LC–MS (ESI-TOF) Calcd for $C_{30}H_{30}ClFN_5O_5S_2^+$ $[M+H]^+$: 658.14; found: 658.20.

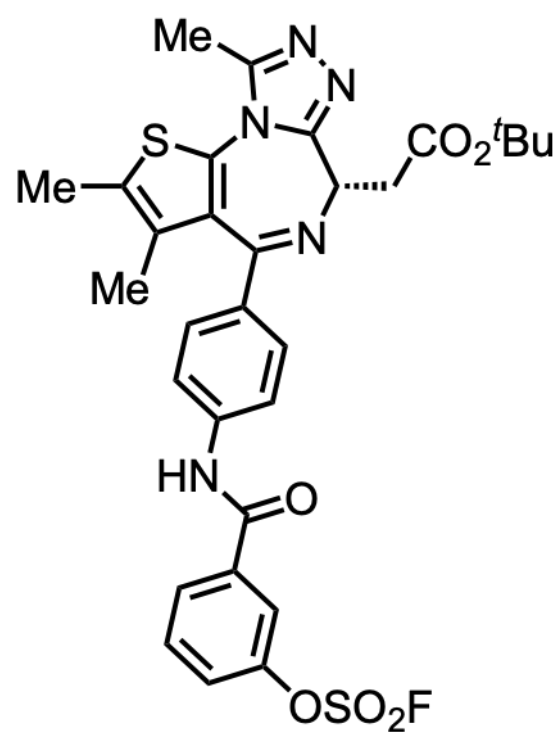

***tert*-butyl (*S*)-2-(4-(4-(3-((fluorosulfonyl)oxy)benzamido)phenyl)-2,3,9-trimethyl-6*H*-thieno[3,2-*f*][1,2,4]triazolo[4,3-*a*][1,4]diazepin-6-yl)acetate (ZZ7-14-038; 37)**

Following General Procedure using JQ1-NH_2, purification by preparative RP-HPLC afforded the title compound (1.27 mg, 20% yield).

^1H NMR (500 MHz, DMSO-d_6) δ 10.63 (s, 1H), 8.15 – 8.09 (m, 2H), 7.92 – 7.80 (m, 3H), 7.78 (t, *J* = 8.0 Hz, 1H), 7.44 (d, *J* = 8.3 Hz, 2H), 4.40 (dd, *J* = 8.2, 6.3 Hz, 1H), 3.30 – 3.28 (m, 2H), 2.61 (s, 3H), 2.43 (s, 3H), 1.68 (s, 3H), 1.43 (s, 9H).

LC–MS (ESI-TOF) Calcd for $C_{30}H_{31}FN_5O_6S_2^+$ $[M+H]^+$: 640.17; found: 640.20.

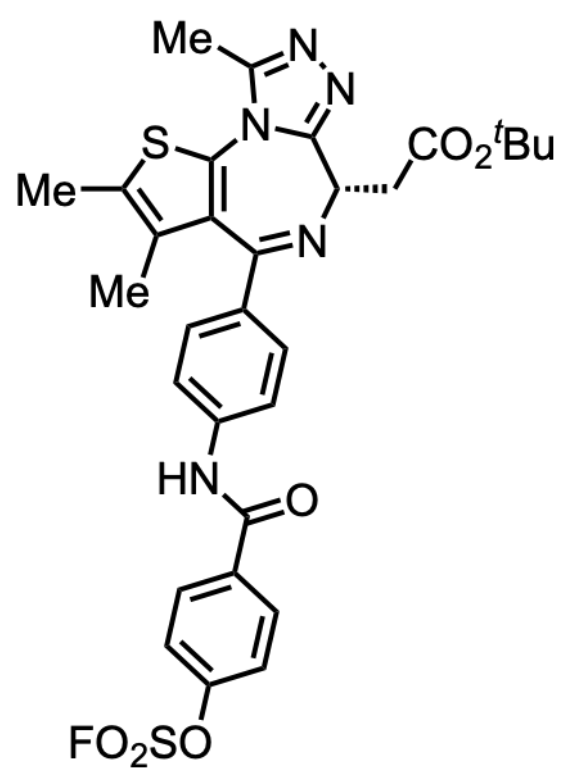

***tert*-butyl (*S*)-2-(4-(4-(4-((fluorosulfonyl)oxy)benzamido)phenyl)-2,3,9-trimethyl-6*H*-thieno[3,2-*f*][1,2,4]triazolo[4,3-*a*][1,4]diazepin-6-yl)acetate (ZZ7-14-039; 38)**

Following General Procedure using JQ1-NH_2, purification by preparative RP-HPLC afforded the title compound (1.39 mg, 22% yield).

^{1}H NMR (500 MHz, DMSO-d_6) δ 10.61 (s, 1H), 8.12 (d, J = 8.7 Hz, 2H), 7.82 (d, J = 8.5 Hz, 2H), 7.78 (d, J = 8.7 Hz, 2H), 7.43 (d, J = 8.5 Hz, 2H), 4.45 – 4.36 (m, 1H), 3.34 – 3.32 (m, 2H), 2.60 (s, 3H), 2.43 (s, 3H), 1.67 (s, 3H), 1.43 (s, 9H).

LC–MS (ESI-TOF) Calcd for $C_{30}H_{31}FN_5O_6S_2^+$ [M+H]$^+$: 640.17; found: 640.15.

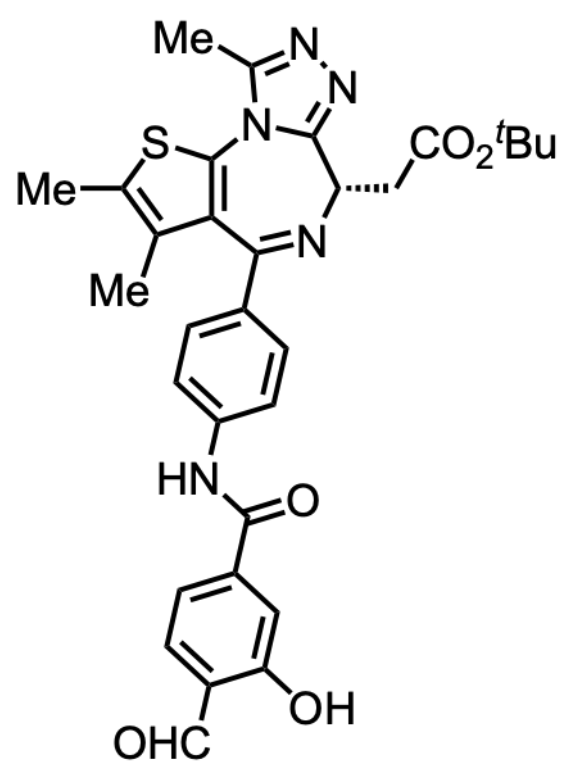

***tert*-butyl (*S*)-2-(4-(4-(4-formyl-3-hydroxybenzamido)phenyl)-2,3,9-trimethyl-6*H*-thieno[3,2-*f*][1,2,4]triazolo[4,3-*a*][1,4]diazepin-6-yl)acetate (ZZ7-14-041; 39)**

Following General Procedure, purification by preparative RP-HPLC afforded the title compound (0.76 mg, 13% yield).

^{1}H NMR (500 MHz, DMSO-d$_6$) δ 11.02 (s, 1H), 10.58 (s, 1H), 10.34 (s, 1H), 7.83 (d, *J* = 8.8 Hz, 2H), 7.77 (d, *J* = 8.0 Hz, 1H), 7.52 – 7.45 (m, 2H), 7.42 (d, *J* = 8.4 Hz, 2H), 4.40 (dd, *J* = 8.2, 6.3 Hz, 1H), 3.33 – 3.30 (m, 2H), 2.61 (s, 3H), 2.43 (s, 3H), 1.68 (s, 3H), 1.43 (s, 9H).

LC–MS (ESI-TOF) Calcd for $C_{31}H_{32}N_5O_5S^+$ $[M+H]^+$: 586.21; found: 586.29.

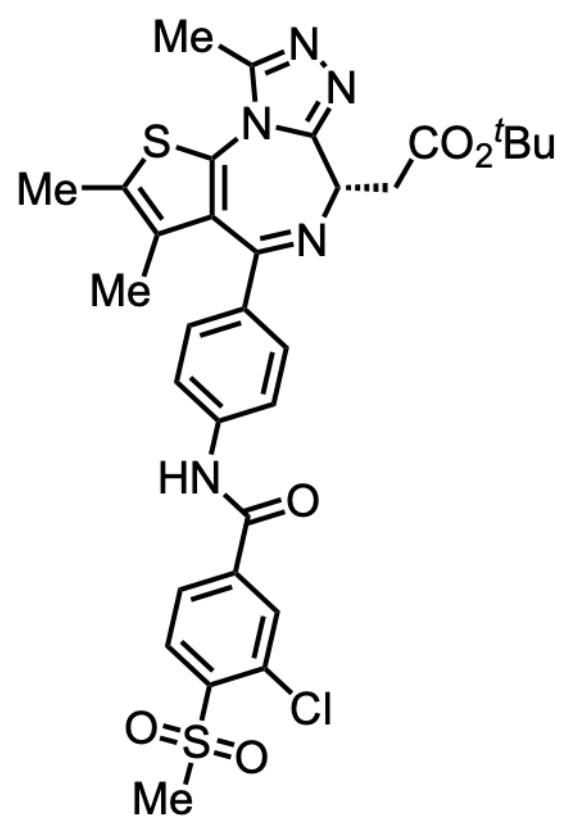

***tert*-butyl (*S*)-2-(4-(4-(3-chloro-4-(methylsulfonyl)benzamido)phenyl)-2,3,9-trimethyl-6*H*-thieno[3,2-*f*][1,2,4]triazolo[4,3-*a*][1,4]diazepin-6-yl)acetate (ZZ7-23-086; 40)**

Following General Procedure using JQ1-NH_2, purification by preparative RP-HPLC afforded the title compound (2.30 mg, 40% yield).

^{1}H NMR (500 MHz, DMSO-d_6) δ 10.75 (s, 1H), 8.26 (t, J = 1.9 Hz, 1H), 8.18 (d, J = 8.2 Hz, 1H), 8.15 – 8.09 (m, 1H), 7.83 (dd, J = 9.0, 2.6 Hz, 2H), 7.45 (dd, J = 8.6, 4.0 Hz, 2H), 4.48 – 4.36 (m, 1H), 3.43 (s, 3H), 3.18 – 3.08 (m, 2H), 2.61 (s, 3H), 2.43 (s, 3H), 1.67 (s, 3H), 1.43 (s, 9H).

LC–MS (ESI-TOF) Calcd for $C_{31}H_{33}ClN_5O_5S_2^+$ [M+H]$^+$: 654.16; found: 654.26.

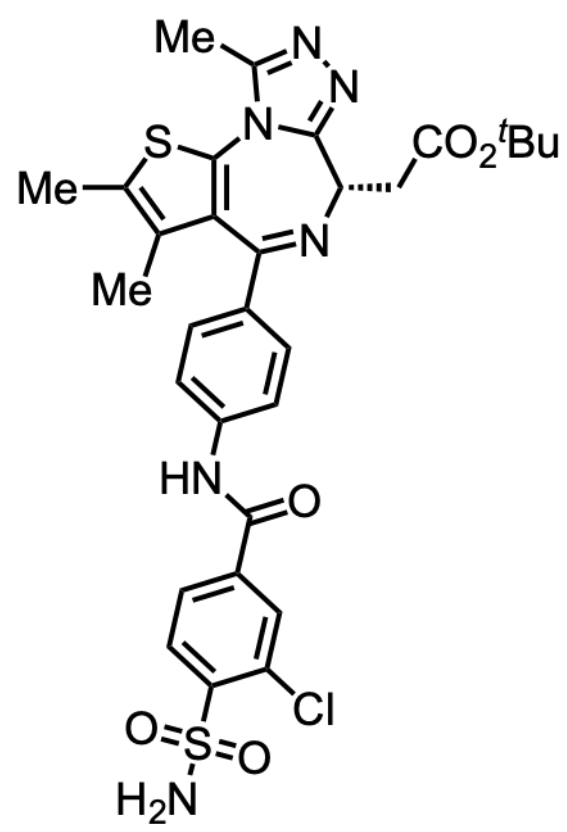

***tert*-butyl (*S*)-2-(4-(4-(3-chloro-4-sulfamoylbenzamido)phenyl)-2,3,9-trimethyl-6*H*-thieno[3,2-*f*][1,2,4]triazolo[4,3-*a*][1,4]diazepin-6-yl)acetate (ZZ7-23-087; 41)**

Following General Procedure using JQ1-NH_2, purification by preparative RP-HPLC afforded the title compound (2.70 mg, 41% yield).

^{1}H NMR (500 MHz, DMSO-d_6) δ 10.68 (s, 1H), 8.21 – 8.14 (m, 1H), 8.10 (d, *J* = 8.2 Hz, 1H), 8.06 – 8.00 (m, 1H), 7.85 – 7.79 (m, 2H), 7.79 (s, 2H), 7.49 – 7.39 (m, 2H), 4.46 – 4.36 (m, 1H), 3.34 – 3.31 (m, 2H), 2.61 (s, 3H), 2.43 (s, 3H), 1.67 (s, 3H), 1.43 (s, 9H).

LC–MS (ESI-TOF) Calcd for $C_{30}H_{32}ClN_6O_5S_2^+$ [M+H]$^+$: 655.16; found: 655.31.

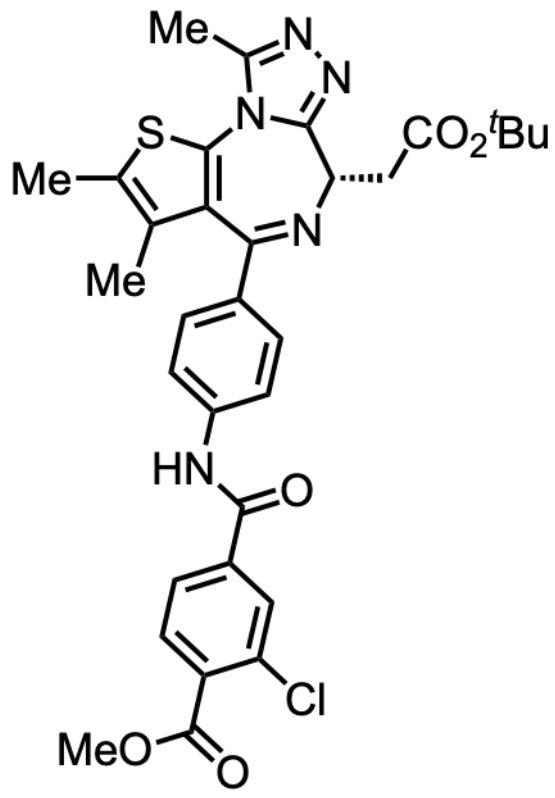

methyl (*S*)-4-((4-(6-(2-(*tert*-butoxy)-2-oxoethyl)-2,3,9-trimethyl-6*H*-thieno[3,2-*f*][1,2,4]triazolo[4,3-*a*][1,4]diazepin-4-yl)phenyl)carbamoyl)-2-chlorobenzoate (ZZ7-23-088; 42)

Following General Procedure using JQ1-NH_2, purification by preparative RP-HPLC afforded the title compound (2.80 mg, 44% yield).

^{1}H NMR (500 MHz, DMSO-d_6) δ 10.66 (s, 1H), 8.13 (d, *J* = 1.7 Hz, 1H), 7.99 (dd, *J* = 8.1, 1.7 Hz, 1H), 7.94 (d, *J* = 8.1 Hz, 1H), 7.83 (d, *J* = 8.6 Hz, 2H), 7.44 (d, *J* = 8.6 Hz, 2H), 4.40 (dd, *J* = 8.2, 6.3 Hz, 1H), 3.90 (s, 3H), 3.35 – 3.29 (m, 2H), 2.61 (s, 3H), 2.43 (s, 3H), 1.67 (s, 3H), 1.43 (s, 9H).

LC–MS (ESI-TOF) Calcd for $C_{32}H_{33}ClN_5O_5S^+$ $[M+H]^+$: 634.19; found: 634.31.

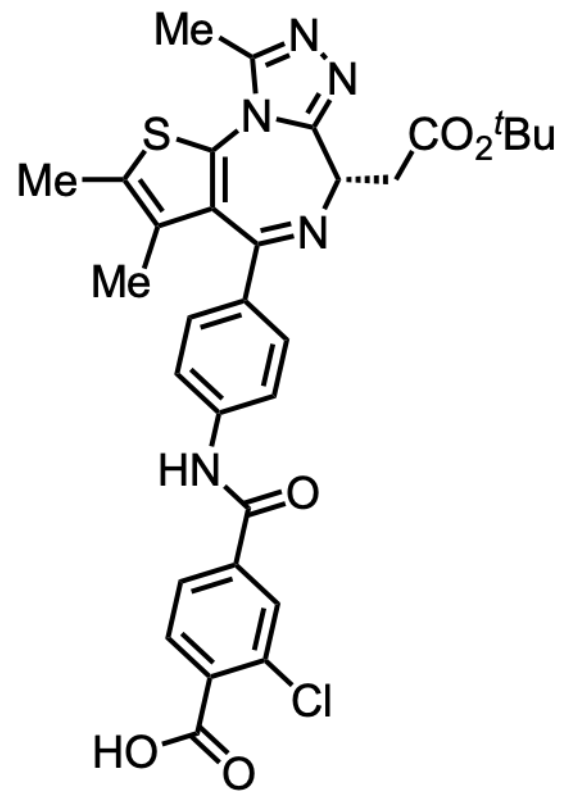

(*S*)-4-((4-(6-(2-(*tert*-butoxy)-2-oxoethyl)-2,3,9-trimethyl-6*H*-thieno[3,2-*f*][1,2,4]triazolo[4,3-*a*][1,4]diazepin-4-yl)phenyl)carbamoyl)-2-chlorobenzoic acid (ZZ7-23-089; 43)

Following General Procedure using JQ1-NH_2, purification by preparative RP-HPLC afforded the title compound (1.80 mg, 29% yield).

^{1}H NMR (500 MHz, DMSO-d_6) δ 10.64 (s, 1H), 8.13 – 8.05 (m, 1H), 8.00 – 7.87 (m, 2H), 7.83 (d, *J* = 8.3 Hz, 2H), 7.44 (d, *J* = 8.3 Hz, 2H), 4.45 – 4.38 (m, 1H), 3.19 – 3.16 (m, 2H), 2.61 (s, 3H), 2.43 (s, 3H), 1.68 (s, 3H), 1.44 (s, 9H).

LC–MS (ESI-TOF) Calcd for $C_{31}H_{31}ClN_5O_5S^+$ $[M+H]^+$: 620.17; found: 620.26.

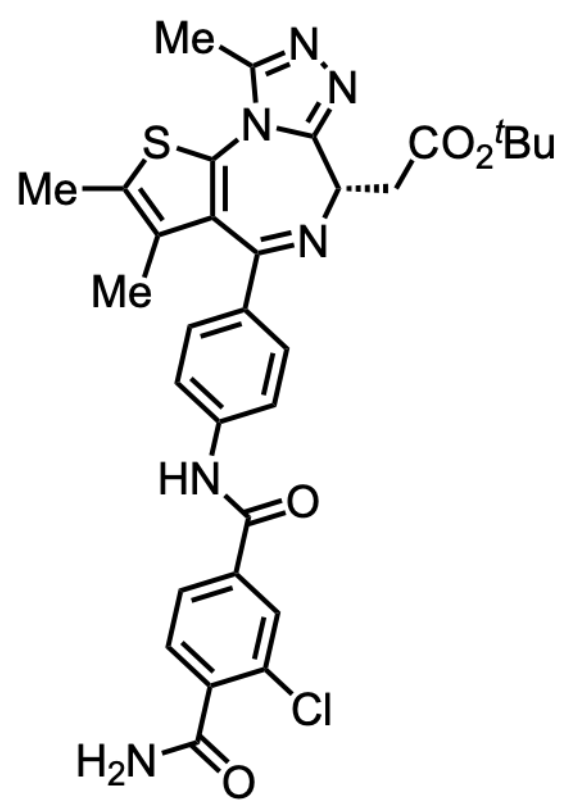

***tert*-butyl (*S*)-2-(4-(4-(4-carbamoyl-3-chlorobenzamido)phenyl)-2,3,9-trimethyl-6*H*-thieno[3,2-*f*][1,2,4]triazolo[4,3-*a*][1,4]diazepin-6-yl)acetate (ZZ7-23-090; 44)**

Following General Procedure using JQ1-NH_2, purification by preparative RP-HPLC afforded the title compound (2.24 mg, 36% yield).

^{1}H NMR (500 MHz, DMSO-d_6) δ 10.58 (s, 1H), 8.05 (d, *J* = 1.7 Hz, 1H), 8.02 (s, 1H), 7.94 (dd, *J* = 7.9, 1.7 Hz, 1H), 7.84 (d, *J* = 8.6 Hz, 2H), 7.73 (s, 1H), 7.58 (d, *J* = 7.9 Hz, 1H), 7.43 (d, *J* = 8.6 Hz, 2H), 4.41 (dd, *J* = 8.2, 6.3 Hz, 1H), 3.35 – 3.32 (m, 2H), 2.61 (s, 3H), 2.43 (s, 5H), 1.67 (s, 3H), 1.43 (s, 9H).

LC–MS (ESI-TOF) Calcd for $C_{31}H_{32}ClN_6O_4S^+$ [M+H]$^+$: 619.19; found: 619.30.

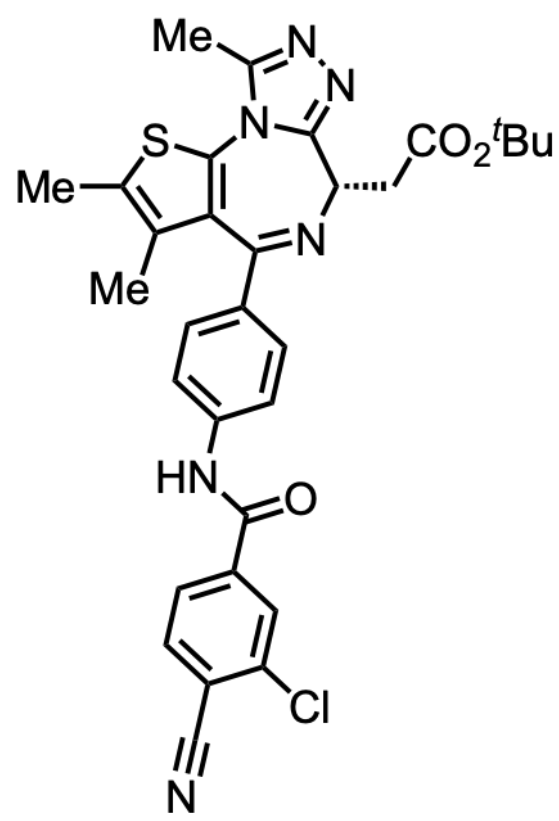

***tert*-butyl (*S*)-2-(4-(4-(3-chloro-4-cyanobenzamido)phenyl)-2,3,9-trimethyl-6*H*-thieno[3,2-*f*][1,2,4]triazolo[4,3-*a*][1,4]diazepin-6-yl)acetate (ZZ7-23-091; 45)**

Following General Procedure using JQ1-NH_2, purification by preparative RP-HPLC afforded the title compound (3.65 mg, 61% yield).

^{1}H NMR (500 MHz, DMSO-d_6) δ 10.73 (s, 1H), 8.26 (d, *J* = 1.6 Hz, 1H), 8.16 (d, *J* = 8.1 Hz, 1H), 8.04 (dd, *J* = 8.1, 1.7 Hz, 1H), 7.85 – 7.80 (m, 2H), 7.45 (d, *J* = 8.4 Hz, 2H), 4.41 (dd, *J* = 8.2, 6.3 Hz, 1H), 3.39 – 3.27 (m, 2H), 2.61 (s, 3H), 2.43 (s, 3H), 1.67 (s, 3H), 1.43 (s, 9H).

LC–MS (ESI-TOF) Calcd for $C_{31}H_{30}ClN_6O_3S^+$ $[M+H]^+$: 601.18; found: 601.25.

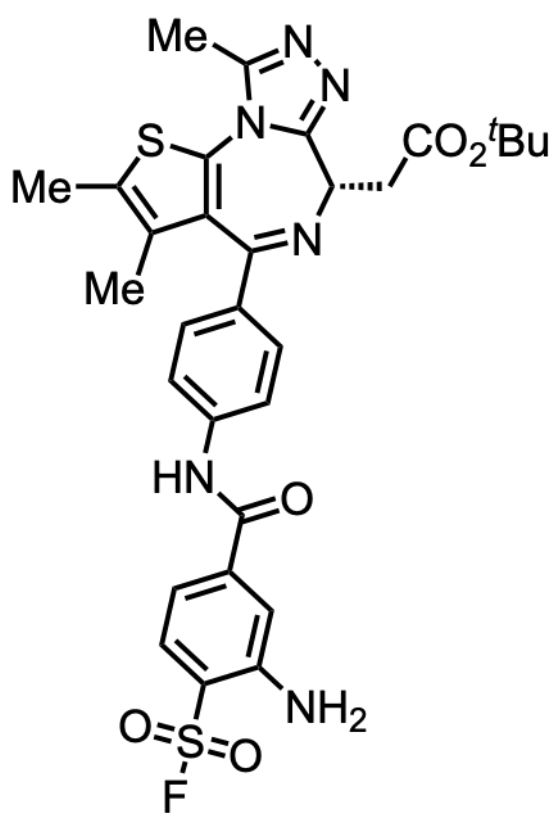

***tert*-butyl (*S*)-2-(4-(4-(3-amino-4-(fluorosulfonyl)benzamido)phenyl)-2,3,9-trimethyl-6*H*-thieno[3,2-*f*][1,2,4]triazolo[4,3-*a*][1,4]diazepin-6-yl)acetate (ZZ7-20-072; 46)**

Following General Procedure using JQ1-NH_2, purification by preparative RP-HPLC afforded the title compound (1.20 mg, 19% yield).

^{1}H NMR (500 MHz, DMSO-d_6) δ 10.62 (s, 1H), 7.80 (d, J = 8.3 Hz, 2H), 7.76 (d, J = 8.5 Hz, 1H), 7.48 – 7.37 (m, 3H), 7.15 (d, J = 8.5 Hz, 1H), 6.76 (s, 2H), 4.40 (t, J = 7.2 Hz, 1H), 3.36 – 3.27 (m, 2H), 2.60 (s, 3H), 2.43 (s, 3H), 1.67 (s, 3H), 1.43 (s, 9H).

LC–MS (ESI-TOF) Calcd for $C_{30}H_{32}FN_6O_5S_2^+$ $[M+H]^+$: 639.19; found: 639.20.

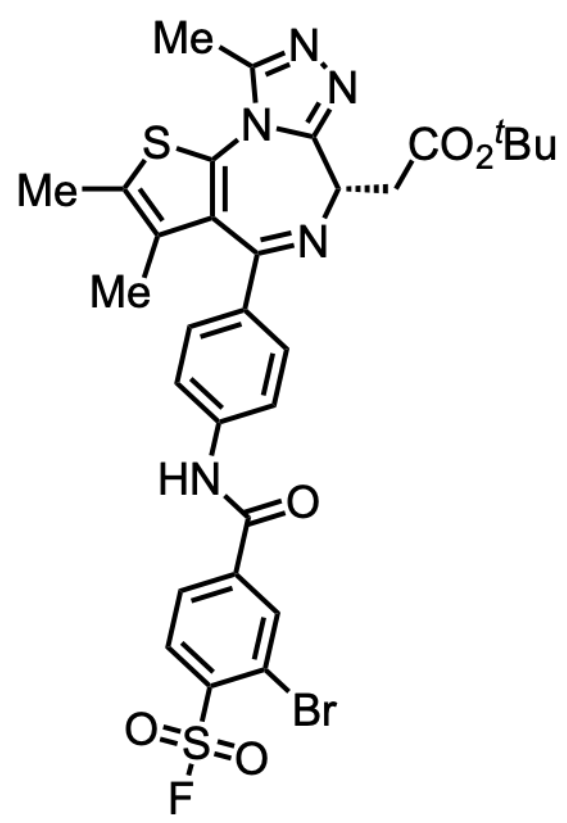

***tert*-butyl (*S*)-2-(4-(4-(3-bromo-4-(fluorosulfonyl)benzamido)phenyl)-2,3,9-trimethyl-6*H*-thieno[3,2-*f*][1,2,4]triazolo[4,3-*a*][1,4]diazepin-6-yl)acetate (ZZ7-20-090; 47)**

Following General Procedure using JQ1-NH_2, purification by preparative RP-HPLC afforded the title compound (0.83 mg, 12% yield).

^{1}H NMR (500 MHz, DMSO-d_6) δ 10.82 (s, 1H), 8.56 (d, *J* = 1.6 Hz, 1H), 8.38 (d, *J* = 8.3 Hz, 1H), 8.21 (d, *J* = 8.3 Hz, 1H), 7.83 (d, *J* = 8.5 Hz, 2H), 7.46 (d, *J* = 8.3 Hz, 2H), 4.41 (dd, *J* = 8.2, 6.3 Hz, 1H), 3.38 – 3.27 (m, 2H), 2.61 (s, 3H), 2.43 (s, 3H), 1.67 (s, 3H), 1.43 (s, 9H).

LC–MS (ESI-TOF) Calcd for $C_{30}H_{30}BrFN_5O_5S_2^+$ $[M+H]^+$: 702.09; found: 704.15.

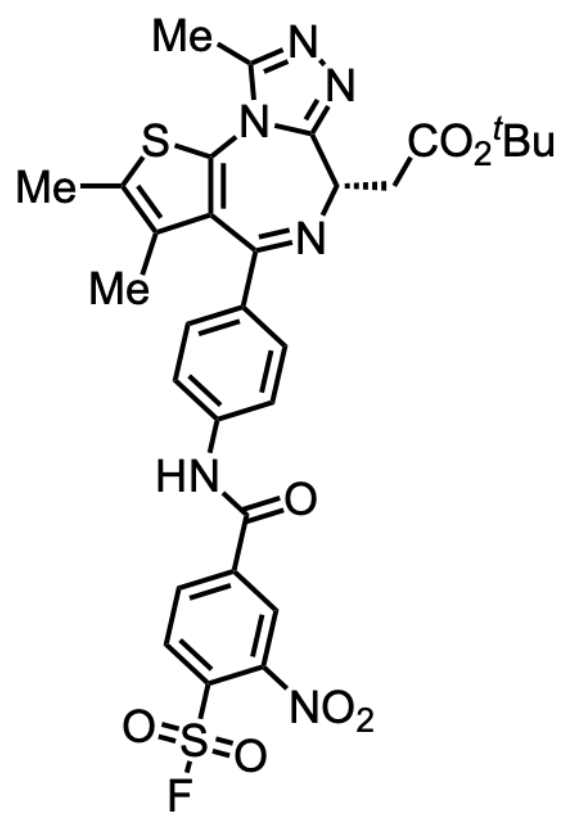

***tert*-butyl (*S*)-2-(4-(4-(4-(fluorosulfonyl)-3-nitrobenzamido)phenyl)-2,3,9-trimethyl-6*H*-thieno[3,2-*f*][1,2,4]triazolo[4,3-*a*][1,4]diazepin-6-yl)acetate (ZZ7-20-071; 48)**

Following General Procedure using JQ1-NH_2, purification by preparative RP-HPLC afforded the title compound (1.11 mg, 17% yield).

^{1}H NMR (500 MHz, DMSO-d_6) δ 10.98 (s, 1H), 8.82 (d, *J* = 1.7 Hz, 1H), 8.60 – 8.48 (m, 2H), 7.84 (d, *J* = 8.3 Hz, 2H), 7.47 (d, *J* = 8.3 Hz, 2H), 4.42 (dd, *J* = 8.2, 6.2 Hz, 1H), 3.40 – 3.26 (m, 2H), 2.61 (s, 3H), 2.43 (s, 3H), 1.67 (s, 3H), 1.43 (s, 9H).

LC–MS (ESI-TOF) Calcd for $C_{30}H_{30}FN_6O_7S_2^+$ $[M+H]^+$: 669.16; found: 669.25.

GSH chemical stability of ZZ1 and ZZ2.

Afatinib (Ctrl)	**Time (min)**	**Peak Area Ratio**	**%Remaining**	**Ln (%remaining)**	**$T_{1/2}$ (min)**
	0	3.41E-01	100.00	4.61	25.23
	5	2.80E-01	82.11	4.41	
	15	1.64E-01	48.09	3.87	
	30	9.41E-02	27.60	3.32	
	60	4.71E-02	13.81	2.63	
	120	1.17E-02	3.43	1.23	

**ZZ1**	**Time (min)**	**Peak Area Ratio**	**%Remaining**	**Ln (%remaining)**	**$T_{1/2}$ (min)**
	0	6.26E-01	100.00	4.61	<5
	5	1.67E-01	26.68	3.28	
	15	5.86E-02	9.36	2.24	
	30	2.01E-02	3.21	1.17	
	60	1.95E-02	3.12	1.14	
	120	1.42E-02	2.27	0.82	

**ZZ2**	**Time (min)**	**Peak Area Ratio**	**%Remaining**	**Ln (%remaining)**	**$T_{1/2}$ (min)**
	0	1.51E-01	100.00	4.61	<5
	5	1.40E-02	9.27	2.23	
	15	7.12E-03	4.72	1.55	
	30	0.00E+00	0.00		
	60	0.00E+00	0.00		
	120	0.00E+00	0.00		

Reference

1. Blake, R. A. et al. Preparation of tert-Butyl(S)-2-(4-(Phenyl)-6H-thieno[3,2-f][1,2,4]triazolo[4,3-a][1,4]-diazepin-6-yl)acetate Derivatives and Related Compounds as Bromodomain BRD4 Inhibitors for the Treatment of Cancer. WO2020055976 (2020).
2. Mukherjee, H. et al. A study of the reactivity of S(VI)–F containing warheads with nucleophilic amino-acid side chains under physiological conditions. *Org Biomol Chem* **15**, 9685-9695 (2017).
3. Wang, L. et al. MicroRNA detection in biologically relevant media using a split aptamer platform. *Bioorganic & Medicinal Chemistry* **69**, 116909 (2022).
4. Gray, N. S. et al. Preparation of triazolyl derivatives - bifunctional compounds as degraders that target proteins via KEAP1. WO2020018788 (2020).
5. Salvino, J. et al. Preparation of pyrimidine derivatives as proteolysis-targeting chimeras (PROTACs). WO2020092662 (2020).
6. Zhuang, Z.*, Byun, W. S.* et al. Discovery of electrophilic degraders that exploit SNAr chemistry. *bioRxiv* (2024).
7. Li, Z. et al. Development and Characterization of a Wee1 Kinase Degrader. *Cell Chem Biol* **27**, 57-65.e9 (2020).

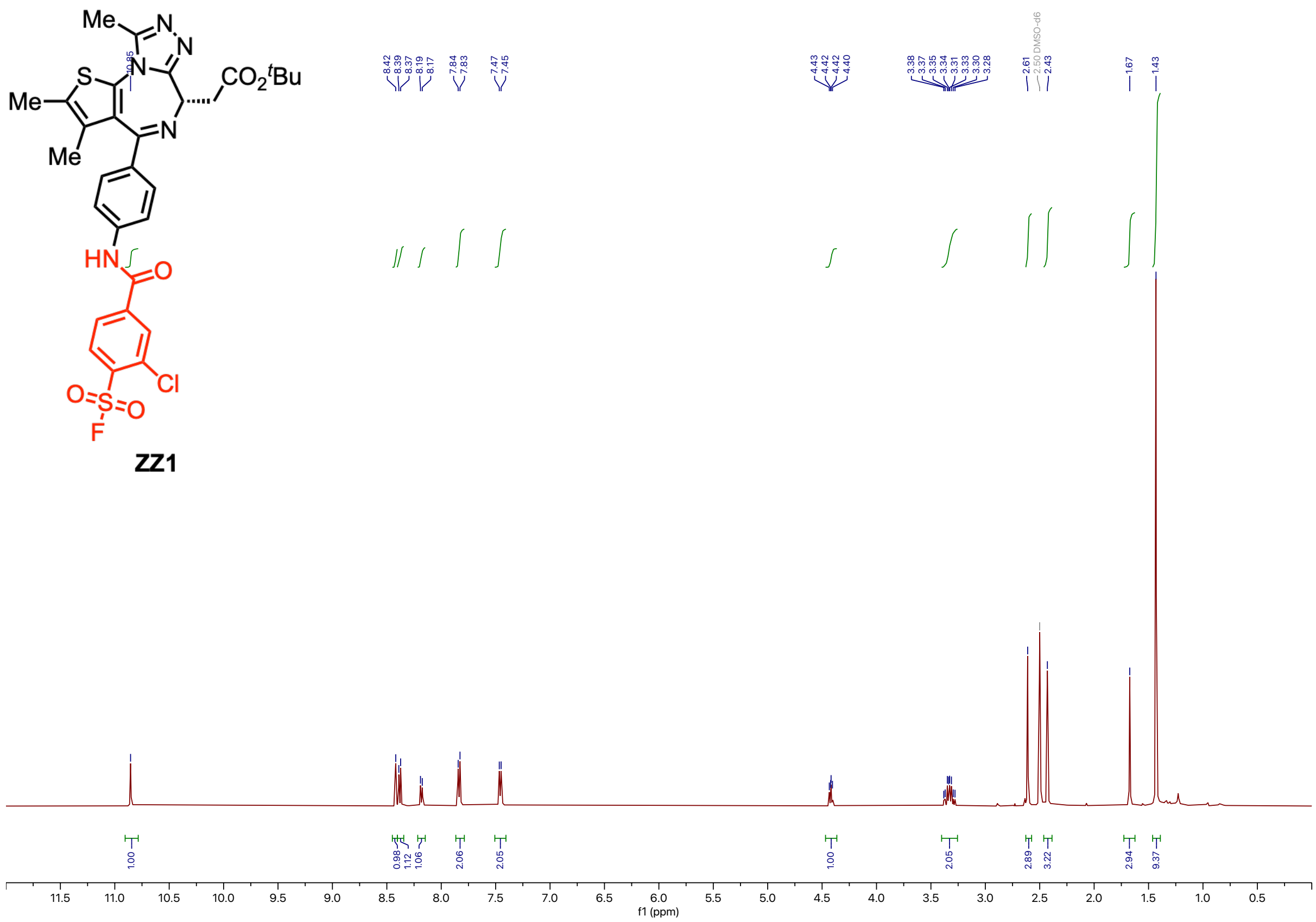

ZZ1
10.85
8.42 8.39 8.37 8.19 8.17 7.84 7.83 7.47 7.45
4.43 4.42 4.42 4.40
3.38 3.37 3.35 3.34 3.31 3.33 3.30 3.28
2.61 2.50 DMSO-d6 2.43
1.67 1.43
1.00 0.98 1.12 1.06 2.06 2.05 1.00 2.05 2.89 3.22 2.94 9.37
f1 (ppm)

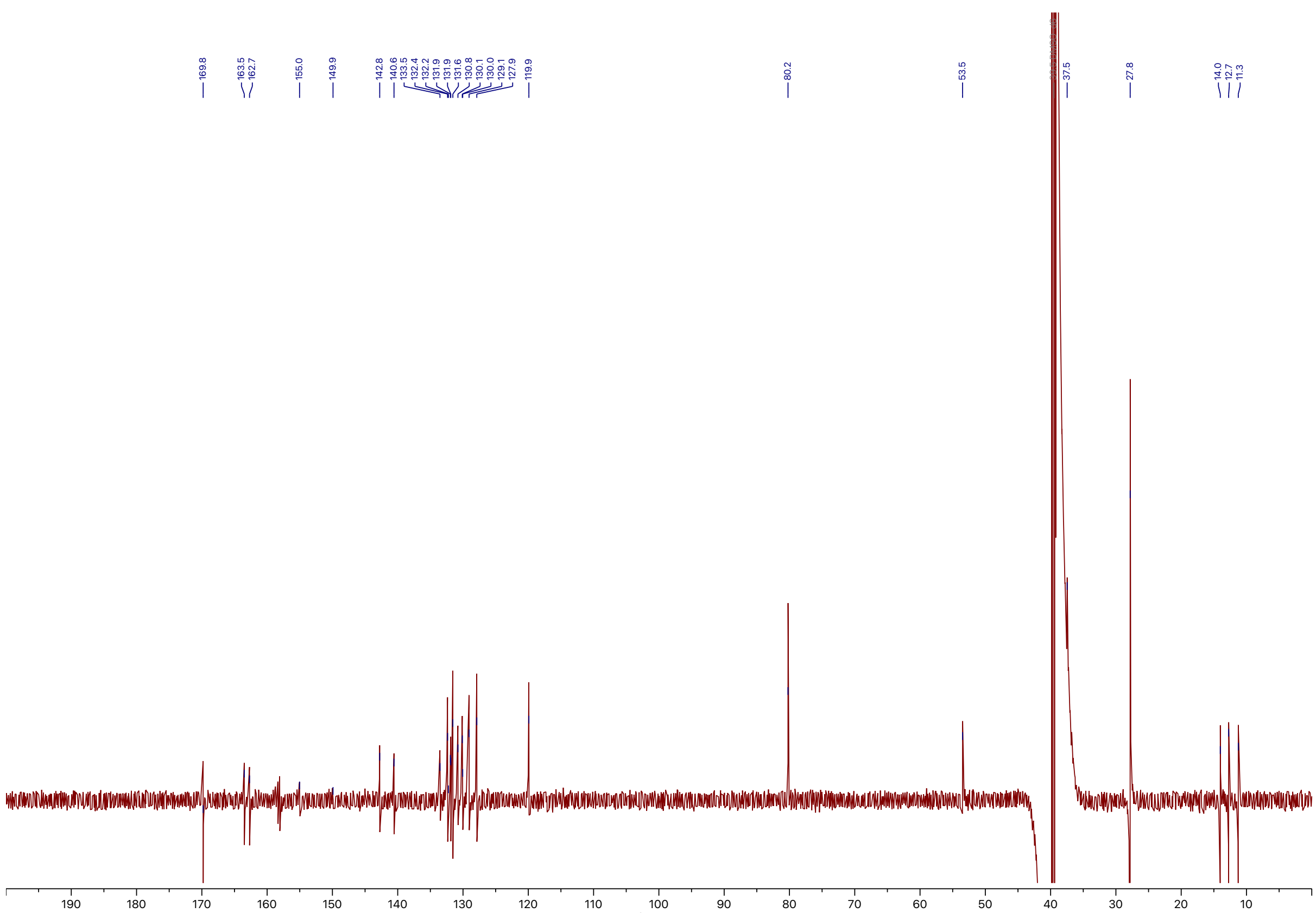
169.8 163.5 162.7 155.0 149.9 142.8 140.6 133.5 132.4 132.2 131.9 131.9 131.6 130.8 130.1 130.0 129.1 127.9 119.9
80.2
53.5
37.5
27.8
14.0 12.7 11.3
f1 (ppm)

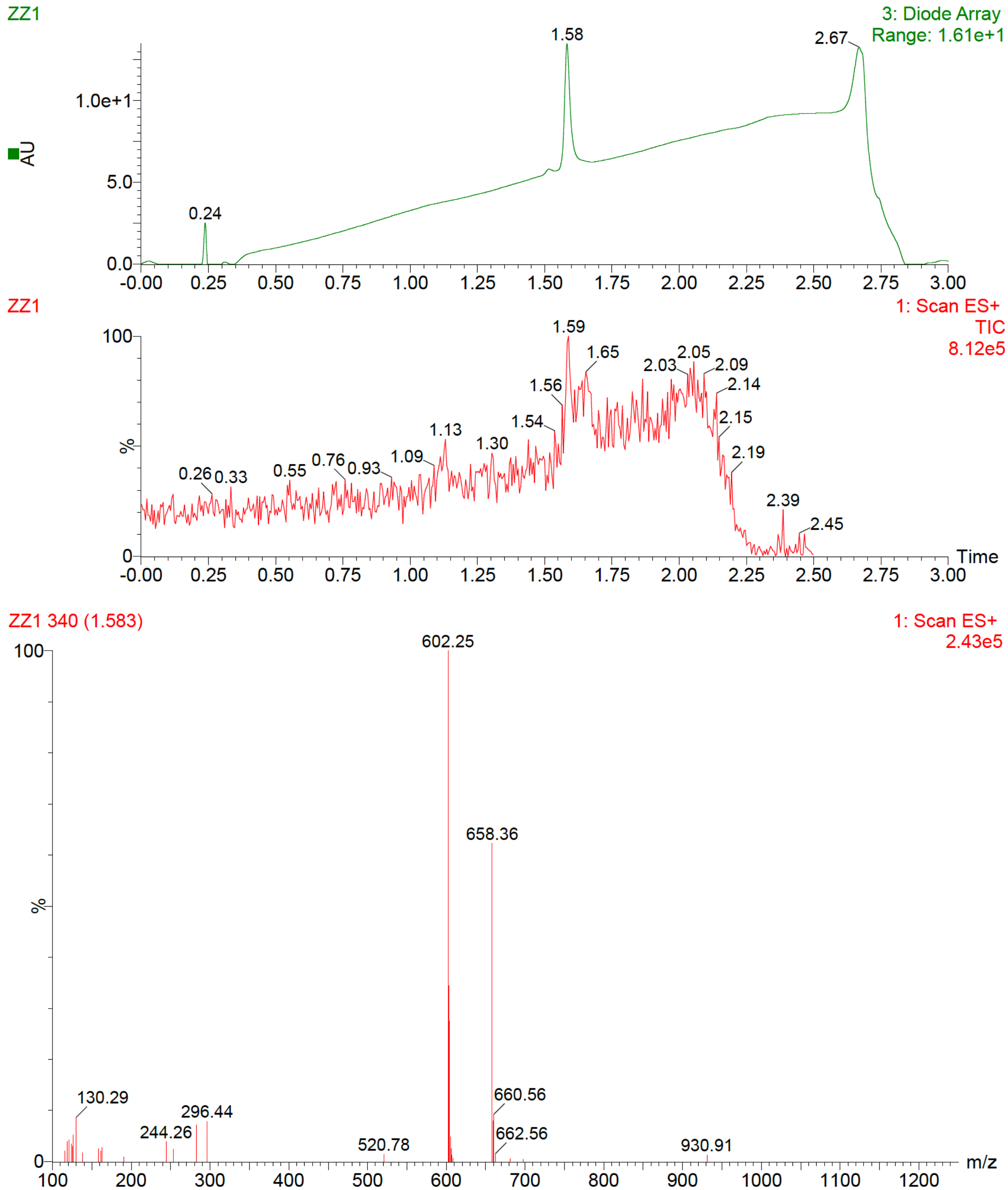
ZZ1
3: Diode Array
Range: 1.61e+1
AU
1.58
2.67
0.24
ZZ1
1: Scan ES+
TIC
8.12e5
1.59
1.65
2.03
2.05
2.09
2.14
1.56
1.54
2.15
1.13
1.30
2.19
1.09
0.26
0.33
0.55
0.76
0.93
2.39
2.45
Time
ZZ1 340 (1.583)
1: Scan ES+
2.43e5
602.25
658.36
660.56
662.56
130.29
296.44
244.26
520.78
930.91
m/z

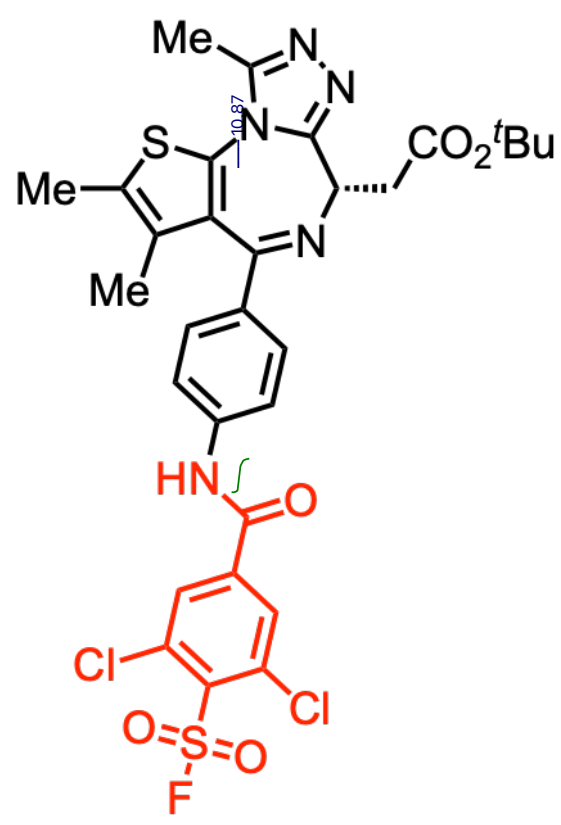
Me
N
N
S
N
CO2tBu
Me
N
Me
HN
O
Cl
Cl
O
S
O
F

ZZ2

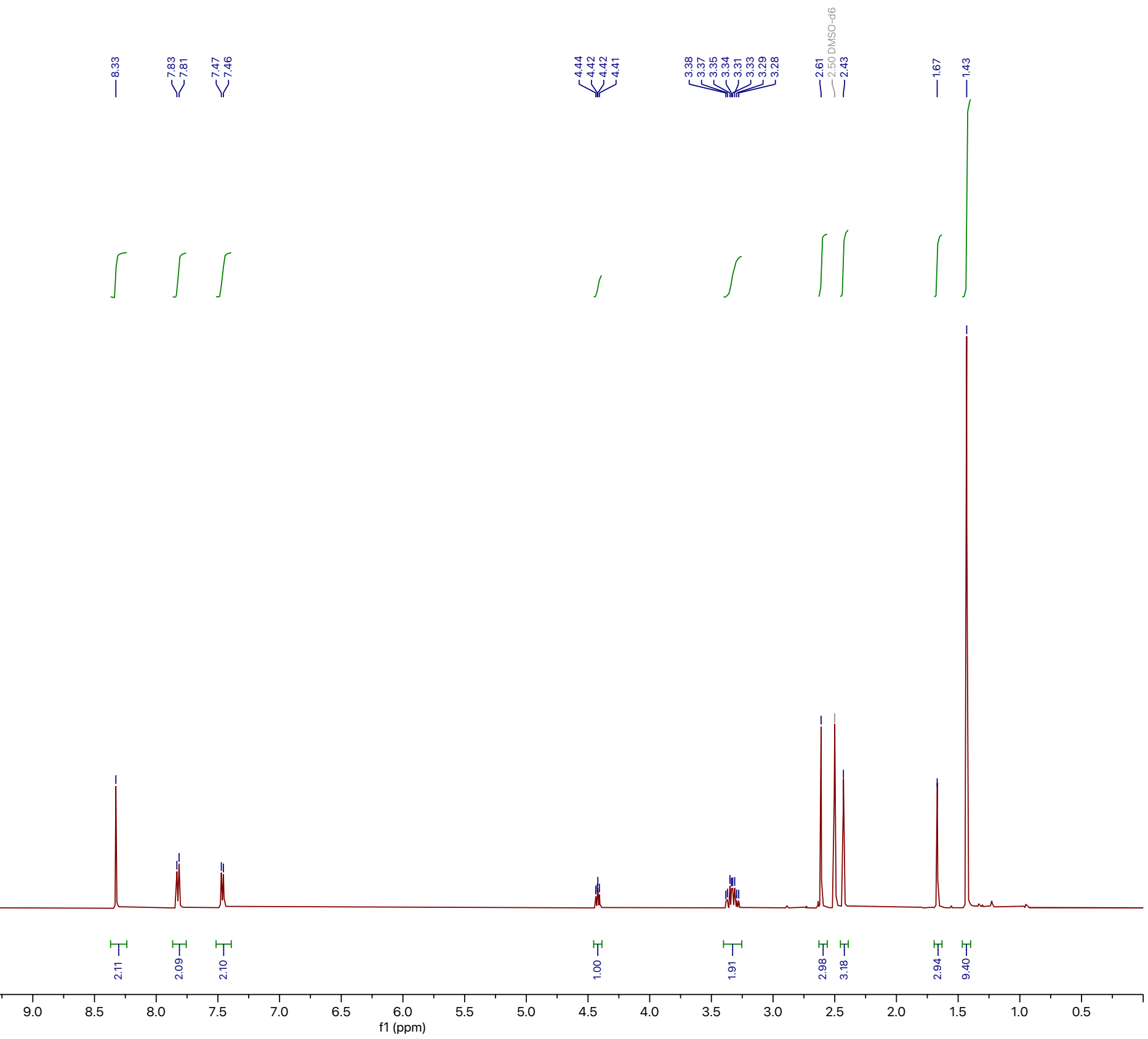
8.33
7.83
7.81
7.47
7.46
4.44
4.42
4.42
4.41
3.38
3.37
3.35
3.34
3.31
3.33
3.29
3.28
2.61
2.50 DMSO-d6
2.43
1.67
1.43
0.98
2.11
2.09
2.10
1.00
1.91
2.98
3.18
2.94
9.40
f1 (ppm)

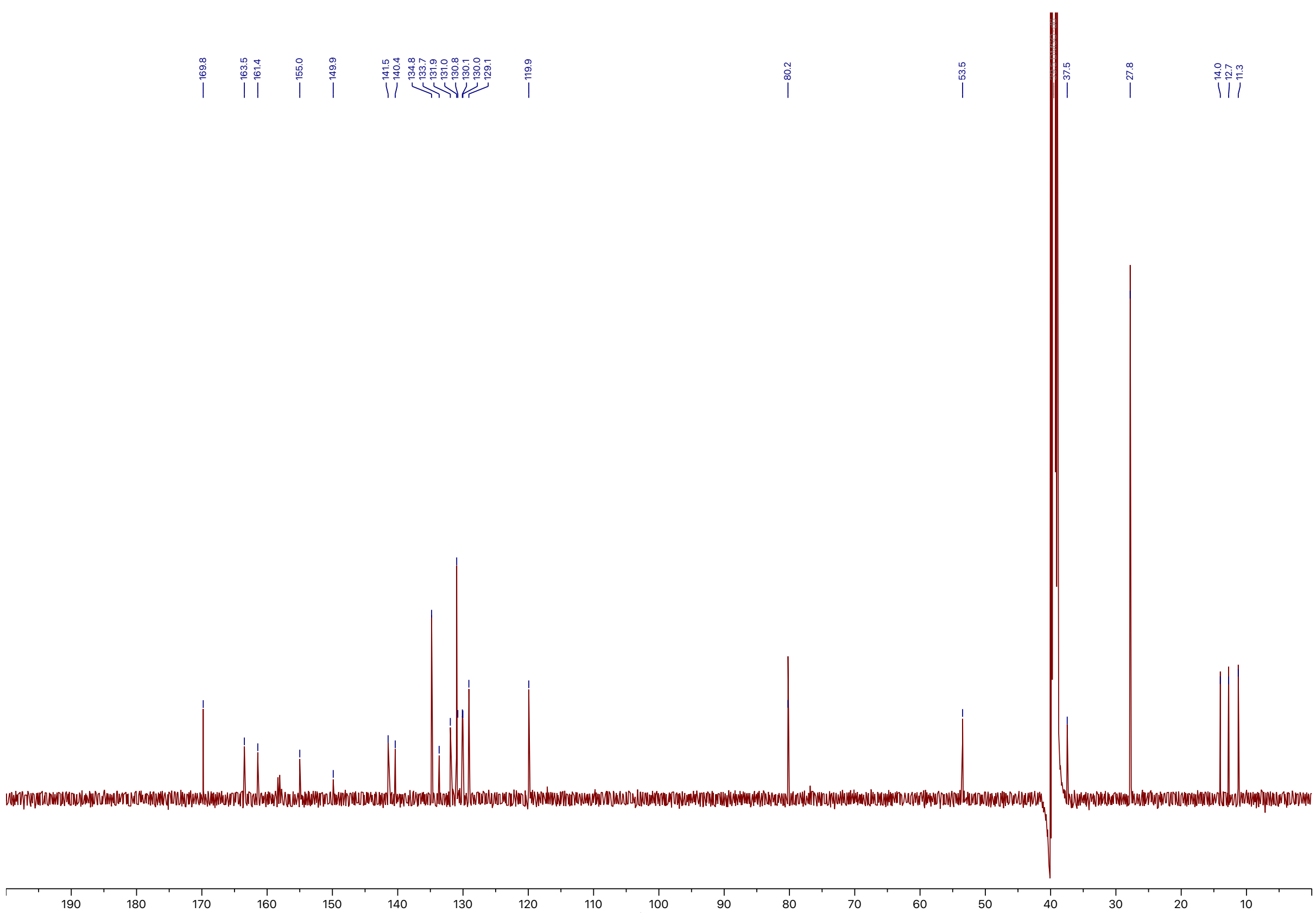
169.8
163.5
161.4
155.0
149.9
141.5
140.4
134.8
133.7
131.9
131.0
130.8
130.1
130.0
129.1
119.9
80.2
53.5
37.5
27.8
14.0
12.7
11.3
f1 (ppm)

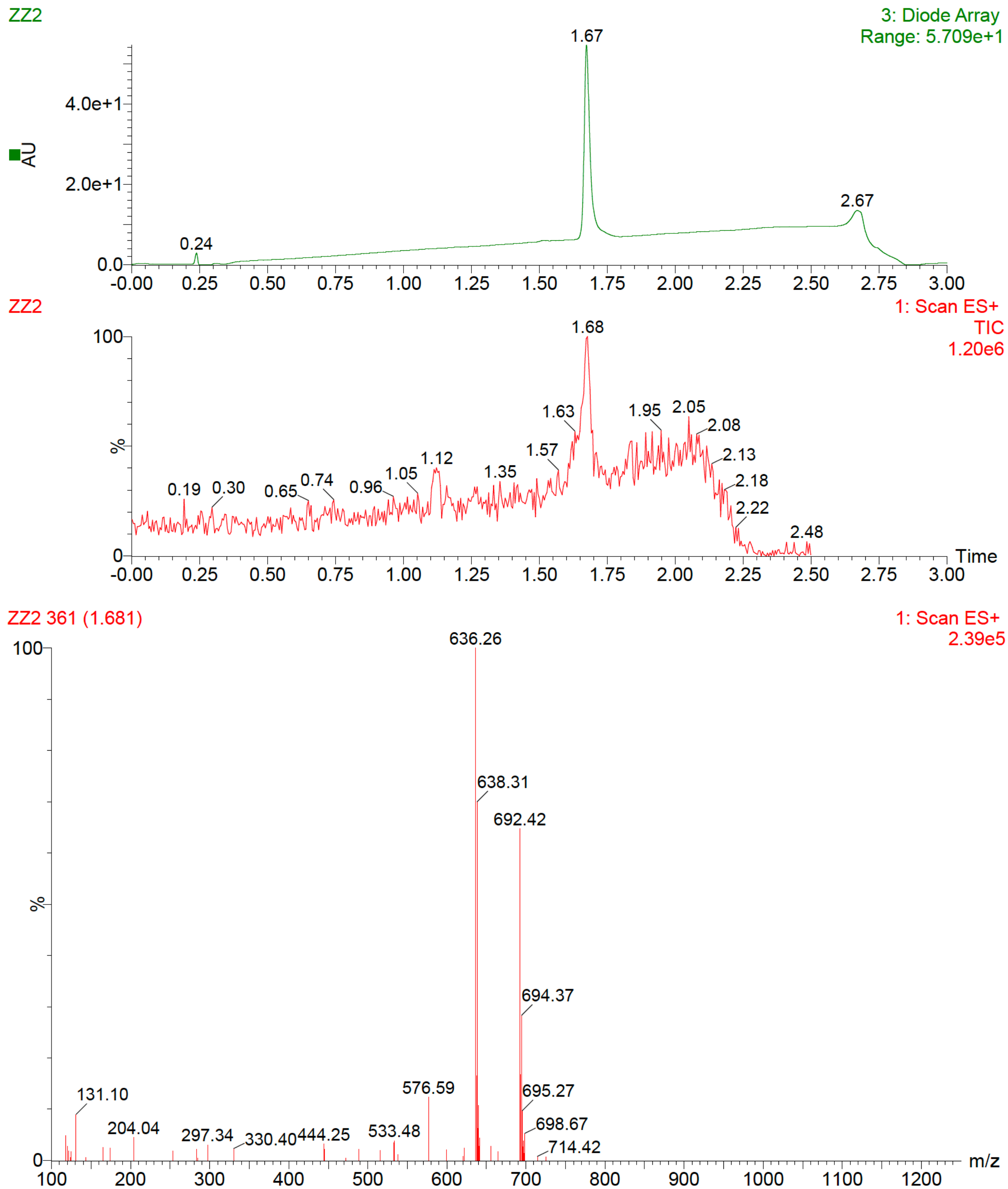
ZZ2
3: Diode Array
Range: 5.709e+1
AU
1.67
0.24
2.67
ZZ2
1: Scan ES+
TIC
1.20e6
1.68
1.63
1.95
2.05
2.08
1.57
2.13
1.12
1.35
1.05
2.18
0.19
0.30
0.65
0.74
0.96
2.22
2.48
Time
ZZ2 361 (1.681)
1: Scan ES+
2.39e5
636.26
638.31
692.42
694.37
576.59
695.27
131.10
204.04
297.34
330.40
444.25
533.48
698.67
714.42
m/z

Uncropped Blot for Supplementary Figure 3b

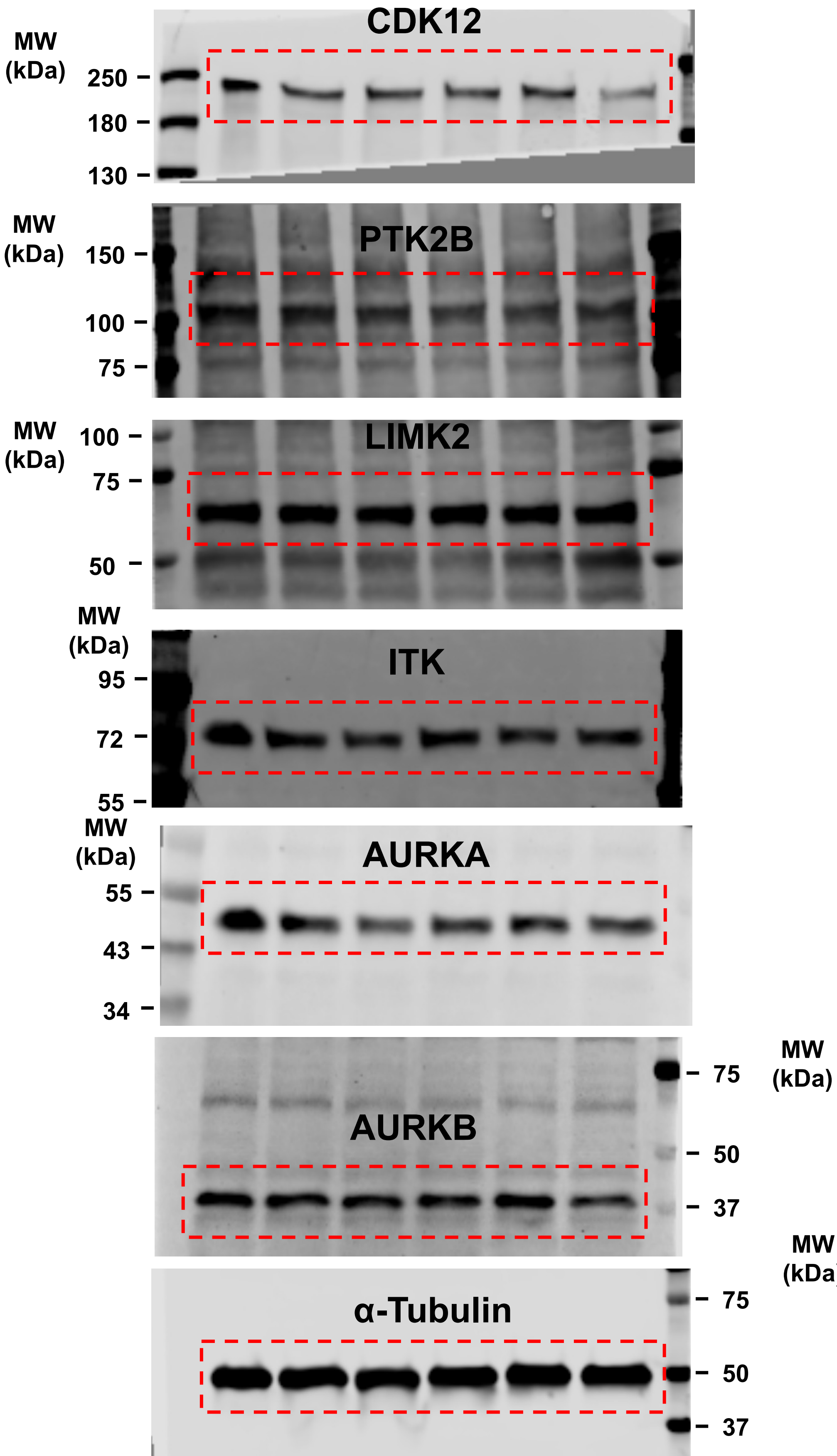

Uncropped Blot for Supplementary Figure 3b

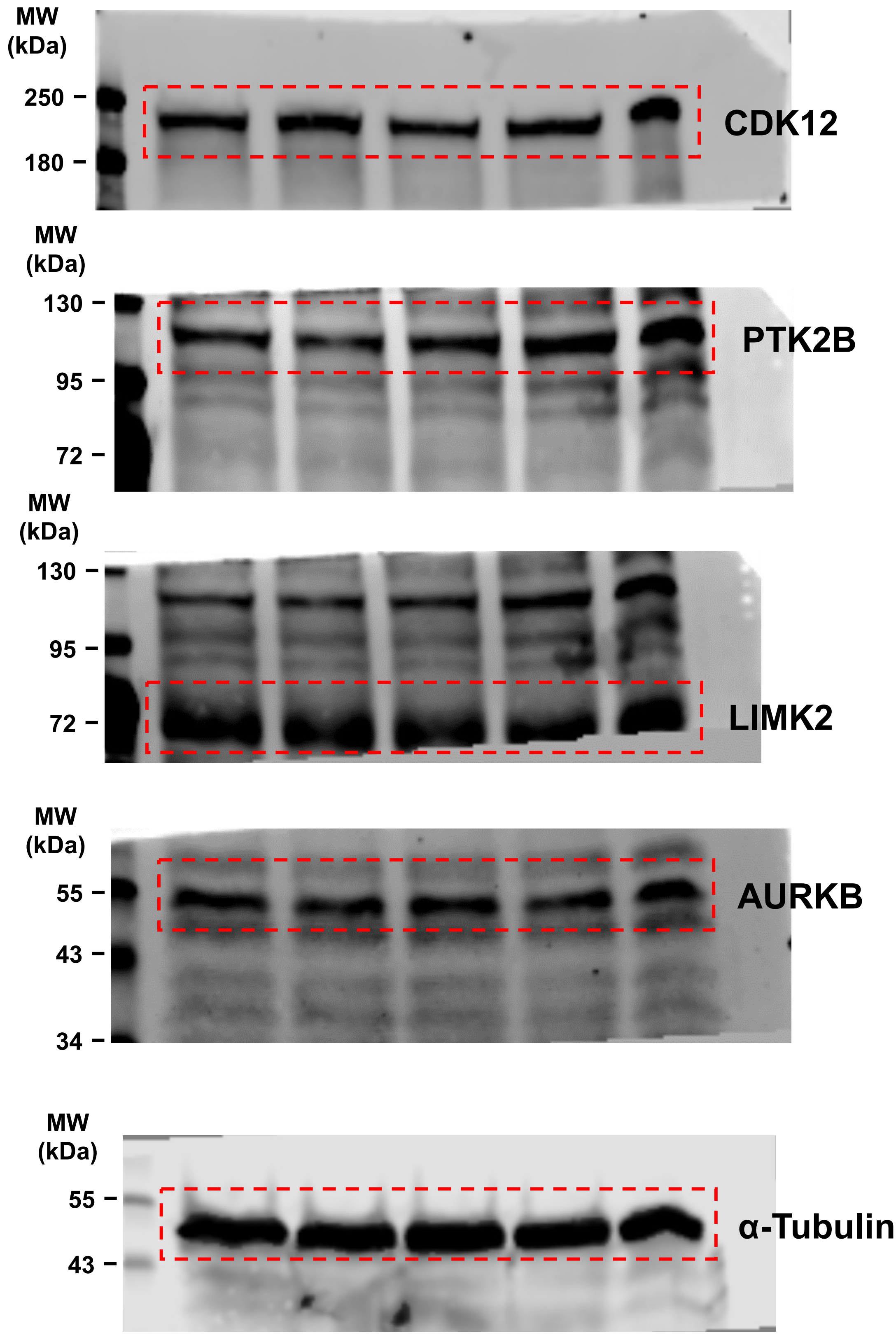

Uncropped Blot for Supplementary Figure 3d

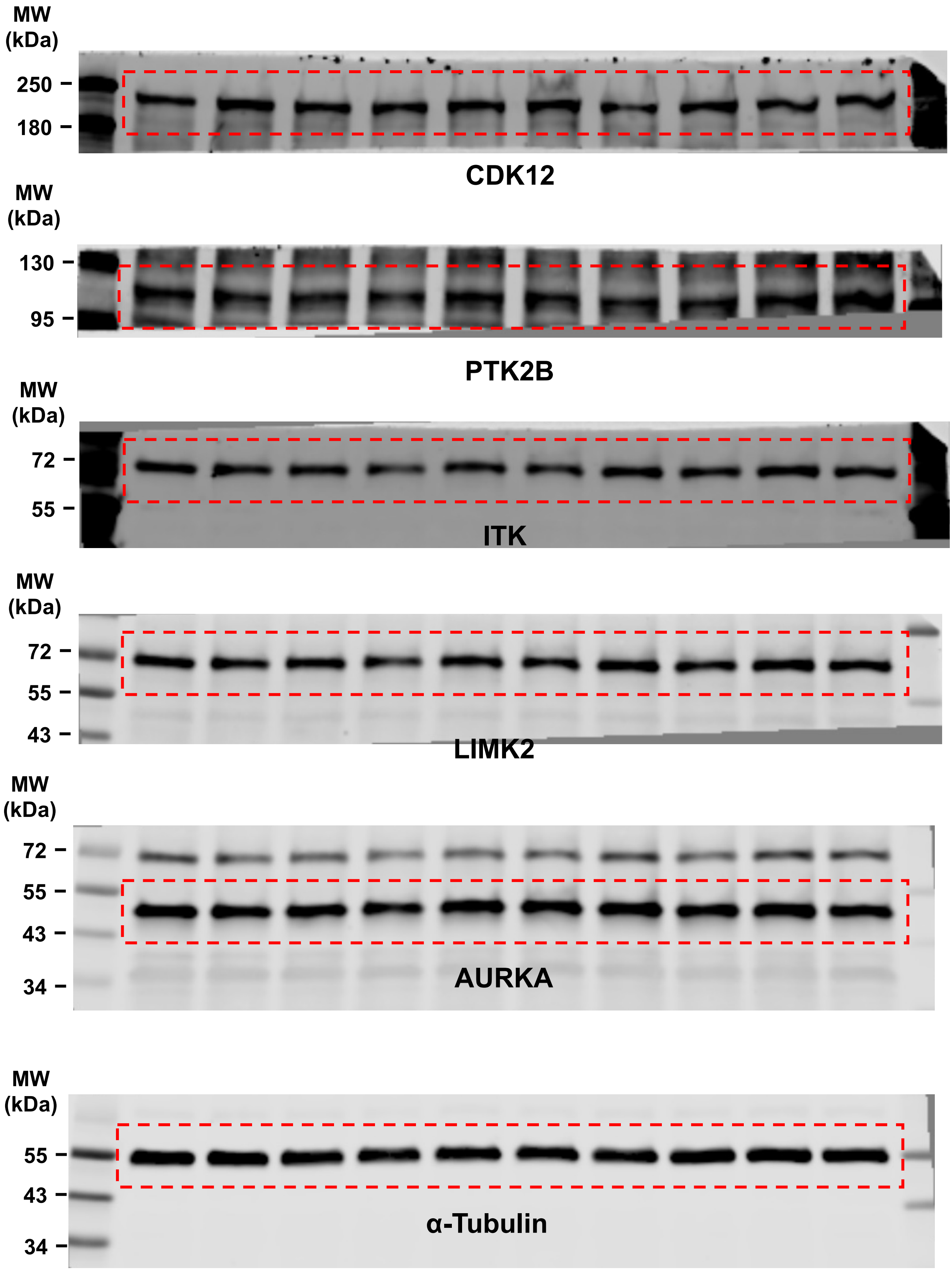

Uncropped Blot for Supplementary Figure 3d

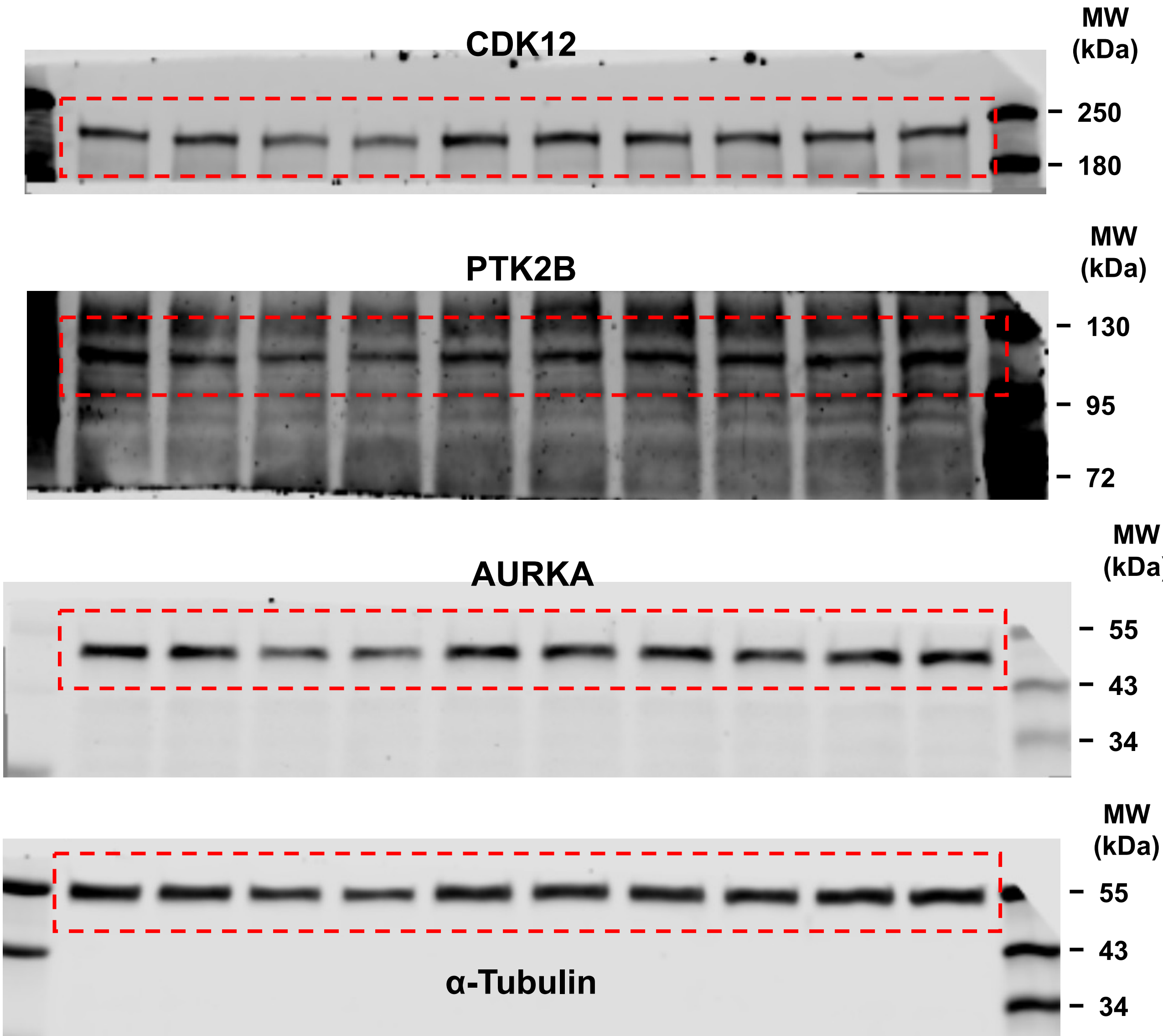

Uncropped Blot for Supplementary Figure 4b

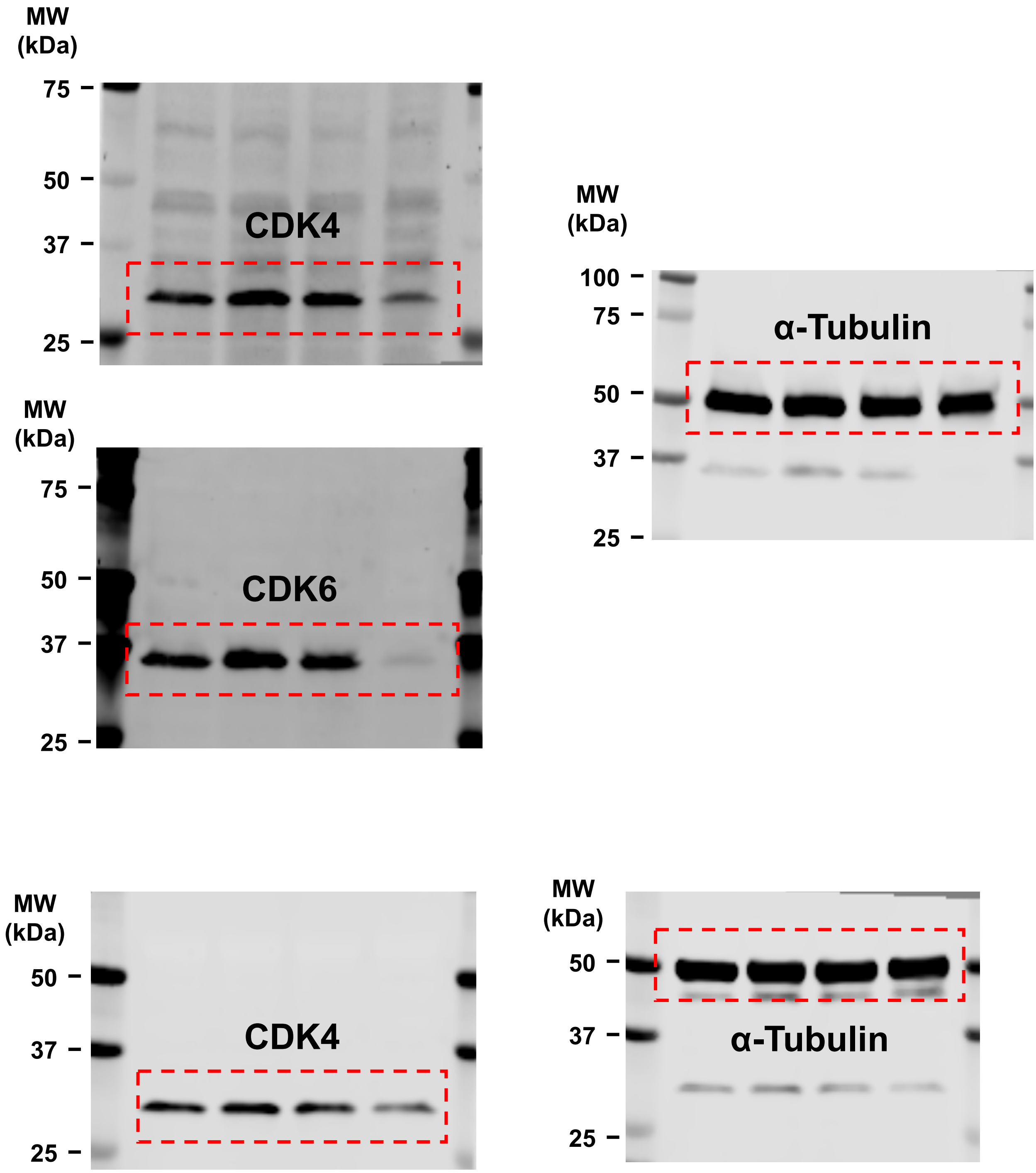

Uncropped Blot for Supplementary Figure 4d

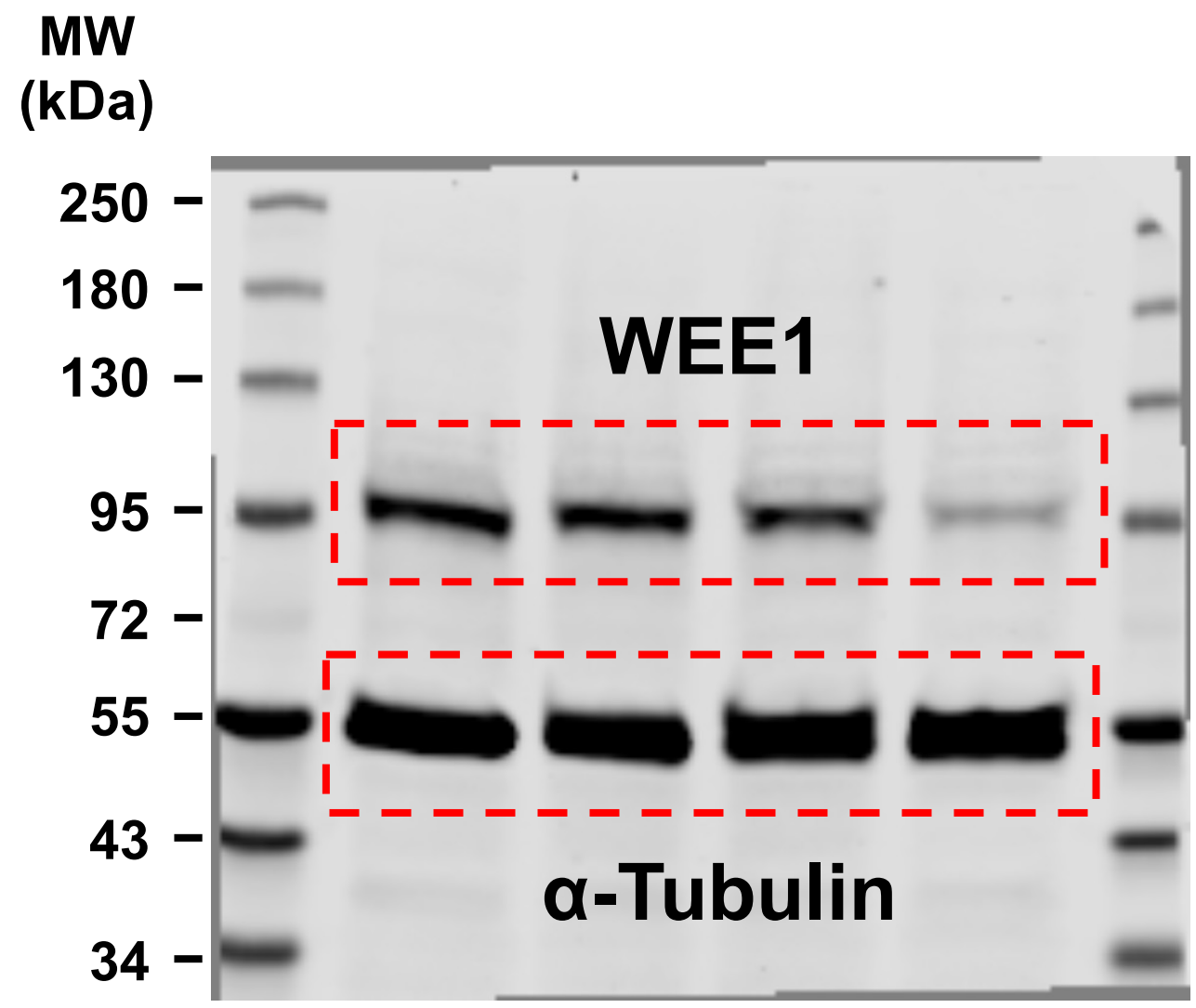

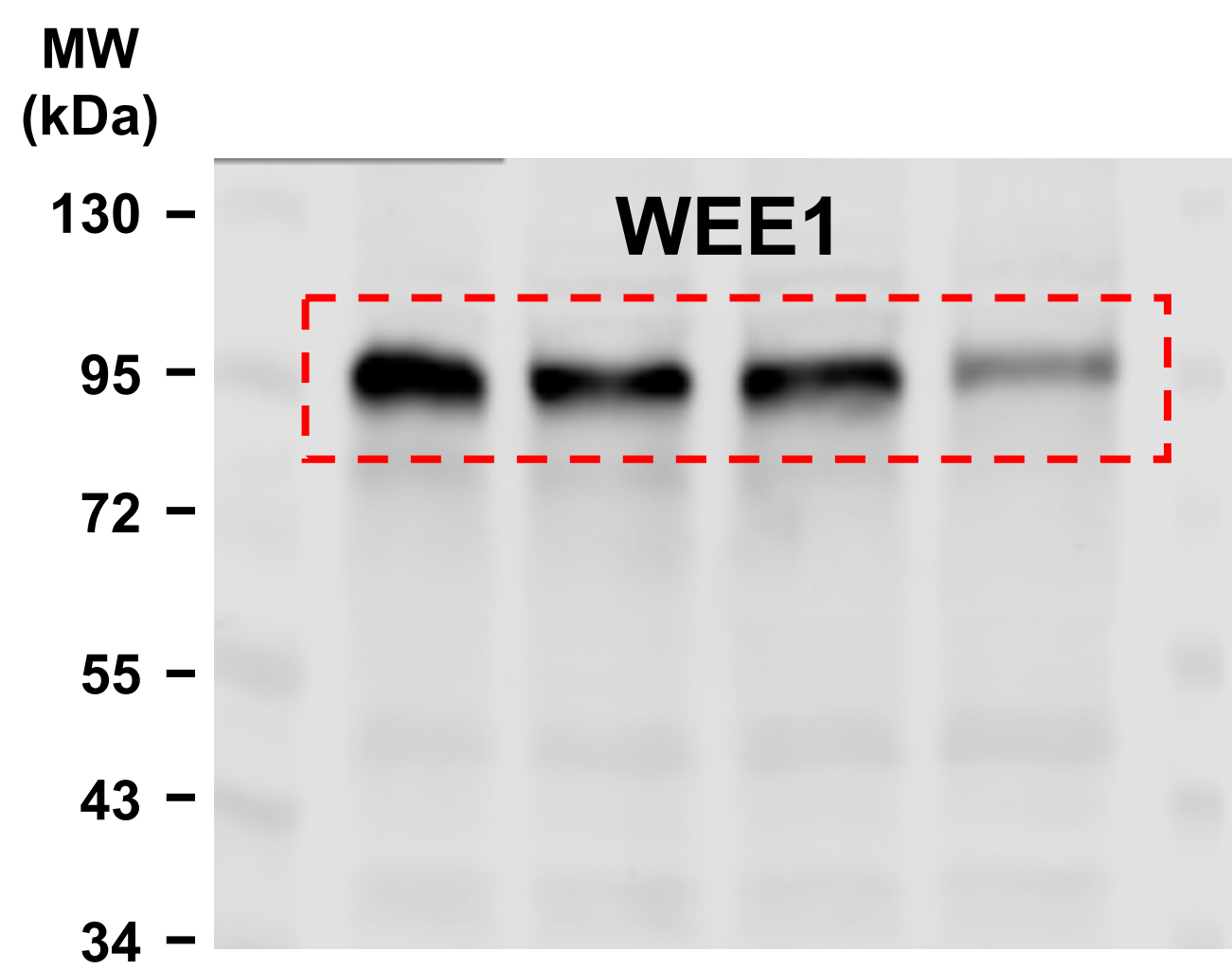

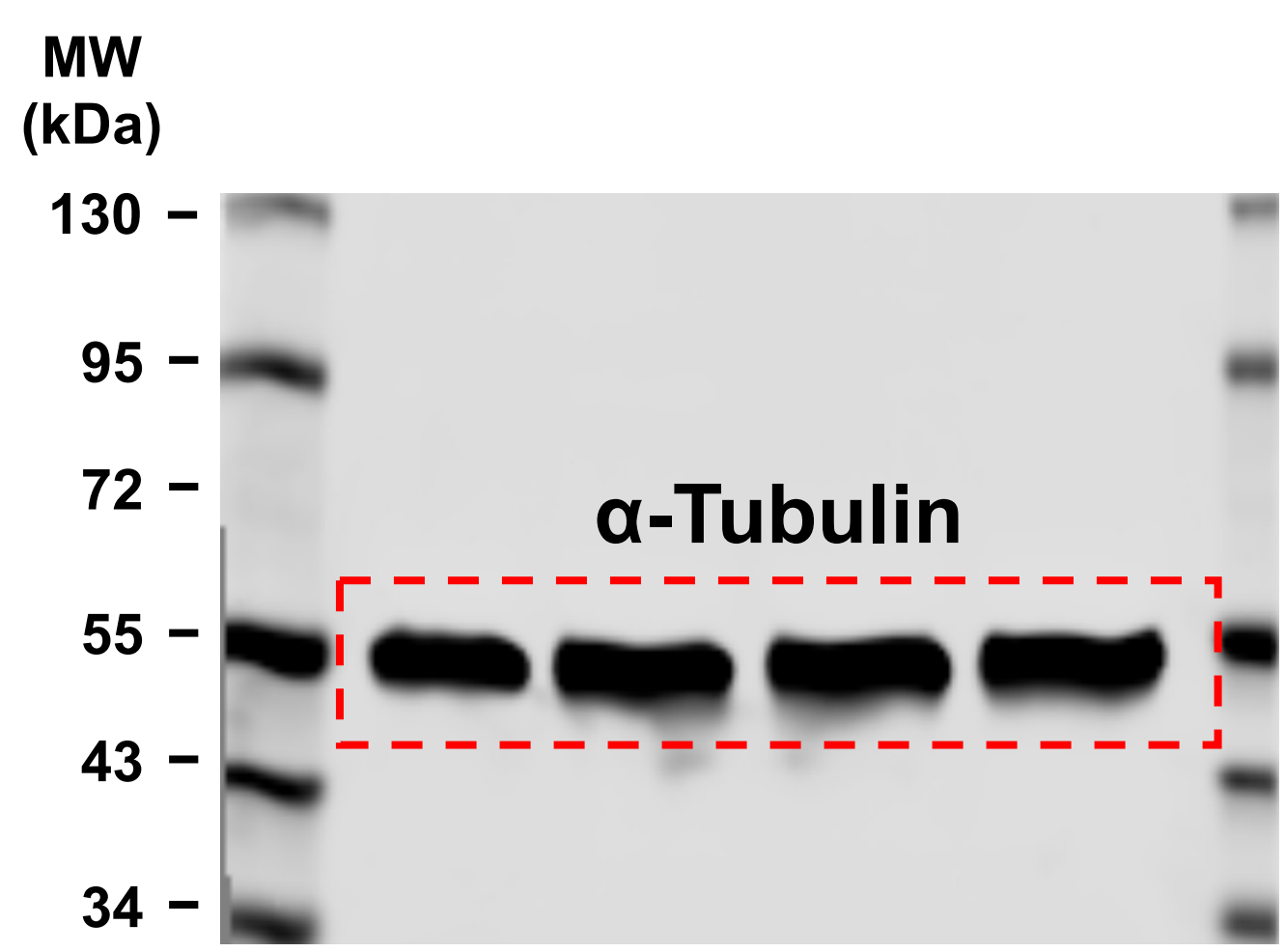

Supplement: Supplementary file 1 [file 41589_2026_2182_MOESM1_ESM.pdf]
