# Supplementary material for: Charged molecular glue discovery enabled by targeted degron display
Source: Nat Chem Biol. 2026 Apr 6;22(9):1446–55. doi: 10.1038/s41589-026-02182-5 (PMC13355314; doi:10.1038/s41589-026-02182-5)
Supplement: Supplementary file 3 [file 41589_2026_2182_MOESM3_ESM.pdf]

Charged Molecular Glue Discovery Enabled by Targeted Degron Display

Uncropped Image Data

Uncropped fluorescent scans of SDS-PAGE gels for Figure 1e

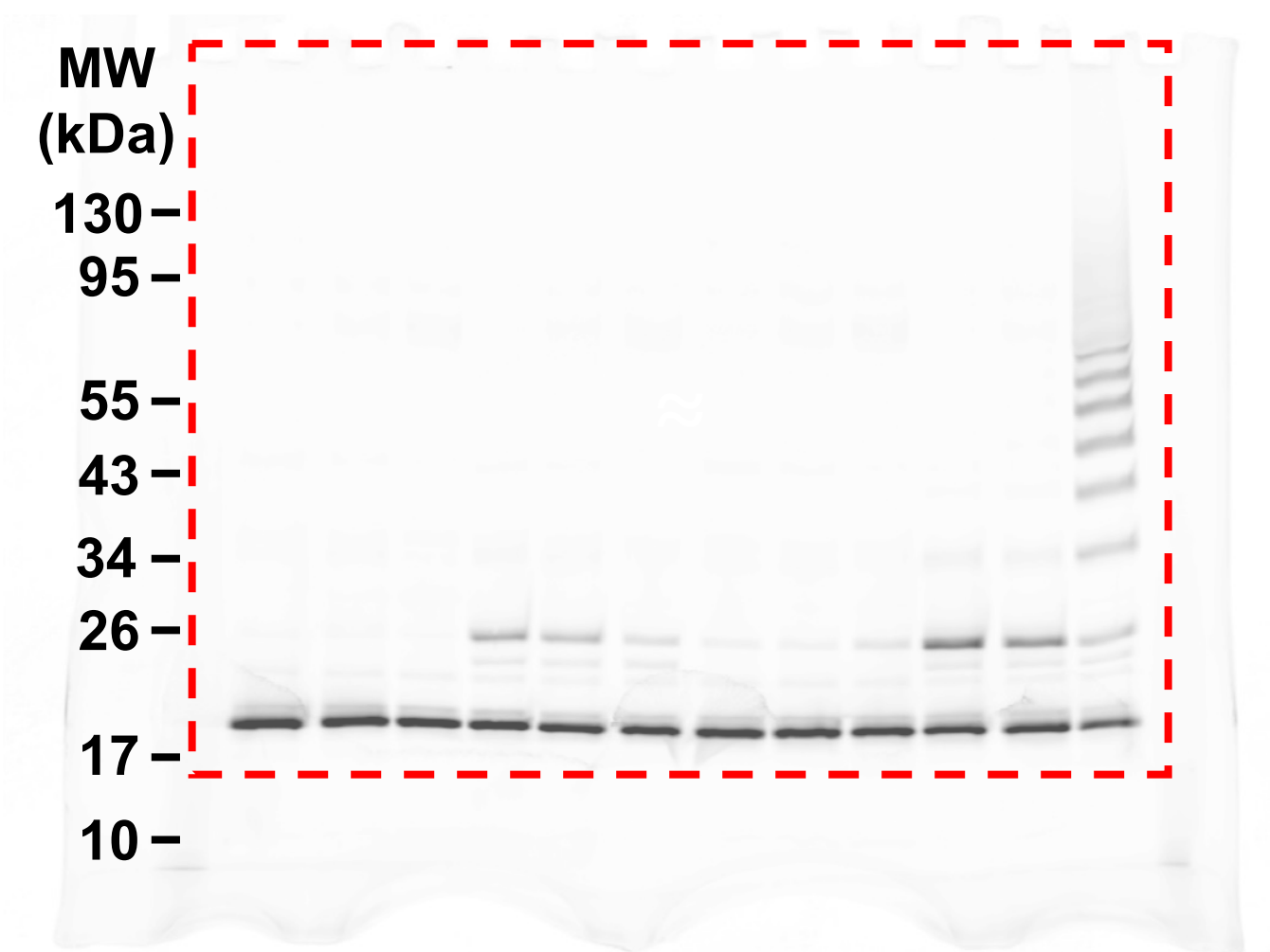

Cy2 scan (visualizes FAM-BRD4$_{BD1}$)

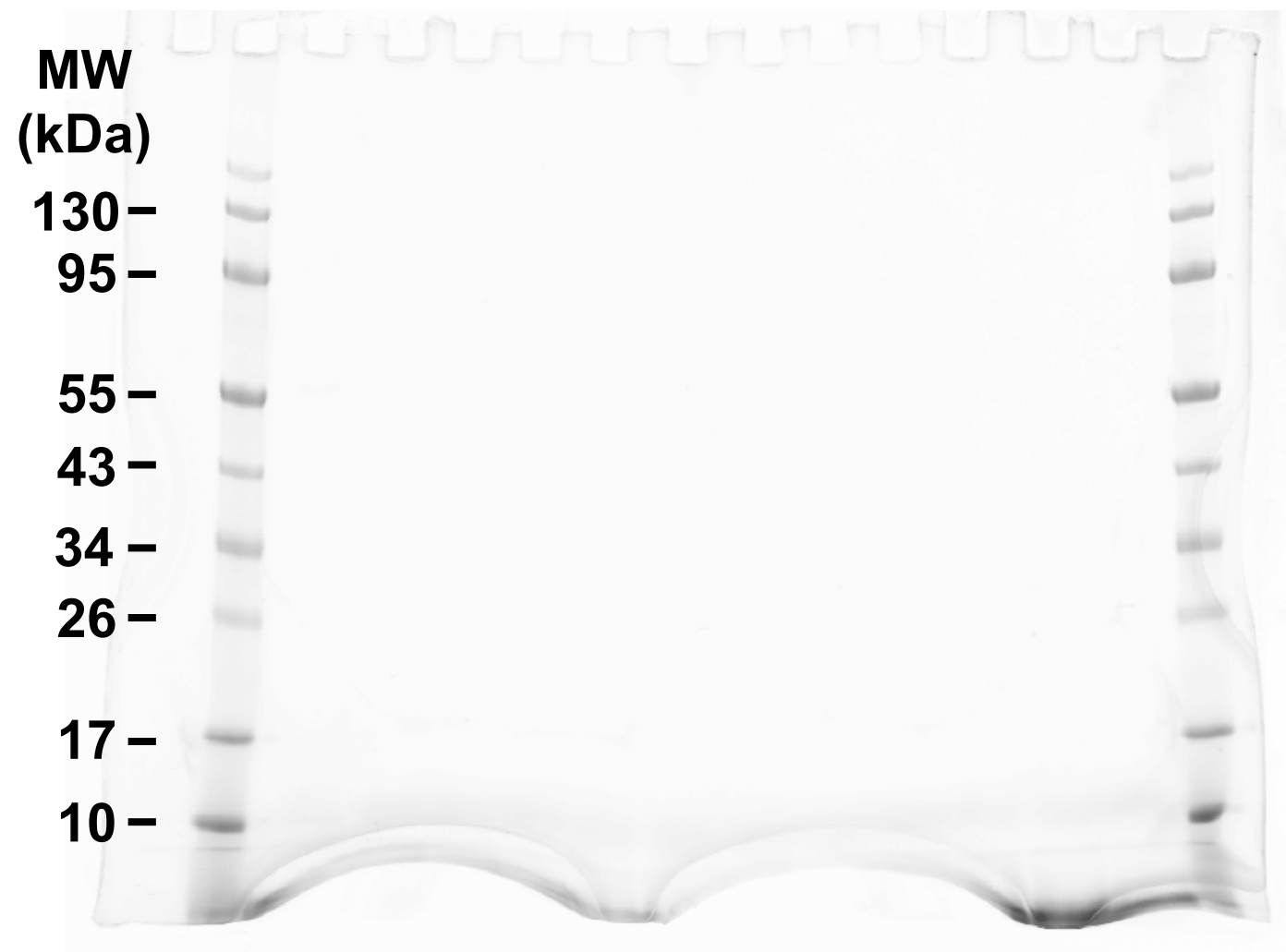

Cy5 scan (visualizes MW marker)

Uncropped fluorescent scans of SDS-PAGE gels for Figure 1f

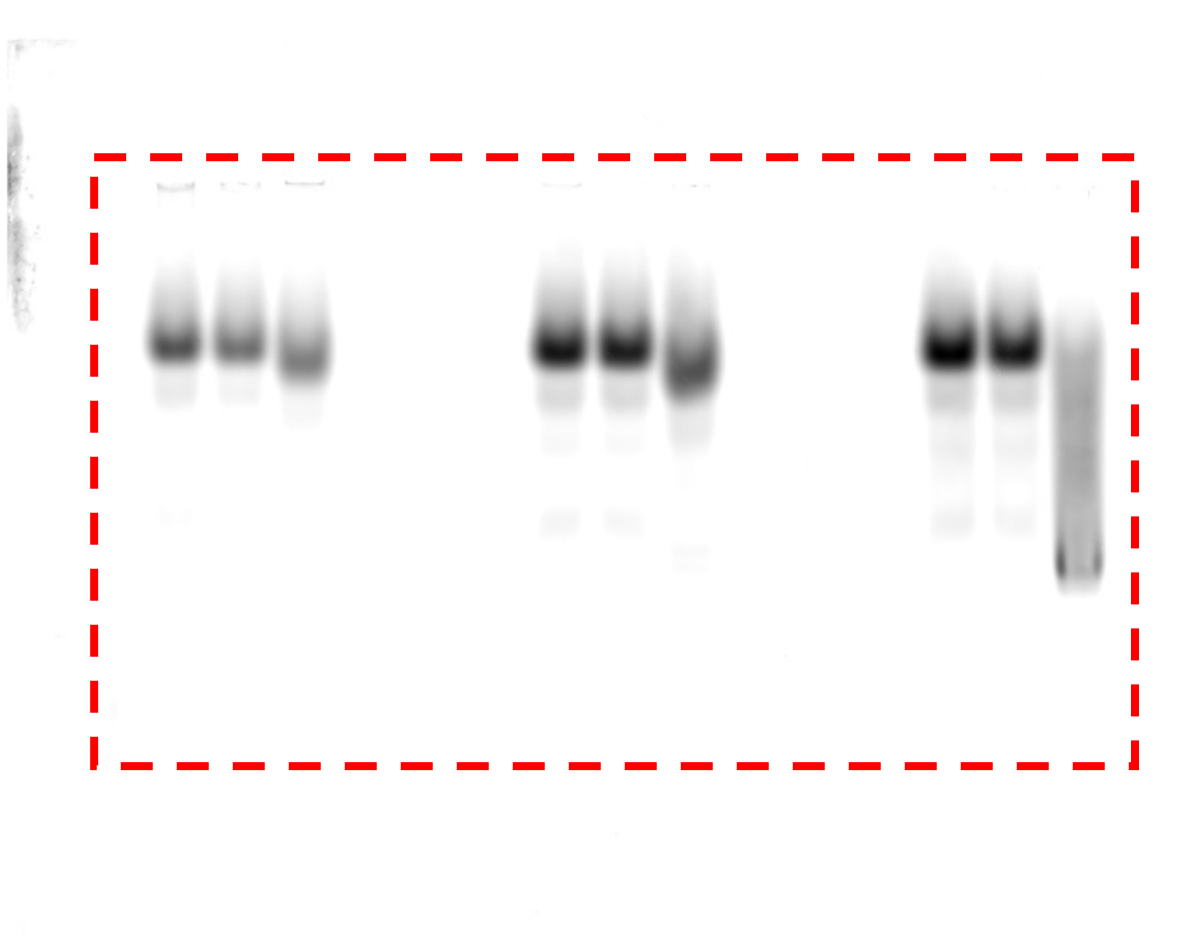

same gel, different contrast

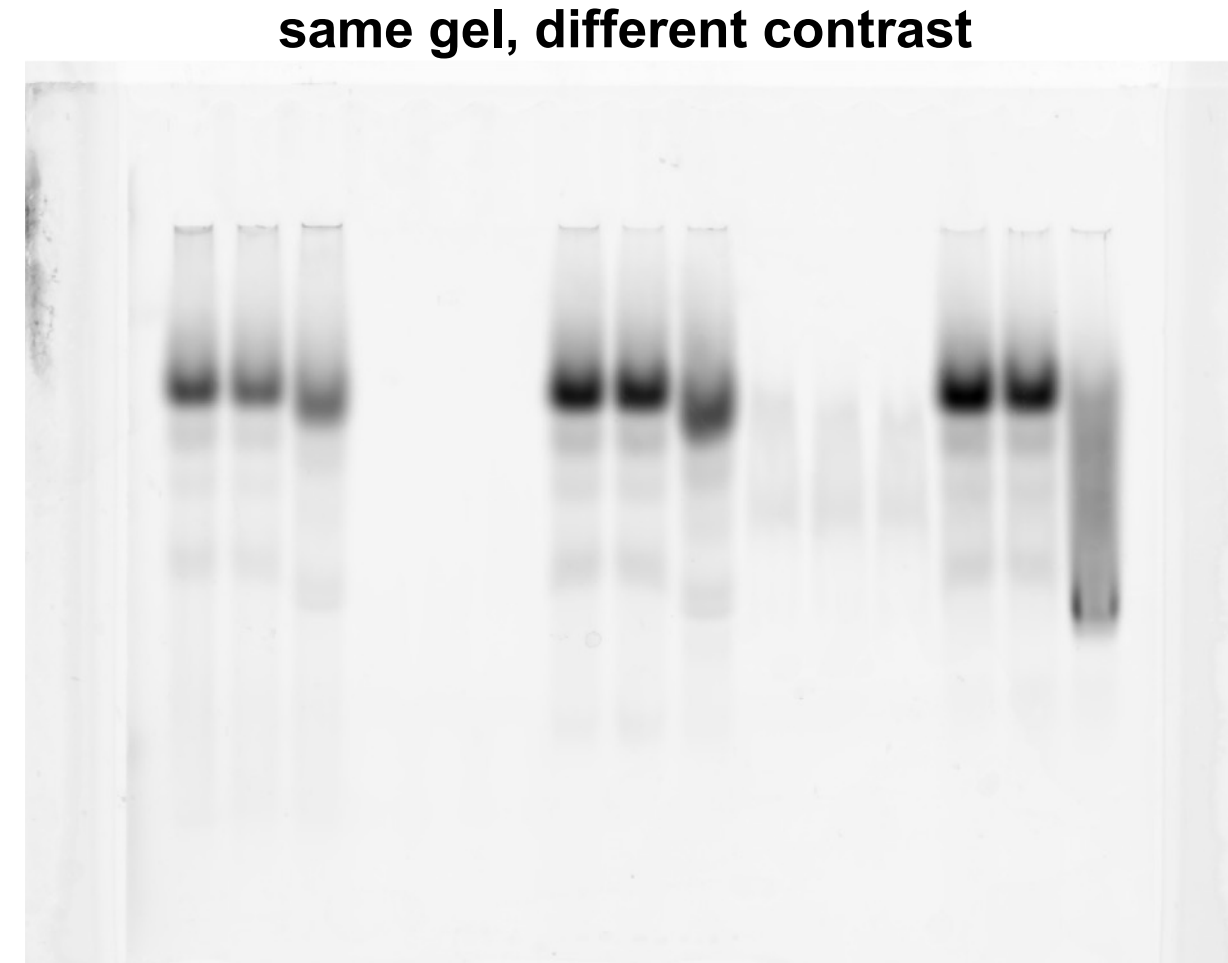

Cy2 scans (visualize FAM-BRD4$_{BD1}$)

Uncropped fluorescent scans of SDS-PAGE gels for Figure 3c

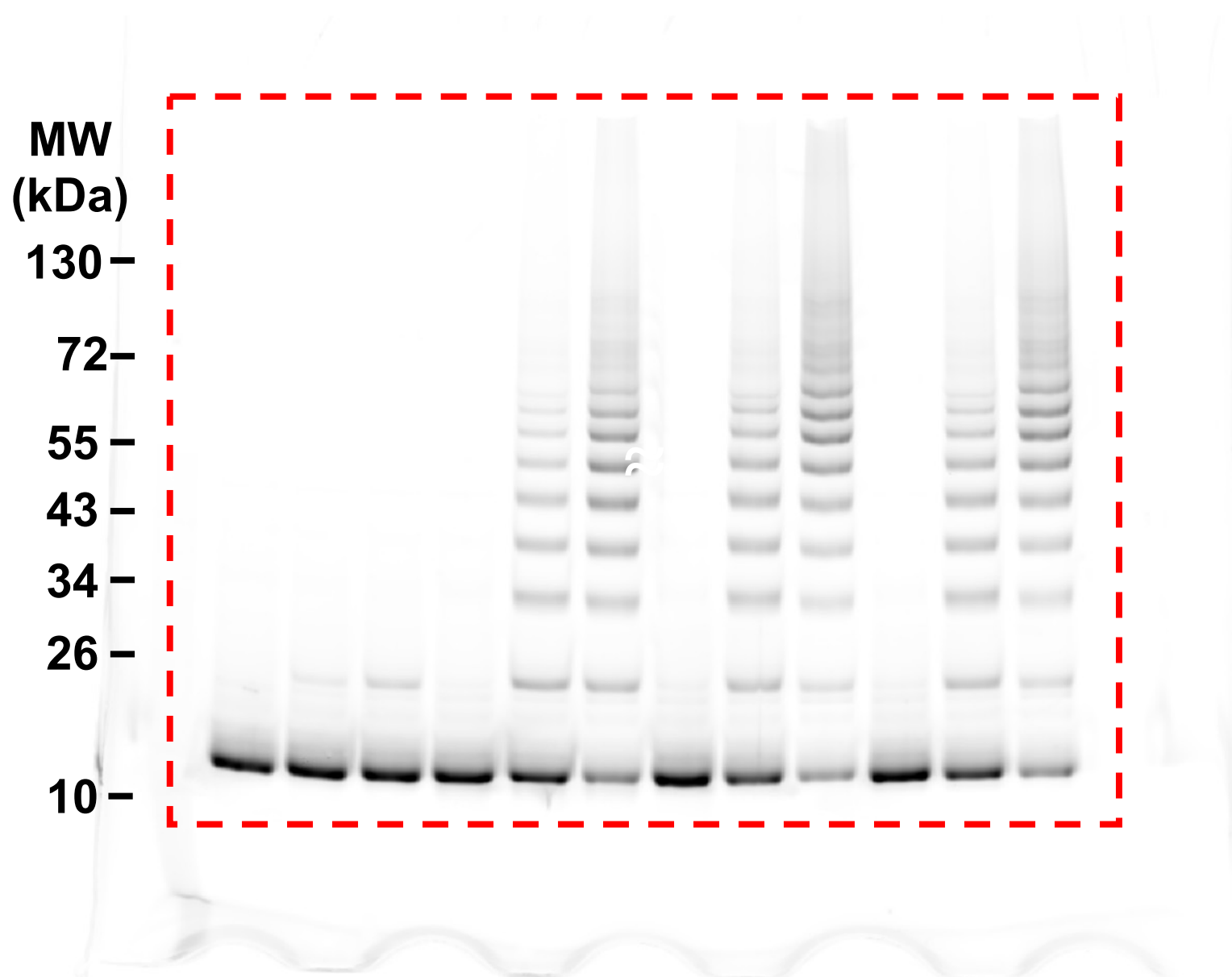

Cy2 scan (visualizes FAM-BRD4$_{BD1}$)

Uncropped fluorescent scans of SDS-PAGE gels for Figure 4c

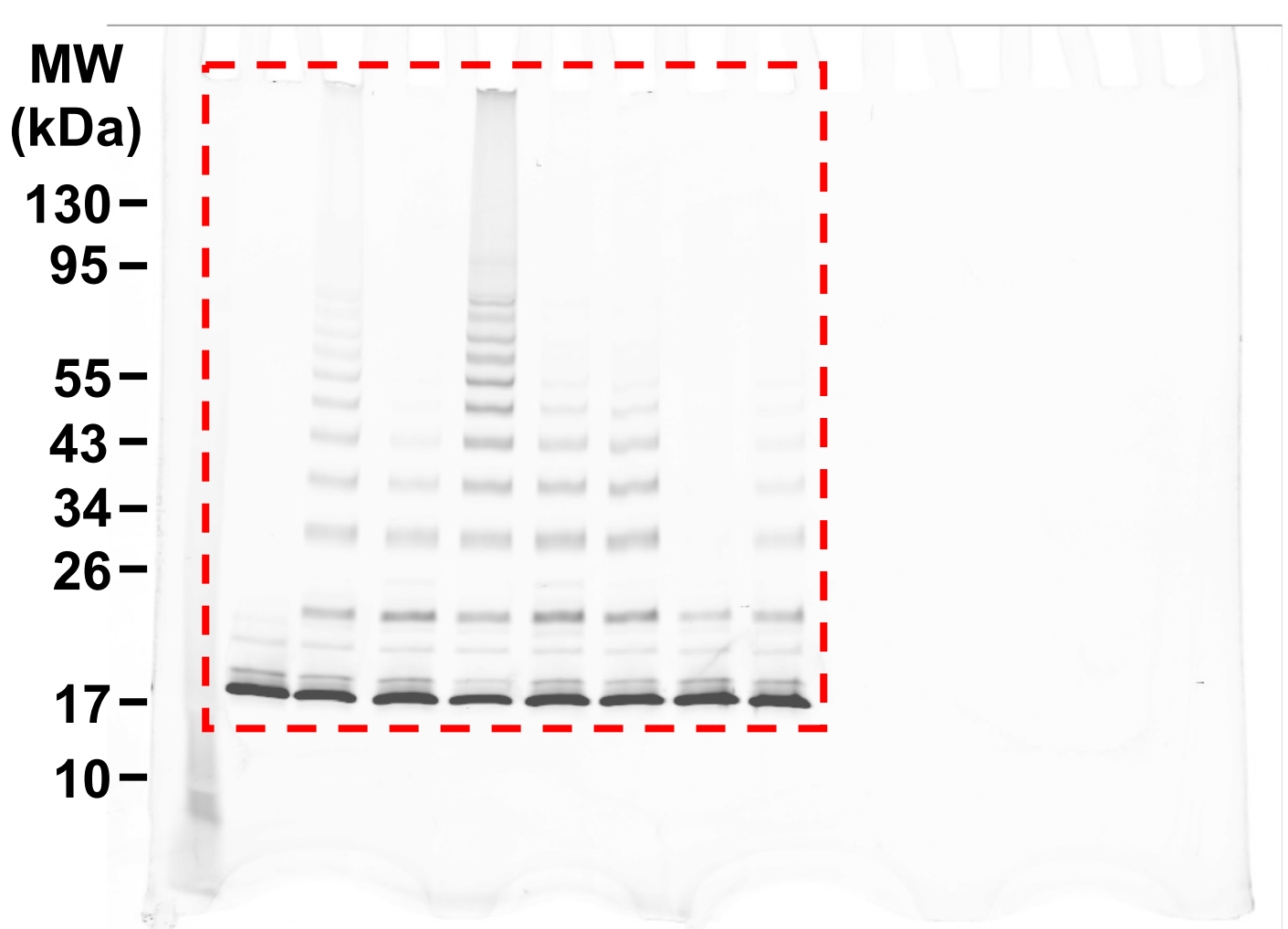

Cy2 scan (visualizes FAM-BRD4$_{BD1}$)

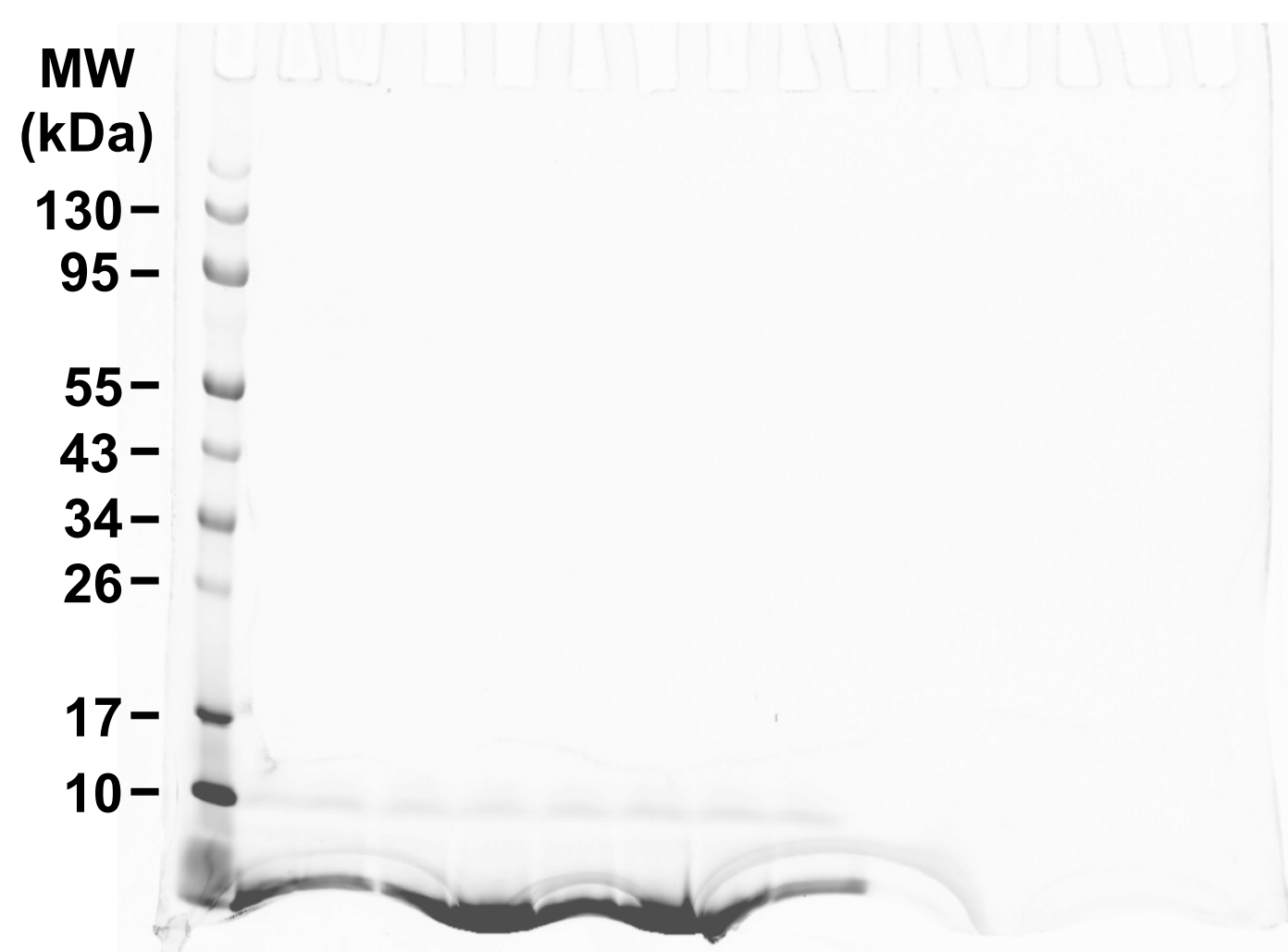

Cy5 scan (visualizes MW marker)

Uncropped fluorescent scans of SDS-PAGE gels for Figure 5d

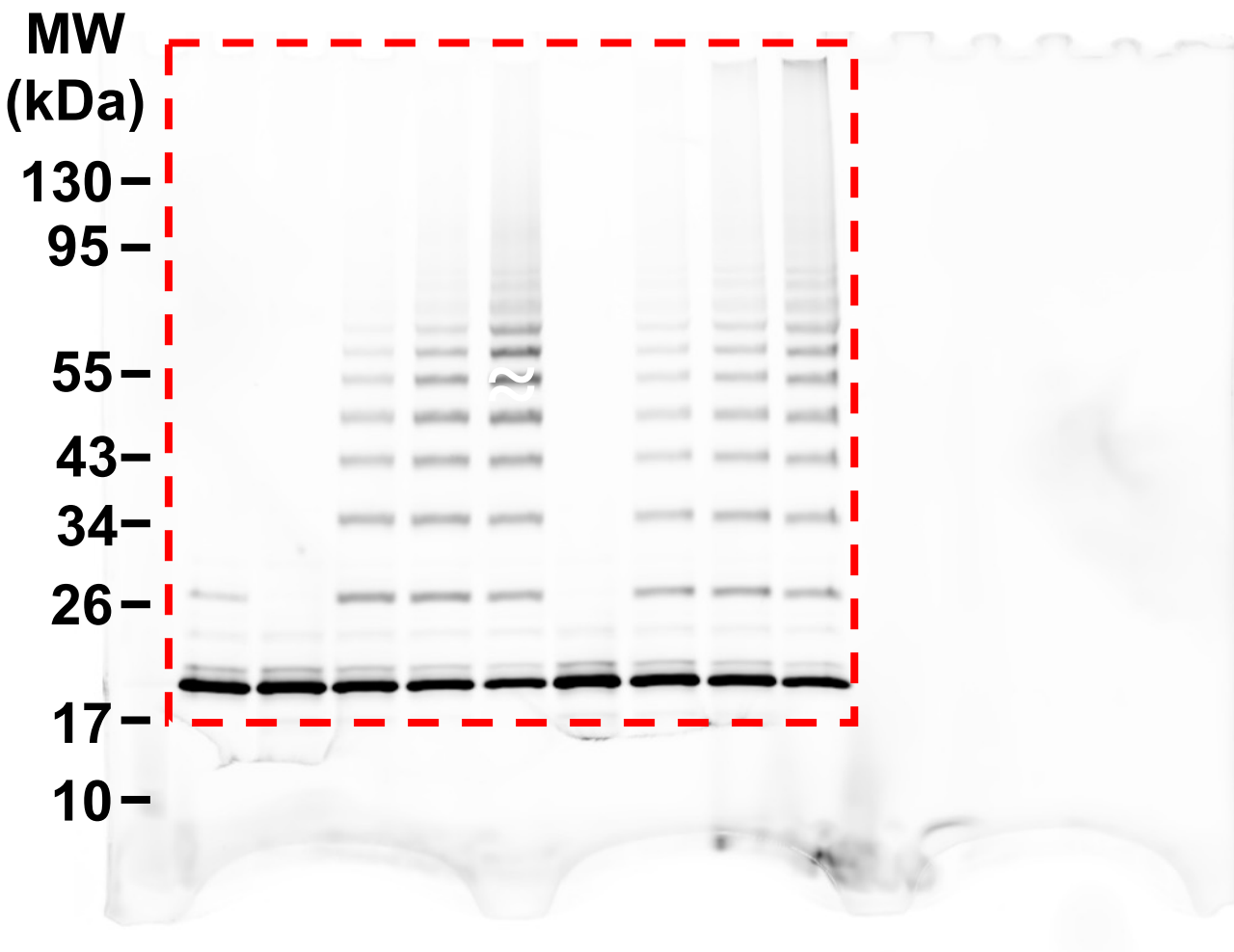

Cy2 scan (visualizes FAM-BRD4$_{BD1}$)

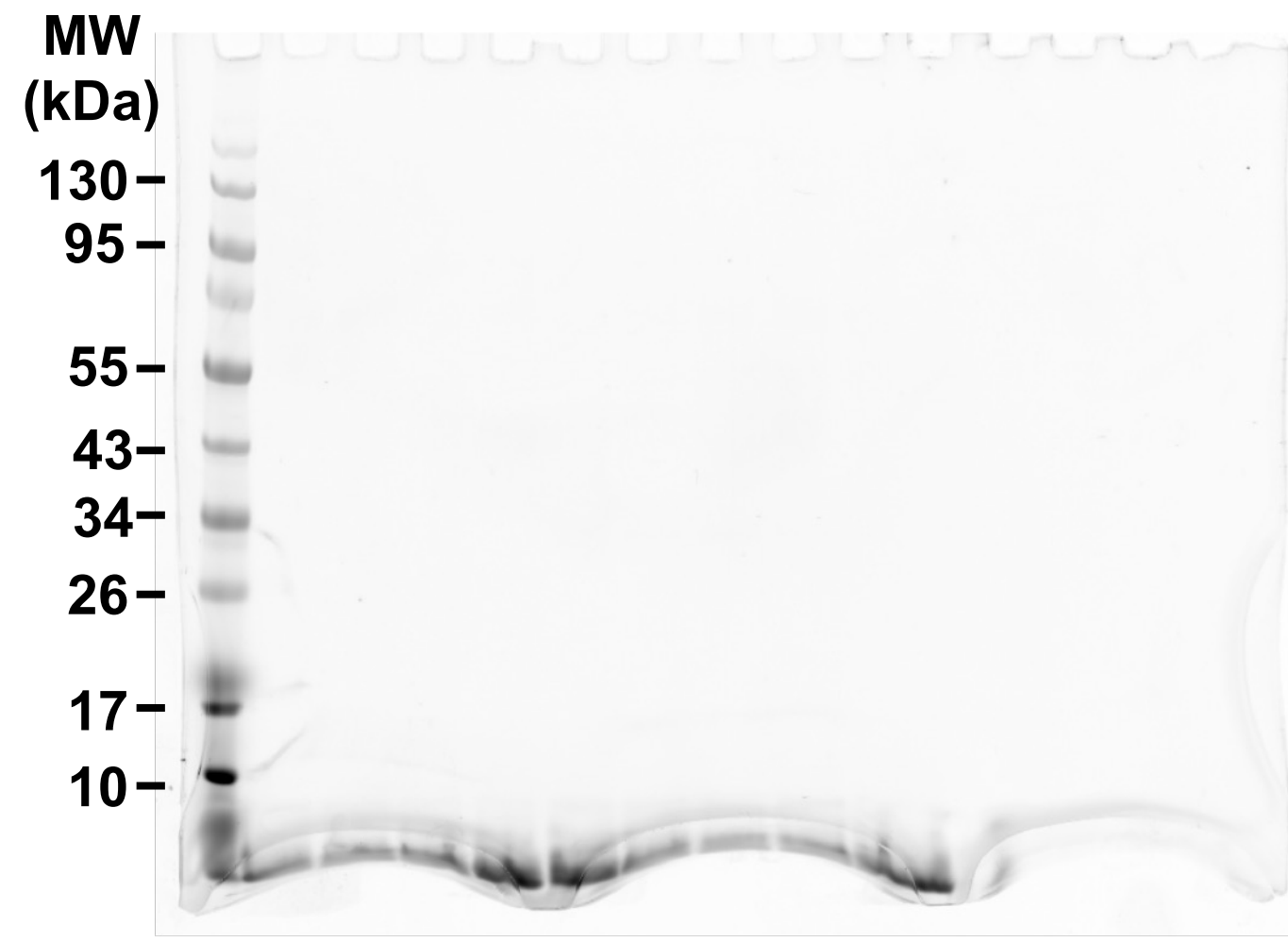

Cy5 scan (visualizes MW marker)

Uncropped Blot for Extended Data Figure 1a

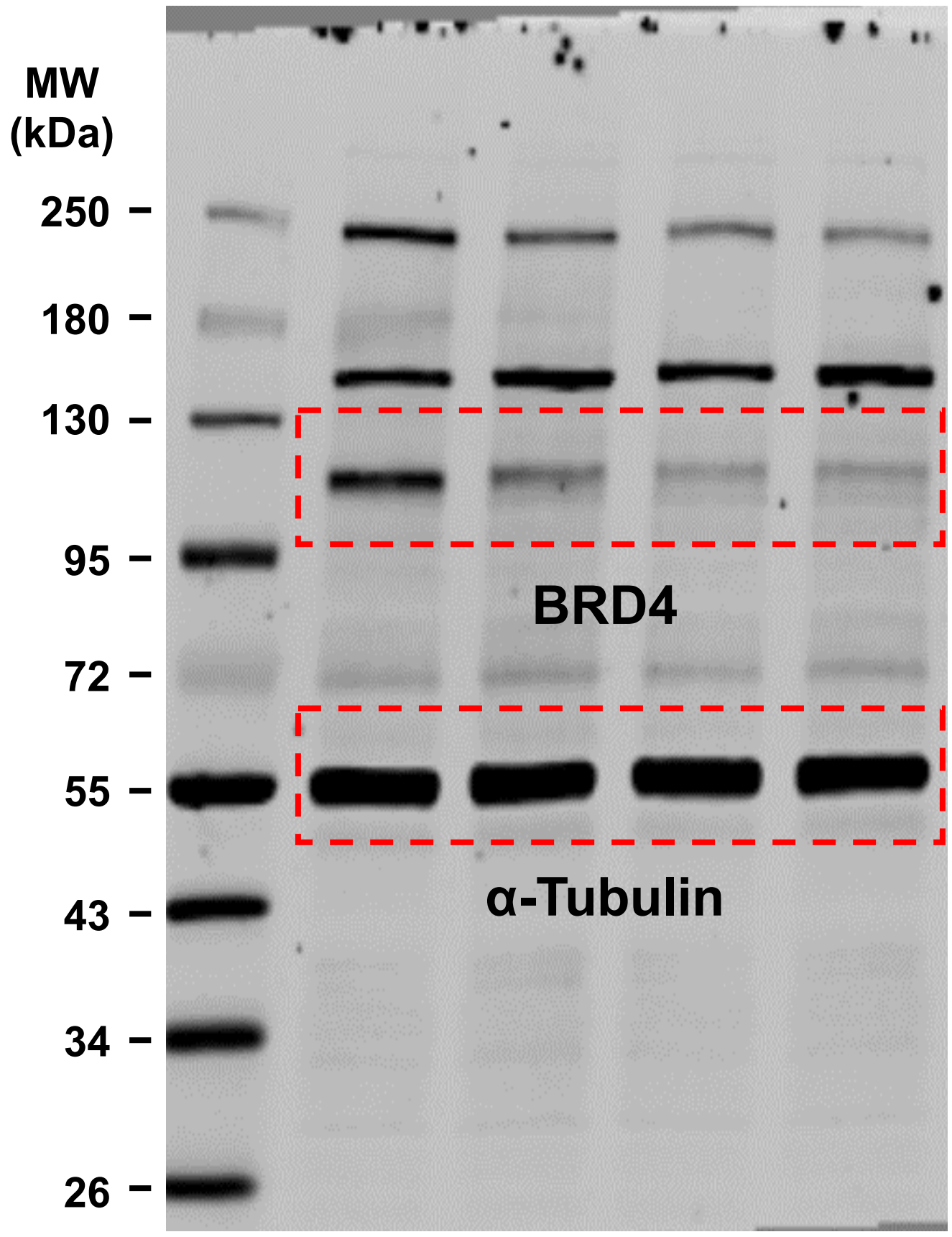

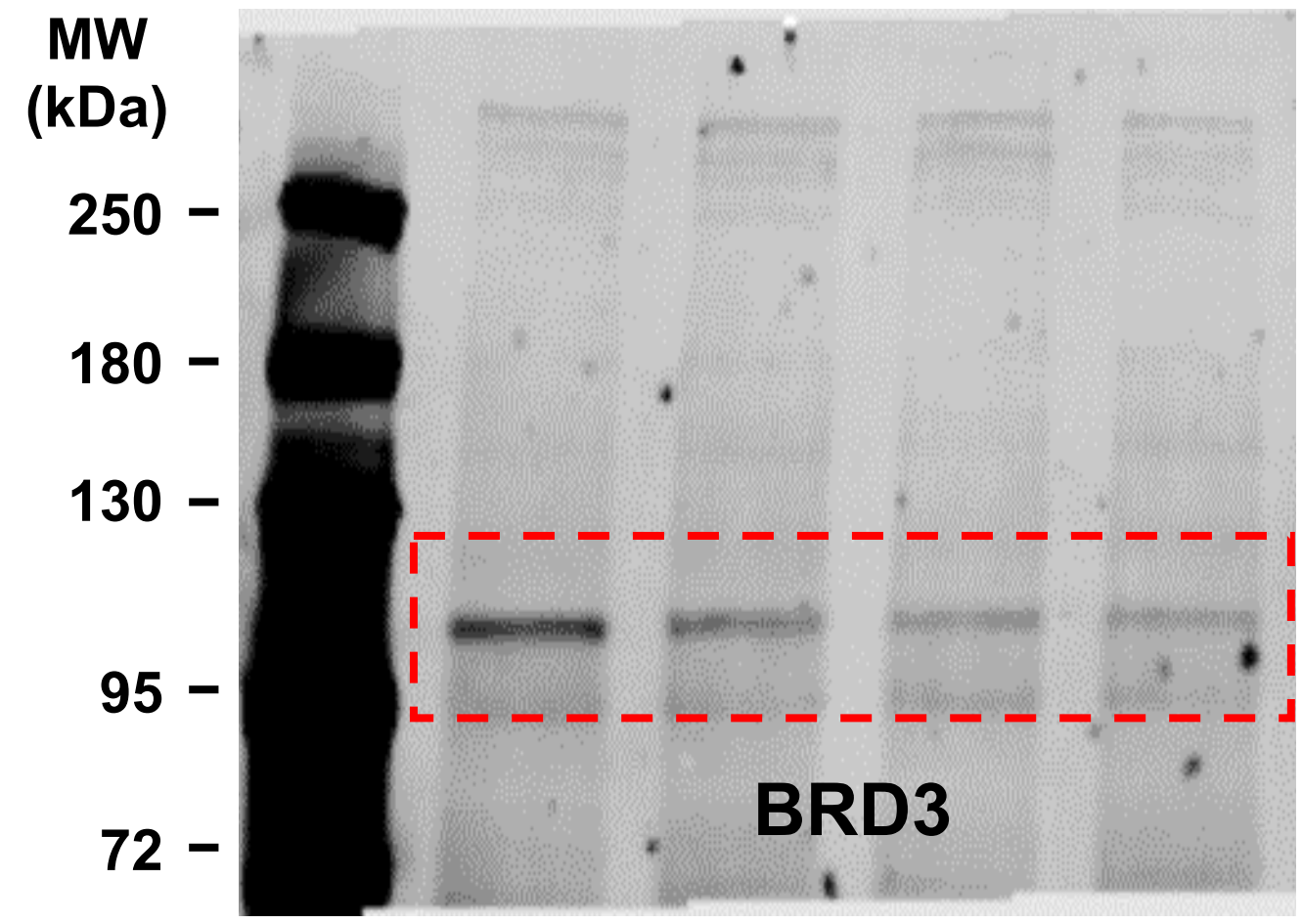

Uncropped Blot for Extended Data Figure 1b

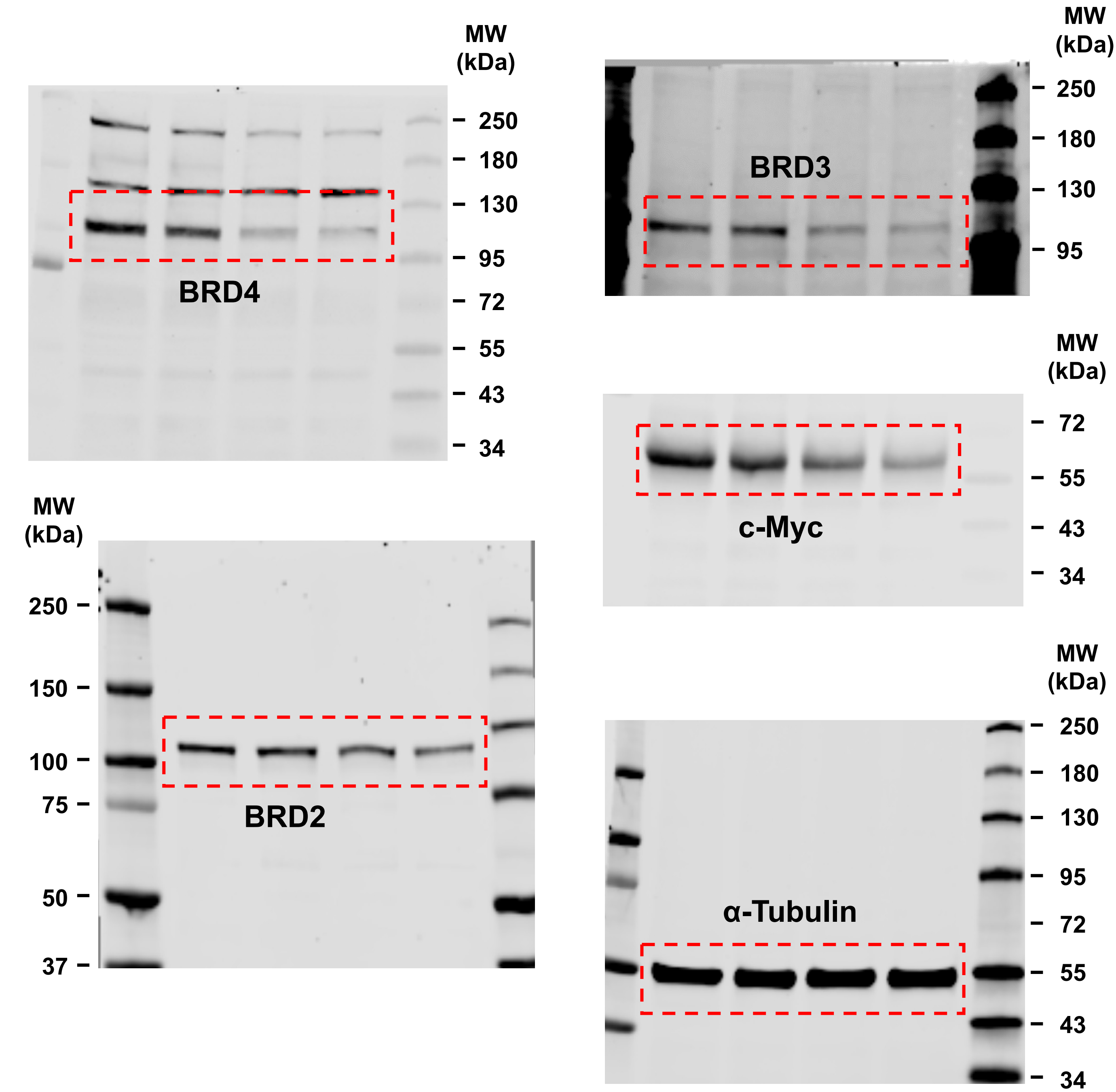

Uncropped fluorescent scans of SDS-PAGE gels for Extended Data Figure 3a

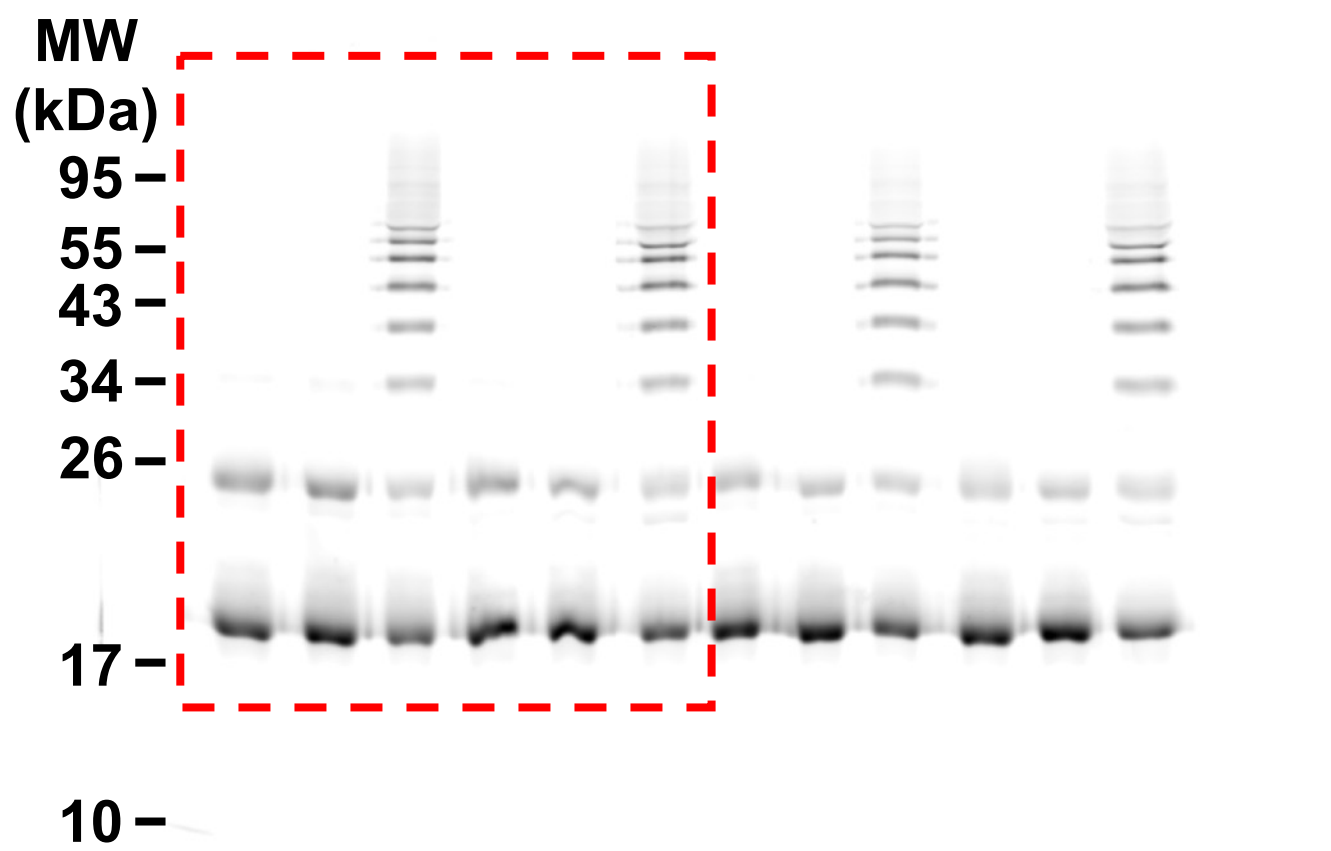

Cy2 scan (visualizes FAM-BRD4$_{BD1}$)

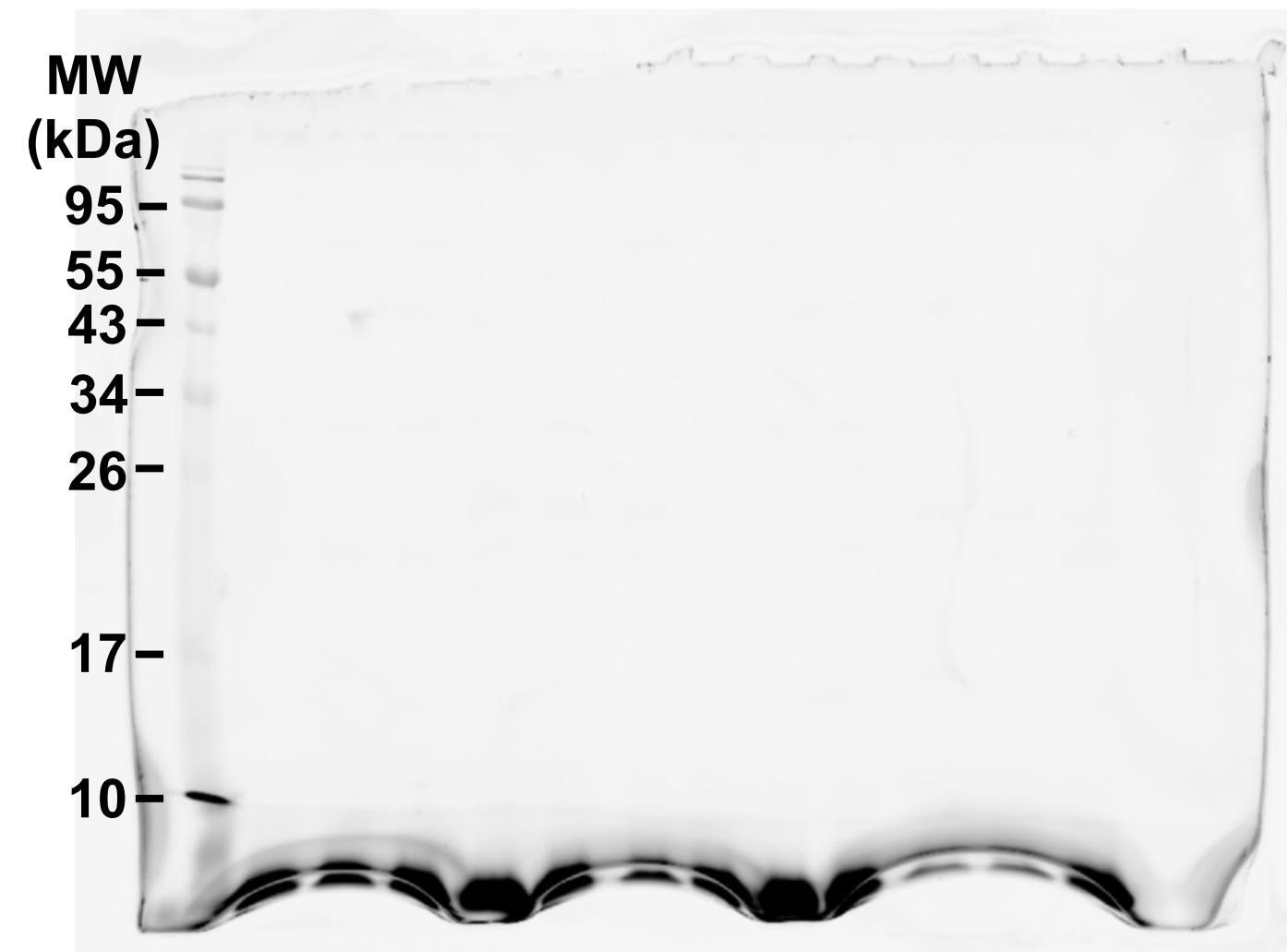

Cy5 scan (visualizes MW marker)

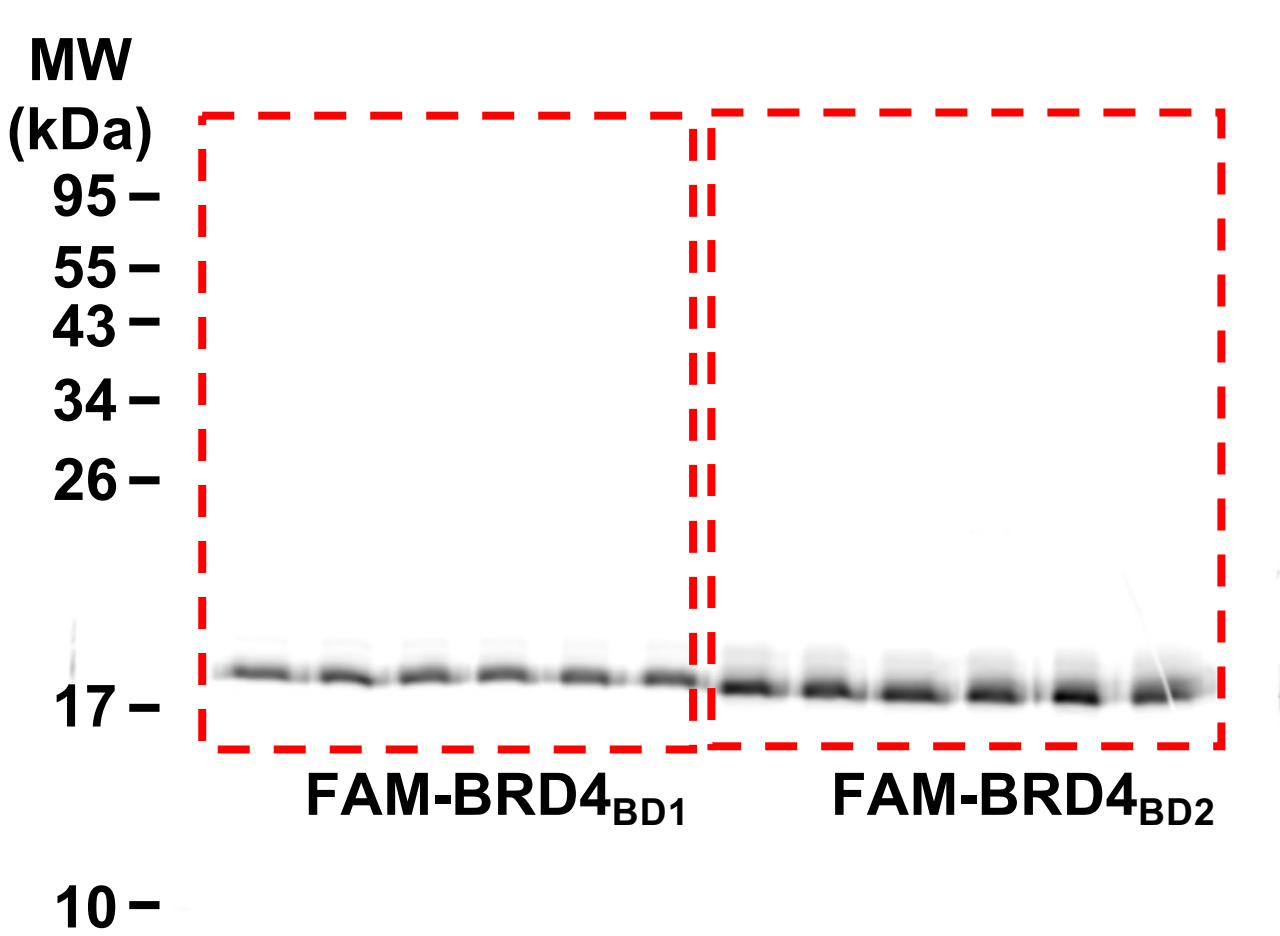

Cy2 scan (visualizes FAM-BRD4$_{BD}$)

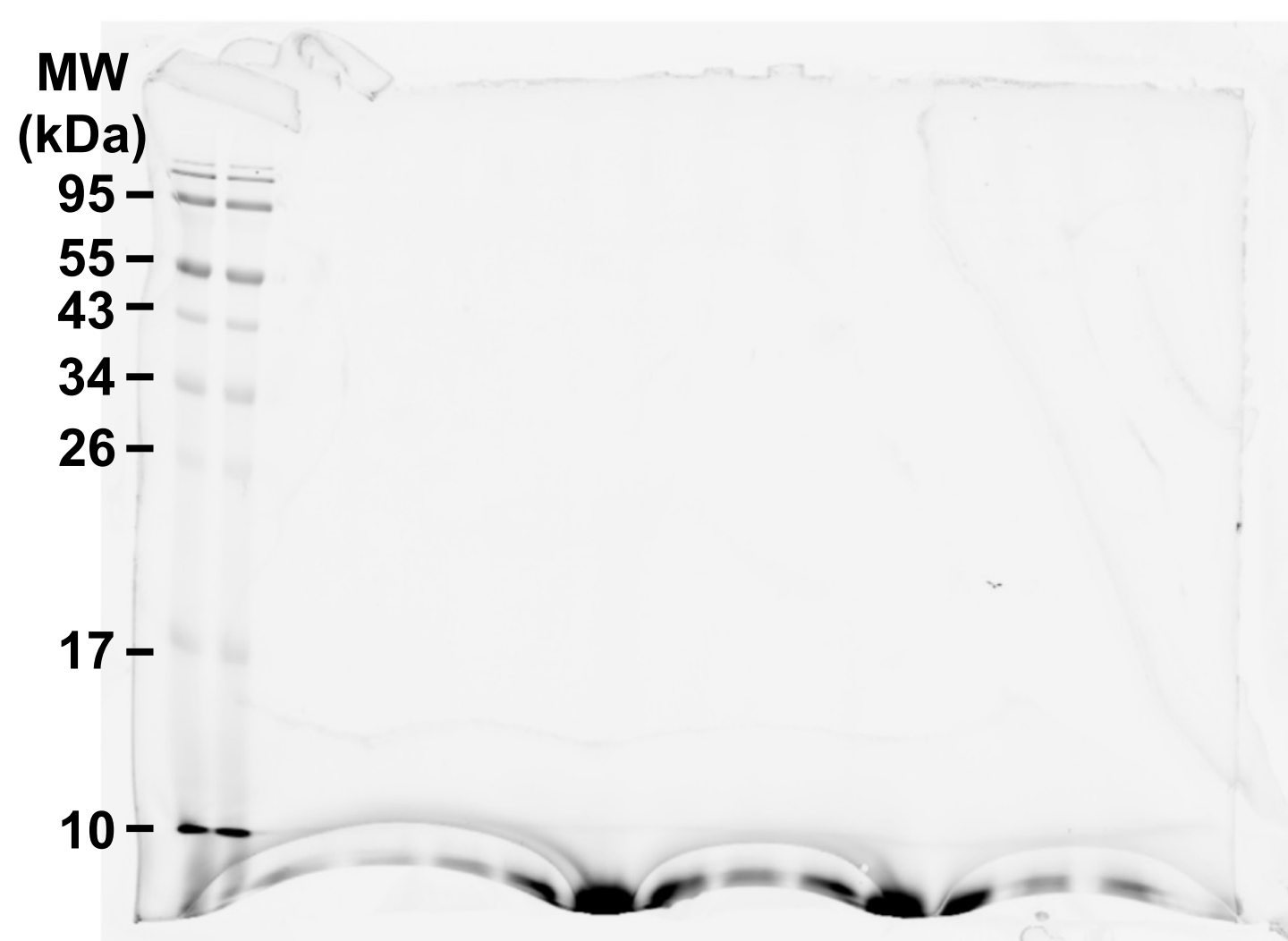

Cy5 scan (visualizes MW marker)

Uncropped fluorescent scans of SDS-PAGE gels for Extended Data Figure 3a

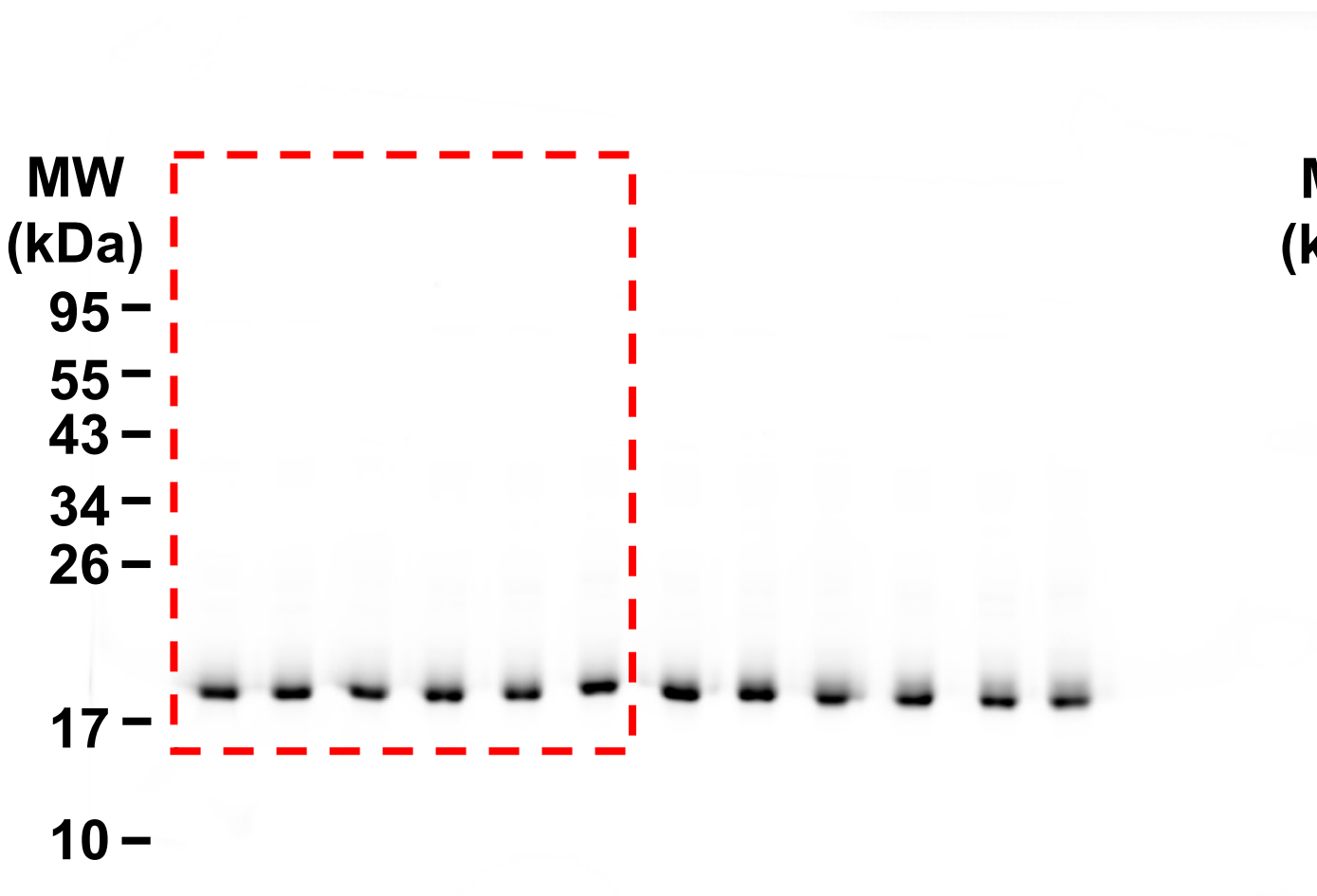

Cy2 scan (visualizes FAM-BRD4$_{BD2}$)

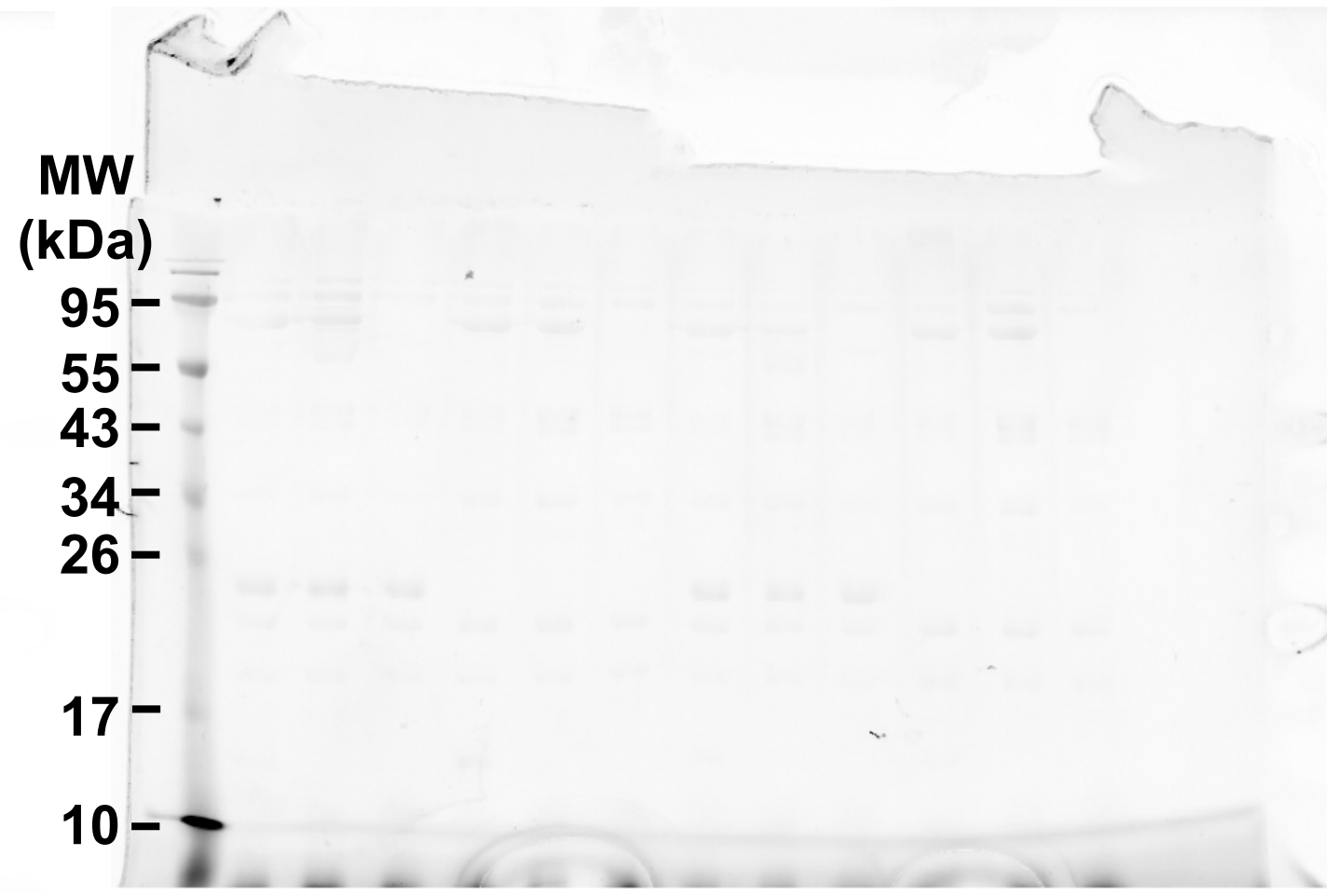

Cy5 scan (visualizes MW marker)

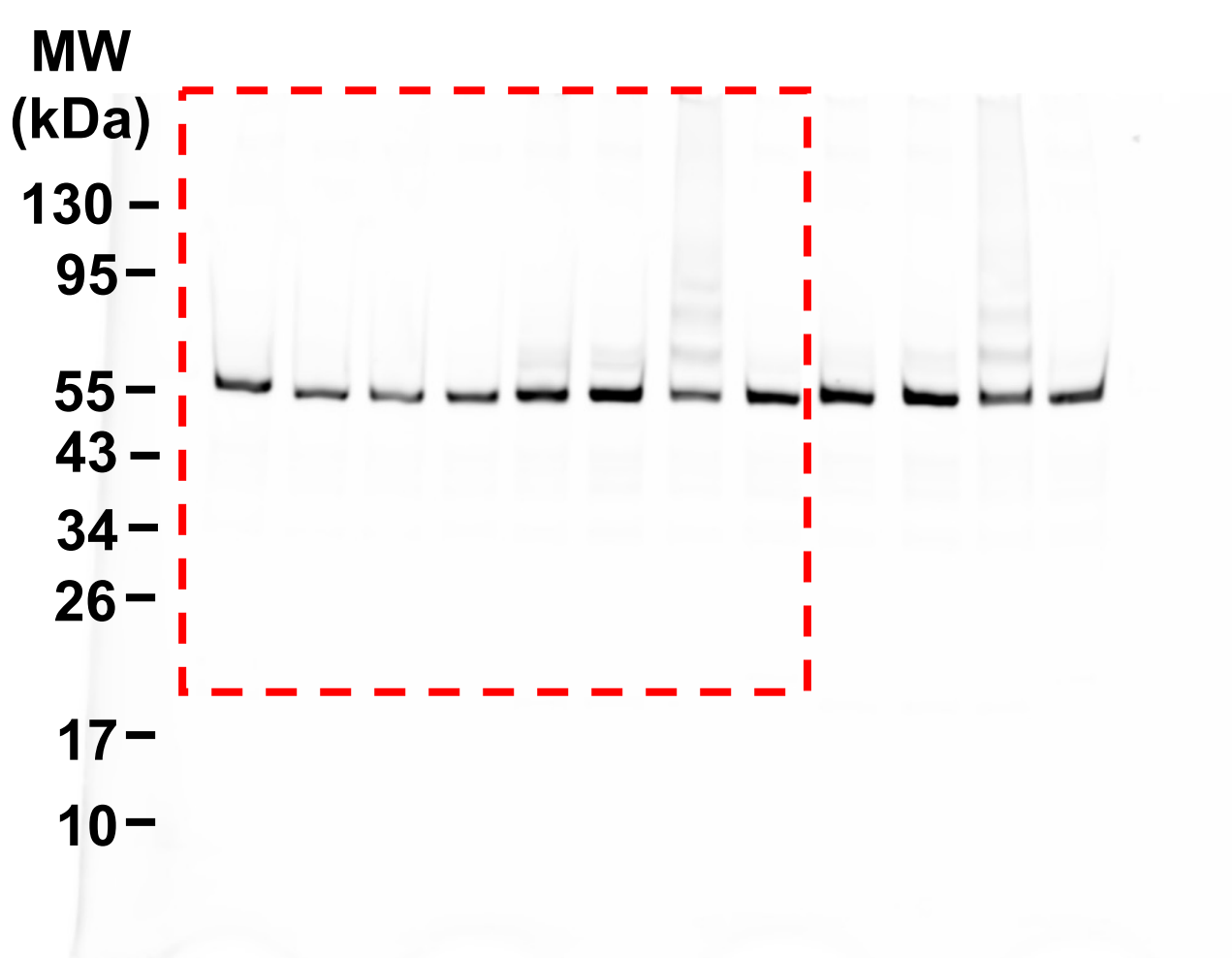

Cy2 scan (visualizes FAM-BRD4$_{BD1+BD2}$)

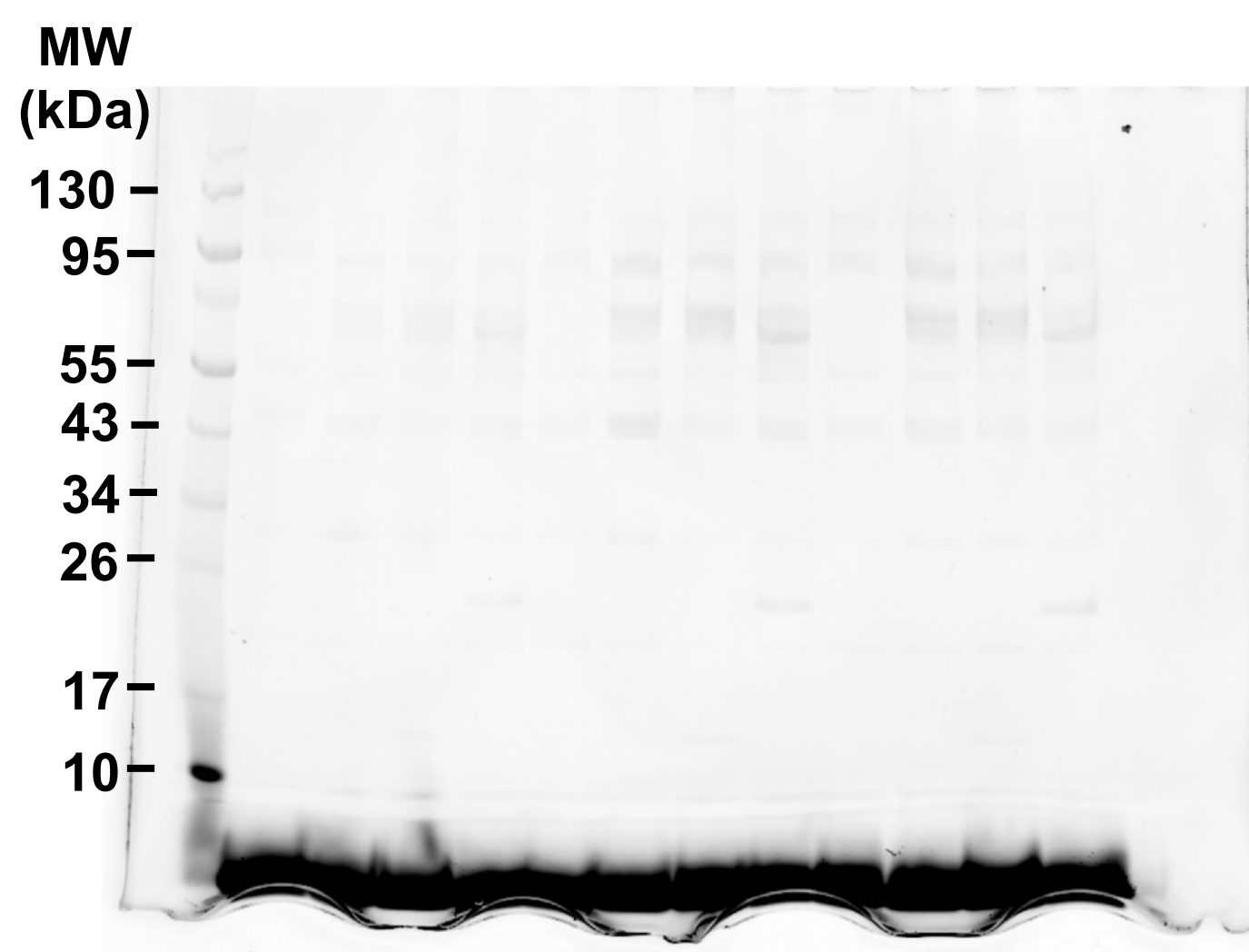

Cy5 scan (visualizes MW marker)

Uncropped Blot for Extended Data Figure 3b

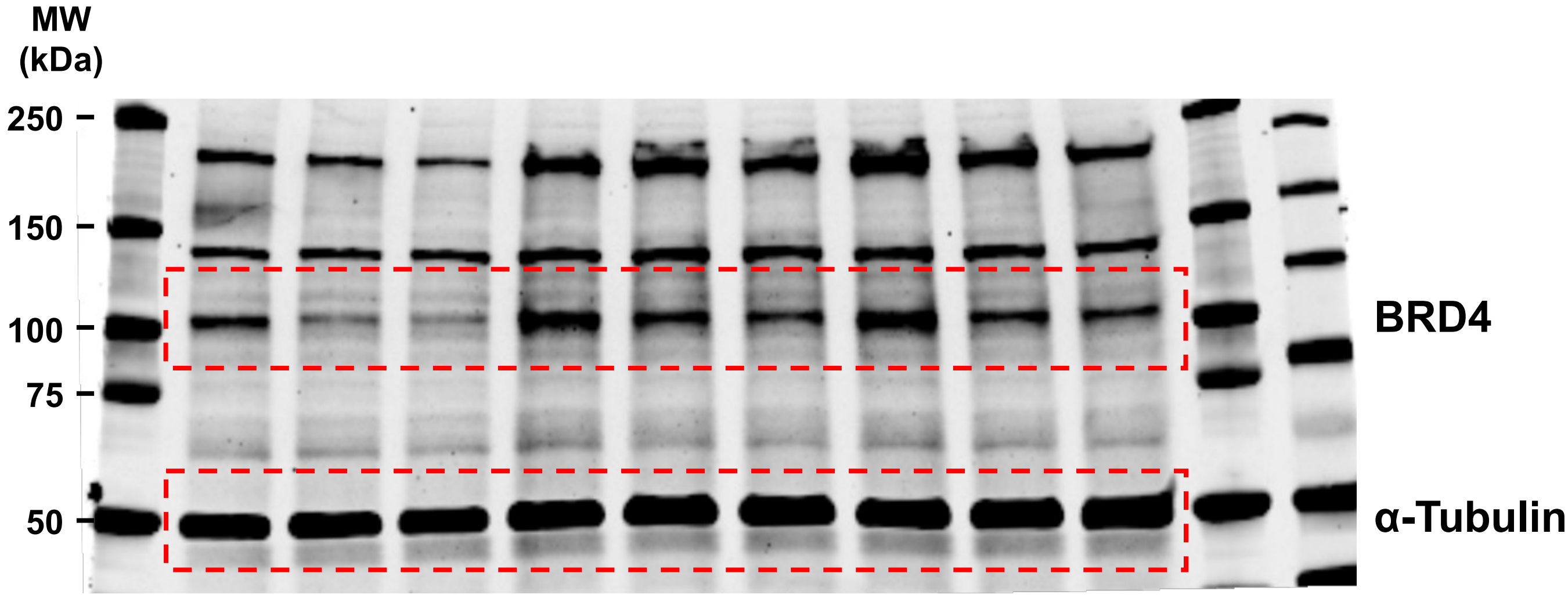

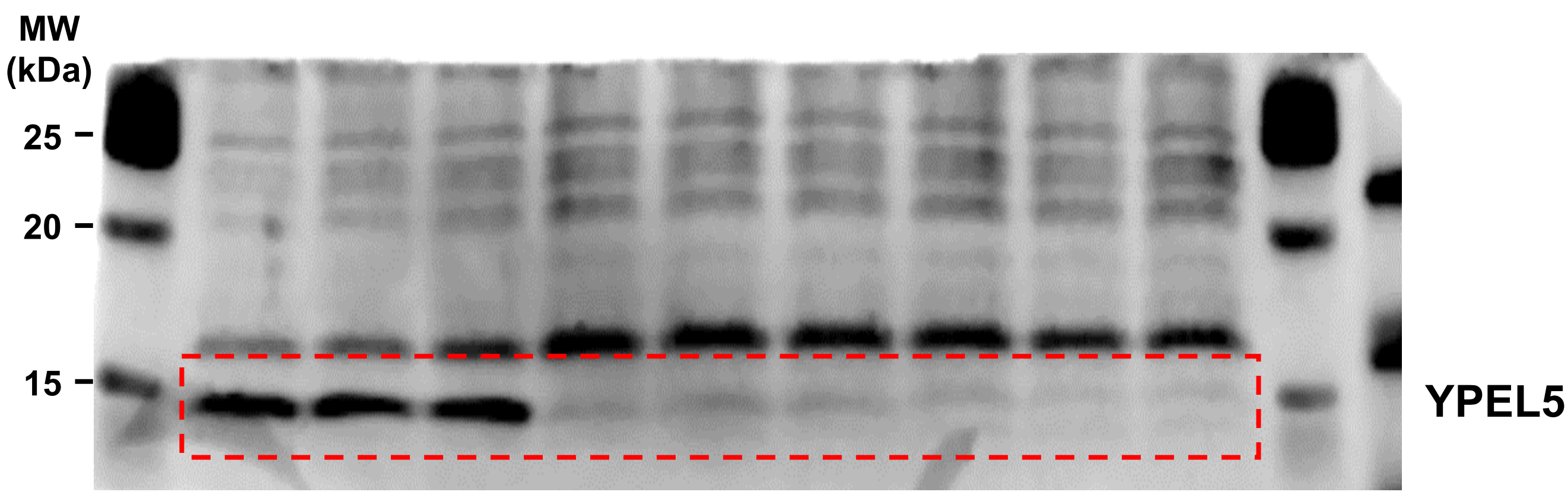

Uncropped Blot for Extended Data Figure 3c

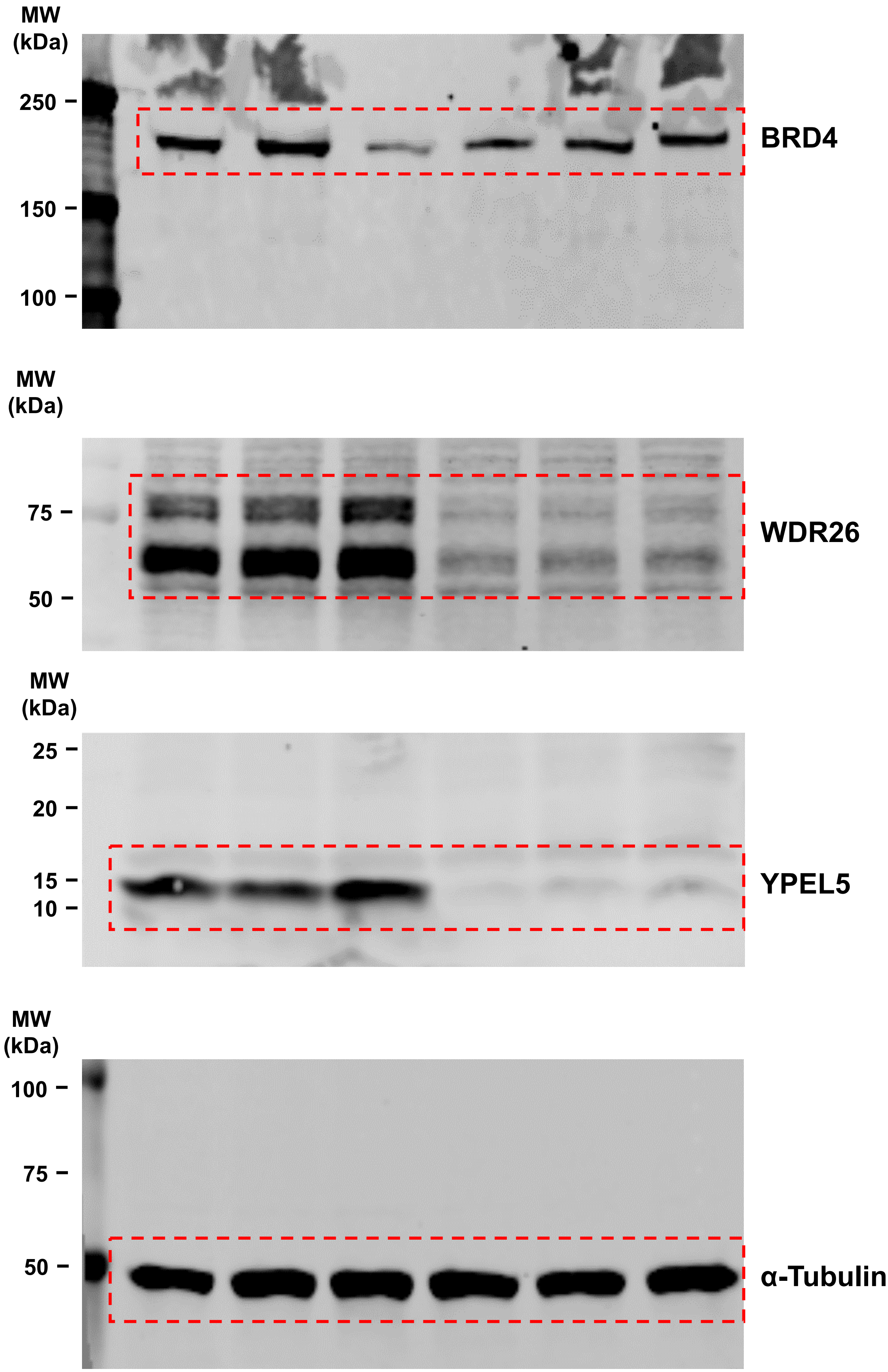

Uncropped Blot for Extended Data Figure 3d

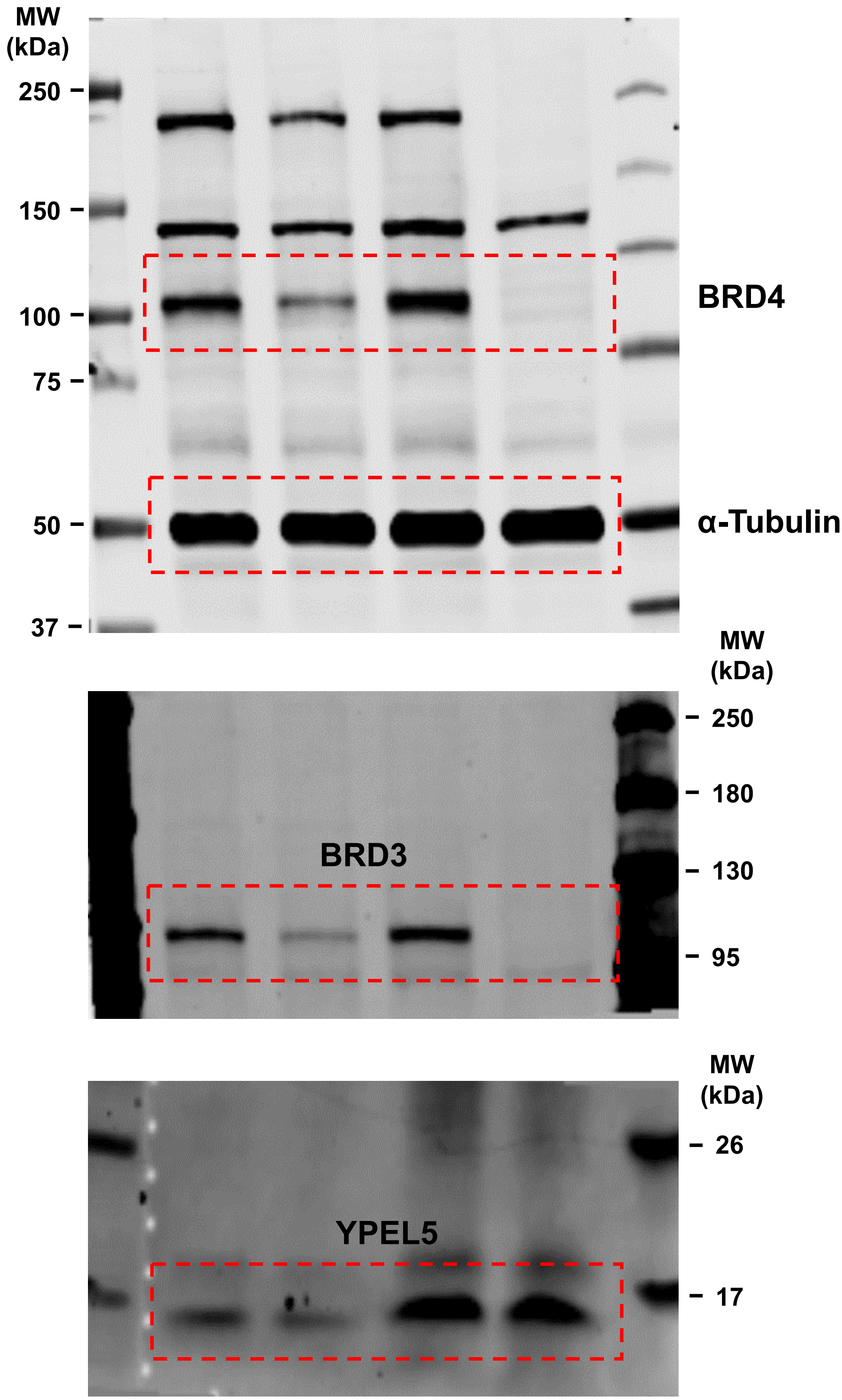

Uncropped Blot for Extended Data Figure 3e

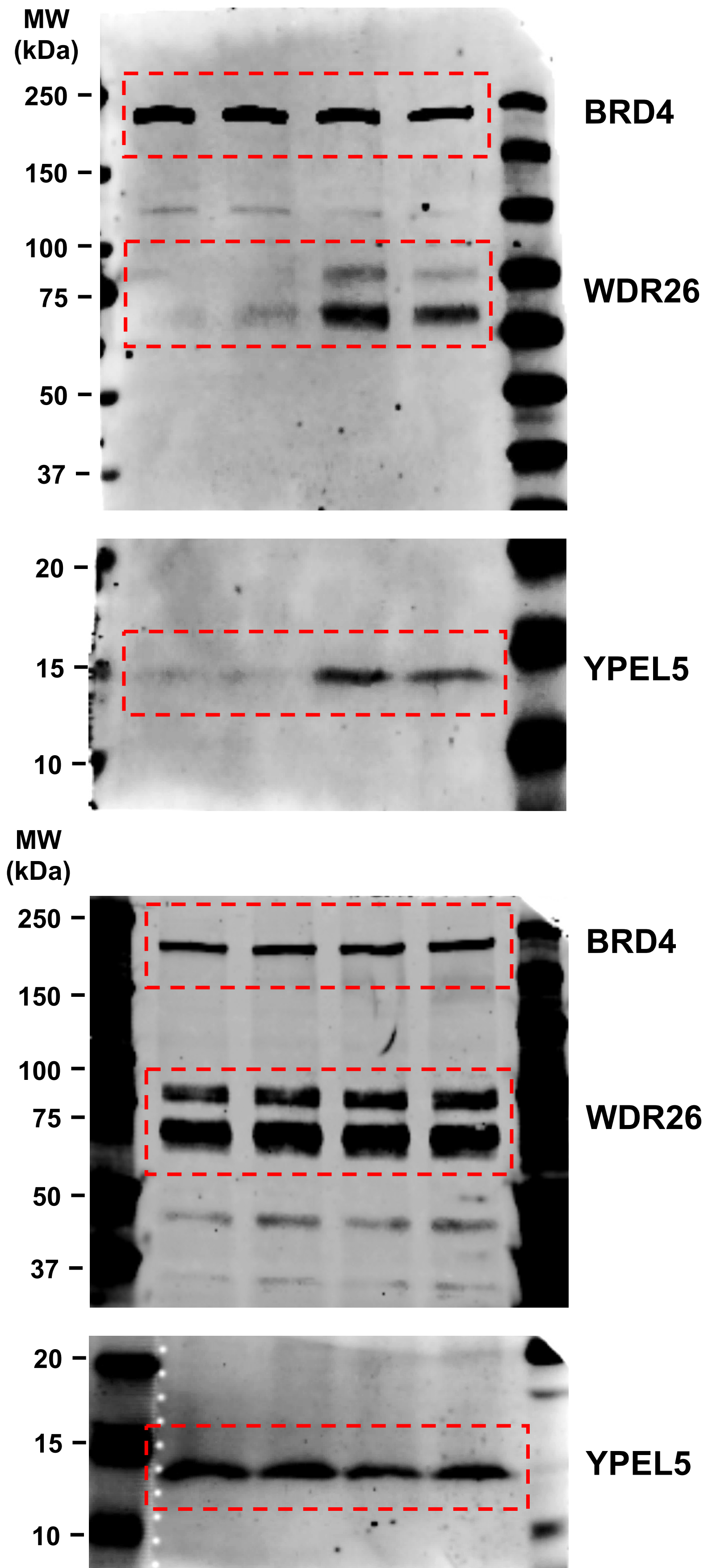

Uncropped fluorescent scans of SDS-PAGE gels for Extended Data Figure 3g

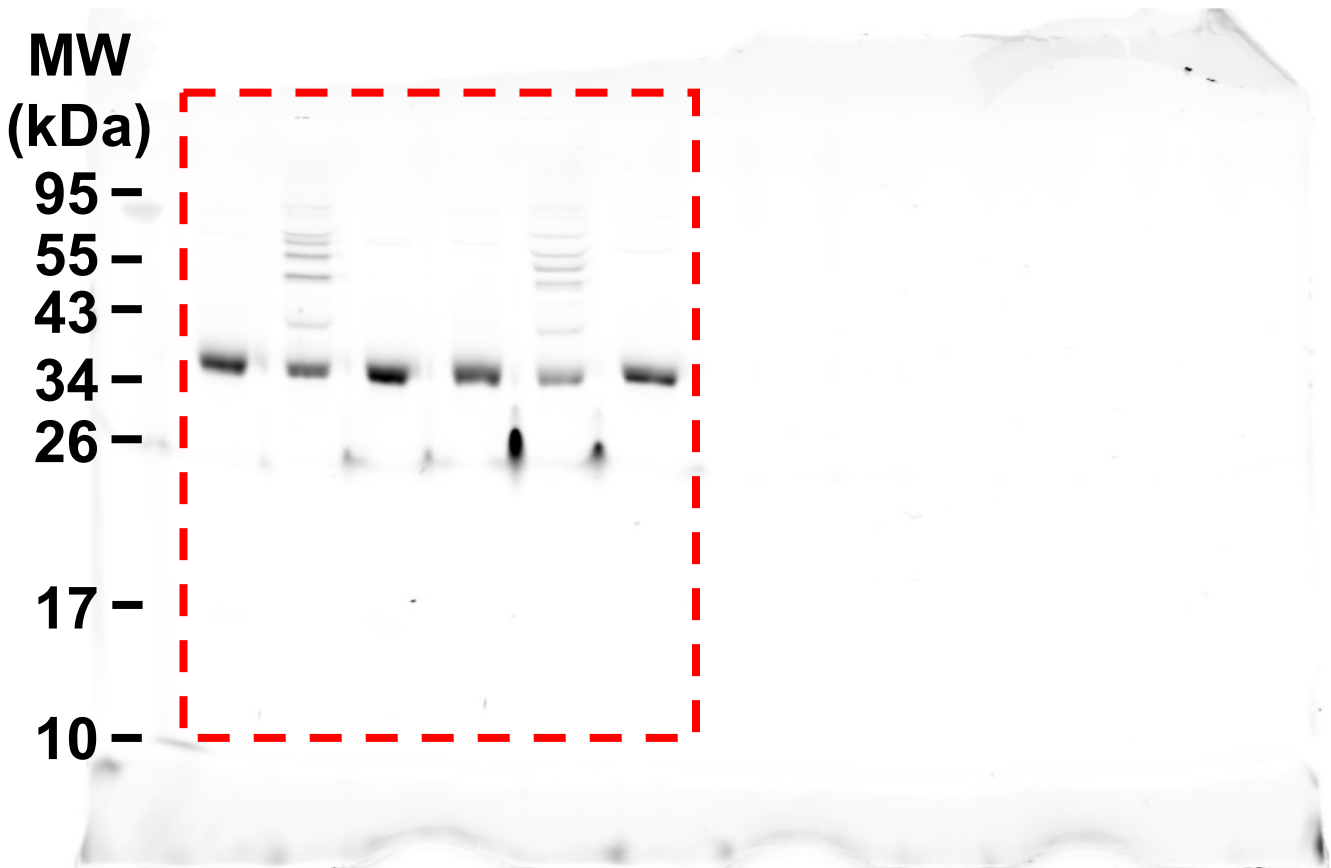

Cy3 scan (visualizes TAMRA-NMNAT1)

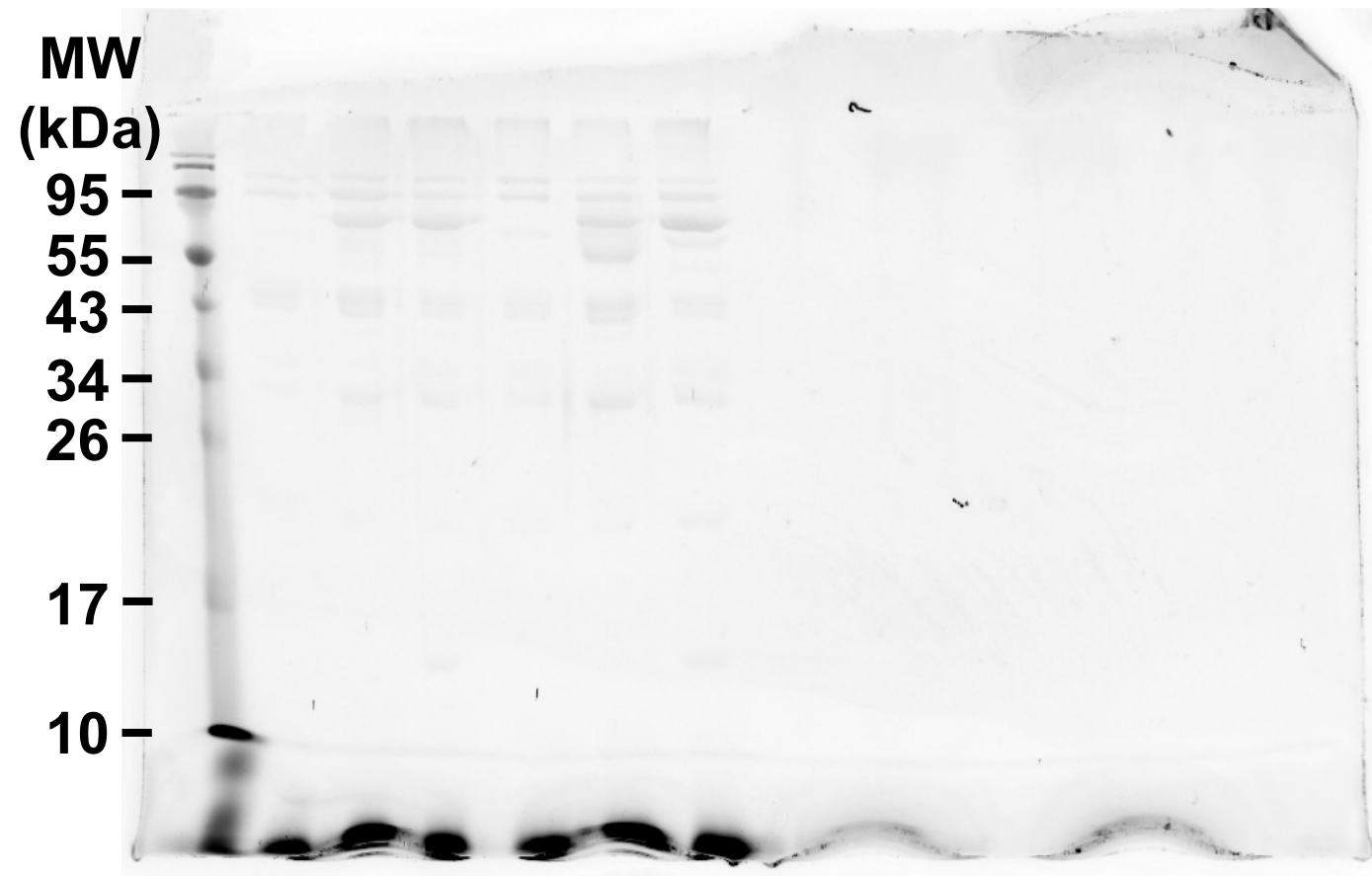

Cy5 scan (visualizes MW marker)

Uncropped fluorescent scans of SDS-PAGE gels for Extended Data Figure 4b

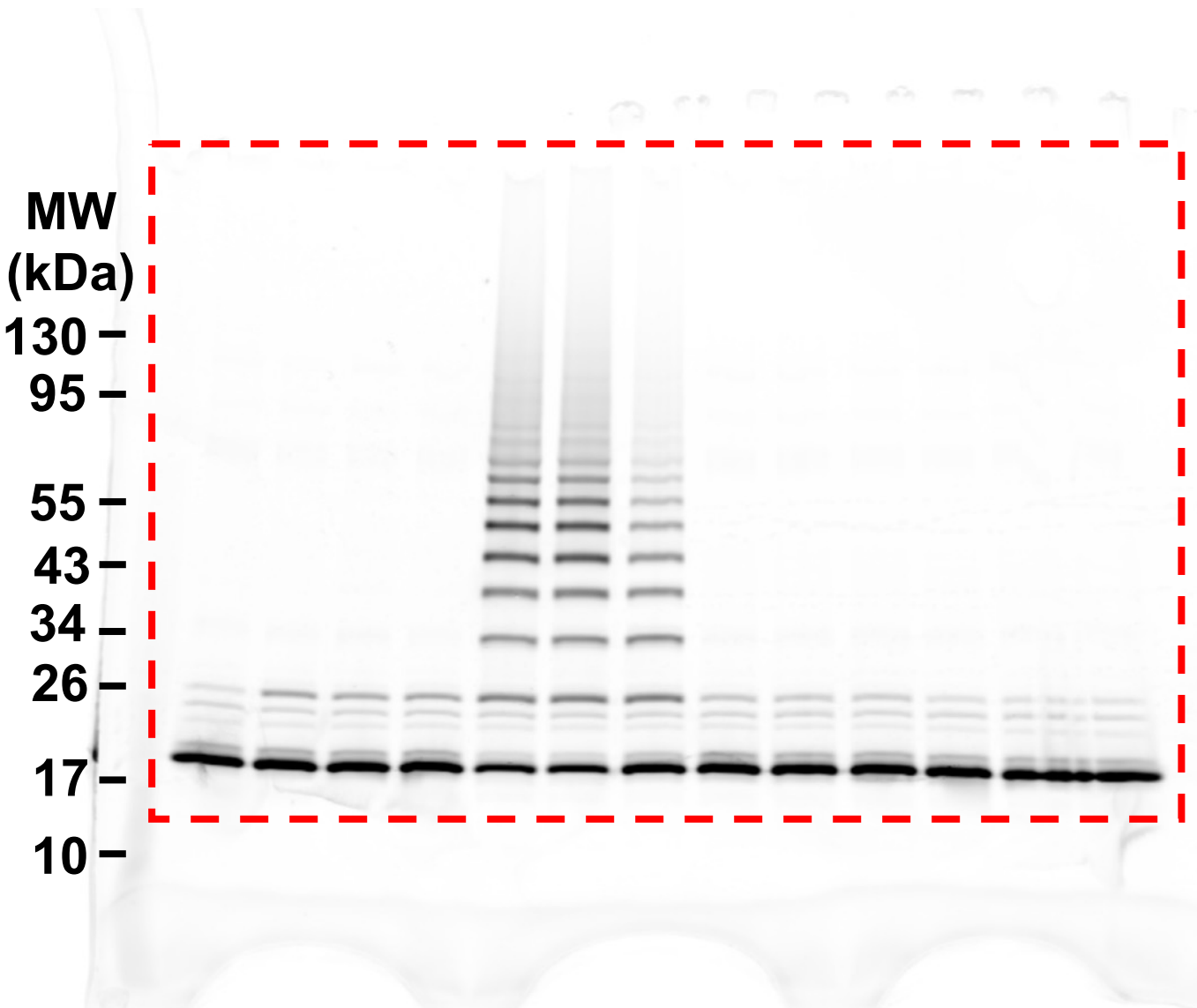

Cy2 scan (visualizes FAM-BRD4$_{BD1}$)

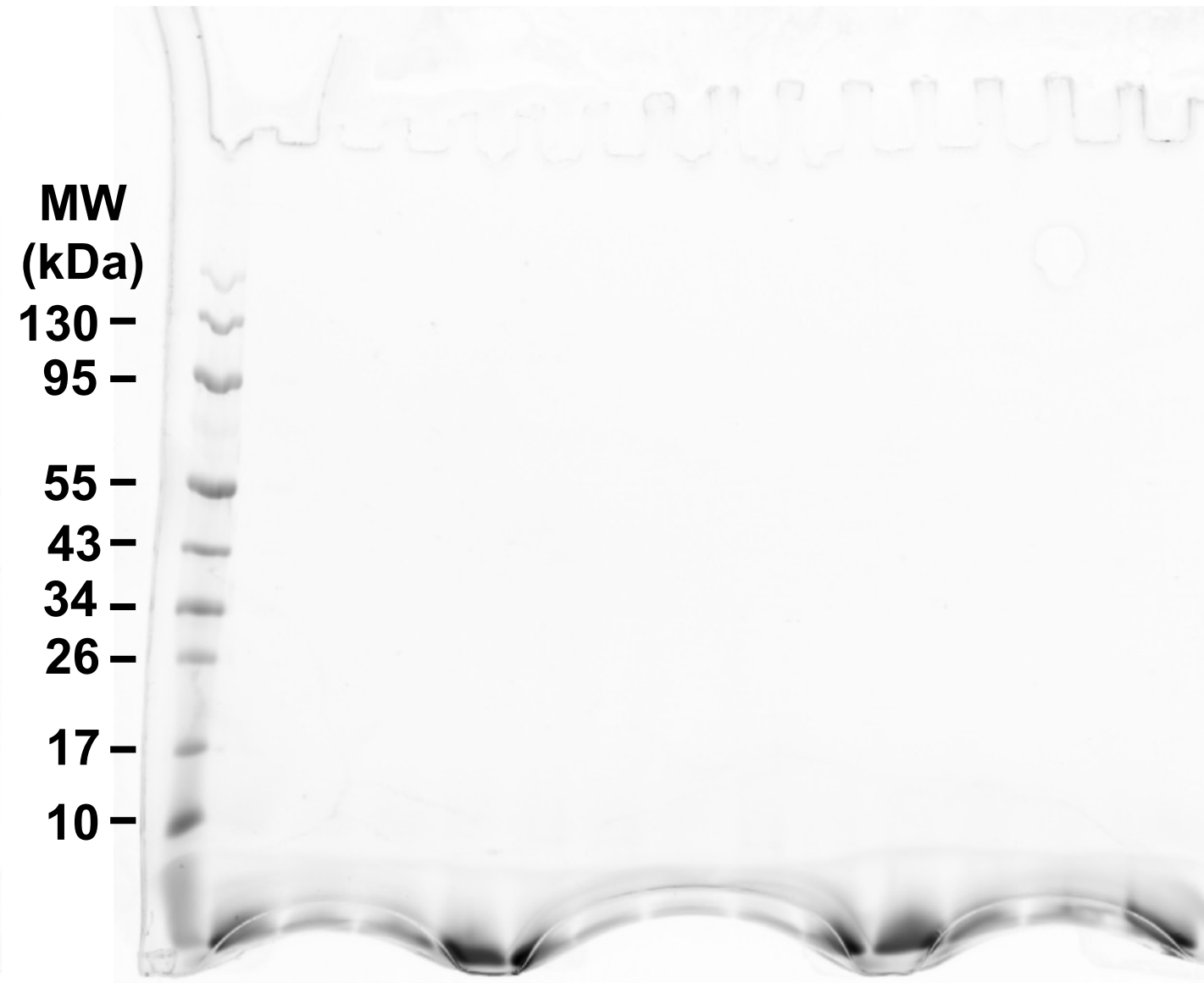

Cy5 scan (visualizes MW marker)

Uncropped fluorescent scans of SDS-PAGE gels for Extended Data Figure 6b

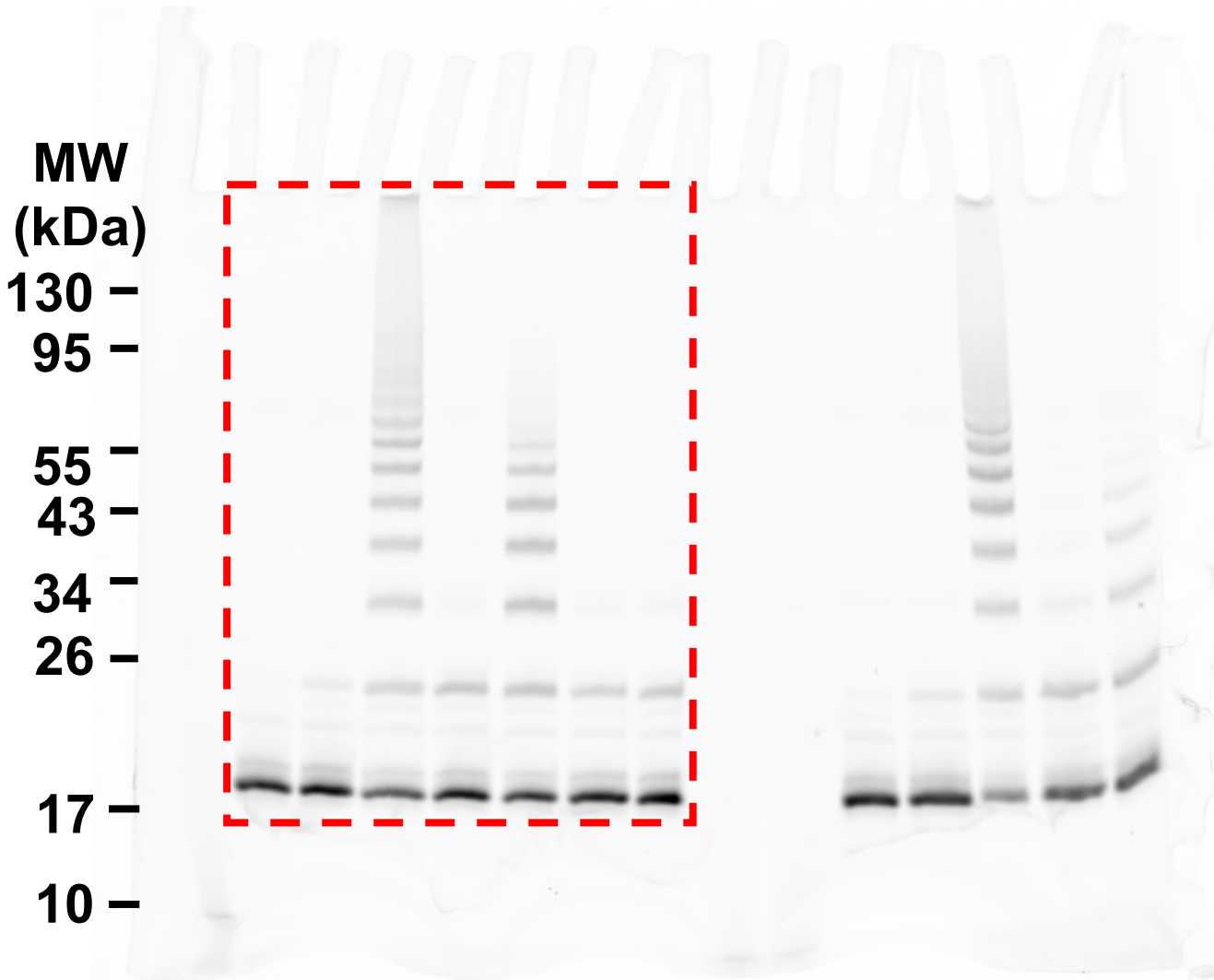

Cy2 scan (visualizes FAM-BRD4$_{BD1}$)

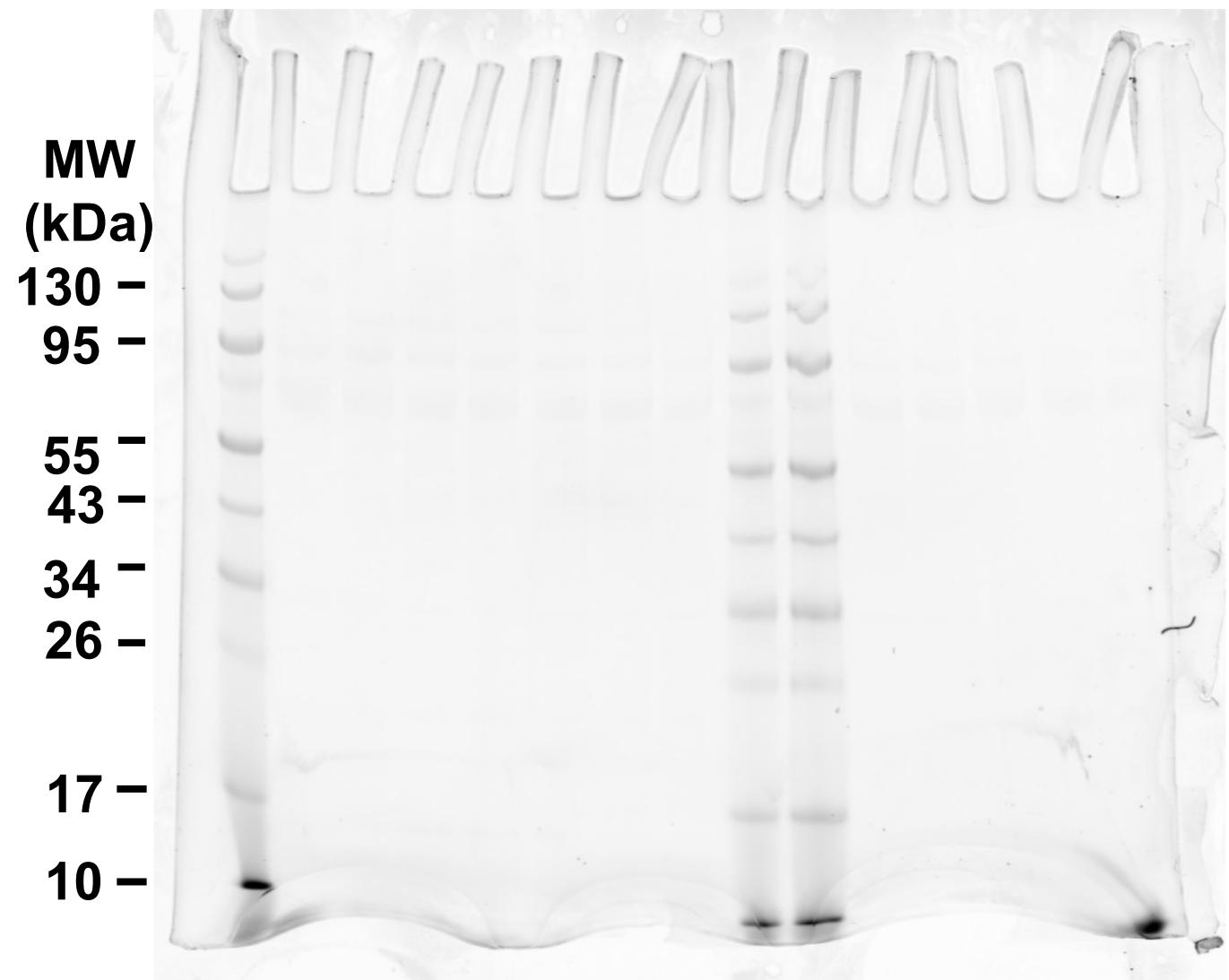

Cy5 scan (visualizes MW marker)

Uncropped fluorescent scans of SDS-PAGE gels for Extended Data Figure 7e

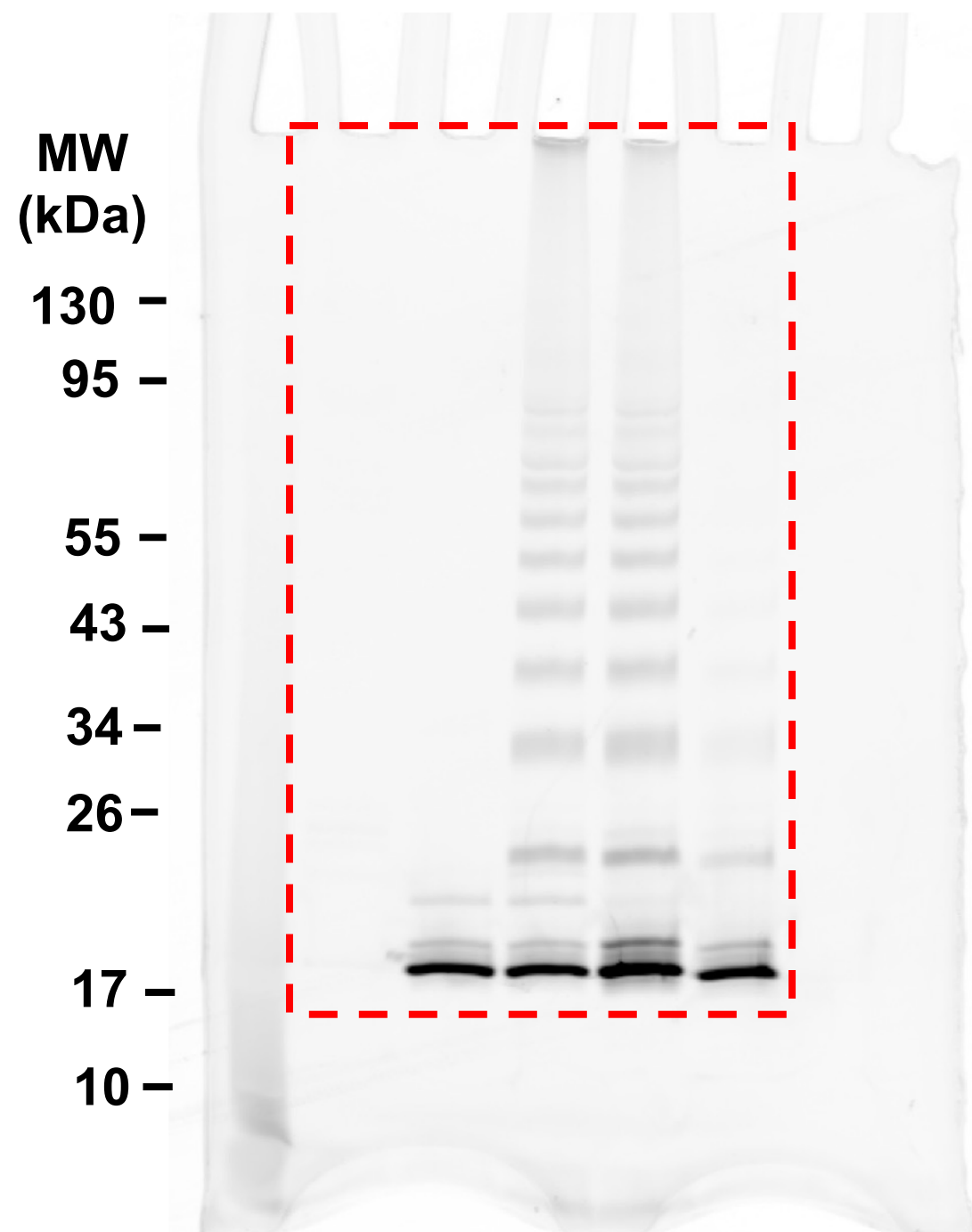

Cy2 scan (visualizes FAM-BRD4$_{BD1}$)

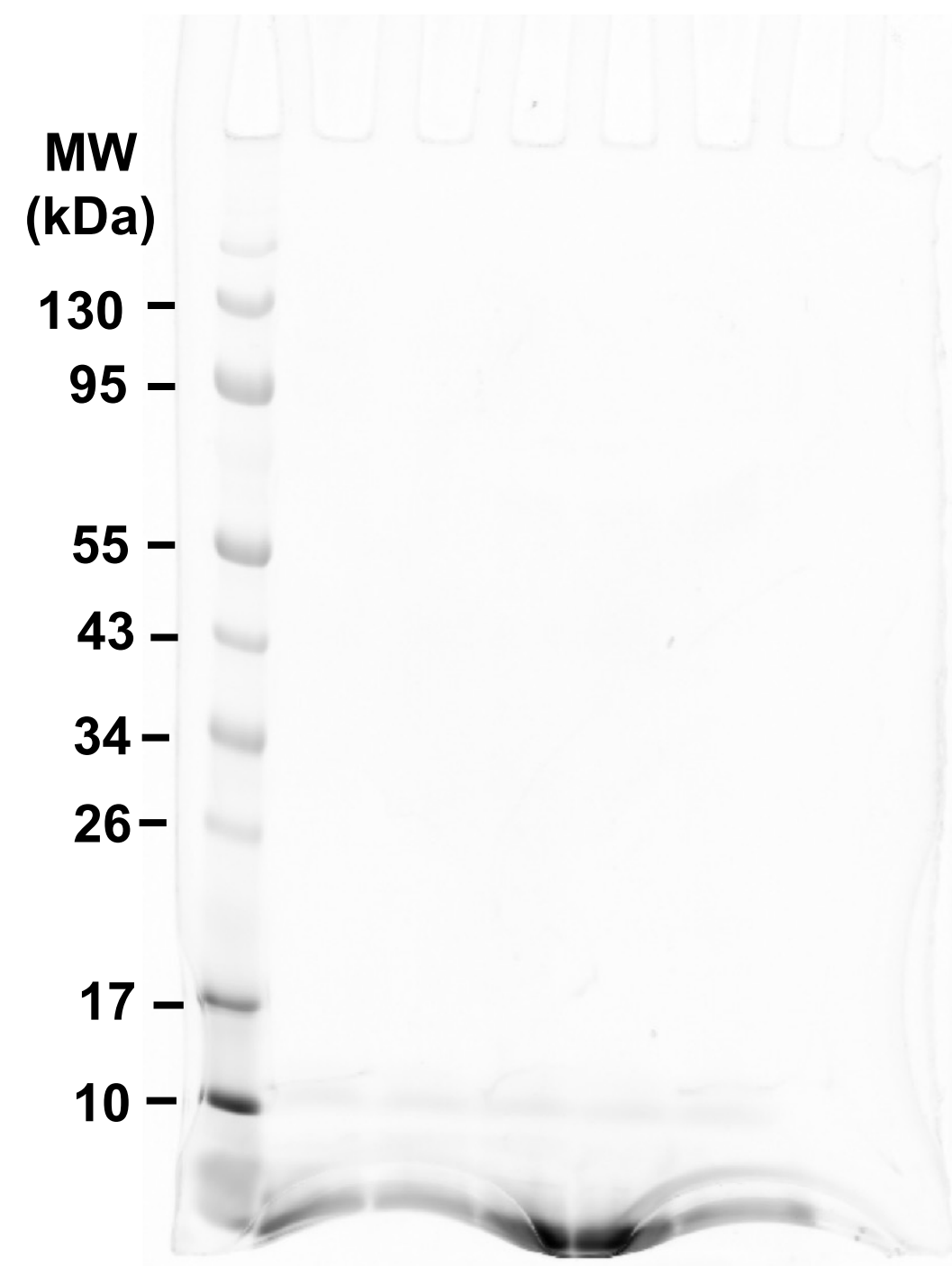

Cy5 scan (visualizes MW marker)

Uncropped fluorescent scans of SDS-PAGE gels for Extended Data Figure 7i

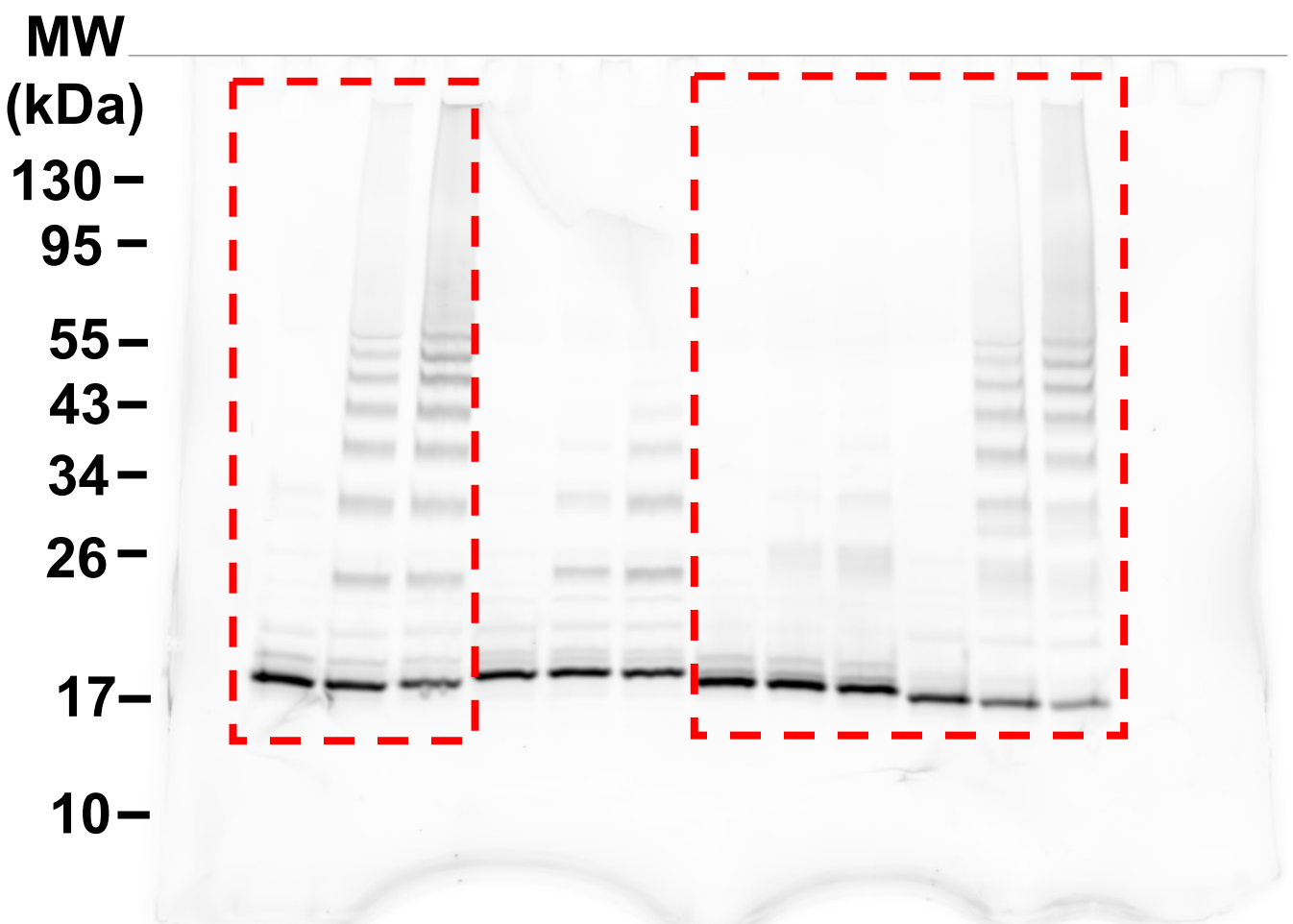

Cy2 scan (visualizes FAM-$BRD4_{BD}$)

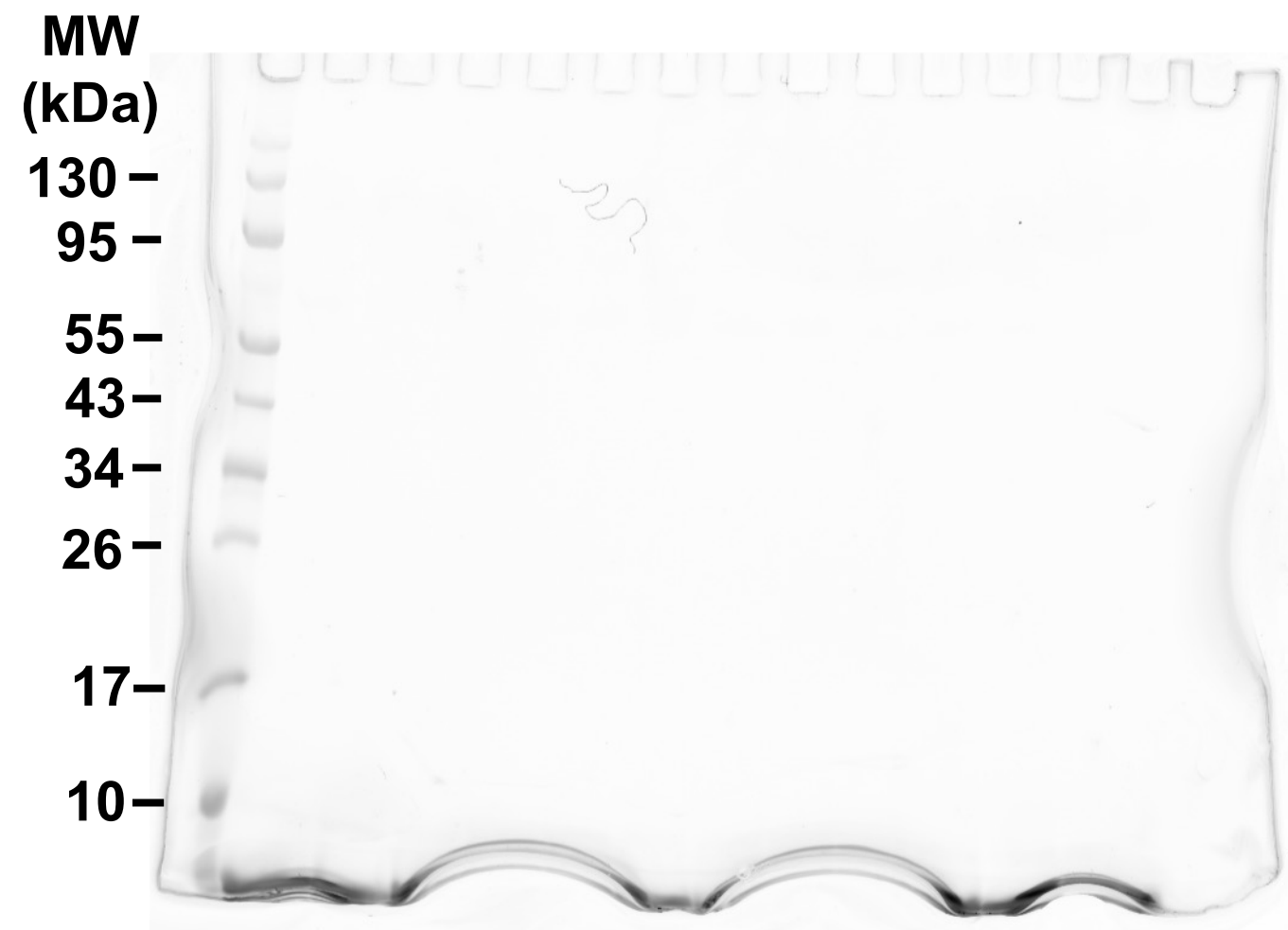

Cy5 scan (visualizes MW marker)

Uncropped fluorescent scans of SDS-PAGE gels for Extended Data Figure 8a

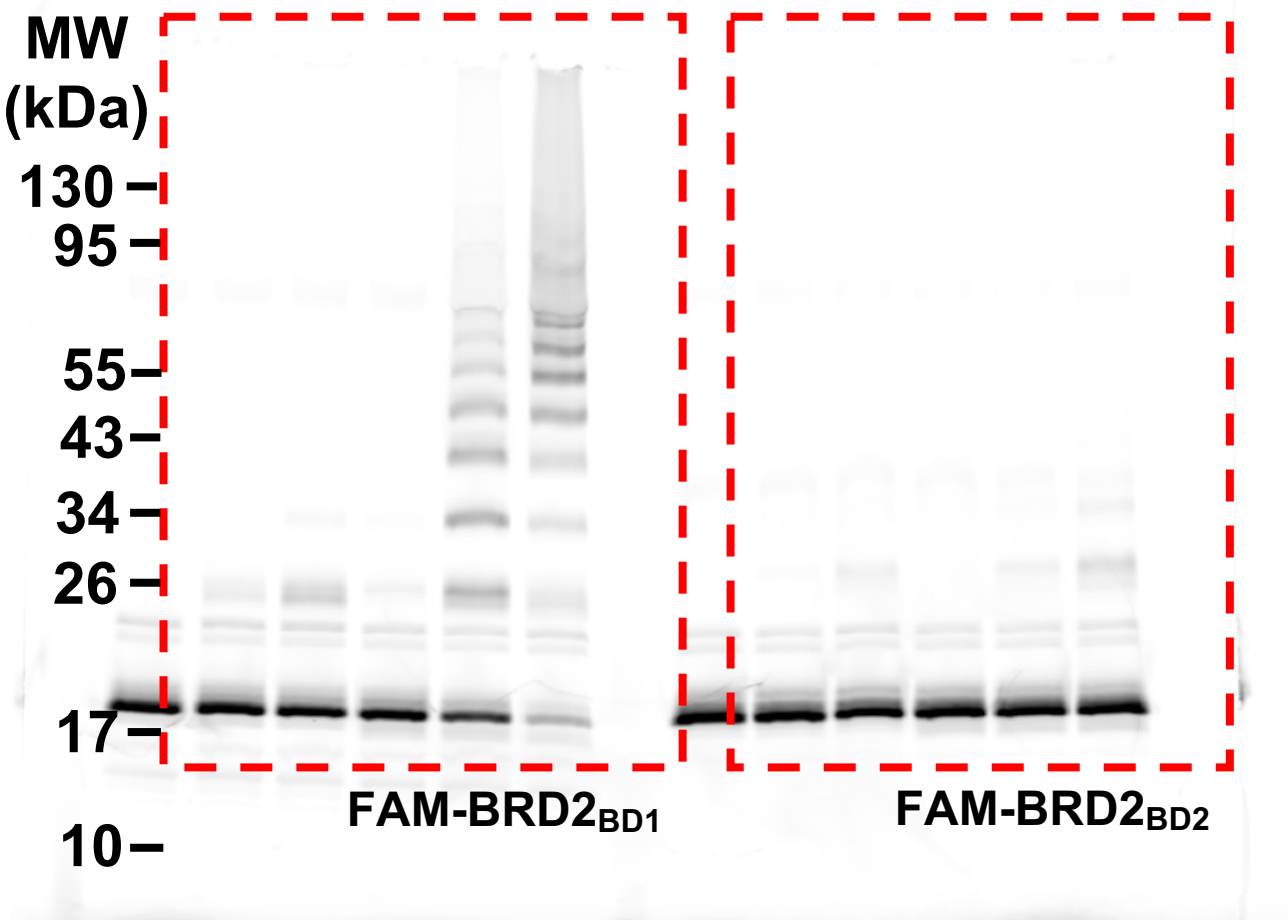

Cy2 scan (visualizes FAM-BRD2$_{BD}$)

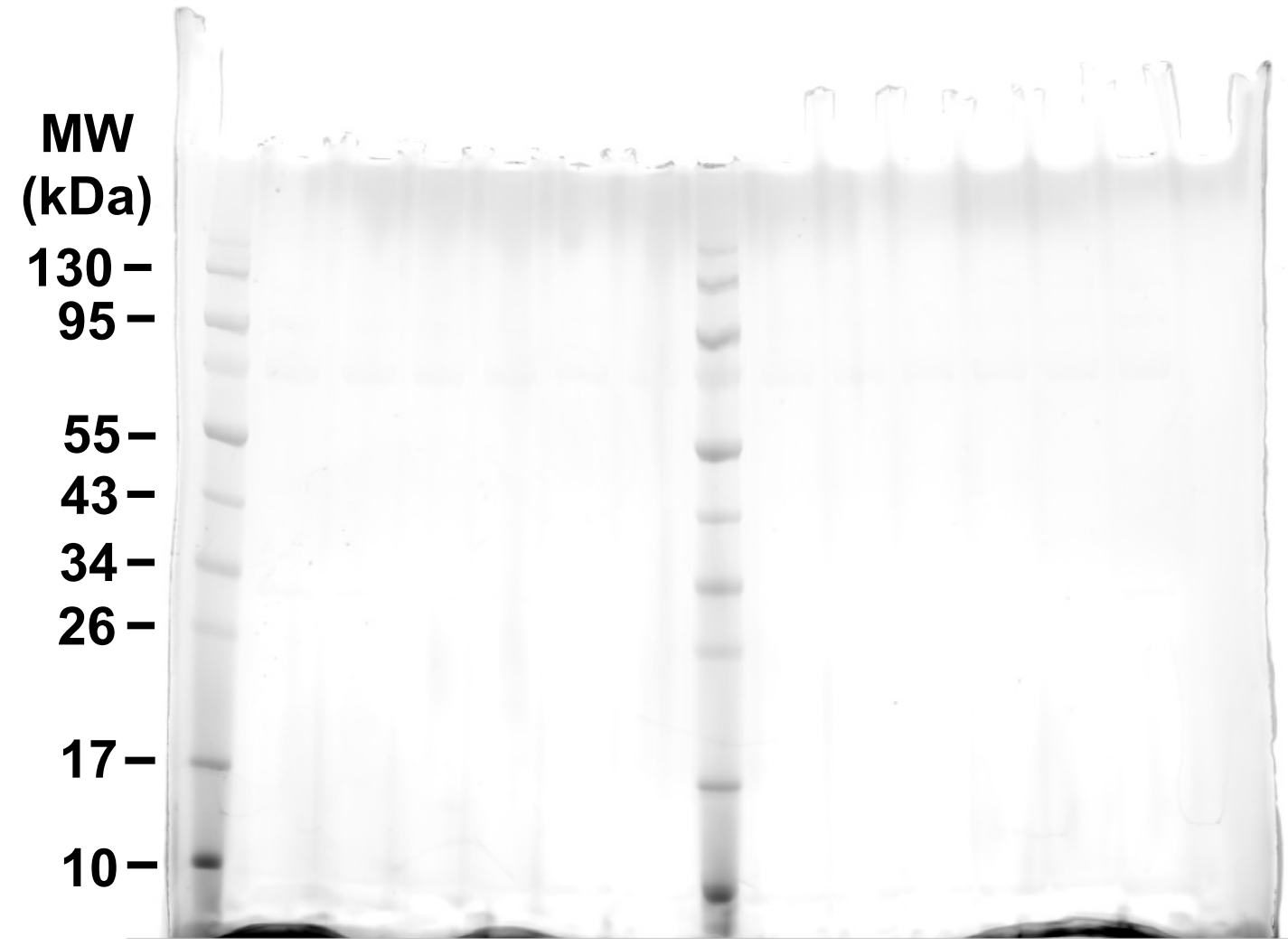

Cy5 scan (visualizes MW marker)

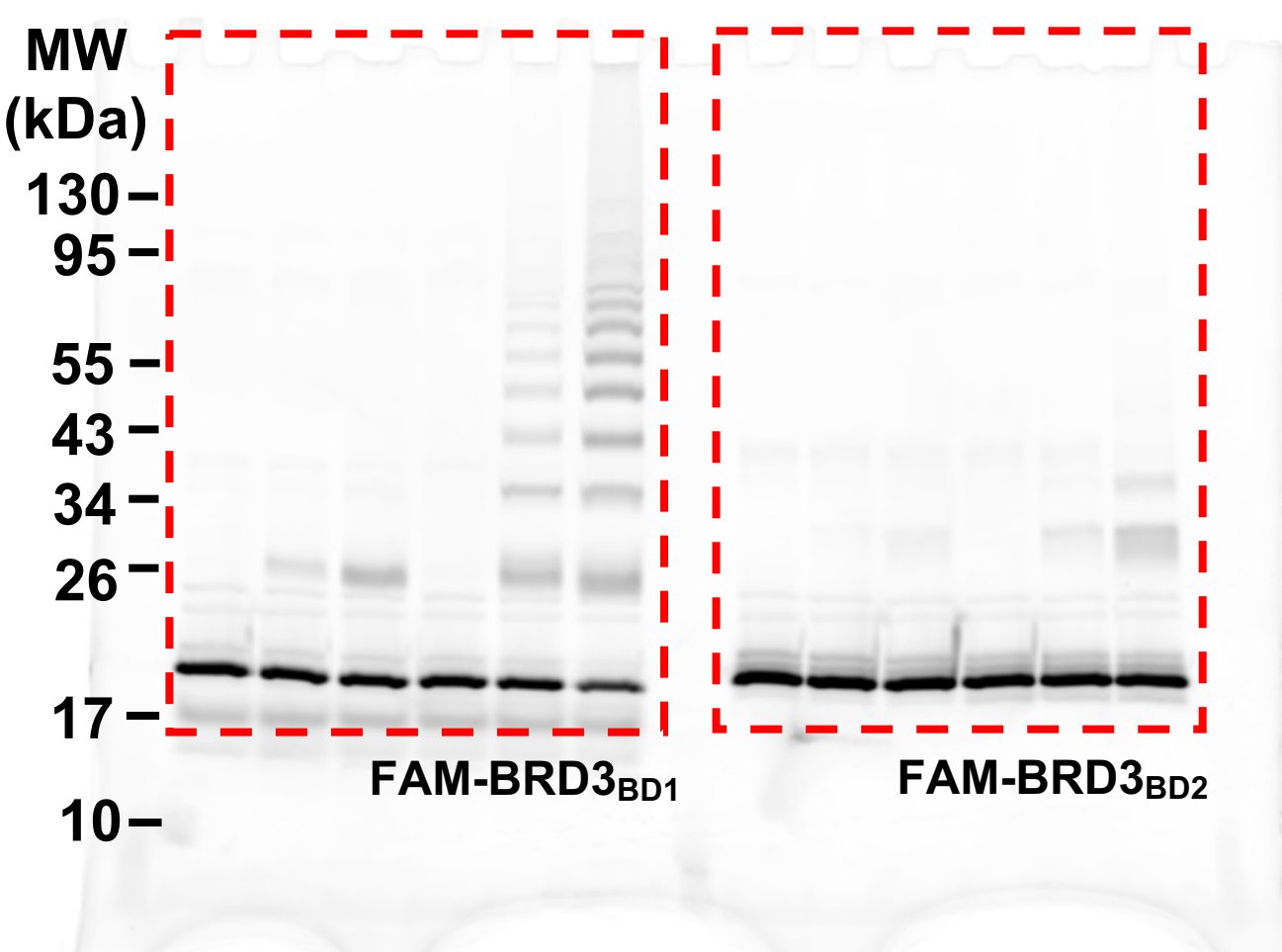

Cy2 scan (visualizes FAM-BRD3$_{BD}$)

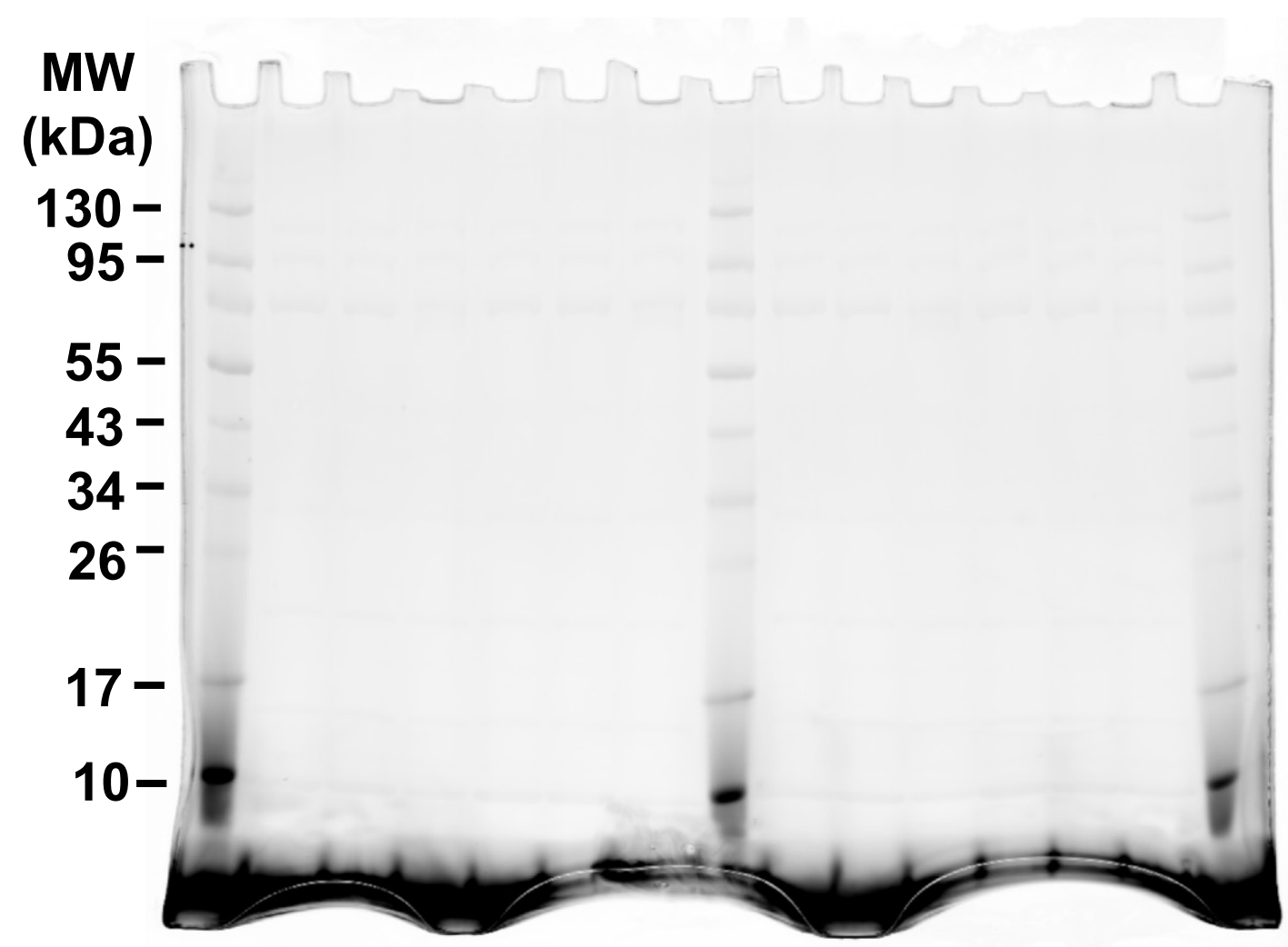

Cy5 scan (visualizes MW marker)

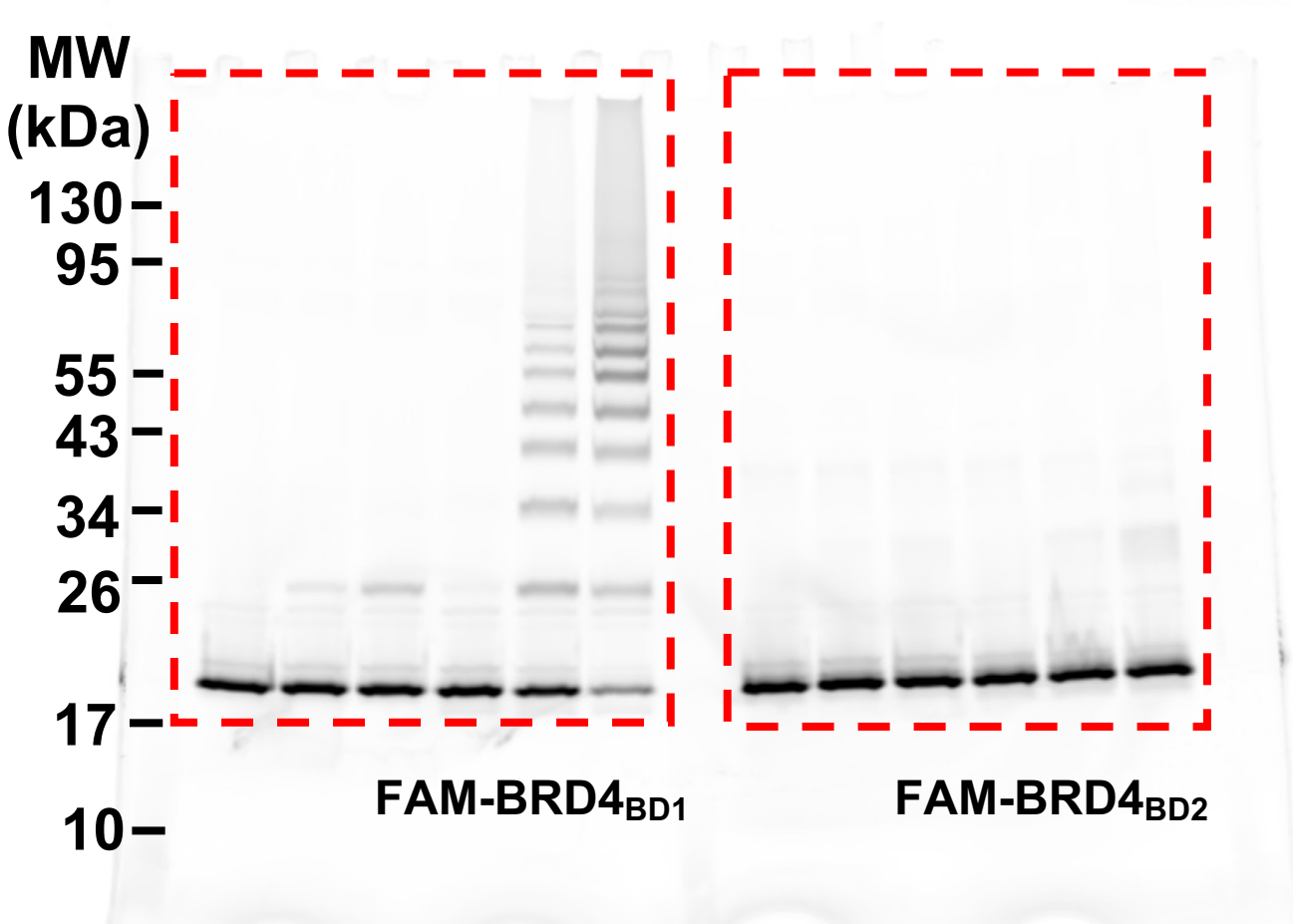

Cy2 scan (visualizes FAM-BRD4$_{BD}$)

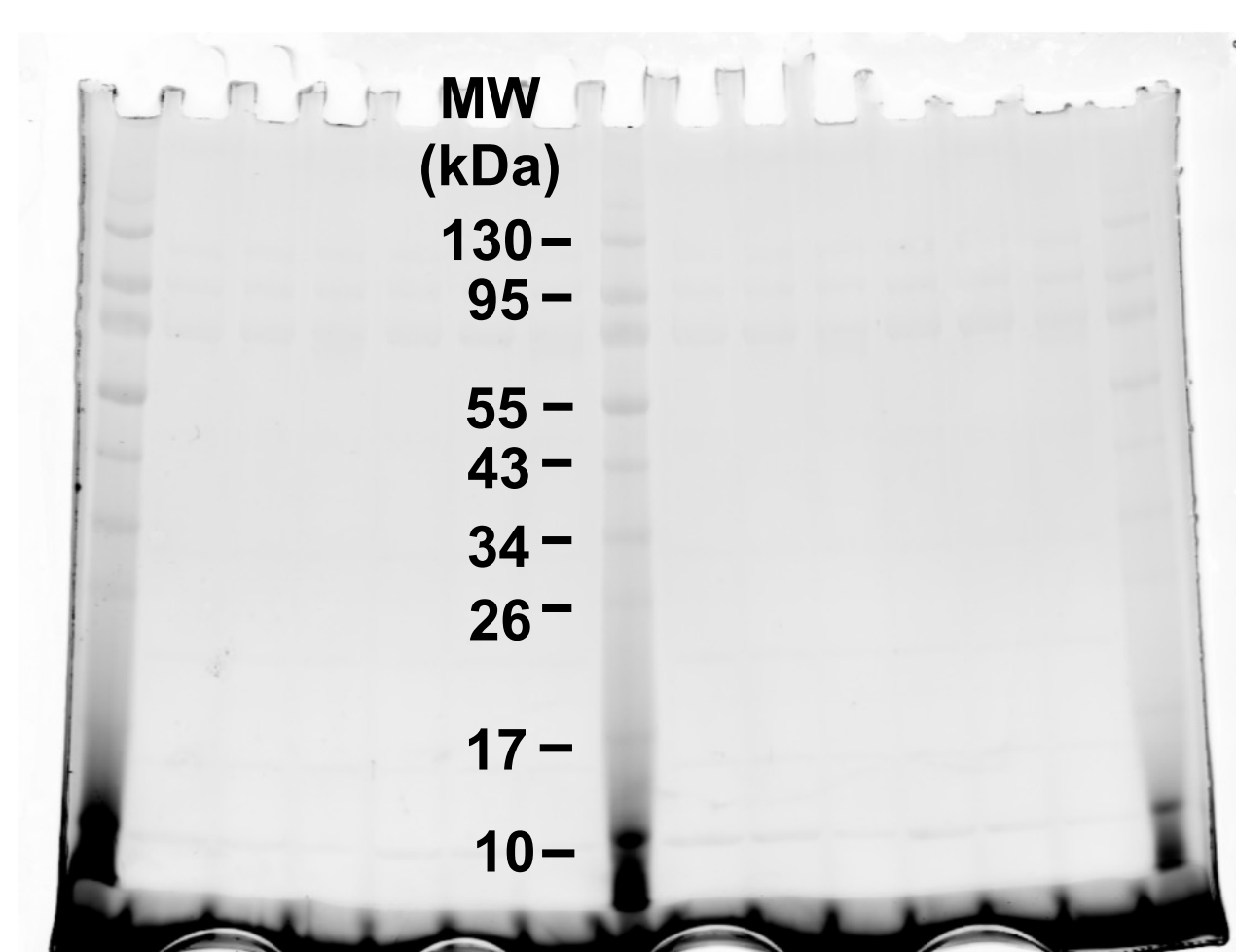

Cy5 scan (visualizes MW marker)

Uncropped Blot for Extended Data Figure 10b

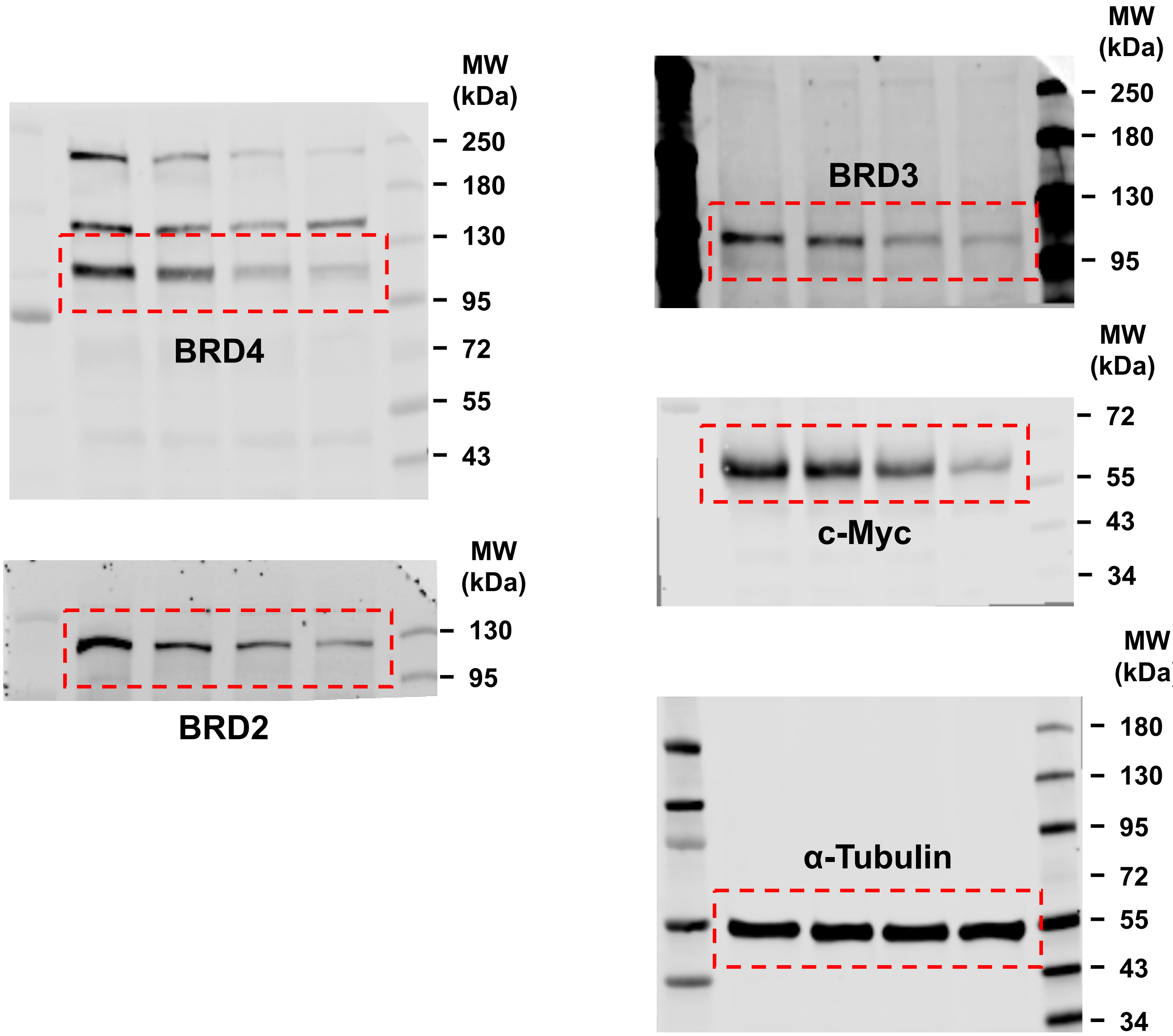

Uncropped Blot for Extended Data Figure 10c

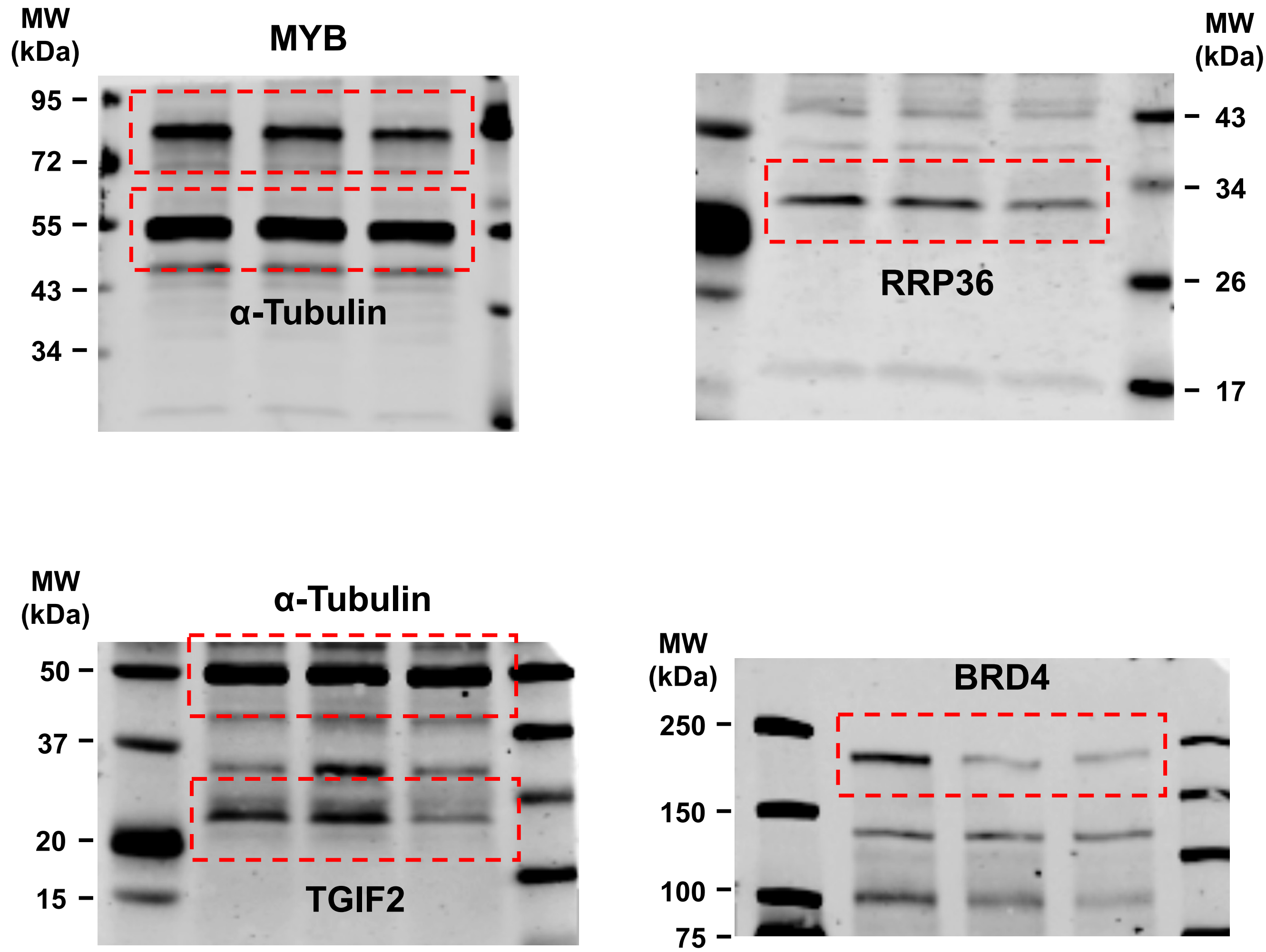

Uncropped Blot for Extended Data Figure 10c

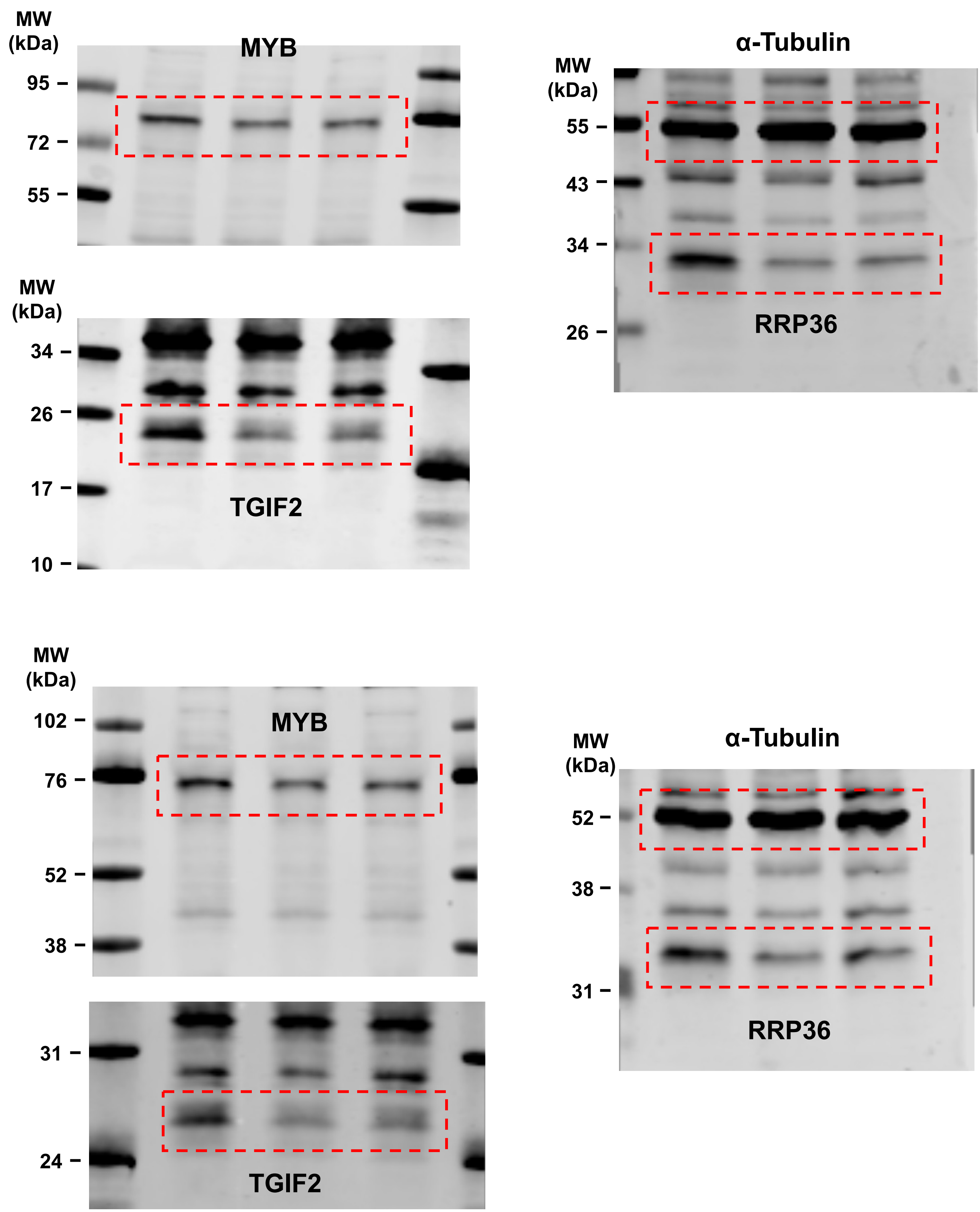

Uncropped fluorescent scans of SDS-PAGE gels for Extended Data Figure 10j

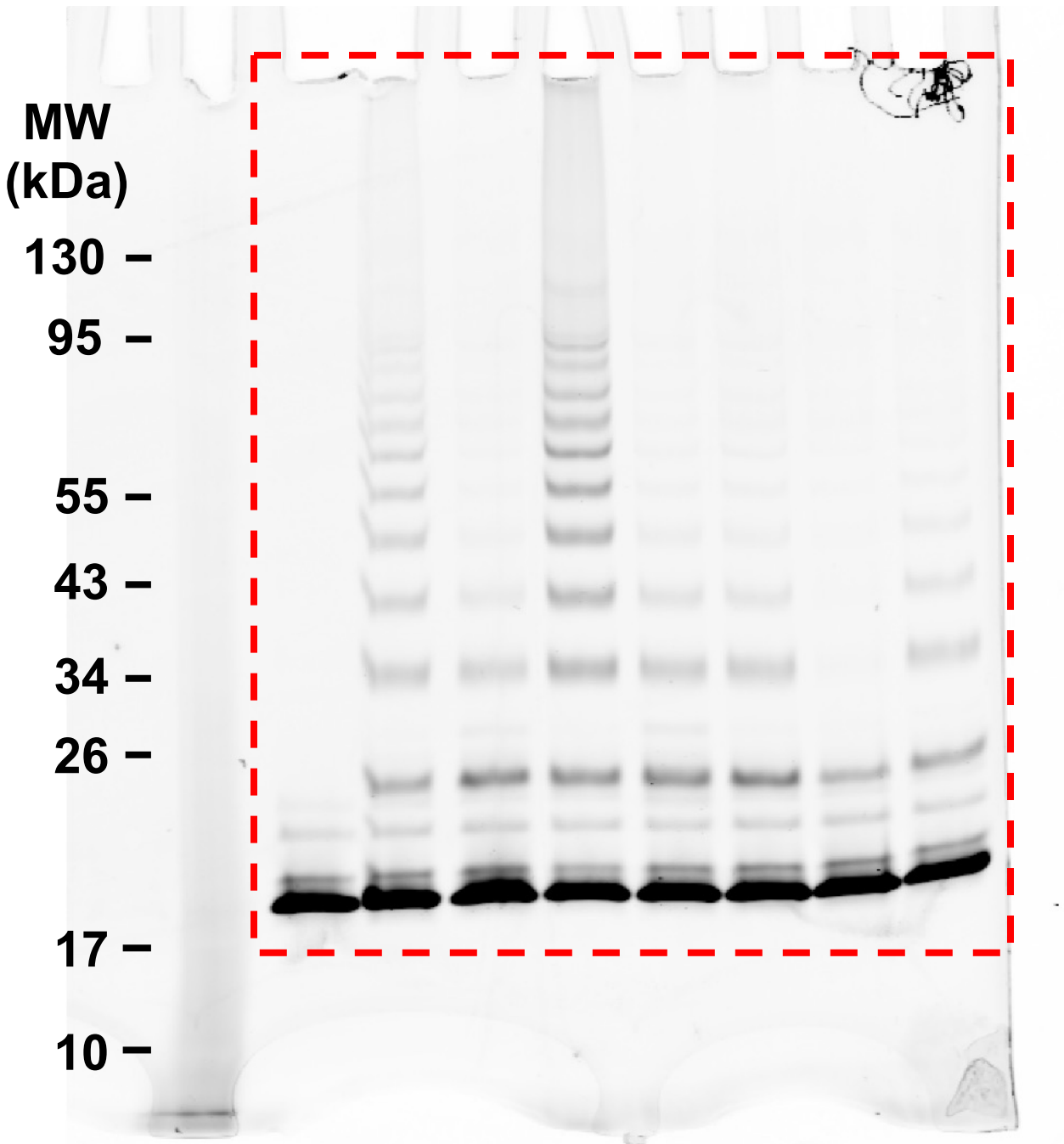

Cy2 scan (visualizes FAM-BRD4$_{BD1}$)

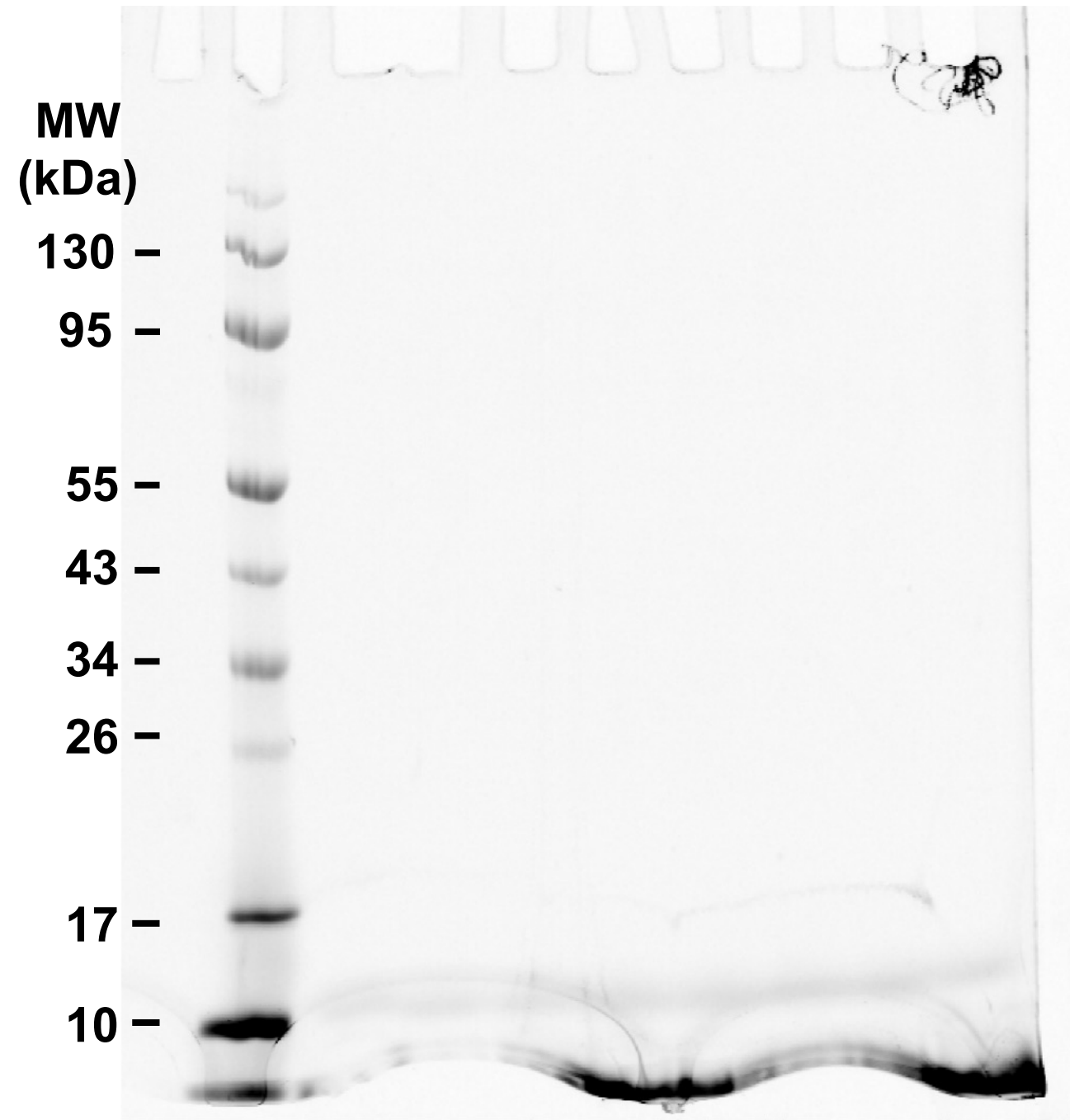

Cy5 scan (visualizes MW marker)
